# Anti-inflammatory and Hepatoprotective Iridoid Glycosides from the Roots of *Gomphandra mollis*

**DOI:** 10.1021/acs.jnatprod.4c01484

**Published:** 2025-02-13

**Authors:** Quoc-Dung Tran Huynh, Thuy-Tien Thi Phan, Ta-Wei Liu, Thanh-Vu Nguyen, Truc-Ly Thi Duong, Su-Jung Hsu, Man-Hsiu Chu, Yun-Han Wang, Bien-Thuy Nguyen Bui, Dang-Khoa Nguyen, Thanh-Hoa Vo, Ching-Kuo Lee

**Affiliations:** † Ph.D. Program in Clinical Drug Development of Herbal Medicine, College of Pharmacy, Taipei Medical University, Taipei 11031, Taiwan; ‡ Institute of Pharmaceutical Education and Research, Binh Duong University, Thu Dau Mot 820000, Binh Duong, Vietnam; § Graduate Institute of Biomedical Materials and Tissue Engineering, College of Biomedical Engineering, Taipei Medical University, Taipei 11031, Taiwan; ∥ School of Pharmacy, College of Pharmacy, Taipei Medical University, Taipei 11042, Taiwan; ⊥ Biotechnology Center of Ho Chi Minh City, Ho Chi Minh City 700000, Vietnam; # Faculty of Traditional Medicine, Can Tho University of Medicine and Pharmacy, Can Tho 900000, Vietnam; g Faculty of Pharmacy, Ton Duc Thang University, Ho Chi Minh City 700000, Vietnam; h National Research Institute of Chinese Medicine, Ministry of Health and Welfare, Taipei 11221, Taiwan; i University of Health Sciences, Vietnam National University Ho Chi Minh City, Ho Chi Minh 700000, Vietnam; k Center for Discovery and Development of Healthcare Product, Vietnam National University Ho Chi Minh City, Ho Chi Minh 700000, Vietnam; l Institute of Fisheries Science, National Taiwan University, Taipei 106, Taiwan; m Graduate Institute of Pharmacognosy, College of Pharmacy, Taipei Medical University, Taipei 11042, Taiwan; n Department of Chemistry, Chung Yuan Christian University, Zhongli District, Taoyuan 32023, Taiwan

## Abstract

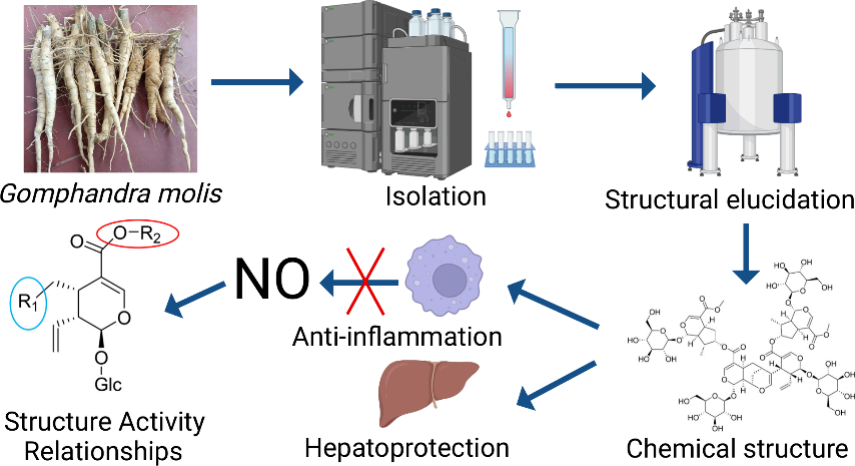

Ten previously undescribed iridoid glycosides (**1**–**10**), including monoiridoids, hybrid iridoid-alkaloids, bis-iridoids, noriridoid–iridoid dimers, and tetramers, were isolated from the roots of *Gomphandra mollis* Merr. Structural elucidation revealed unique sugar chains not previously observed for iridoids and complex tetrameric configurations that are rare in nature. Compounds **9**, **10**, and **15** demonstrated significant anti-inflammatory effects, with IC_50_ values ranging from 6.13 to 13.0 μM, and compounds **6**, **7**, and **11**–**13** showed notable hepatoprotective activity in HepG2 cells. Additionally, structure–activity relationship (SAR) analysis on anti-inflammatory effects was also conducted. This study enriches the structural database of iridoids, particularly complex derivatives, and highlights their therapeutic potential in addressing inflammation-related and liver diseases.

Hepatoprotective and anti-inflammatory agents play a crucial role in mitigating liver damage and managing inflammatory diseases, which are significant global health concerns.^1−4^ Liver injury, often caused by oxidative stress, inflammation, or toxic substances, can lead to severe conditions such as fibrosis, cirrhosis, or hepatocellular carcinoma.^5,6^ Similarly, chronic inflammation, mediated by overproduction of inflammatory mediators such as nitric oxide (NO), is associated with numerous disorders, including arthritis, cardiovascular diseases, and neurodegenerative conditions. NO, although essential in physiological processes, becomes detrimental when produced excessively by activated macrophages during inflammation.^7,8^

Iridoids make up a class of monoterpenoids characterized by their distinctive cyclopentanopyran ring structure. They are commonly found as glycosides (iridoid glycosides) and exhibit a diverse range of biological activities, including anti-inflammatory, hepatoprotective, anticancer, antibacterial, antioxidant, and neuroprotective effects. Most iridoids in nature occur in monomeric forms, with some existing as bis-iridoids. However, trimeric forms and particularly tetrameric forms are rarely observed. Furthermore, β-d-glucose derivatives are the most prevalent, while derivatives of other monosaccharides are rarely identified.^9−12^

*Gomphandra mollis* Merr. is a Vietnamese endemic medicinal plant with sweet, a little bitter, cool, and nontoxic properties. In traditional medicine, the roots of *G. mollis* are used to strengthen the body, increase fluids, nourish the spleen, act as a laxative and diuretic, and promote lactation.^13^ In contrast to the extensive research conducted on other species within the *Gomphandra* genus, which revealed the presence of various classes of natural products, particularly iridoids,^14−16^ no studies on *G. mollis* have been reported to date. In an effort to explore the potential of *G. mollis* as a source of iridoid compounds with hepatoprotective and anti-inflammatory properties, herein we report a chemical investigation focusing on the iridoid class and assess their bioactivities in liver protection and inflammation mitigation.

## Results and Discussion

### Structure Elucidation

By using a combination of various chromatographic techniques, we isolated 18 iridoid glycosides including one new monoiridoid (**1**), one new hybrid iridoid-alkaloid (**2**), one new noriridoid–iridoid dimer (**3**), three new bis-iridoids (**4**–**6**), two new noriridoid–iridoid tetramers (**7**, **8**), two new tetra-iridoids (**9**, **10**), and eight known iridoid glycosides (**11**–**18**), whose structures were identified by comparison of NMR data reported in the literature, including cantleyoside-dimethyl-acetal (**11**),^17^ cantleyoside (**12**),^17^ sweroside (**13**),^18^ 6′-*O*-β-apiofuranosylsweroside (**14**),^19^ dipsanoside A (**15**),^20^ dipsaperine (**16**),^21^ dipsanoside B (**17**),^20^ and loganin (**18**).^22^

All previously reported compounds have been shown to exhibit β-orientation at the C-1 position in the loganin and secologanin moieties.^22−28^ This configuration is further supported by the well-established biosynthetic pathway of iridoids.^29−32^ Consistently, all known compounds isolated from this plant in this study also demonstrated the same stereochemistry at C-1. Thus, we assume that the C-1 position adopts a β-orientation for iridoids from *G. mollis*, which serves as the basis for identifying the relative configuration of compounds **1**–**10**. Additionally, the sugar types in compounds **1**–**10** were identified using the UHPLC-MS method by comparison with reference NAIM-sugar derivatives (Supporting Information S2). The analysis revealed that compound **1** contained d-glucose and d-galactose; compounds **2** and **7**–**10** contained d-glucose; and compounds **3**–**6** contained d-glucose, d-galactose, and d-fructose.

**Figure 1:**
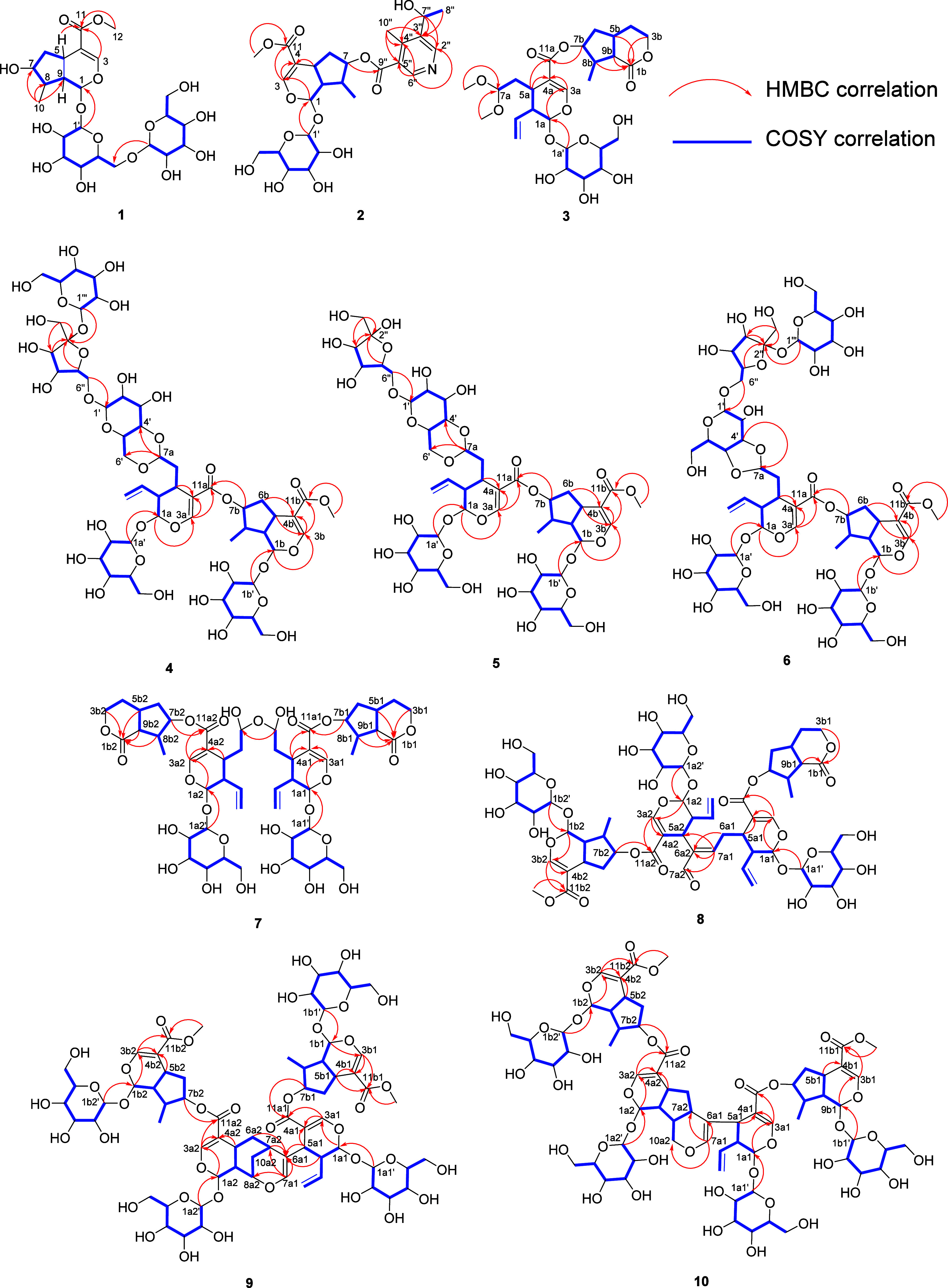
Key COSY and HMBC correlations of compounds **1**–**10**.

**Figure 2:**
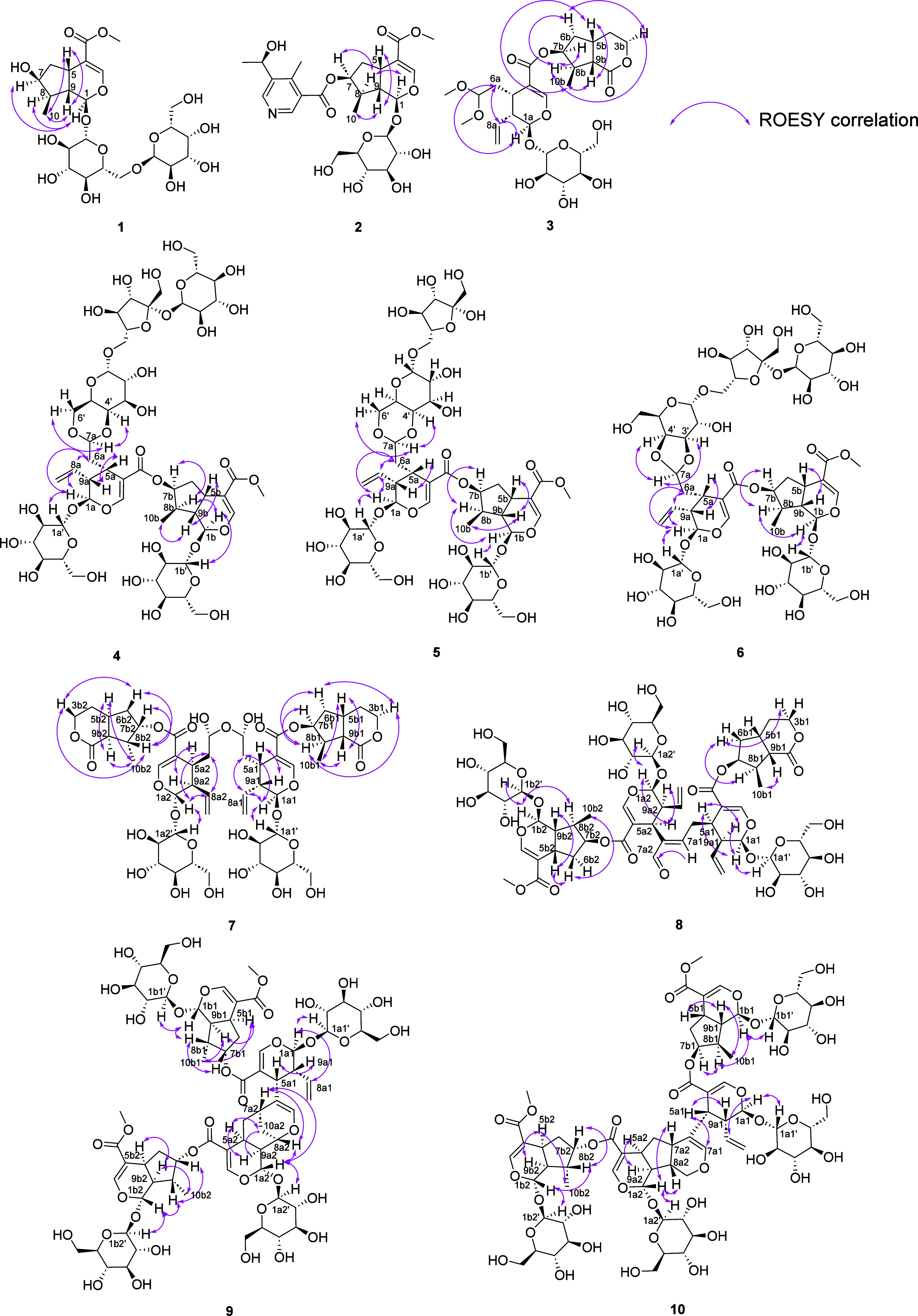
Key ROESY correlations of compounds **1**–**10**.

Compound **1** was isolated as a white amorphous powder. Its molecular formula of C_23_H_36_O_15_ was established by HRESIMS analysis (*m*/*z* 597.20258 [M + HCOO]^−^). The UV spectrum of **1** showed an absorption maxima at 235 nm. Its IR spectrum showed absorption bands consistent with the presence of hydroxy (3370 cm^–1^) and conjugated carbonyl (1666 cm^–1^). ^1^H NMR showed one olefinic proton at δ_H_ 7.39 (d, 1.3 Hz, H-3), one methoxy singlet at δ_H_ 3.69 (s), one methyl doublet at δ_H_ 1.09 (d, 6.9 Hz, H-10), and two anomeric protons at δ_H_ 4.66 (d, 8.0 Hz, Glc-H-1′) and 4.87 (d, 3.6 Hz, Gal-H-1″) with β and α orientation, respectively, as indicated by their coupling constant values. ^13^C NMR and DEPT analysis revealed two quaternary carbons including one ester carbon (δ_C_ 169.5) and one olefinic quaternary carbon (δ_C_ 114.0), one methyl group (δ_*C*_ 13.6), one methoxy carbon (δ_C_ 57.1), three methylene carbons (δ_C_ 42.8, 62.7, 67.9), and 16 methine carbons (δ_C_ 32.3, 42.4, 46.5, 70.4, 71.0, 71.6, 71.8, 72.4, 74.6, 75.0, 76.6, 78.0, 98.2, 100.2, 100.5, 152.2). Analysis of the COSY and HMBC spectra identified compound **1** as an iridoid diglycoside (Figure 1). The ^1^H and ^1^^3^C NMR data (Table 1) were unambiguously assigned by using 1D and 2D NMR spectroscopy. In the ROESY spectrum, the correlations from H-9 (δ_H_ 2.02) to CH_3_ (δ_H_ 1.09) and H-5 (δ_H_ 3.11) confirmed the *cis* configuration of H-9/H-5/CH_3_; the cross peaks from H-8 (δ_H_ 1.87) to H-1 (δ_H_ 5.19) and H-7 (δ_H_ 4.04) to H-1 (δ_H_ 5.19) also confirmed the *cis* orientations of H-1/H-7/H-8 (Figure 2). The *cis* relationship between H-1 and H-7 was further supported by comparing the chemical shift of C-7 (δ_C_ 75.0)^33^ to that of the loganin moiety (δ_C_ 75.0),^33^ indicative of a *cis* configuration, and 7-epiloganin ((δ_C_ 79.7),^34^ indicative of a *trans* configuration. Furthermore, the absolute configuration of compound **1** was determined using DP4+ analysis, which compared the experimental and calculated NMR data (Supporting Information S3). The results indicated a (1*S*,5*S*,7*S*,8*R*,9*S*) configuration, aligning with our assumption of the β-orientation at C-1. In addition, their NMR data were similar to those of loganin 6′-*O*-α-d-glucopyranoside,^35^ except for the substitution of α-d-glucopyranoside with α-d-galactopyranoside. This sugar chain was also identified in another iridoid, phlotuberoside I, which was isolated from *Phlomoides tuberosa*.^36^ From the above analysis, the absolute structure of **1** was identified as loganin 6′-*O*-α-d-galactopyranoside, named gomphandranoside A.

**Table 1:** ^1^H (600 MHz) and ^13^C (150 MHz) NMR Data of Compounds **1** and **2** in CD_3_OD

	Compound **1**			Compound **2**	
No.	*δ*_C_, type	*δ*_H_ (*J* in Hz)	No.	*δ*_C_, type	*δ*_H_ (*J* in Hz)
1	98.2, CH	5.19 d (4.7)	1	97.5, CH	5.33 d (5.1)
3	152.2, CH	7.39 d (1.3)	3	152.3, CH	7.46 d (1.3)
4	114.0, C		4	113.0, C	
5	32.3, CH	3.11 qd (7.9,1.3)	5	32.8, CH	3.19 ovl
6	42.8, CH_2_	2.24 ddd (14.0, 7.9, 1.6);	6	40.5, CH_2_	1.88 ddd (14.7, 8.1, 5.1);
		1.58 ddd (14.0, 7.9, 5.0)			2.45 ddd (14.7, 7.7, 1.6)
7	75.0, CH	4.04 td (5.0, 1.6)	7	80.1, CH	5.44 td (5.1, 1.6)
8	42.4, CH	1.87 m	8	41.1, CH	2.27 m
9	46.5, CH	2.02 td (9.1, 4.8)	9	47.1, CH	2.13 td (8.8, 5.1)
10	13.6, CH_3_	1.09 d (6.9)	10	14.0, CH_3_	1.15 d (6.9)
11	169.5, C		11	169.3, C	
12	57.1, CH_3_	3.69 s	12	51.8, CH_3_	3.70 s
β-d-glucose					
1′	100.5, CH	4.66 d (8.0)	1′	100.2, CH	4.68 d (7.9)
2′	74.6, CH	3.21 dd (9.0, 8.0)	2′	74.7, CH	3.21 dd (9.3, 7.9)
3′	78.0, CH	3.38 t (9.0)	3′	78.0, CH	3.38 t (9.3)
4′	71.8, CH	3.34 ovl	4′	71.6, CH	3.27 dd (9.3, 8.7)
5′	76.6, CH	3.54 ddd (9.6, 6.0, 2.2)	5′	78.4, CH	3.32 ovl
6′	67.9, CH_2_	3.90 dd (11.0, 6.0)	6′	62.8, CH_2_	3.66 dd (12.0, 6.2)
		3.76 ovl			3.91 dd (12.0, 2.2)
α-d-galactose			2″	149.5, CH	8.77 s
1″	100.2, CH	4.87 d (3.6)	3″	128.9, C	
2″	70.4, CH	3.75 m	4″	147.2, C	
3″	71.6, CH	3.73 ovl	5″	142.8, C	
4″	71.0, CH	3.88 dd (3.1, 1.3)	6″	150.0, CH	8.76 s
5″	72.4, CH	3.85 td (6.0, 1.3)	7″	65.8, CH	5.22 d (6.5)
6″	62.7, CH_2_	3.69 ovl	8″	24.3, CH_3_	1.48 d (6.5)
			9″	167.3, C	
			10″	15.8, CH_3_	2.59 s

Compound **2** was obtained as a white amorphous powder with the molecular formula C_26_H_35_NO_12_ deduced by HRESIMS analysis (*m*/*z* 598.21350 [M + HCOO]^−^); *m*/*z* 554.22278 [M + H]^+^). Its NMR data closely resemble those of the loganin unit^17,22^ and alstochonine A,^37^ but differ in the chemical shifts of C-7 and C-9″, suggesting that **2** contains a combination of loganin and alstochonine A linked by an ester bridge at C-7 and C-9″. This conclusion was confirmed by the HMBC correlation of H-7 (δ_H_ 5.44)/C-9″ (δ_C_ 167.3) (Figure 1). The relative configuration of the loganin unit was analyzed in a manner similar to that of compound **1**, with the key difference being at position C-7, which exhibited esterification to C-9′. This modification resulted in a chemical shift to δ_C_ 80.1, compared to δ_C_ 75.0 observed for compound **1** (Figure 2). To determine the absolute configurations of C-7 and C-7″, NMR calculations and DP4+ analysis were performed, which indicated a 7*S*, 7″*R* configuration (Supporting Information S4). Furthermore, with the assumption of a β-oriented C-1, the remaining stereochemistries were assigned accordingly. Based on the above analysis, the absolute structure of compound **2** was thus determined and named gomphandranoside B.

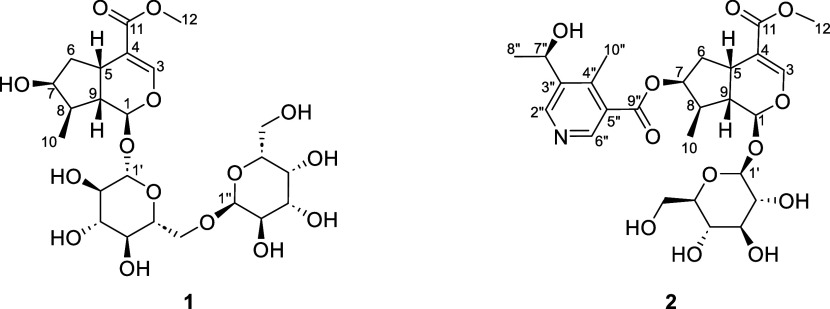



Compound **3** was isolated as white amorphous solid. Its molecular formula C_27_H_40_O_13_ was established by HRESIMS analysis (*m*/*z* 617.24408 [M + HCOO]^−^). By comparing the ^13^C NMR data of **3** to that of secologanin dimethyl acetal^38,39^ and boonein,^40^ the structure of **3** was suggested to be an iridoid dimeric glycoside, incorporating the two aforementioned moieties. The conjugate ester linkage was confirmed by the HMBC correlation of H-7b (δ_H_ 5.22)/C-11a (δ_C_ 168.1) (Figure 1). For the relative configuration of the secologanin dimethyl acetal moiety, the ROESY correlations among H-1a, H-8a, and H-6a indicated their *cis* configuration (Figure 2). Based on this, the assumption of a β-oriented C-1 suggested the absolute configuration of the secologanin dimethyl acetal moiety. For the boonein moiety, based on the key ROESY correlations shown in Figure 2 and the close agreement of the ^13^C NMR data of compound **3** with those of boonein,^40^ except for C-7b position, the absolute configuration of this moiety was established. Besides, for the absolute configuration of C-7b, the ROESY correlation from H-7b to H-8b and H-6b (Figure 2) along with the DP4+ analysis (Supporting Information S5) confirmed the 7b-*S* orientation. From the above analysis combined with the β-oriented C-1a assumption, the absolute structure of **3** was established and named gomphandranoside C.
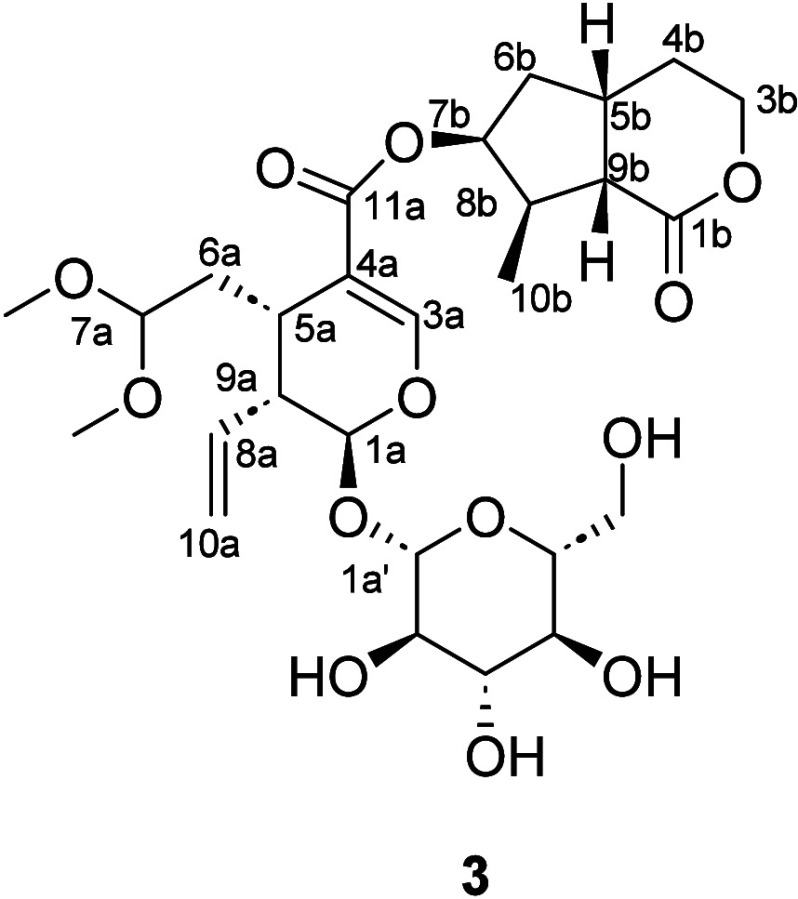


Compound **4** was isolated as white amorphous powder. Its molecular formula, C_51_H_76_O_34_, was deduced by HRESIMS analysis (*m*/*z* 1277.41711 [M + HCOO]^−^). Analysis of 1D and 2D data enabled the assignment of ^1^H and ^13^C NMR resonances of **4** (Table 2). The close agreement of ^13^C NMR data of **4** to those of cantleyoside-dimethyl-acetal,^38,39^ except for replacing two methoxy groups in the glycosidic chain, suggested **4** to be a cantleyoside-dimethyl-acetal derivative. UPLC-MS sugar analysis (Supporting Information S2) indicated that compound **4** contained d-glucose, d-galactose, and d-fructose. In the glycoside chain, the coupling constant of the anomeric hydrogens of gal-*J*_H1,H2_ = 3.6 Hz and glc-*J*_H1,H2_ = 3.6 Hz indicated α orientations for both. Comparing the C-3 chemical shift (δ_C_ 78.8) to that of α-fructofuranose (δ_C_ 84.2) and β-fructofuranose (δ_C_ 76.84)^41^ suggested a β-fructofuranoside configuration. In addition, glycoside chain linkages and the connection to the bis-iridoid glycoside moiety were established based on HMBC correlations of H-7a/C-4′, C-6′; H-6″/C-1′; and H-1‴/C-2″ (Figure 1). ROESY correlations between H-7a/H-4′ and H-7a/H-6′ indicated the *cis* orientation of H-7a relative to those of both H-4′ and H-6′. For the remaining bis-iridoid moiety, the absolute configuration was suggested based on the close agreement of the ^13^C NMR data of compound **4** with those of cantleyoside-dimethyl-acetal,^38,39^ as well as similar ROESY analysis as **1**–**3** (Figure 2) and the assumption of β-orientations for both C-1a and C-1b. From the above analysis, the absolute structure of compound **4** was determined and named gomphandranoside D.

**Table 2:** ^1^H (600 MHz) and ^13^C (150 MHz) NMR Data of Compound **3** in CD_3_OD

	Compound **3**	
No.	*δ*_C_, type	*δ*_H_ (*J* in Hz)
1a	97.8, CH	5.52 d (5.4)
3a	153.2, CH	7.46 d (1.3)
4a	112.0, C	
5a	29.4, CH	2.92 dtd (8.4, 5.7, 1.3)
6a	33.3, CH_2_	1.64 ovl
		2.08 ovl
7a	104.4, CH	4.51 dd (7.1, 4.5)
8a	135.8, CH	5.74 ddd (17.3, 10.4, 8.9)
9a	45.4, CH	2.69 dt (8.9, 5.4)
10a	119.8, CH_2_	5.27 dd (10.4, 1.8)
		5.31 dd (17.3, 1.8)
11a	168.1, C	
OMe	52.9, CH_3_	3.30 s
OMe	53.8, CH_3_	3.30 s
1a′	100.1, CH	4.68 d (7.9)
2a′	74.6, CH	3.20 dd (9.2, 7.9)
3a′	78.1, CH	3.36 dd (9.3, 8.5)
4a′	71.6, CH	3.27 dd (9.7, 8.5)
5a′	78.4, CH	3.30 ovl
6a′	62.8, CH	3.67 dd (12.0, 6.0);
		3.90 dd (12.0, 2.1);
1b	177.3, C	
3b	68.5, CH_2_	4.37 ddd (11.2, 6.1, 3.0);
		4.28 ddd (11.2, 9.0, 2.4);
4b	30.4, CH_2_	1.52 m
		2.11 ovl
5b	35.4, CH	2.85 ovl
6b	39.7, CH_2_	1.64 ovl;
		2.14 ddd (14.0, 7.6, 1.2)
7b	79.3, CH	5.22 t (4.2)
8b	43.9, CH	2.42 m
9b	49.0, CH	2.78 dd (11.3, 10.2)
10b	14.6, CH_3_	1.16 d (6.8)

Compound **5** was obtained as a white amorphous powder and presented the molecular formula C_45_H_66_O_29_ by HRESIMS analysis (*m*/*z* 1115.36523 [M + HCOO]^−^). By comparing MS and ^13^C NMR data of **5** to those of **4**, as well as key COSY and HMBC correlations (Figure 1), the structure of **5** was identified to have one less glucose unit relative to **4**. Based on the same key ROESY correlation analysis of **4** as those of **5** (Figure 2), combined with the β-oriented C-1a and C-1b assumption, the absolute structure of **5** was established, and it was named gomphandranoside E.

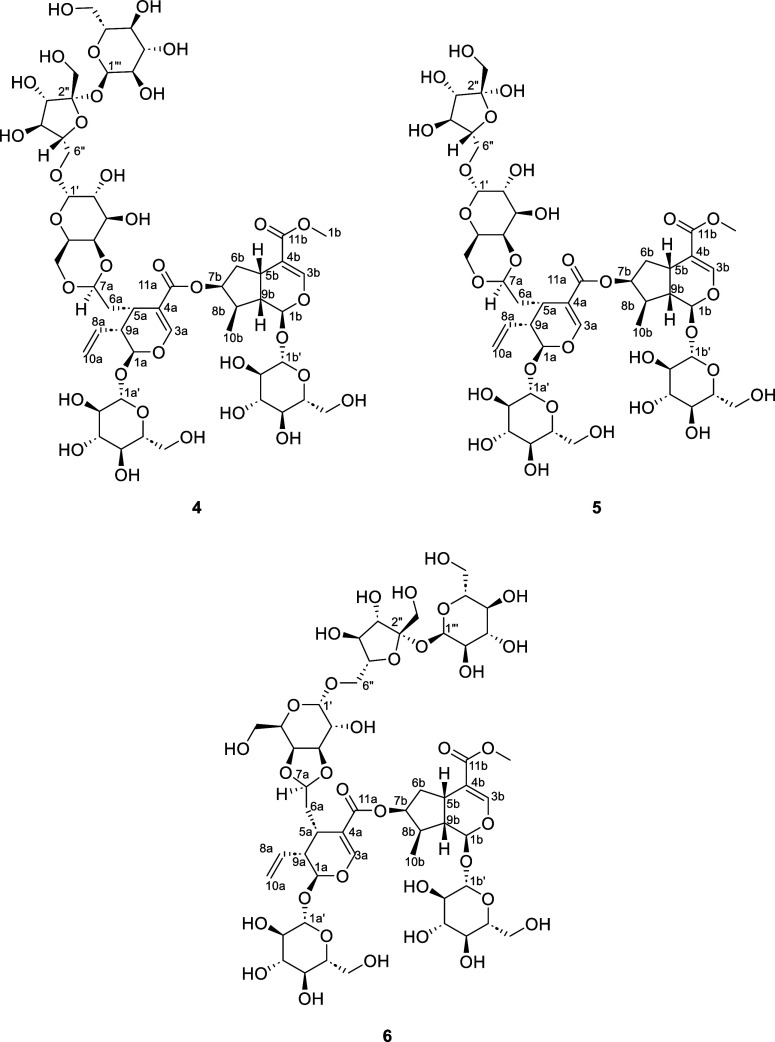



Compound **6** was isolated as a white amorphous powder. Its molecular formula C_51_H_76_O_34_ was deduced by HRESIMS analysis (*m*/*z* 1277.41797 [M + HCOO]^−^), identified as an isomer of **4**. The ^13^C NMR spectrum of compound **6** closely resembled that of compound **4**, with two key exceptions: an upfield shift of C-6′ (from 69.4 to 62.7) and a downfield shift of C-3′ (from 70.0 to 76.8). These shifts suggest a difference in the linkage between the bis-iridoid moiety and the glycoside chain in compound **6** compared to compound **4**. In compound **4**, the connection occurs through oxygen bridges at C-4′ and C-6′ to C-7a, whereas in compound **6**, the linkage involves C-4′ and C-3′ to C-7a. Furthermore, HMBC correlation of H-3′/C-7a and ROESY correlation of H-7a/H-4′ provided additional evidence for the ether linkage for C-7a to C-3′ and C-4′. Besides, ROESY cross peaks from H-7a to H-3′ and H-4′ also confirm the α orientation of H-7a (Figure 2). Besides the sugar chain, the key ROESY and ^13^C NMR of the remaining bis-iridoid was determined to be the same as compounds **4**, **5**, and cantleyoside-dimethyl-acetal^38,39^ (Figure 2, Table 3). These data, combined with the assumption of β**-**oriented C-1a and C-1b, enabled the absolute structure assignment of **6**, named gomphandranoside F.

**Table 3:** ^1^H (600 MHz) and ^13^C (150 MHz) NMR Data of Compounds **4**–**6** in CD_3_OD

	Compound **4**		Compound **5**		Compound **6**	
No.	*δ*_C_, type	*δ*_H_ (*J* in Hz)	*δ*_C_, type	*δ*_H_ (*J* in Hz)	*δ*_C_, type	*δ*_H_ (*J* in Hz)
1b	97.8, CH	5.27 d (4.8)	97.7, CH	5.28 d (4.6)	97.3, CH	5.33 d (4.4)
3b	152.6, CH	7.45 d (1.3)	152.5, CH	7.44 d (1.3)	152.6, CH	7.42 d (1.2)
4b	113.3, C		113.4, C		113.5, C	
5b	32.8, CH	3.16 td (8.0, 6.7)	32.7, CH	3.18 ovl	32.3, CH	3.10 q (6.4)
6b	40.5, CH_2_	2.32 ddd (14.4, 7.6, 1.6);	40.5, CH_2_	2.31 ddd (14.5, 7.7, 1.6);	40.4, CH_2_	2.32 ddd (14.4, 7.6, 1.6)
		1.73 m		1.73 ddd (14.5, 8.1, 5.0)		1.73 m
7b	78.4, CH	5.17 td (4.8, 1.6)	78.4, CH	5.18 m	78.4, CH	5.17 td (4.8, 1.6)
8b	41.0, CH	2.14 ovl	41.0, CH	2.14 ovl	41.0, CH	2.11 ovl
9b	47.0, CH	2.13 ovl	47.0, CH	2.14 ovl	47.0, CH	2.23 td (9.2, 4.4)
10b	14.0, CH_3_	1.09 d (6.4)	13.8, CH_3_	1.09 d (6.5)	14.0, CH3	1.08 d (6.9)
11b	169.4, C		169.4, C		169.4, C	
OMe	51.8, CH_3_	3.70 s	51.8, CH_3_	3.70 s	51.8, CH_3_	3.70 s
1b′	100.2, CH	4.68 d (7.9)	100.2, CH	4.67 d (7.9)	100.2, CH	4.66 d (7.9)
2b′	74.7, CH	3.21 ovl	74.7, CH	3.20 ovl	74.7, CH	3.22 ovl
3b′	78.0, CH	3.37 ovl	78.0, CH	3.37 ov)	78.0, CH	3.37 t (8.9)
4b′	71.6, CH	3.29 ovl	71.6, CH	3.29 ov)	71.7, CH	3.30 ovl
5b′	78.4, CH	3.32 ovl	78.4, CH	3.31 ovl	78.4, CH	3.31 ovl
6b′	62.8, CH_2_	3.90 ovl	62.8, CH_2_	3.90 ovl	62.6, CH_2_	3.90 ovl
		3.67 ovl		3.67 ovl		3.68 ovl
1a	97.7, CH	5.54 d (6.0)	97.6, CH	5.54 d (6.2)	97.7, CH	5.58 d (6.0)
3a	153.3, CH	7.45 d (1.1)	153.3, CH	7.45 d (1.1)	153.3, CH	7.48 d (1.0)
4a	111.9, C		112.0, C		111.7, C	
5a	29.8, CH	3.07 dt (7.7, 5.5)	29.9, CH	3.06 dt (7.7, 5.5)	29.7, CH	3.08 m
6a	36.7, CH_2_	1.83 m;	35.9, CH_2_	1.84 ddd (13.9, 8.4, 3.5);	36.1, CH_2_	1.94 m;
		2.11 ovl		2.09 ovl		2.08 ovl
7a	101.7, CH	4.75 dd (7.1, 3.7)	101.8, CH	4.75 dd (7.0, 3.5)	105.0, CH	5.09 t (4.9)
8a	136.0, CH	5.78 ddd (17.3, 10.5, 8.7)	136.0, CH	5.79 ddd (17.4, 10.5, 8.7)	135.7, CH	5.77 ddd (17.3, 10.5, 8.4)
9a	45.3, CH	2.72 dt (8.7, 5.6)	45.4, CH	2.72 dt (8.7 5.6)	45.3, CH	2.72 m
10a	120.0, CH_2_	5.25 dd (10.5, 1.8)	119.7, CH_2_	5.25 dd (10.5, 1.9)	119.7, CH_2_	5.28 m
		5.33 ovl		5.32 ddd (17.4, 1.9)		5.33 m
11a	168.4, C		168.4, C		168.4, C	
1a′	100.1, CH	4.70 d (7.9)	100.1, CH	4.70 d (7.9)	100.0, CH	4.70 d (7.9)
2a′	74.7, CH	3.21 ovl	74.6, CH	3.20 ovl	74.7, CH	3.22 ovl
3a′	78.0, CH	3.37 ovl	78.0, CH	3.37 ovl	78.0, CH	3.37 t (8.9)
4a′	71.6, CH	3.29 ovl	71.6, CH	3.29 ovl	71.5, CH	3.30 ovl
5a′	78.4, CH	3.32 ovl	78.4, CH	3.31 ovl	78.4, CH	3.31 ovl
6a′	62.8, CH_2_	3.90 ovl;	62.8, CH_2_	3.90 ovl;	62.8, CH_2_	3.90 ovl;
		3.67 ovl		3.67 ovl		3.68 ovl
1′	100.9, CH	4.87 ovl	100.9, CH	4.90 d (3.6)	100.2, CH	4.80 d (3.6)
2′	70.1, CH	3.77 dd (10.1, 3.6)	70.2, CH	3.78 dd (10.1, 3.6)	72.5, CH	3.57 dd (7.6, 3.6)
3′	70.0, CH	3.86 dd (10.2, 3.6)	70.1, CH	3.86 dd (10.2, 3.6)	76.8, CH	4.18 dd (7.6, 5.9)
4′	77.5, CH	3.99 ovl	77.5, CH	3.98 ovl	77.0, CH	4.09 dd (5.9, 3.0)
5′	64.6, CH	3.75 brs	64.6, CH	3.71 ovl	69.6, CH	4.17 ovl
6′	69.4, CH	4.06 ovl;	69.4, CH	3.99 ovl;	62.7, CH	3.78 ovl
		3.81 ovl		3.81 ovl		
1″	63.9, CH_2_	3.61 d (12.4);	64.5, CH_2_	3.45 d (11.7);	63.8, CH_2_	3.62 s
		3.60 d (12.4)		3.44 d (11.7)		
2″	105.3, C		103.3, C		105.5, C	
3″	78.8, CH	4.08 d (8.5)	77.1, CH	4.05 d (7.9)	78.8, CH	4.10 d (8.2)
4″	76.6, CH	3.99 t (8.5)	77.0, CH	4.11 dd (7.9, 7.1)	76.7, CH	4.00 t (8.2)
5″	81.5, CH	3.91 ovl	81.2, CH	3.83 ovl	81.4, CH	3.96 m
6″	70.4, CH	4.05 ovl;	69.8, CH_2_	4.99 ovl;	70.9, CH_2_	3.67 ovl;
		3.65 ovl		3.62 ovl		4.10 ovl
1‴	93.3, CH	5.32 d (3.9)			93.4, CH	5.40 d (3.8)
2‴	73.3, CH	3.40 dd (9.7, 3.8)			73.2, CH	3.43 dd (9.8, 3.8)
3‴	74.6, CH	3.70 ovl			74.7, CH	3.69 ovl
4‴	71.8, CH	3.27 ovl			71.6, CH	3.31 ovl
5‴	74.2, CH	3.82 ovl			74.2, CH	3.84 ovl
6‴	62.9, CH_2_	3.82 ovl;			62.7, CH_2_	3.68 ovl;
		3.71 ovl				3.82 ovl

Compounds **4** and **6** contain a planteose sugar, while compound **5** features a plantebiose sugar. This is the first report of these sugars being identified in the iridoid class.

Compound **7** was obtained as a white amorphous powder with the molecular formula C_50_H_70_O_25_ by HRESIMS analysis (*m*/*z* 1051.40649 [M – H_2_O – H]^−^). A careful comparison of the ^1^^3^C NMR data of **7** with those of **3** revealed two sets of NMR signals corresponding to the structure of **3** lacking methoxy substituents at C-7a1 and C-7a2. This suggests that **7** is a dimeric form of **3** without a methoxy substitution at the C-7 positions. The HMBC correlation between H-7a1 and H-7a2 indicated that C-7a1 and C-7a2 are connected via an ether linkage (Figure 1). Furthermore, the key ROESY correlations observed for compound **7** were the same as those of compound **3** (Figure 2). From the above analysis, combined with the assumption of β-orientations for C-1a1 and C-1a2, the absolute structure of compound **7** was established and named gomphandranoside G.

Compound **8** was isolated as a white, amorphous solid. Its molecular formula C_58_H_78_O_30_ was established by analysis of HRESIMS data (*m*/*z* 1299.45386 [M + HCOO]^−^). Its structure was established by analysis of 1D and 2D NMR data and comparison with NMR data of **3** and of cantleyoside.^22^ The complete correspondence of the ^1^^3^C NMR data of **8** with those of the boonein unit in **3** and **7** indicated that **8** contains a boonein unit in its structure. These data demonstrate that **8** includes one glycosidic iridoid trimer, two secoiridoid-type units, and one iridoid-type unit as well as one boonein unit. The absolute structure of the boonein unit was identified by the similar ROESY correlations and the close agreement of the ^13^C NMR data to those of compound **3**. For the iridoid-type units, a comparison with the NMR data of loganin and cantleyoside,^22^ along with the 2D NMR signals, suggested that the loganin segment is esterified at C-7 (Figures 1, 2). The HMBC correlations between H-7a1/C-6a2, C-7a2, C-5a2m and H-6a1/C-6a2 established the linkage of two secoiridoid-type units (Figure 1). The *Z*-configuration of this double bond was also determined by the ROESY correlation H-7a1/H-7a2 (aldehyde group) (Figure 2). Besides, the relative configuration was identified based on the key ROESY correlation analysis (Figure 2). From the above analysis and the assumption of a β-configuration at C-1a1, C-1a2, and C-1b2, the absolute structure of **8** was proposed and named gomphandranoside H.

Compound **9** was isolated as a white amorphous powder with the molecular formula C_66_H_90_O_37_ established by HRESIMS analysis (*m*/*z* 1519.51025 [M + HCOO]^−^). Analysis of the 1D and 2D spectra indicated four sets of olefinic proton signals at δ_H_ 7.56 (s, 1H), 7.42 (d, 1.3 Hz, 1H), 7.27 (d, 1.8 Hz, 1H), and 7.42 (d, 1.3 Hz, 1H), and β-glucopyranoside anomeric signals at δ_C_ 100.1/δ_H_ 4.73 (d, 7.9 Hz, 1H), δ_C_ 100.1/δ_H_ 4.67 (d, 7.9 Hz, 1H), δ_C_ 99.7/δ_H_ 4.67 (d, 7.9 Hz, 1H), and δ_C_ 100.1/δ_H_ 4.67 (d, 7.9 Hz, 1H), which indicated that **9** is an iridoid glycosidic tetramer. In addition, two signals at δ_C_ 14.1/δ_H_ 1.09 (d, 6.7 Hz, 3H) × 2 and good agreement of the ^13^C NMR data with the loganin unit of cantleyoside^17^ indicated that **9** contains two loganin units, connected to the remaining part through ester linkages. Their linkages were confirmed by HMBC correlations between H-7b1 and C-11a1 and H-7b2 and C-11a2. The other units were determined by observation of key COSY and HMBC correlations, and their connections were established according to HMBC cross peaks of H-6a2, H-7a2/C-6a1; H-5a1, H-9a1/C-6a1; and H-7a1/C-6a1, C-7a2, C-8a2 (Figure 1). The ROESY spectrum and analysis of the coupling constants enabled us to propose the relative configuration of **9**. ROESY correlations of H-5a1/H-9a1 and H-1a1/H-8a1, along with the large coupling constant of H-1a1 (8.5 Hz), indicated a *cis* configuration for H-5a1–H-9a1 and a *trans* configuration for H-1a1–H-9a1. In addition, the ROESY cross peaks between H-10a2 and H-5a2 and H-10a2 and H-9a2 suggested their *cis* orientation (Figure 2). The *cis* orientation of H-1a2, H-8a2, and H-7a2 was also established by the ROESY correlations from H-1a2 to H-8a2 and H-7a2 (Figure 2). From the above analysis and the assumption of a β-configuration at C-1a1, C-1a2, C-1b1, and C-1b2, the absolute structure of **9** was determined as gomphandranoside I.

Compound **10** was obtained as a white amorphous powder. Its molecular formula, C_66_H_90_O_37_, was established by analysis of HRESIMS data (*m*/*z* 1519.51270 [M + HCOO]^−^), indicating it as an isomer of compound **9**. Therefore, compound **10** is also a glycosidic iridoid tetramer with the same two loganin units but differs in the other components compared to **9**. The ester linkages between the two loganin units to the other ones were determined based on HMBC correlations of H-7b1/C-11a1 and H-7b2/C-11a2. The connections of the other units were established by the analysis of HMBC correlations of H-7a1/C-6a1, C-10a2, C-7a2 and a ROESY correlation of H-5a1/H-7a1 (Figures 1 and 2). The large coupling constant of H_1a1,9a1_ = 8.5 Hz and the ROESY cross peaks of H-5a1/H-9a1 suggested a *cis* orientation of H-5a1–H-9a1 and a *trans* orientation of H-1a1–H-9a1. Furthermore, the ROESY correlations of H-5a2/H-9a2 and H-1a2/H-8a2/H-7a2 and no ROESY signals observed between H-8a2/H-9a2 and H-5a2/H-7a2 indicated *cis* configurations of H-5a2–H-9a2 and H-7a2–H-8a2 and *trans* configurations of H-1a2–H-9a2 and H-9a2–H-8a2 (Figure 2). From the above analysis and the assumption of a β-configuration at C-1a1, C-1a2, C-1b1, and C-1b2, the absolute structure of **10** was established and named gomphandranoside K.

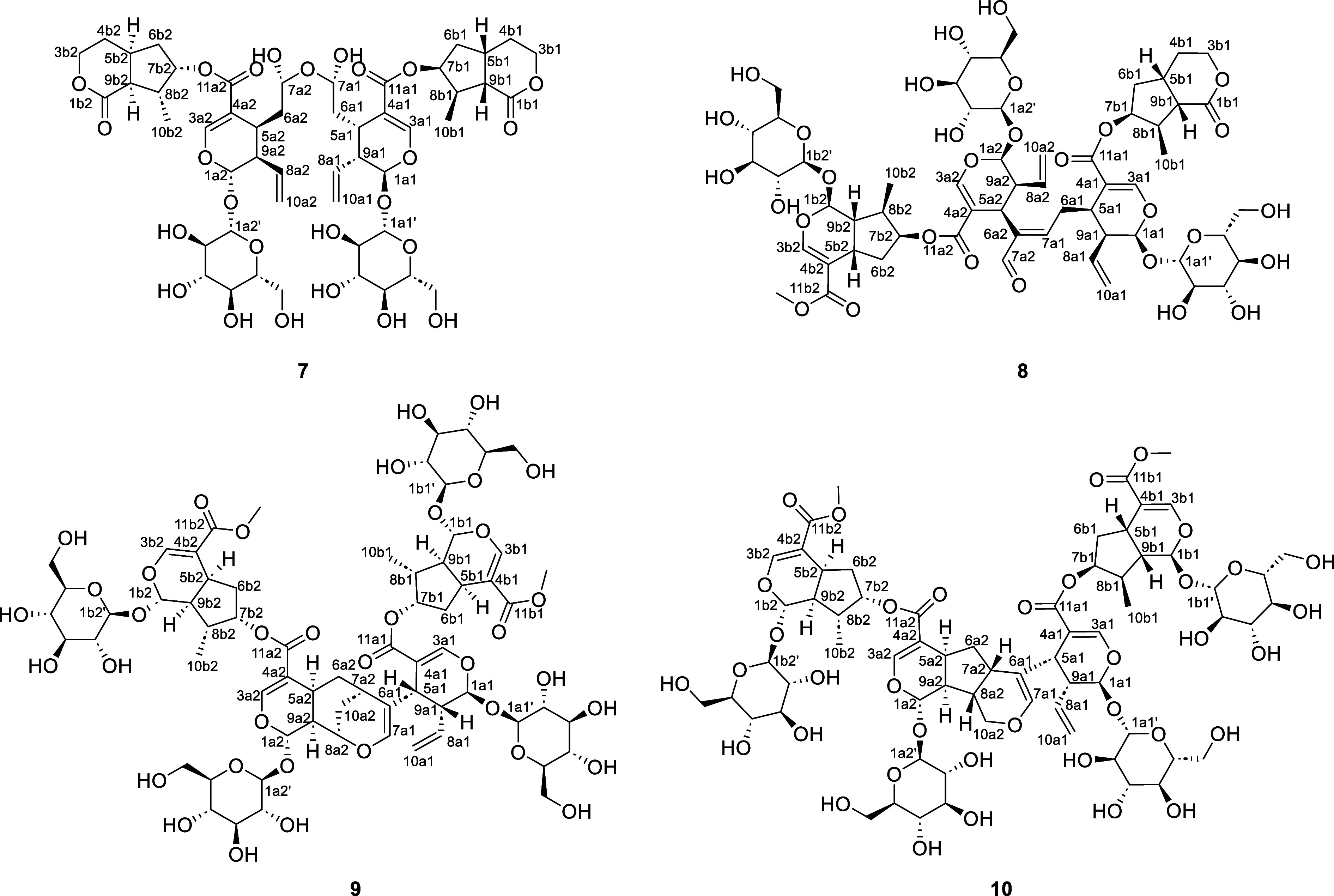



## Bioactivity Assessment

### Anti-inflammatory Activity and Structure–Activity Relationship (SAR)

For the assessment of the anti-inflammatory activity, compounds **1**, **3**–**13**, and **15**–**18** demonstrated NO inhibition with IC_50_ values ranging from 6.13 to 51.1 μM. Among these, compound **15** exhibited the highest inhibitory potency, achieving an IC_50_ value of 6.13 μM, which surpassed the potency of the positive control (dexamethasone). This was followed by compound **1**, with an IC_50_ value of 9.18 μM (Table 5). These results highlight the significant anti-inflammatory bioactivity of iridoids from *G. mollis*, which is consistent with findings from previous studies on the iridoid class.^42,43^ Compound **18** (logain) is a well-known anti-inflammatory agent with diverse mechanisms of action, including the inhibition of IL-6, TNF-α, and IL-1β expression, suppression of TLR4/NF-κB and JAK/STAT3 signaling pathways, and upregulation of the Nrf2/HO-1 signaling pathway.^44,45^ Similarly, compound **13** (sweroside) demonstrated anti-inflammatory effects by alleviating NF-κB signaling, enhancing SIRT1 expression, and reducing NF-κB pathway activity.^46^ Compound **12** (canthleyoside) can also reduce pro-inflammatory cytokines such as TNF-α and IL-1β/6.^47^ This study represents the first assessment of the remaining compounds’ anti-inflammatory activity. Interestingly, despite extensive research demonstrating the anti-inflammatory effects and mechanisms of compounds **18** and **13**, they exhibited the weakest effects among the tested compounds. This result suggests that the remaining compounds may possess significant potential while also highlighting the promising potential of *G. mollis* iridoids and the plant extract in the treatment of inflammation-related diseases.

**Table 4:** ^1^H (600 MHz) and ^13^C (150 MHz) NMR Data of Compounds **7**–**10** in CD_3_OD

	Compound **7**		Compound **8**		Compound **9**		Compound **10**	
No.	*δ*_C_, type	*δ*_H_ (*J* in Hz)	*δ*_C_, type	*δ*_H_ (*J* in Hz)	*δ*_C_, type	*δ*_H_ (*J* in Hz)	*δ*_C_, type	*δ*_H_ (*J* in Hz)
1a1	97.9, CH	5.52 d (5.5)	97.8, CH	5.57 d (5.0)	98.0, CH	5.46 d (8.5)	97.3, CH	5.52 d (8.5)
3a1	153.7, CH	7.45 d (1.3)	154.2, CH	7.5s brs	153.9, CH	7.56 s	153.5, CH	7.55 s
4a1	112.1, C		111.0, C		110.6, C		111.1, C	
5a1	29.5, CH	2.98 m	33.2, CH	3.00 ovl	39.4, CH	3.23 d (5.1)	74.7, CH	3.32 ovl
6a1	37.3, CH_2_	1.95 dt (13.5, 6.1);	29.6, CH_2_	3.20 ovl	114.1, C		113.3, C	
		1.72 ddd (13.5, 8.1, 5.0)		2.38 m				
7a1	98.3, CH	4.62 ovl	156.1, CH	6.77 t (7.1)	144.2, CH	6.09 s	144.0, CH	6.07 s
8a1	135.7, CH	5.74 m	135.3, CH	5.77 m	137.5, CH	5.66 ddd (17.3, 10.5, 8.5)	136.6, CH	5.67 m
9a1	45.3, CH	2.68 m	45.3, CH	2.80 ovl	47.7, CH	2.57 td (8.5, 5.1)	47.3, CH	2.63 td (8.5, 6.1)
10a1	119.8, CH_2_	5.26 m	120.6, CH_2_	5.31 brd (10.7)	118.5, CH_2_	5.19 ovl	118.9, CH_2_	5.16 m
				5.38 brd (17.1, 1.9)				
11a1	168.3, C		168.1, C		167.5, C		168.3, C	
1a1′	100.0, CH	4.68 d (7.9)	100.1, CH	4.68 d (7.8)	100.1, CH	4.73 d (7.9)	99.9, CH	4.74 d (7.9)
2a1′	74.6, CH	3.20 ovl	74.4, CH	3.28 ovl	74.7, CH	3.20 ovl	74.7, CH	3.21 ovl
3a1′	78.0, CH	3.36 t (8.8)	77.8, CH	3.37 ovl	78.0, CH	3.37 ovl	77.8, CH	3.43 t (9.0)
4a1′	71.5, CH	3.29 ovl	71.6, CH	3.30 ovl	71.5, CH	3.30 ovl	71.6, CH	3.26 ovl
5a1′	78.4, CH	3.31 ovl	78.3, CH	3.31 ovl	78.4, CH	3.31 ovl	78.3, CH	3.31 ovl
6a1′	62.7, CH_2_	3.90 dd (12.0, 2.1);	62.7, CH_2_	3.90 ovl;	62.8, CH_2_	3.90 ovl;	62.7, CH_2_	3.66 ovl;
		3.67 ovl		3.67 ovl		3.67 ovl		3.90 ovl
1b1	177.4, C		177.3, C		97.5, CH	5.30 d (4.7)	97.6, CH	5.27 d (4.3)
3b1	68.6, CH_2_	4.28 ddd (11.2, 8.9, 2.5);	68.4, CH_2_	4.27 m	152.5, CH	7.42 d (1.3)	152.4, CH	7.41 d (1.3)
		4.37 ddd (11.2, 6.1, 3.0)		4.36 m				
4b1	30.4, CH_2_	1.52 dtd (14.5, 8.9, 3.0);	30.3, CH_2_	1.51 dtd (14.3, 8.9, 3.0)	113.3, C		113.3, C	
		2.11 ovl		2.11 ovl				
5b1	35.4, CH	2.86 m	35.4, CH	2.84 ovl	32.5, CH	3.08 ovl	32.7, CH	3.14 ovl
6b1	39.7, CH_2_	2.14 ovl;	39.7, CH_2_	1.70 ddd (14.3, 10.0, 4.0)	40.1, CH_2_	1.77 ovl;	40.6, CH_2_	2.30 ovl
		1.67 ovl		2.16 ovl		2.25 ovl		1.68 ddd (14.4, 8.1, 4.8)
7b1	79.3, CH	5.22 dt (14.9, 4.2)	79.6, CH	5.26 brt (4.0)	78.1, CH	5.22 ovl	78.3, CH	5.18 ovl
8b1	43.9, CH	2.41 m	43.9, CH	2.45 m	40.7, CH	2.13 ovl	41.1, CH	2.10 ovl
9b1	49.0, CH	2.79 m	49.2, CH	2.78 ovl	47.4, CH	2.10 ovl	46.9, CH	2.10 ovl
10b1	14.7, CH_3_	1.15 d (6.8)	14.8, CH_3_	1.17 d (6.8)	14.1, CH_3_	1.09 d (6.7)	13.9, CH_3_	1.01 d (6.0)
11b1					169.3, C		169.2, C	
OMe					51.8, CH_3_	3.70 s	51.8, CH_3_	3.69 s
1b1′					100.1, CH	4.67 d (7.9)	100.2, CH	4.65 d (7.9)
2b1′					74.7, CH	3.20 ovl	74.7, CH	3.21 ovl
3b1′					78.0, CH	3.37 ovl	78.0, CH	3.37 ovl
4b1′					71.5, CH	3.30 ovl	71.5, CH	3.31 ovl
5b1′					78.2, CH	3.31 ovl	78.4, CH	3.31 ovl
6b1′					62.8, CH_2_	3.90 ovl;	62.7, CH_2_	3.66 ovl;
						3.67 ovl		3.90 ovl
1a2	97.8, CH	5.52 d (5.5)	97.1, CH	5.49 d (2.9)	97.2, CH	5.55 d (3.5)	96.1, CH	5.46 d (2.2)
3a2	153.4, CH	7.46 d (1.3)	151.9, CH	7.48 d (2.2)	153.2, CH	7.27 d (1.8)	151.0, CH	7.36 s
4a2	112.1, C		109.8, C		112.3, C		116.0, C	
5a2	29.8, CH	2.98 m	30.7, CH	4.10 brd (5.4)	24.2, CH	2.88 m	32.1, CH	3.07 dt (10.3, 7.2)
6a2	37.8, CH_2_	1.68 ovl;	143.7, C		32.7, CH_2_	1.94 m;	38.5, CH	2.51 dd (7.2, 5.3)
		2.03 ddd (13.2, 7.2, 5.5)				2.06 ovl		
7a2	98.1, CH	4.66 ovl	197.1, CH	9.32 s	28.3, CH	2.22 ovl	42.7, CH	2.31 ovl
8a2	135.9, CH	5.74 m	135.6, CH	5.64 dt (17.0, 10.0)	71.0, CH	4.59 m	45.1, CH	1.79 ovl
9a2	45.4, CH	2.68 m	46.6, CH	2.58 m	43.8, CH	2.23 ovl	43.2, CH	2.14 ovl
10a2	119.8, CH_2_	5.30 m	119.4, CH2	5.02 brd (10.0);	29.1, CH_2_	1.82 ovl;	70.4, CH_2_	4.35 dd (10.1, 3.7);
				5.06 brd (17.0)		1.74 ovl		3.85 ovl
11a2	168.3, C		168.3, C		168.3, C		168.6, C	
1a2′	100.0, CH	4.68 d (7.9)	99.5, CH	4.68 d (7.8)	99.7, CH	4.67 d (7.9)	99.9, CH	4.61 d (8.0)
2a2′	74.6, CH	3.20 ovl	74.8, CH	3.28 ovl	74.6, CH	3.20 ovl	74.5, CH	3.16 ovl
3a2′	78.0, CH	3.36 t (8.8)	78.2, CH	3.37 ovl	78.0, CH	3.37 ovl	78.0, CH	3.37 ovl
4a2′	71.5, CH	3.29 ovl	71.6, CH	3.30 ovl	71.5, CH	3.30 ovl	71.6, CH	3.31 ovl
5a2′	78.4, CH	3.31 ovl	78.4, CH	3.31 ovl	78.2, CH	3.31 ovl	78.4, CH	3.31 ovl
6a2′	62.7, CH_2_	3.90 dd (12.0, 2.1);	62.9, CH_2_	3.90 ovl;	62.8, CH_2_	3.90 ovl;	62.9, CH_2_	3.66 ovl;
		3.67 ovl		3.67 ovl		3.67 ovl		3.90 ovl
1b2	177.4, C		98.3, CH	5.19 d (5.9)	97.4, CH	5.33 d (4.2)	98.3, CH	5.26 d (5.2)
3b2	68.6, CH_2_	4.28 ddd (11.2, 8.9, 2.5);	153.2, CH	7.45 (s)	152.4, CH	7.42 d (1.3)	152.7, CH	7.46 d (1.3)
		4.37 ddd (11.2, 6.1, 3.0)						
4b2	30.4, CH_2_	1.52 dtd (14.5, 8.5, 3.1);	112.6, C		113.3, C		113.3, C	
		2.11 ovl						
5b2	35.3, CH	2.86 m	33.5, CH	3.02 ovl	32.3, CH	3.08 ovl	33.1, CH	3.14 ovl
6b2	39.7, CH_2_	2.14 ovl;	40.3, CH_2_	1.61 ddd (13.9, 9.1, 4.8);	40.1, CH_2_	1.77 ovl;	40.8, CH_2_	1.76 ovl;
		1.67 ovl		2.16 ovl		2.25 ovl		2.39 dd (14.1, 8.1)
7b2	79.3, CH	5.22 dt (14.9, 4.2)	78.5, CH	5.16 t (5.1)	78.0, CH	5.22 ovl	78.4, CH	5.24 td (4.9, 1.4)
8b2	43.9, CH	2.41 m	41.1, CH	2.14 ovl	40.8, CH	2.13 ovl	41.3, CH	2.17 ovl
9b2	49.0, CH	2.79 m	46.9, CH	1.98 q (7.0)	47.4, CH	2.10 ovl	47.3, CH	2.10 ovl
10b2	14.6, CH_3_	1.16 d (6.8)	14.3, CH_3_	1.01 d (7.0)	14.1, CH_3_	1.09 d (6.7)	14.3, CH_3_	1.10 d (6.8)
11b2			169.5, C		169.3, C		169.2, C	
OMe			51.8, CH_3_	3.72 s	51.9, CH_3_	3.70 s	51.8, CH_3_	3.69 s
1b2′			100.4, CH	4.66 d (7.9)	100.1, CH	4.67 d (7.9)	100.6, CH	4.68 d (7.8)
2b2′			74.7, CH	3.21 dd (9.3, 7.9)	74.7, CH	3.20 ovl	74.5, CH	3.16 ovl
3b2′			78.0, CH	3.37 dd (9.3 8.6)	78.0, CH	3.37 ovl	78.1, CH	3.37 ovl
4b2′			71.5, CH	3.30 ovl	71.5, CH	3.30 ovl	71.7, CH	3.31 ovl
5b2′			78.4, CH	3.30 ovl	78.4, CH	3.31 ovl	78.5, CH	3.31 ovl
6b2′			62.8, CH_2_	3.90 ovl;	62.8, CH_2_	3.90 ovl;	62.9, CH_2_	3.66 ovl;
				3.67 ovl		3.67 ovl		3.90 ovl

**Table 5:** NO Production Inhibitory Effects of Compounds **1**, **3**–**13**, **15**–**18**, and Dexamethasone

Compd	IC_50_ value (μM)	Cell viability (at 80 μM) (%)	Compd	IC_50_ value (μM)	Cell viability (at 80 μM) (%)
**1**	9.18 ± 0.3	97.7 ± 2.3	**11**	26.2 ± 1.3	94.8 ± 3.7
**3**	23.8 ± 2.6	103.4 ± 3.5	**12**	23.1 ± 1.7	93.9 ± 2.5
**4**	19.5 ± 0.3	95.1 ± 2.8	**13**	36.0 ± 1.6	94.7 ± 4.2
**5**	17.7 ± 0.4	98.9 ± 3.1	**15**	6.13 ± 0.5	99.5 ± 3.6
**6**	20.2 ± 1.1	94.6 ± 4.5	**16**	17.0 ± 1.1	95.2 ± 2.1
**7**	14.7 ± 0.9	95.3 ± 2.4	**17**	14.4 ± 1.5	94.2 ± 4.6
**8**	14.4 ± 1.2	100.2 ± 2.5	**18**	51.1 ± 1.8	98.1 ± 4.0
**9**	12.9 ± 0.5	98.3 ± 2.6	positive control (dexamethasone)	9.08 ± 0.5	96.3 ± 2.6
**10**	12.2 ± 1.1	96.7 ± 1.5			(at 20 μM)

To investigate the SAR, it was observed that iridoid glycosides generally exhibited a strong correlation between NO inhibition effects and structural complexity, with the notable exception of compound **1**. Monomeric compounds **18** and **13** displayed the weakest activities, with IC_50_ values of 51.1 and 36.0 μM, respectively. In contrast, dimeric compounds (**3**–**6**, **11**, **12**, and **16**) demonstrated enhanced activity, with IC_50_ values ranging from 17.0 to 26.2 μM. Remarkably, tetrameric compounds (**7**–**10**, **15**, and **17**) exhibited the highest potency, with IC_50_ values between 6.13 and 14.7 μM. Compound **1**, despite being a monomeric iridoid, displayed an IC_50_ value of 9.19 μM, which significantly surpassed compound **18** (IC_50_ = 51.1 μM). This activity, superior to those of most dimeric and even some tetrameric forms, highlights the critical role of the conjugated α-d-galactosyl moiety at the C-6′ position of loganin in enhancing biological efficacy.

Further analysis of monomeric compounds revealed that compound **18**, a seco-iridoid containing an intramolecular lactone, exhibited better activity than compound **13**. This finding suggests that the seco-iridoid framework, specifically the presence of a lactone ring, plays a pivotal role in enhancing NO inhibitory effects.

**Figure 3:**
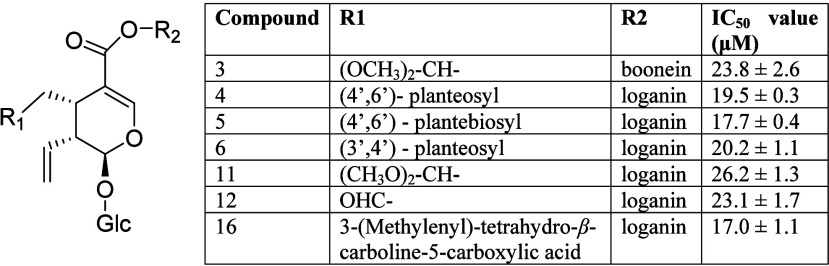
**S**tructures and IC_50_ values for NO inhibitory effects of dimeric iridoid compounds.

For the analysis of dimeric compounds **3**–**6**, **11**, **12**, and **16**, their structures and IC_50_ values are summarized in Figure 3. The anti-inflammatory differences among these compounds are attributed to variations in the R_1_ and R_2_ positions. Using compound **11** as the reference, replacing the loganin unit with the boonein unit (3) at the R_2_ position or substituting the (CH_3_O)_2_CH– group in **11** with a −CHO group (compound **12**) causes no significant change in bioactivity (*p* > 0.05). However, introducing more complex groups, such as sugar chains (**4**–**6**) or the tetrahydro-β-carboline-5-carboxylic acid group (**16**) at the R_1_ position, resulted in better IC_50_ values ranging from 17.0 to 19.5 μM, which were superior to those of simpler groups (compounds **3**, **11**, and **12**). Among these, the tetrahydro-β-carboline-5-carboxylic acid group (**16**) exhibited the highest activity, followed by planteose (compounds **4** and **6**) and plantebiose (**5**). For compounds with the same planteose sugar group, the (4′,6′)-*O*-glycoside linkage (**4**) showed no significant difference (*p* > 0.05) compared to the (4′,3′)-*O*-glycoside linkage (**6**).

For the analysis of tetrameric compounds **7**–**10**, **15**, and **17**, the results indicated that an increase in the number of glucose units corresponded to enhanced NO inhibitory activity. Compound **7**, with two glucose units, exhibited an IC_50_ value of 14.7 μM, while compound **8**, containing three glucose units, displayed an IC_50_ value of 14.4 μM. Compounds **9**, **10**, **15**, and **17**, which have four glucose units, showed IC_50_ values ranging from 6.13 to 14.4 μM. Interestingly, compounds **9**, **10**, **15**, and **17** are dimers of canthleyoside, linked either through a double-bond bridge (compound **15** with an *E* configuration and compound **17** with a *Z* configuration) or via a cyclic system (compound **9** with a [6–6–6] ring system and compound **10** with a [6–5–6] ring system). The results revealed the following trend in IC_50_ values: compound **15** (6.13 μM) < compound **10** (12.2 μM) < compound **9** (12.9 μM) < compound **17** (14.4 μM). This suggests that the mode of conjugation between the two canthleyoside units significantly affects their NO inhibitory activity.

### Hepatoprotective Activity

**Figure 4:**
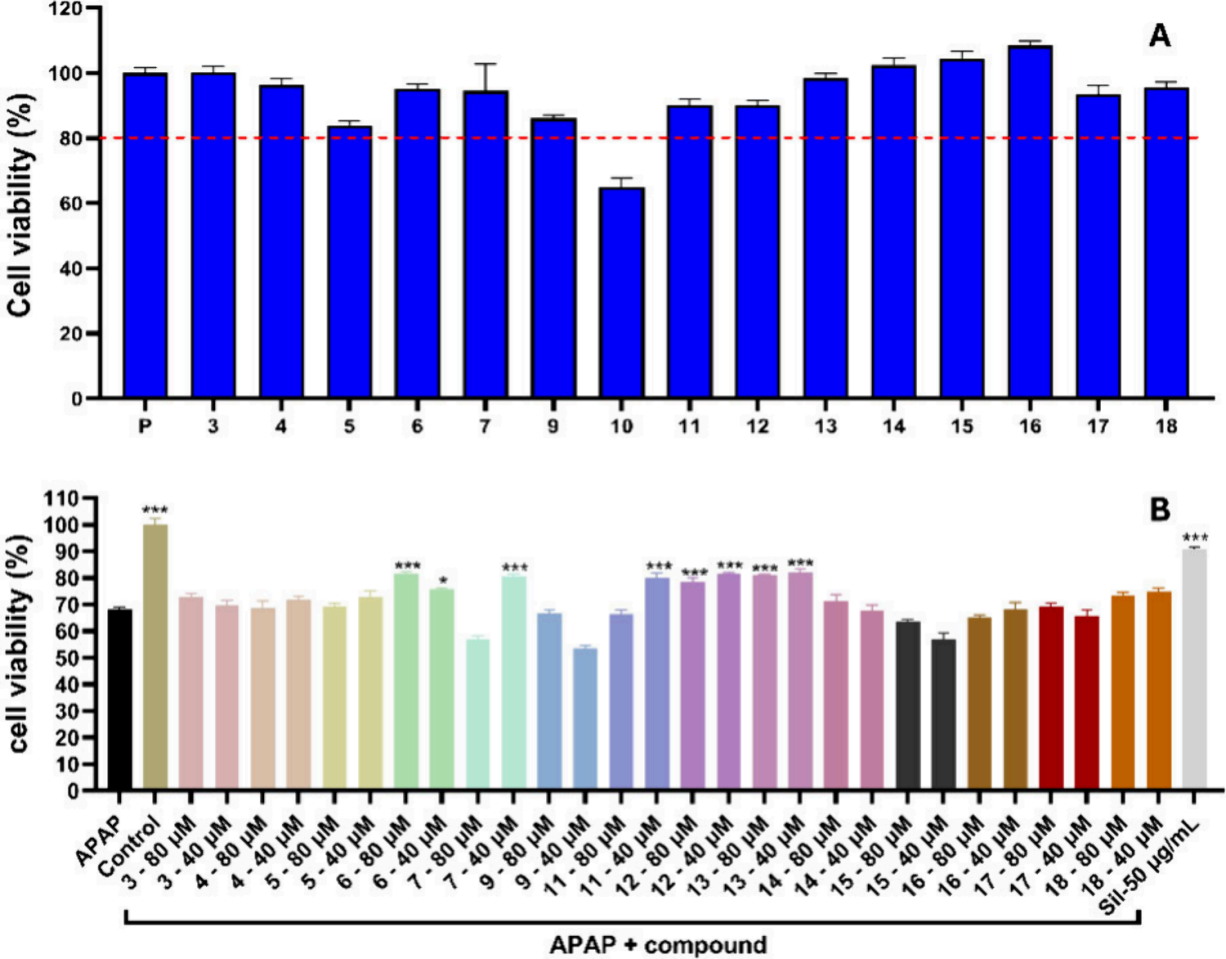
HepG2 cytotoxicity test at 80 μM (A) and hepatoprotective effects prevent APAP toxicity of compounds **3**–**7**, **9**, and **11**–**18** (80 and 40 μM, pretreated for 8 h) by 6 mM APAP for 36 h (*P < 0.05 vs APAP; ***P < 0.001 vs APAP) (B).

For the hepatoprotective activity, compounds **3**–**7**, **9**, and **11**–**18**, which yield cell viabilities above 80% at concentration of 80 μM, were further screened for liver protective effects in acetaminophen-induced hepatoxicity in HepG2 models. The sample treated only with acetaminophen (APAP) showed a cell viability of 68.1%. In contrast, the control samples not exposed to APAP demonstrated a cell viability of 100%. The positive control sample, treated with both APAP and silymarin, exhibited the strongest activity among the tested samples, with a cell viability of 90.9%, confirming the successful establishment of the model. Compounds **6**, **12**, and **13**, at both 80 and 40 μM, as well as compounds **7** and **11** at 40 μM, exhibited significant hepatoprotective effects, with cell viability ranging from 75.6% to 81.9% (Figure 4). These findings align with the well-documented hepatoprotective properties commonly associated with iridoid compounds. Iridoids are known for their ability to modulate oxidative stress, reduce inflammation, and protect hepatocytes from damage caused by toxins or other stressors.^48−51^

In conclusion, our study not only enriches the structural database of iridoids, particularly of complex iridoids, but also underscores their promising therapeutic potential for liver protection and inflammation-related diseases. This study also provides additional chemical and biological evidence for the iridoid compounds of *G. mollis*, paving the way for further research and enhancing the value and potential applications of this medicinal plant.

## Experimental Section

### General Experimental Procedures

UV–vis absorption spectra were measured using a Thermo UV–vis Helios spectrophotometer (USA), while infrared (IR) spectra were recorded on a JASCO FT/IR 4100 spectrometer (Japan). Optical rotations were determined with a JASCO P-2000 polarimeter (Japan), and NMR spectra were obtained by using an Agilent DD2 600 MHz spectrometer (USA). HPLC analysis was performed on a Hitachi L-7000 system, and preparative HPLC was carried out on a Shimadzu HPLC-20AR system (Japan). Medium-pressure liquid chromatography (MPLC) was conducted by using the Isolera ONE system (Sweden). Polysaccharide composition was analyzed by using a kit from Sugar Light Co. (Taiwan). ELISA measurements were conducted on a NanoQuant Infinite M200 Pro reader (Tecan, Switzerland). HepG2 cells were obtained from the Bioresource Collection and Research Center (BCRC) in Taiwan for bioassays. Raw264.7 cells were obtained from the Biotechnology Center of Ho Chi Minh City, Vietnam.

### Material

The root of *Gomphandra mollis* Merr. was collected in the northwest area of Vietnam, in July 2021. The scientific name was confirmed by Dr. Vo Thanh Hoa, University of Health Science, Vietnam National University, Ho Chi Minh City. A voucher specimen (GM_062021) is stored at the Pharmacognosy lab, College of Pharmacy, Taipei Medical University.

### Extraction and Isolation

A total of 2.0 kg of dry material (*Gomphandra mollis* root) was extracted under reflux with 40 L of 96% EtOH during 5 h, followed by evaporation of the solvent to yield 132 g of total extract. This extract was subjected to chromatography on a Diaion HP-20 column and washed by H_2_O and eluted with MeOH and acetone. The MeOH fraction (49.4 g) was applied to a normal phase (silica gel) chromatography column with a gradient condition using EtOAc–MeOH–H_2_O (100:0:0, 90:10:0, 80:20:0, 80:20:1, 80:20:3, 75:25:5) as mobile phase. Eleven fractions (Fr.1 to Fr.11) were obtained. Fr.11 (2.1 g) was purified by MPLC with a C-18 column using a MeOH–H_2_O (0:100, 15:85, 20:80, 25:75) step gradient. The fraction eluted with 25% MeOH was concentrated to obtain compound **4** (750 mg). Fr.3 (2.9 g), Fr.4 (6.6 g), and Fr.5 (4.3 g) were also separated with an MPLC C-18 column with the same separation conditions (MeOH–H_2_O, 0:100–50:50) to yield subfractions Fr.3.1–Fr.3.13, Fr.4.1–Fr.4.9, and Fr.5.1–Fr.5.7. After TLC and HPLC analyses, subfractions Fr.3.10, Fr.4.8, and Fr.5.6 yielded compound **11** (3259 mg). Subfractions Fr.3.9, Fr.4.6, and Fr.5.3 were also combined and evaporated to give compound **12** (2815 mg). Compound **14** (7.5 mg) was isolated from subfraction Fr.3.5 (421 mg) by preparative HPLC (C-18 column, MeCN 9%, 8 mL/min). Fr.7 (2.1 g) was separated by normal phase (silica gel) column chromatography with CH_2_Cl_2_–MeOH (9:1–5:5) as the mobile phase to yield compound **1** (7.5 mg) and compound **8** (3 mg). Fr.10 (2.0 g) was purified by MPLC (C-18 column, MeOH–H_2_O (0:100–40:60)) to give 10 subfractions, Fr.10.1–Fr.10.10. Subfraction Fr.10.7 (87 mg) was purified by preparative HPLC (C-18 column, MeCN 20%, 8 mL/min) to yield compound **9** (9.7 mg, 25 min) and compound **16** (26.5 mg, 43 min). Compound **10** (9.1 mg, 57 min) was also isolated from Fr.10.8 (169 mg) with the same preparative HPLC condition of Fr.10.7. Fr.10.3 (215 mg) was separated by preparative HPLC (C-18 column, MeCN 15%, 8 mL/min) to give compound **5** (37.7 mg, 37 min) and compound **6** (25 mg, 48 min). Fr.8.1–Fr.8.7 were separated from Fr.8 (3.0 g) by the MPLC method (C-18 column, MeOH–H_2_O (10:90–40:60). Separation of subfraction Fr.8.6 (1.1 g) by repeated preparative HPLC (C-18 column, MeCN 20%, 8 mL/min) provided compound **17** (34.9 mg, 26 min) and compound **15** (384 mg, 37 min). From Fr.2 (3.6 g), MPLC with a C-18 column as stationary phase and MeOH–H_2_O (5:95–30:70) as mobile phase yielded 10 subfractions, Fr.2.1–Fr.2.10. Compound **13** (500 mg) and compound **18** (154.8 mg) were isolated from Fr2.2 (604 mg) and Fr.2.3 (277 mg) under the same conditions of preparative HPLC (C-18 column, MeOH 25%, 7 mL/min). Using the same preparative HPLC condition (C-18 column, MeCN 16%, 8 mL/min), Fr.2.7 (263 mg) and Fr.2.8 (231 mg) were obtained. Fr.2.9 (333 mg) was applied to preparative HPLC (C-18 column, MeCN 22%, 8 mL/min) to give compound **7** (15.3 mg, 31 min), compound **2** (7.5 mg, 41 min), and compound **3** (54.2 mg, 70 min). The detailed spectroscopic data of compounds **1**–**10** are shown in Supporting Information S2.

#### Gomphandranoside A (**1**)

White amorphous powder; [α]^20^_D_ −3.6 (*c* 0.10, MeOH); UV (MeOH) λ_*max*_ 235 nm; IR (ATR) υ_*max*_ 1056, 1292, 1666, 3370 cm^–1^; NMR (600 MHz, MeOD-*d*_4_), see Table 1. C_23_H_36_O_15_; HRESIMS *m*/*z* 553.21198 ([M + H]^+^ calcd for C_23_H_37_O_15_).

#### Gomphandranoside B (**2**)

White amorphous powder; [α]^20^_D_ −46.2 (*c* 0.10, MeOH); UV (MeOH) λ_*max*_ 206, 228 nm; IR (ATR) υ_*max*_ 1052, 1276, 1700, 3382, 3860 cm^–1^; NMR (600 MHz, MeOD-*d*_4_), see Table 1; HRESIMS *m*/*z* 554.22278 [M + H]^+^ (calcd for C_23_H_37_O_15_).

#### Gomphandranoside C (**3**)

White amorphous powder; [α]^20^_D_ −74.4 (*c* 0.10, MeOH); UV (MeOH) λ_*max*_ 205, 232 nm; IR (ATR) υ_*max*_ 1066, 1265, 1708, 3405, 3856 cm^–1^; NMR (600 MHz, MeOD-*d*_4_), see Table 2; HRESIMS *m*/*z* 617.24408 [M + HCOO]^−^ (calcd for C_28_H_41_O_15_).

#### Gomphandranoside D (**4**)

White amorphous powder; [α]^20^_D_ −26.2 (*c* 0.10, MeOH); UV (MeOH) λ_*max*_ 226 nm; IR (ATR) υ_*max*_ 1041, 1284, 1662, 3370, 3860 cm^–1^; NMR (600 MHz, MeOD-*d*_4_), see Table 3; HRESIMS *m*/*z* 1233.42908 [M + H]^+^ (calcd for C_51_H_77_O_34_).

#### Gomphandranoside E (**5**)

White amorphous powder; [α]^20^_D_ −50.2 (*c* 0.10, MeOH); UV (MeOH) λ_*max*_ 229 nm; IR (ATR) υ_*max*_ 1033, 1292, 1670, 2055, 3386, 3687, 3864 cm^–1^. NMR (600 MHz, MeOD-*d*_4_), see Table 3; HRESIMS *m*/*z* 1093.35693 [M + Na]^+^ (calcd for C_45_H_66_O_29_Na).

#### Gomphandranoside F (**6**)

White amorphous powder; [α]^20^_D_ −32.0 (*c* 0.10, MeOH); UV (MeOH) λ_*max*_ 233 nm; IR (ATR) υ_*max*_ 1033, 1292, 1670, 2055, 3386, 3687, 3864 cm^–1^; NMR (600 MHz, MeOD-*d*_4_), see Table 3; HRESIMS *m*/*z* 1231.41235 [M – H]^−^ (calcd for C_51_H_75_O_34_).

#### Gomphandranoside G (**7**)

White amorphous powder; [α]^20^_D_ −86.6 (*c* 0.10, MeOH); UV (MeOH) λ_*max*_ 238 nm; IR (ATR) υ_*max*_ 1076, 1276, 1527, 1704, 3424, 3856 cm^–1^; NMR (600 MHz, MeOD-*d*_4_), see Table 4; HRESIMS *m*/*z* 1097.40759 [M – H_2_O + HCOO]^−^ (calcd for C_51_H_69_O_26_).

#### Gomphandranoside H (**8**)

White amorphous powder; [α]^20^_D_ −54.6 (*c* 0.10, MeOH); UV (MeOH) λ_*max*_ 233 nm; IR (ATR) υ_*max*_ 1083, 1280, 1677, 3397, 3856 cm^–1^; NMR (600 MHz, MeOD-*d*_4_), see Table 4; HRESIMS *m*/*z* 1255.46411 [M + H]^+^ (calcd for C_58_H_79_O_30_).

#### Gomphandranoside I (**9**)

White amorphous powder; [α]^20^_D_ −126.6 (*c* 0.10, MeOH); UV (MeOH) λ_*max*_ 226 nm; IR (ATR) υ_*max*_ 1091, 1284, 1527, 1673, 3413, 3853 cm^–1^; NMR (600 MHz, MeOD-*d*_4_), see Table 4; HRESIMS *m*/*z* 1475.52185 [M + H]^+^ (calcd for C_66_H_91_O_37_).

#### Gomphandranoside K (**10**)

White amorphous powder; [α]^20^_D_ −157.2 (*c* 0.10, MeOH); UV (MeOH) λ_*max*_ 231 nm; IR (ATR) υ_*max*_ 1060, 1527, 1706, 3378, 3860 cm^–1^; NMR (600 MHz, MeOD-*d*_4_) see Table 4; HRESIMS *m*/*z* 1475.52258 [M + H]^+^ (calcd for C_66_H_91_O_37_).

### Monosaccharide Identification

The sugar analysis was carried out using a polysaccharide assay kit, following a standard reference protocol.^52−54^ A 0.5 mL portion of hydrolysis reagent was added to the sample (1.0 mg to 2.0 mg), and the mixture was heated at 80 °C for 60 min. Afterward, the reaction mixture was dried under a vacuum. Next, 1.5 mg of reagent A (naphthimidazole) and 1.0 mL of reagent B (iodine) were added to the dried product, and the mixture was stirred for 60 min. This reaction yielded a solution that was subsequently dried. The residue was then reconstituted in 1.0 mL of deionized H_2_O, followed by centrifugation at 3000 rpm for 20 min. The supernatant was filtered through a 0.22 μm membrane before LC-MS analysis. Sugar standards were prepared similarly without the hydrolysis step.

The UPLC-MS mobile phase consisted of channels A (0.1% HCOOH in MeCN) and B (0.1% HCOOH in H_2_O). The gradient conditions were as follows: 0 min (channel A at 7%), 6 min (channel A at 7%), 8 min (channel A at 13%), 10 min (channel A at 18%), 10.5 min (channel A at 50%), 12 min (channel A at 50%), 12.1 min (channel A at 7%), and 17 min (channel A at 7%). The flow rate was set at 0.3 μL/min, and the injection volume was 10.0 μL. The analysis was conducted on an ACQUITY UPLC BEH C18 column (2.1 × 100 mm, 1.7 μm; Waters). For the mass spectrometry (MS) analysis, electrospray ionization (ESI) was performed in both positive and negative ion modes using Xcalibur software for control. The ESI parameters included spray voltages of 3.5 kV at positive modes, with capillary and source heater temperatures maintained at 360 °C. d-Glucose-NAIM and d-galactose-NAIM were detected as ions with *m*/*z* = 319, and d-fructose-NAIM was detected as ions with *m*/*z* = 289.

### NMR Calculation and DP4+ Analysis

The 3D structures were created by Chem3D 22.0; then all possible conformers of isomers were found by Spartan’14 by using MMFF molecular force fields.^55^ The conformers were initially preoptimized using the PM3 semiempirical method, followed by refinement with the Hartree–Fock method employing the 3-21G basis set. Stable conformers with a population distribution above 1% were further subjected to optimization and frequency calculations using Gaussian 16W software. These steps utilized the DFT method with the B3LYP functional and the 6-31G(d,p) basis set. Subsequently, conformers without imaginary frequencies were selected for NMR chemical shift calculations, which were performed using the gauge-independent atomic orbital (GIAO) method with the mPW1PW91 functional and the 6-311+G(d,p) basis set, considering methanol as the solvent. The NMR chemical shift values were calculated based on the Boltzmann distribution. The correlation between the calculated and experimental NMR shifts was evaluated through linear regression analysis to obtain the linear correlation coefficients (*R*^2^), while DP4+ probability calculations were also used to identify the most likely isomers.^56−59^

### Anti-inflammatory Effects Based on Inhibition of Nitric Oxide Production Assay

#### Cell Culture and Cell Viability

RAW264.7 cells were cultured in Dulbecco’s modified essential medium (DMEM, Gibco). The cells were incubated at 37 °C in DMEM with 10% fetal bovine serum (FBS, Gibco), penicillin (100 U/mL, Gibco), and streptomycin sulfate (100 μg/mL, Gibco) under a humidified atmosphere containing 5% CO_2_. Briefly, 2 × 10^4^ cells/well treated for 24 h with vehicle or compounds were examined for cell viability based on the MTT (3-(4,5-dimethylthiazol-2-yl)-2,5-diphenyltetrazolium bromide assay) method.^60^ Survival of macrophage cells after treatment with compounds was measured at a wavelength of 570 nm.

#### Determination of NO Production

RAW264.7 cells (2 × 10^4^ cells/well) were stimulated with or without 1 μg/mL lipopolysaccharide (LPS, Sigma) for 24 h in the presence or absence of the test compounds at different concentrations. Afterward, 100 μL of the cell culture supernatant was mixed with 100 μL of Griess reagent.^61^ The remaining cells from the Griess assay were then utilized to determine their viability by using the MTT colorimetric assay described above.

### Hepatoprotective Effect in Acetaminophen-Induced Hepatotoxicity in the HepG2 Cell Model

Human hepatocellular carcinoma HepG2 cells were cultured in DMEM (Gibco) supplemented with 10% FBS (Gibco) and 1% antibiotic–antimycotic (Gibco, 15240062). Once cells reached approximately 80% confluence, they were washed with PBS and detached using trypsin-EDTA (Gibco, 25200072) at 37 °C for 5 min. Cells were then seeded into 96-well plates at a density of 3 × 10^4^ cells per well (100 μL/well) and incubated for 24 h at 37 °C in a humidified atmosphere with 5% CO_2_.

After the initial incubation, the medium was replaced with 100 μL of fresh medium containing the test compounds, and the cells were incubated for an additional 48 h. Cell viability was determined using the MTT assay, following standard protocols with untreated cells serving as the control. The percentage of cell viability was calculated using the following formula: % viability = [(number of viable cells)/(total cells)] × 100. The concentration of the test compounds was set at 80 μM for the screening.

Compounds that maintained cell viability above 80% were subsequently tested for their hepatoprotective properties using an acetaminophen-induced hepatotoxicity model in HepG2 cells. For this assay, HepG2 cells were again seeded at a density of 3 × 10^4^ cells per well and incubated for 24 h. Afterward, the cells were treated with the test compounds. Eight hours later, hepatotoxicity was induced by adding acetaminophen (6 mM, Sigma-Aldrich A7085) to the wells. Cell viability was measured using the MTT assay 36 h after acetaminophen exposure, and the results were used to assess the hepatoprotective effects of the compounds.

### Statistical Analysis

Statistical analysis and graphical charts were performed using GraphPad Prism 9.5.0. Data are presented as the mean ± standard deviation (SD) and were analyzed using a one-way ANOVA, followed by Tukey’s posthoc multiple comparison test. Statistical significance was defined as a *P* value of less than 0.05.

## Data Availability

The NMR data for the following compounds have been deposited in the Natural Products Magnetic Resonance Database (NP-MRD; www.np-mrd.org) and can be found at NP0350652 (Gomphandranoside A), NP0350653 (Gomphandranoside B), NP0350654 (Gomphandranoside C), NP0350655 (Gomphandranoside D), NP0350656 (Gomphandranoside E), NP0350657 (Gomphandranoside F), NP0350658 (Gomphandranoside G), NP0350659 (Gomphandranoside H), NP0350660 (Gomphandranoside I), and NP0350661 (Gomphandranoside K).

## Supporting Information Available

Spectroscopic data of compounds **1**–**10**; UHPLC-MS chromatogram for monosaccharide identification of compounds **1**–**10**; NMR calculation and DP4+ analysis of compounds **1**–**3** (PDF)

## References

[ref1] Ojeaburu S. I.; Oriakhi K. Hepatoprotective, Antioxidant and, Anti-Inflammatory Potentials of Gallic Acid in Carbon Tetrachloride-Induced Hepatic Damage in Wistar Rats. Toxicol. Rep. 2021, 8, 177–185. 10.1016/j.toxrep.2021.01.001. 33489777 PMC7806503

[ref2] Gupta A.; Kumar R.; Ganguly R.; Singh A. K.; Rana H. K.; Pandey A. K. Antioxidant, Anti-Inflammatory and Hepatoprotective Activities of Terminalia Bellirica and Its Bioactive Component Ellagic Acid against Diclofenac Induced Oxidative Stress and Hepatotoxicity. Toxicol. Rep. 2021, 8, 44–52. 10.1016/j.toxrep.2020.12.010. 33391996 PMC7772792

[ref3] Song X.; Liu Z.; Zhang J.; Yang Q.; Ren Z.; Zhang C.; Liu M.; Gao Z.; Zhao H.; Jia L. Anti-Inflammatory and Hepatoprotective Effects of Exopolysaccharides Isolated from Pleurotus Geesteranus on Alcohol-Induced Liver Injury. Sci. Rep. 2018, 8 (1), 10493 10.1038/s41598-018-28785-0. 30002448 PMC6043593

[ref4] Salem G. A.; Alamyel F. B.; Abushaala F. A.; Elnory M. A.; Abusheba H.; Sahu R. P. Evaluation of the Hepatoprotective, Anti-Inflammatory, Antinociceptive and Antiepileptic Activities of Chrysanthemum Trifurcatum. Biomed. Pharmacother. 2019, 117, 109123 10.1016/j.biopha.2019.109123. 31234026

[ref5] Arroyo V.; Moreau R.; Kamath P. S.; Jalan R.; Ginès P.; Nevens F.; Fernández J.; To U.; García-Tsao G.; Schnabl B. Acute-on-Chronic Liver Failure in Cirrhosis. Nat. Rev. Dis. Primers 2016, 2, 16041 10.1038/nrdp.2016.41. 27277335

[ref6] Singh S.; Muir A. J.; Dieterich D. T.; Falck-Ytter Y. T. American Gastroenterological Association Institute Technical Review on the Role of Elastography in Chronic Liver Diseases. Gastroenterology 2017, 152 (6), 1544–1577. 10.1053/j.gastro.2017.03.016. 28442120

[ref7] Scotece M.; Conde-Aranda J. Inflammation in Health and Disease: New Insights and Therapeutic Avenues. Int. J. Mol. Sci. 2022, 23 (15), 8392 10.3390/ijms23158392. 35955527 PMC9369237

[ref8] Furman D.; Campisi J.; Verdin E.; Carrera-Bastos P.; Targ S.; Franceschi C.; Ferrucci L.; Gilroy D. W.; Fasano A.; Miller G. W.; Miller A. H.; Mantovani A.; Weyand C. M.; Barzilai N.; Goronzy J. J.; Rando T. A.; Effros R. B.; Lucia A.; Kleinstreuer N.; Slavich G. M. Chronic Inflammation in the Etiology of Disease across the Life Span. Nat. Med. 2019, 25 (12), 1822–1832. 10.1038/s41591-019-0675-0. 31806905 PMC7147972

[ref9] Dinda B.; Debnath S.; Harigaya Y. Naturally Occurring Iridoids. A Review, Part 1. Chem. Pharm. Bull. 2007, 55 (2), 159–222. 10.1248/cpb.55.159. 17268091

[ref10] Dinda B.; Debnath S.; Harigaya Y. Naturally Occurring Secoiridoids and Bioactivity of Naturally Occurring Iridoids and Secoiridoids. A Review, Part 2. Chem. Pharm. Bull. 2007, 55 (5), 689–728. 10.1248/cpb.55.689. 17473457

[ref11] Dinda B.; Roy Chowdhury D.; Mohanta B. C. Naturally Occurring Iridoids, Secoiridoids and Their Bioactivity. An Updated Review, Part 3. Chem. Pharm. Bull. 2009, 57 (8), 765–96. 10.1248/cpb.57.765. 19652401

[ref12] Dinda B.; Debnath S.; Banik R. Naturally Occurring Iridoids and Secoiridoids. An Updated Review, Part 4. Chem. Pharm. Bull. 2011, 59 (7), 803–833. 10.1248/cpb.59.803. 21720031

[ref13] Huy Bich D.; Quang Chung D.; Xuan Chuong B.; Thuong Dong N.; Trung Dam D.; Van Hien P.; Ngoc Lo V.; Duy Mai P.; Kim Man P.; Thi Nhu D.; Tap N.; Toan T. Cây Thuoc Và Đong Vat Làm Thuoc O Viet Nam; Science and Technics Publishing House, 2006, Vol. 1, pp 249–251.

[ref14] Ramesha B. T.; Suma H. K.; Senthilkumar U.; Priti V.; Ravikanth G.; Vasudeva R.; Kumar T. R. S.; Ganeshaiah K. N.; Shaanker R. U. New Plant Sources of the Anti-Cancer Alkaloid, Camptothecine from the Icacinaceae Taxa, India. Phytomedicine 2013, 20 (6), 521–527. 10.1016/j.phymed.2012.12.003. 23474217

[ref15] Haron N. W.; Ping S. T. Distribution and Taxonomic Significance of Flavonoids in the Olacaceae and Icacinaceae. Biochem. Syst. Ecol. 1997, 25 (3), 263–265. 10.1016/S0305-1978(96)00105-6.

[ref16] Kamperdick C. Constituents from Gomphandra Tetranda (Icacinaceae). Viet. J. Chem. 2002, 3 (40), 108–110.

[ref17] Graikou K.; Aligiannis N.; Chinou I. B.; Harvala C. Cantleyoside-Dimethyl-Acetal and Other Iridoid Glucosides from Pterocephalus Perennis - Antimicrobial Activities. Z. Naturforsch. C 2002, 57 (1–2), 95–99. 10.1515/znc-2002-1-217. 11926551

[ref18] CAMBIE R. C.; LAL A. R.; RICKARD C. E. F.; TANAKA N. Chemistry of Fijian Plants: V: Constituents of Fagraea Gracilipes A. Gray. Chem. Pharm. Bull. (Tokyo) 1990, 38 (7), 1857–1861. 10.1248/cpb.38.1857.

[ref19] Kumar S.; Sati O. P.; Semwal V. D.; Nautiyal M.; Sati S.; Takeda Y. Iridoid Glycosides from Lonicera Quinquelocularis. Phytochemistry 2000, 53 (4), 499–501. 10.1016/S0031-9422(99)00426-4. 10731029

[ref20] Tian X. Y.; Wang Y. H.; Yu S. S.; Fang W. S. Two Novel Tetrairidoid Glucosides from Dipsacus Asper. Org. Lett. 2006, 8 (10), 2179–2182. 10.1021/ol060676k. 16671811

[ref21] Yu Z. P.; Wang Y. Y.; Yu S. J.; Bao J.; Yu J. H.; Zhang H. Absolute Structure Assignment of an Iridoid-Monoterpenoid Indole Alkaloid Hybrid from Dipsacus Asper. Fitoterapia 2019, 135, 99–106. 10.1016/j.fitote.2019.04.015. 31051193

[ref22] Garaev E. E.; Mahiou-Leddet V.; Mabrouki F.; Herbette G.; Garaev E. A.; Ollivier E. Chemical Constituents from Roots of Cephalaria Media. Chem. Nat. Compd. 2014, 50 (4), 756–758. 10.1007/s10600-014-1075-9.

[ref23] Hase T.; Takao H.; Iwagawa T. The Bitter Iridoids from Viburnum Urceolatum. Phytochemistry 1983, 22 (9), 1977–1982. 10.1016/0031-9422(83)80027-2.

[ref24] Podányi B.; Reid R. S.; Kocsis A.; Szabo L. Laciniatoside V: A New Bis-Iridoid Glucoside. Isolation and Structure Elucidation by 2d Nmr Spectroscopy. J. Nat. Prod. 1989, 52 (1), 135–142. 10.1021/np50061a017.

[ref25] Tomita H.; Mouri Y. An Iridoid Glucoside from Dipsacus Asperoides. Phytochemistry 1996, 42 (1), 239–240. 10.1016/0031-9422(95)00904-3. 8728068

[ref26] Damtoft S.; Franzyk H.; Jensen S. R. Iridoid Glucosides from Picconia Excelsa. Phytochemistry 1997, 45 (4), 743–750. 10.1016/S0031-9422(97)00023-X.

[ref27] Müller A. A.; Kufer J. K.; Dietl K. G.; Weigend M. A Dimeric Iridoid from Loasa Acerifolia. Phytochemistry 1998, 49 (6), 1705–1707. 10.1016/S0031-9422(98)00194-0. 11711085

[ref28] Tomassini L.; Foddai S.; Serafini M.; Cometa M. F. Bis-Iridoid Glucosides from Abelia Chinensis. J. Nat. Prod. 2000, 63 (7), 998–999. 10.1021/np9904713. 10924185

[ref29] Yuan H. Y.; Kwaku O. R.; Pan H.; Han J. X.; Yang C. R.; Xu M. Iridoid Glycosides from the Genus Gentiana (Gentianaceae) and Their Chemotaxonomic Sense. Nat. Prod. Commun. 2017, 12 (10), 1663–1670. 10.1177/1934578X1701201035.

[ref30] Dinda B. Chemistry and Biosynthesis of Iridoids. In Pharmacology and Applications of Naturally Occurring Iridoids 2019, 119–143. 10.1007/978-3-030-05575-2_4.

[ref31] Nagatoshi M.; Terasaka K.; Nagatsu A.; Mizukami H. Iridoid-Specific Glucosyltransferase from Gardenia Jasminoides. J. Biol. Chem. 2011, 286 (37), 32866–32874. 10.1074/jbc.M111.242586. 21799001 PMC3173207

[ref32] Miettinen K.; Dong L.; Navrot N.; Schneider T.; Burlat V.; Pollier J.; Woittiez L.; Van Der Krol S.; Lugan R.; Ilc T.; Verpoorte R.; Oksman-Caldentey K. M.; Martinoia E.; Bouwmeester H.; Goossens A.; Memelink J.; Werck-Reichhart D. The Seco-Iridoid Pathway from Catharanthus Roseus. Nat. Commun. 2014, 5, 3606 10.1038/ncomms4606. 24710322 PMC3992524

[ref33] Kanchanapoom T.; Picheansoonthon C.; Kasai R.; Yamasaki K. New Glucosides from Thai Medicinal Plant, Balanophora Latisepala. Nat. Med. 2001, 55 (4), 213–216.

[ref34] Itoh A.; Kumashiro T.; Yamaguchi M.; Nagakura N.; Mizushina Y.; Nishi T.; Tanahashi T. Indole Alkaloids and Other Constituents of Rauwolfia Serpentina. J. Nat. Prod. 2005, 68 (6), 848–852. 10.1021/np058007n. 15974606

[ref35] Itoh A.; Tanahashi T.; Tabata M.; Shikata M.; Kakite M.; Nagai M.; Nagakura N. Tetrahydroisoquinoline-Monoterpene and Iridoid Glycosides from Alangium Lamarckii. Phytochemistry 2001, 56 (6), 623–630. 10.1016/S0031-9422(00)00417-9. 11281140

[ref36] Olennikov D. N.; Chirikova N. K. Phlotuberosides I and II, New Iridoid Glycosides from Phlomoides Tuberosa. Chem. Nat. Compd. 2017, 53 (2), 269–272. 10.1007/s10600-017-1968-5.

[ref37] Zhang F.; Liu H.; Yang K.; Yang T.; Zhou R.; Miao R.; Zhan G.; Guo Z. New Phenylpropanoids and Monoterpene Alkaloids with Vasorelaxant Activities from the Branches of Alstonia Scholaris. Fitoterapia 2022, 158, 105143 10.1016/j.fitote.2022.105143. 35124162

[ref38] Sakai H.; Kakuda R.; Yaoita Y.; Kikuchi M. Secoiridoid Glycosides from the Leaves of Hydrangea Macrophylla Subsp. Serrata. J. Nat. Med. 2007, 61 (2), 226–228. 10.1007/s11418-006-0123-6.

[ref39] Lee E. J.; Lee J. Y.; Kim J. S.; Kang S. S. Phytochemical Studies on Lonicerae Flos (1) - Isolation of Iridoid Glycosides and Other Constituents. Nat. Prod. Sci. 2010, 16 (1), 32–38.

[ref40] Cimanga R. K.; Nsaka S. L.; Tshodi M. E.; Mbamu B. M.; Kikweta C. M.; Makila F. B. M.; Cos P.; Maes L.; Vlietinck A. J.; Exarchou V.; Tuenter E.; Pieters L. In Vitro and in Vivo Antiplasmodial Activity of Extracts and Isolated Constituents of Alstonia Congensis Root Bark. J. Ethnopharmacol 2019, 242, 111736 10.1016/j.jep.2019.02.019. 30763695

[ref41] Richards K.; Tran K.; Levine R.; Luo R.; Maia J.; Sabaa-Srur A.; Maciel M.; Melo E.; Moraes M.; Godoy H.; Chaves M.; Sacramento C.; Thomas A.; Monroe D.; Smith R. Improved Extraction of Soluble Solids from Some Brazilian and North American Fruits. Nat. Prod. J. 2014, 4 (3), 201–210. 10.2174/2210315504666141112222818.

[ref42] Hussain H.; Nazir M.; Green I. R.; Saleem M.; Raza M. L. Therapeutic Potential of Iridoid Derivatives: Patent Review. Inventions. 2019, 4 (2), 29 10.3390/inventions4020029.

[ref43] Viljoen A.; Mncwangi N.; Vermaak I. Anti-Inflammatory Iridoids of Botanical Origin. Curr. Med. Chem. 2012, 19 (14), 2104–2127. 10.2174/092986712800229005. 22414102 PMC3873812

[ref44] Wang J. W.; Pan Y. B.; Cao Y. Q.; Wang C.; Jiang W. D.; Zhai W. F.; Lu J. G. Loganin Alleviates LPS-Activated Intestinal Epithelial Inflammation by Regulating TLR4/NF-KB and JAK/STAT3 Signaling Pathways. Kaohsiung J. Med. Sci. 2020, 36 (4), 257–264. 10.1002/kjm2.12160. 31859422 PMC11896463

[ref45] Park C.; Lee H.; Kwon C. Y.; Kim G. Y.; Jeong J. W.; Kim S. O.; Choi S. H.; Jeong S. J.; Noh J. S.; Choi Y. H. Loganin Inhibits Lipopolysaccharide-Induced Inflammation and Oxidative Response through the Activation of the Nrf2/HO-1 Signaling Pathway in RAW264.7 Macrophages. Biol. Pharm. Bull. 2021, 44 (6), 875–883. 10.1248/bpb.b21-00176. 34078820

[ref46] Wang J.; Cai X.; Ma R.; Lei D.; Pan X.; Wang F. Anti-Inflammatory Effects of Sweroside on LPS-Induced ALI in Mice Via Activating SIRT1. Inflammation 2021, 44 (5), 1961–1968. 10.1007/s10753-021-01473-4. 33913051

[ref47] Bai J.; Xie N.; Hou Y.; Chen X.; Hu Y.; Zhang Y.; Meng X.; Wang X.; Tang C. The Enhanced Mitochondrial Dysfunction by Cantleyoside Confines Inflammatory Response and Promotes Apoptosis of Human HFLS-RA Cell Line via AMPK/Sirt 1/NF-KB Pathway Activation. Biomed. Pharmacother. 2022, 149, 112847 10.1016/j.biopha.2022.112847. 35364376

[ref48] Bridi R.; von Poser G. L.; de Carvalho Meirelles G. Iridoids as a Potential Hepatoprotective Class: A Review. Mini-Rev. Med. Chem. 2023, 23 (4), 452–479. 10.2174/1389557522666220816130158. 35975865

[ref49] Dai K.; Yi X. J.; Huang X. J.; Muhammad A.; Li M.; Li J.; Yang G. Z.; Gao Y. Hepatoprotective Activity of Iridoids, Seco-Iridoids and Analog Glycosides from Gentianaceae on HepG2 Cells: Via CYP3A4 Induction and Mitochondrial Pathway. Food Funct. 2018, 9 (5), 2673–2683. 10.1039/C8FO00168E. 29675530

[ref50] Liu Y. F.; Liang D.; Luo H.; Hao Z. Y.; Wang Y.; Zhang C. L.; Zhang Q. J.; Chen R. Y.; Yu D. Q. Hepatoprotective Iridoid Glycosides from the Roots of Rehmannia Glutinosa. J. Nat. Prod. 2012, 75 (9), 1625–1631. 10.1021/np300509z. 22916954

[ref51] Peng W.; Qiu X. Q.; Shu Z. H.; Liu Q. C.; Hu M. B.; Han T.; Rahman K.; Qin L. P.; Zheng C. J. Hepatoprotective Activity of Total Iridoid Glycosides Isolated from Paederia Scandens (Lour.) Merr. Var. Tomentosa. J. Ethnopharmacol 2015, 174, 317–321. 10.1016/j.jep.2015.08.032. 26320683

[ref52] Lin C.; Hung W. T.; Kuo C. Y.; Liao K. S.; Liu Y. C.; Yang W. B. I2-Catalyzed Oxidative Condensation of Aldoses with Diamines: Synthesis of Aldo-Naphthimidazoles for Carbohydrate Analysis. Molecules 2010, 15 (3), 1340–1353. 10.3390/molecules15031340. 20335985 PMC6257232

[ref53] Lin C.; Kuo C. Y.; Liao K. S.; Yang W. B. Monosaccharide-NAIM Derivatives for D-, L-Configurational Analysis. Molecules 2011, 16 (1), 652–664. 10.3390/molecules16010652. 21242944 PMC6259221

[ref54] Vo T. H.; Lin Y. C.; Liaw C. C.; Pan W. P.; Cheng J. J.; Lee C. K.; Kuo Y. H. Triterpene Glycosides and Phenylpropane Derivatives from Staurogyne Concinnula Possessing Anti-Angiogenic Activity. Phytochemistry 2021, 184, 112666 10.1016/j.phytochem.2021.112666. 33524858

[ref55] Zeb M. A.; Pu X.; Wang M. R.; Kong Y. L.; Yang Q. Y.; Tu W. C.; Li X. L.; Li H. L.; Xiao W. L. Structural Characterization of Two New Stereoisomeric Furofuran Lignans Using Modern Spectroscopic Methods and Quantum Chemical Calculation. J. Mol. Struct. 2024, 1308, 138076 10.1016/j.molstruc.2024.138076.

[ref56] Grimblat N.; Sarotti A. M. Computational Chemistry to the Rescue: Modern Toolboxes for the Assignment of Complex Molecules by GIAO NMR Calculations. Chem.—Eur. J. 2016, 22 (35), 12246–12461. 10.1002/chem.201601150. 27405775

[ref57] Shi Q. Q.; Zhang X. J.; Wang T. T.; Wang Q.; Sun T. T.; Amin M.; Zhang R. H.; Li X. L.; Xiao W. L. Euphopias A-C: Three Rearranged Jatrophane Diterpenoids with Tricyclo[8.3.0.02,7]Tridecane and Tetracyclo[11.3.0.02,10.03,7]Hexadecane Cores from Euphorbia Helioscopia. Org. Lett. 2020, 22 (20), 7820–7824. 10.1021/acs.orglett.0c02676. 32991190

[ref58] Tran Huynh Q.-D.; Hsu S.-J.; Duong T.-L. T.; Liu H.-K.; Liu T.-W.; Chu M.-H.; Wang Y.-H.; Nguyen D.-K.; Phan T.-T. T.; Tran N.-K. H.; Vo T.-H.; Hsi H.-Y.; Yeh T.-W.; Lee C.-K. New Hydrogenated Phenanthrene Glycosides from the Edible Vegetable Elatostema Tenuicaudatum W.T.Wang with DPP-IV Inhibitory and Hepatoprotective Activity. J. Agric. Food. Chem. 2025, 73 (2), 1273–1292. 10.1021/acs.jafc.4c08713. 39761081 PMC11741115

[ref59] Grimblat N.; Zanardi M. M.; Sarotti A. M. Beyond DP4: An Improved Probability for the Stereochemical Assignment of Isomeric Compounds Using Quantum Chemical Calculations of NMR Shifts. J. Org. Chem. 2015, 80 (24), 12526–12534. 10.1021/acs.joc.5b02396. 26580165

[ref60] Mosmann T. Rapid Colorimetric Assay for Cellular Growth and Survival: Application to Proliferation and Cytotoxicity Assays. J. Immunol. Methods 1983, 65 (1–2), 55–63. 10.1016/0022-1759(83)90303-4. 6606682

[ref61] Tran H. N. K.; Cao T. Q.; Kim J. A.; Youn U. J.; Kim S.; Woo M. H.; Min B. S. Anti-Inflammatory Activity of Compounds from the Rhizome of Cnidium Officinale. Arch. Pharm. Res. 2018, 41 (10), 977–985. 10.1007/s12272-018-1048-9. 29961195

