# Supplementary material for: Anti-inflammatory and Hepatoprotective Iridoid Glycosides from the Roots of Gomphandra mollis
Source: J Nat Prod. 2025 Feb 13;88(2):577–92. doi: 10.1021/acs.jnatprod.4c01484 (PMC11877508; doi:10.1021/acs.jnatprod.4c01484)
Supplement: Supplementary file 1 — np4c01484_si_001.pdf [file np4c01484_si_001.pdf]

SUPPORTING INFORMATION

Anti-inflammatory and Hepatoprotective Iridoid Glycosides from the Roots of *Gomphandra mollis*

Quoc-Dung Tran Huynh,[a,b] *Thuy-Tien Thi Phan,*[b,c] *Ta-Wei Liu,*[d] *Thanh-Vu Nguyen,*[e] *Truc-Ly Thi Duong,*[f] *Su-Jung Hsu,*[d,l] *Man-Hsiu Chu,*[d] *Yun-Han Wang,*[a] *Bien-Thuy Nguyen Bui,*[a,h,i] *Dang-Khoa Nguyen,*[g] *Thanh-Hoa Vo,*[i,k] *Ching-Kuo Lee* [a,d,m,n*]

[a] Ph.D. Program in Clinical Drug Development of Herbal Medicine, College of Pharmacy, Taipei Medical University, Taipei 11031, Taiwan
[b] Institute of Pharmaceutical Education and Research, Binh Duong University, Thu Dau Mot 820000, Binh Duong, Vietnam
[c] Graduate Institute of Biomedical Materials and Tissue Engineering, College of Biomedical Engineering, Taipei Medical University, Taipei 11031, Taiwan
[d] School of Pharmacy, College of Pharmacy, Taipei Medical University, Taipei 11042, Taiwan
[e] Biotechnology Center Of Ho Chi Minh City, Ho Chi Minh City 700000, Vietnam
[f] Faculty of Traditional medicine, Can Tho University of Medicine and Pharmacy, Can Tho 900000, Vietnam
[g] Faculty of Pharmacy, Ton Duc Thang University, Ho Chi Minh City 700000, Vietnam
[h] National Research Institute of Chinese Medicine, Ministry of Health and Welfare, Taipei 11221, Taiwan
[i] University of Health Sciences, Vietnam National University Ho Chi Minh City, Ho Chi Minh 700000, Vietnam
[k] Center for Discovery and Development of Healthcare Product, Vietnam National University Ho Chi Minh City, Ho Chi Minh 700000, Vietnam
[l] Institute of Fisheries Science, National Taiwan University, Taipei 106, Taiwan
[m] Graduate Institute of Pharmacognosy, College of Pharmacy, Taipei Medical University, Taipei 11042, Taiwan
[n] Department of Chemistry, Chung Yuan Christian University, Zhongli District, Taoyuan 32023, Taiwan
* Correspondence: cklee@tmu.edu.tw; Tel.: + 886-2-27361661 ext. 6150

S1. Spectroscopic data of compounds 1-10 1
Figure S1. ^{1}H-NMR spectrum of compound **1** (600 MHz, CD_3OD) 1
Figure S2. ^{13}C-NMR spectrum of compound **1** (150 MHz, CD_3OD) 1
Figure S3. DEPT-NMR spectrum of compound **1** (150 MHz, CD_3OD) 2
Figure S4. HSQC spectrum of compound **1** (600 MHz, CD_3OD) 2
Figure S5. COSY spectrum of compound **1** (600 MHz, CD_3OD) 3
Figure S6. HMBC spectrum of compound **1** (600 MHz, CD_3OD) 3
Figure S7. ROESY spectrum of compound **1** (600 MHz, CD_3OD) 4
Figure S8. HR-ESI-MS data of compound **1** 4
Figure S9. UV spectrum (in MeOH) of compound **1** 6
Figure S10. IR (ATR) spectrum of compound **1** 6
Figure S11. ^{1}H-NMR spectrum of compound **2** (600 MHz, CD_3OD) 7
Figure S12. ^{13}C-NMR spectrum of compound **2** (150 MHz, CD_3OD) 7
Figure S13. DEPT-NMR spectrum of compound **2** (150 MHz, CD_3OD) 8
Figure S14. HSQC spectrum of compound **2** (600 MHz, CD_3OD) 8
Figure S15. COSY spectrum of compound **2** (600 MHz, CD_3OD) 9
Figure S16. HMBC spectrum of compound **2** (600 MHz, CD_3OD) 9
Figure S17. ROESY spectrum of compound **2** (600 MHz, CD_3OD) 10
Figure S18. HR-ESI-MS data of compound **2** 10
Figure S19. UV spectrum (in MeOH) of compound **2** 12
Figure S20. IR (ATR) spectrum of compound **2** 12
Figure S21. ^{1}H-NMR spectrum of compound **3** (600 MHz, CD_3OD) 13
Figure S22. ^{13}C-NMR spectrum of compound **3** (150 MHz, CD_3OD) 13
Figure S23. DEPT-NMR spectrum of compound **3** (150 MHz, CD_3OD) 14
Figure S24. HSQC spectrum of compound **3** (600 MHz, CD_3OD) 14
Figure S25. COSY spectrum of compound **3** (600 MHz, CD_3OD) 15
Figure S26. HMBC spectrum of compound **3** (600 MHz, CD_3OD) 15
Figure S27. ROESY spectrum of compound **3** (600 MHz, CD_3OD) 16
Figure S28. HR-ESI-MS data of compound **3** 16
Figure S29. UV spectrum (in MeOH) of compound **3** 18
Figure S30. IR (ATR) spectrum of compound **3** 18
Figure S31. ^{1}H-NMR spectrum of compound **4** (600 MHz, CD_3OD) 19
Figure S32. ^{13}C-NMR spectrum of compound **4** (125 MHz, CD_3OD) 19
Figure S33. DEPT-NMR spectrum of compound **4** (125 MHz, CD_3OD) 20
Figure S34. HSQC spectrum of compound **4** (600 MHz, CD_3OD) 20
Figure S35. COSY spectrum of compound **4** (600 MHz, CD_3OD) 21

Figure S36. HMBC spectrum of compound **4** (600 MHz, CD_3OD) 21
Figure S37. ROESY spectrum of compound **4** (600 MHz, CD_3OD) 22
Figure S38. 2D-TOCSY spectrum of compound **4** (600 MHz, CD_3OD) 22
Figure S39. HR-ESI-MS data of compound **4** 23
Figure S40. UV spectrum (in MeOH) of compound **4** 25
Figure S41. IR (ATR) spectrum of compound **4** 25
Figure S42. ^{1}H-NMR spectrum of compound **5 (**600 MHz, CD_3OD) 26
Figure S43. ^{13}C-NMR spectrum of compound **5** (125 MHz, CD_3OD) 26
Figure S44. DEPT-NMR spectrum of compound **5** (125 MHz, CD_3OD) 27
Figure S45. HSQC spectrum of compound **5** (600 MHz, CD_3OD) 27
Figure S46. COSY spectrum of compound **5** (600 MHz, CD_3OD) 28
Figure S47. HMBC spectrum of compound **5** (600 MHz, CD_3OD) 28
Figure S48. ROESY spectrum of compound **5** (600 MHz, CD_3OD) 29
Figure S49. HR-ESI-MS data of compound **5** 29
Figure S50. UV spectrum (in MeOH) of compound **5** 31
Figure S51. IR (ATR) spectrum of compound **5** 31
Figure S52. ^{1}H-NMR spectrum of compound **6** (600 MHz, CD_3OD) 32
Figure S53. ^{13}C-NMR spectrum of compound **6** (125 MHz, CD_3OD) 32
Figure S54. DEPT-NMR spectrum of compound **6** (125 MHz, CD_3OD) 33
Figure S55. HSQC spectrum of compound **6** (600 MHz, CD_3OD) 33
Figure S56. COSY spectrum of compound **6** (600 MHz, CD_3OD) 34
Figure S57. HMBC spectrum of compound **6** (600 MHz, CD_3OD) 34
Figure S58. ROESY spectrum of compound **6** (600 MHz, CD_3OD) 35
Figure S59. HR-ESI-MS data of compound **6** 35
Figure S60. UV spectrum (in MeOH) of compound **6** 37
Figure S61. IR (ATR) spectrum of compound **6** 37
Figure S62. ^{1}H-NMR spectrum of compound **7** (600 MHz, CD_3OD) 38
Figure S63. ^{13}C-NMR spectrum of compound **7** (125 MHz, CD_3OD) 38
Figure S64. DEPT-NMR spectrum of compound **7** (125 MHz, CD_3OD) 39
Figure S65. HSQC spectrum of compound **7** (600 MHz, CD_3OD) 39
Figure S66. COSY spectrum of compound **7** (600 MHz, CD_3OD) 40
Figure S67. HMBC spectrum of compound **7** (600 MHz, CD_3OD) 40
Figure S68. ROESY spectrum of compound **7** (600 MHz, CD_3OD) 41
Figure S69. HR-ESI-MS data of compound **7** (600 MHz, CD_3OD) 41
Figure S70. UV spectrum (in MeOH) of compound **7** 42
Figure S71. IR (ATR) spectrum of compound **7** 42

Figure S72. ^{1}H-NMR spectrum of compound **8** (600 MHz, CD_3OD) 43
Figure S73. ^{13}C-NMR spectrum of compound **8** (125 MHz, CD_3OD) 43
Figure S74. DEPT-NMR spectrum of compound **8** (125 MHz, CD_3OD) 44
Figure S75. HSQC spectrum of compound **8** (600 MHz, CD_3OD) 44
Figure S76. COSY spectrum of compound **8** (600 MHz, CD_3OD) 45
Figure S77. HMBC spectrum of compound **8** (600 MHz, CD_3OD) 45
Figure S78. ROESY spectrum of compound **8** (600 MHz, CD_3OD) 46
Figure S79. HR-ESI-MS data of compound **8** 46
Figure S80. UV spectrum (in MeOH) of compound **8** 48
Figure S81. IR (ATR) spectrum of compound **8** 48
Figure S82. ^{1}H-NMR spectrum of compound **9** (600 MHz, CD_3OD) 49
Figure S83. ^{13}C-NMR spectrum of compound **9** (125 MHz, CD_3OD) 49
Figure S84. DEPT-NMR spectrum of compound **9** (125 MHz, CD_3OD) 50
Figure S85. HSQC spectrum of compound **9** (600 MHz, CD_3OD) 50
Figure S86. COSY spectrum of compound **9** (600 MHz, CD_3OD) 51
Figure S87. HMBC spectrum of compound **9** (600 MHz, CD_3OD) 51
Figure S88. ROESY spectrum of compound **9** (600 MHz, CD_3OD) 52
Figure S89. HR-ESI-MS data of compound **9** 52
Figure S90. UV spectrum (in MeOH) of compound **9** 54
Figure S91. IR (ATR) spectrum of compound **9** 54
Figure S92. ^{1}H-NMR spectrum of compound **10** (600 MHz, CD_3OD) 55
Figure S93. ^{13}C-NMR spectrum of compound **10** (600 MHz, CD_3OD) 55
Figure S94. DEPT-NMR spectrum of compound **10** (600 MHz, CD_3OD) 56
Figure S95. HSQC spectrum of compound **10** (600 MHz, CD_3OD) 56
Figure S96. COSY spectrum of compound **10** (600 MHz, CD_3OD) 57
Figure S97. HMBC spectrum of compound **10** (600 MHz, CD_3OD) 57
Figure S98. ROESY spectrum of compound **10** (600 MHz, CD_3OD) 58
Figure S99. HR-ESI-MS data of compound **10** 58
Figure S100. UV spectrum (in MeOH) of compound **10** 60
Figure S101. IR (ATR) spectrum of compound **10** 60
S2. UHPLC-MS chromatogram for monosaccharide identification of compounds 1–10 61
Figure S102. UHPLC-MS chromatogram of D-glucose-NAIM (4.92 min), D-galactose-NAIM (5.12 min), D-fructose-NAIM (5.84 min) 61
Figure S103. UHPLC-MS chromatogram of compound **1**'s sugar – NAIM 62
Figure S104. UHPLC-MS chromatogram of compound **2**'s sugar – NAIM 63
Figure S105. UHPLC-MS chromatogram of compound **3**'s sugar – NAIM 63

Figure S106. UHPLC-MS chromatogram of compound **4**'s sugar – NAIM 64
Figure S107. UHPLC-MS chromatogram of compound **5**'s sugar – NAIM 65
Figure S108. UHPLC-MS chromatogram of compound **6**'s sugar – NAIM 66
Figure S109. UHPLC-MS chromatogram of compound **7**'s sugar – NAIM 67
Figure S110. UHPLC-MS chromatogram of compound **8**'s sugar – NAIM 67
Figure S111. UHPLC-MS chromatogram of compound **9**'s sugar – NAIM 68
Figure S112. UHPLC-MS chromatogram of compound **10**'s sugar – NAIM 68
S3. NMR calculation and DP4+ analysis of compound 1 69
Figure S113. The energies and equilibrium populations of compound **1**'s stable conformers 69
Figure S114. Cartesian Coordinates of compound **1**'s stable conformers 70
Figure S115. Comparison of the Calculated and Experimental Proton and Carbon Resonances for compound **1** 74
Figure S116. Assignment of the relative stereochemistry of **2** using DP4+ and representing the DP4+ probabilities for each candidate structure 75
S4. NMR calculation and DP4+ analysis of compound 2 76
Figure S117. The energies and equilibrium populations of compound **2**'s stable conformers 76
Figure S118. Cartesian Coordinates of compound **2**'s stable conformers 77
Figure S119. Comparison of the Calculated and Experimental Proton and Carbon Resonances for compound **2** 82
Figure S120. Assignment of the relative stereochemistry of **2** using DP4+ and representing the DP4+ probabilities for each candidate structure 83
S5. NMR calculation and DP4+ analysis of compound 3 84
Figure S121. The energies and equilibrium populations of compound **3**'s stable conformers 84
Figure S122. Cartesian Coordinates of compound **3**'s stable conformers 85
Figure S123. Comparison of the Calculated and Experimental Proton and Carbon Resonances for compound **3** 89
Figure S124. Assignment of the relative stereochemistry of **3** using DP4+ and representing the DP4+ probabilities for each candidate structure 90

S1. Spectroscopic data of compounds 1-10

Figure S1. ^{1}H-NMR spectrum of compound **1** (600 MHz, CD_3OD)

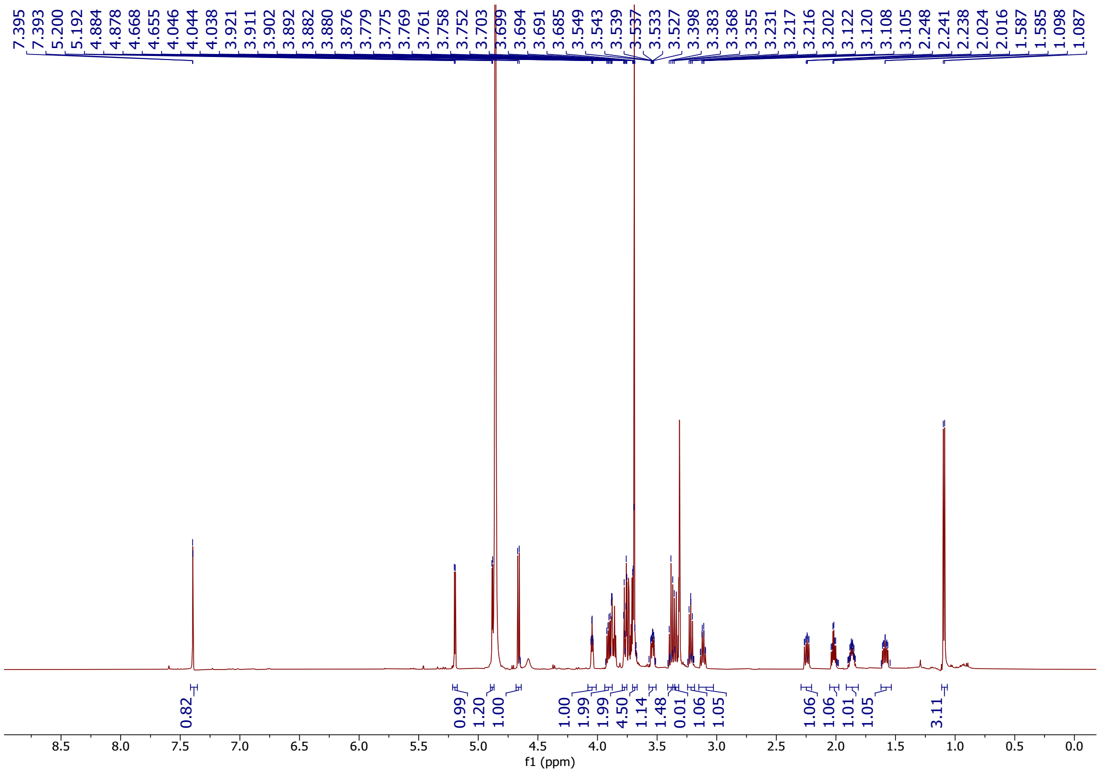

Figure S2. ^{13}C-NMR spectrum of compound **1** (150 MHz, CD_3OD)

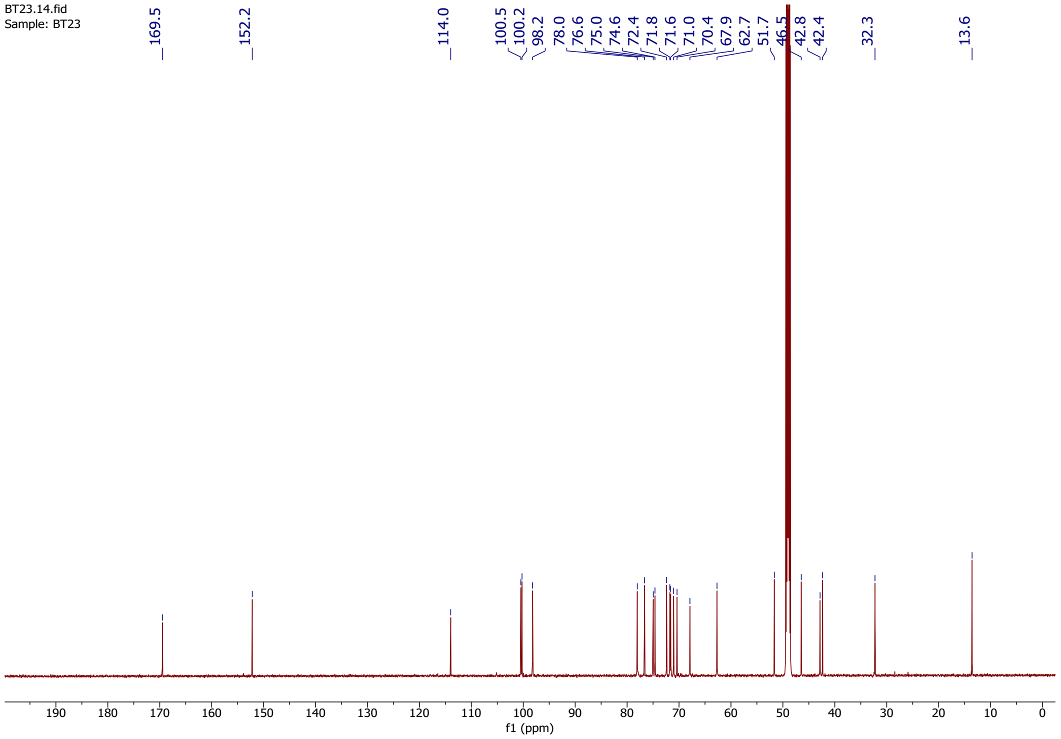

Figure S3. DEPT-NMR spectrum of compound **1** (150 MHz, CD_3OD)

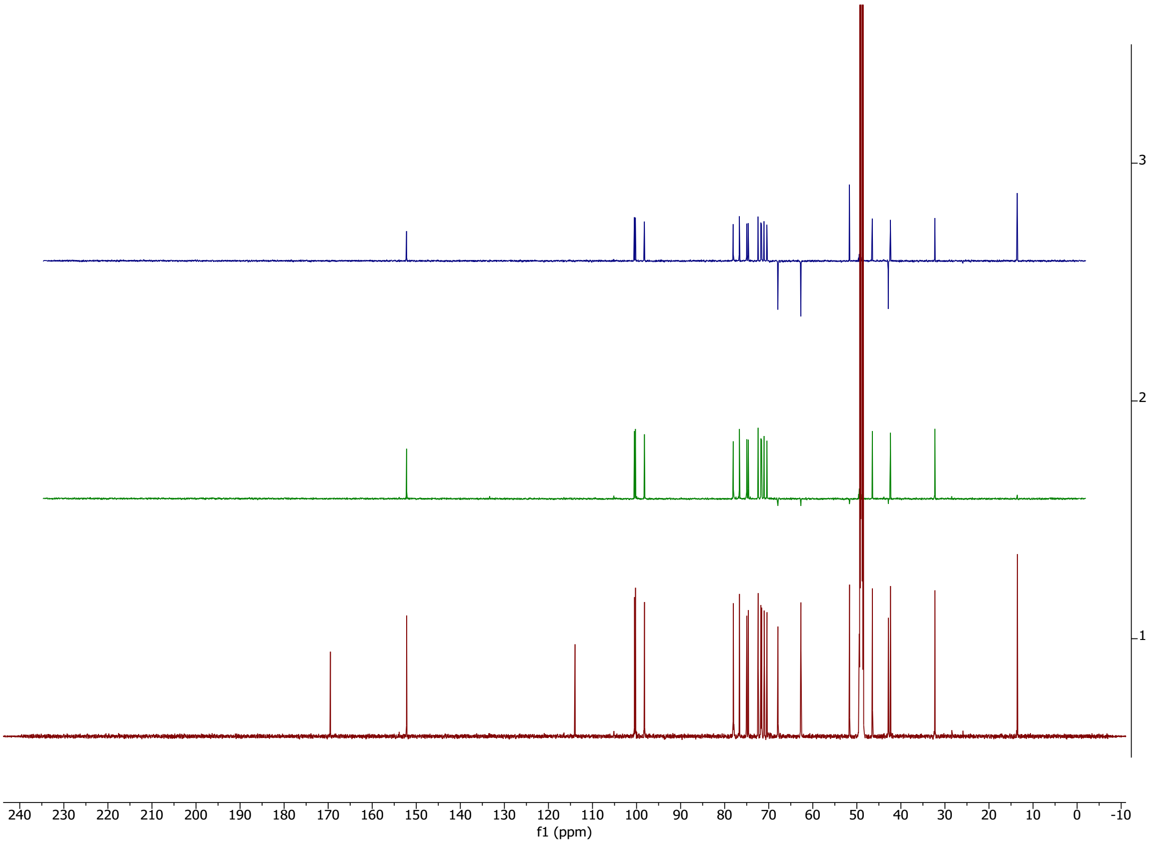

Figure S4. HSQC spectrum of compound **1** (600 MHz, CD_3OD)

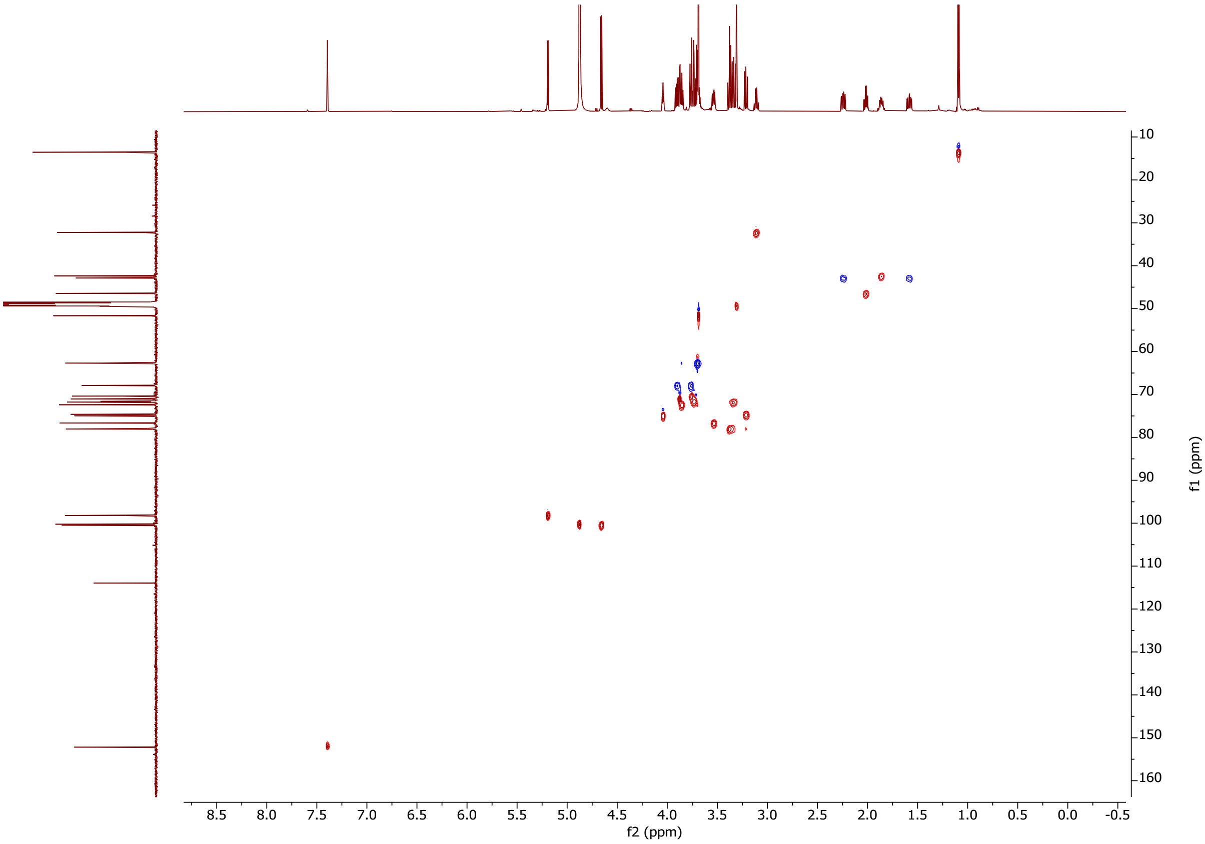

Figure S5. COSY spectrum of compound **1** (600 MHz, CD_3OD)

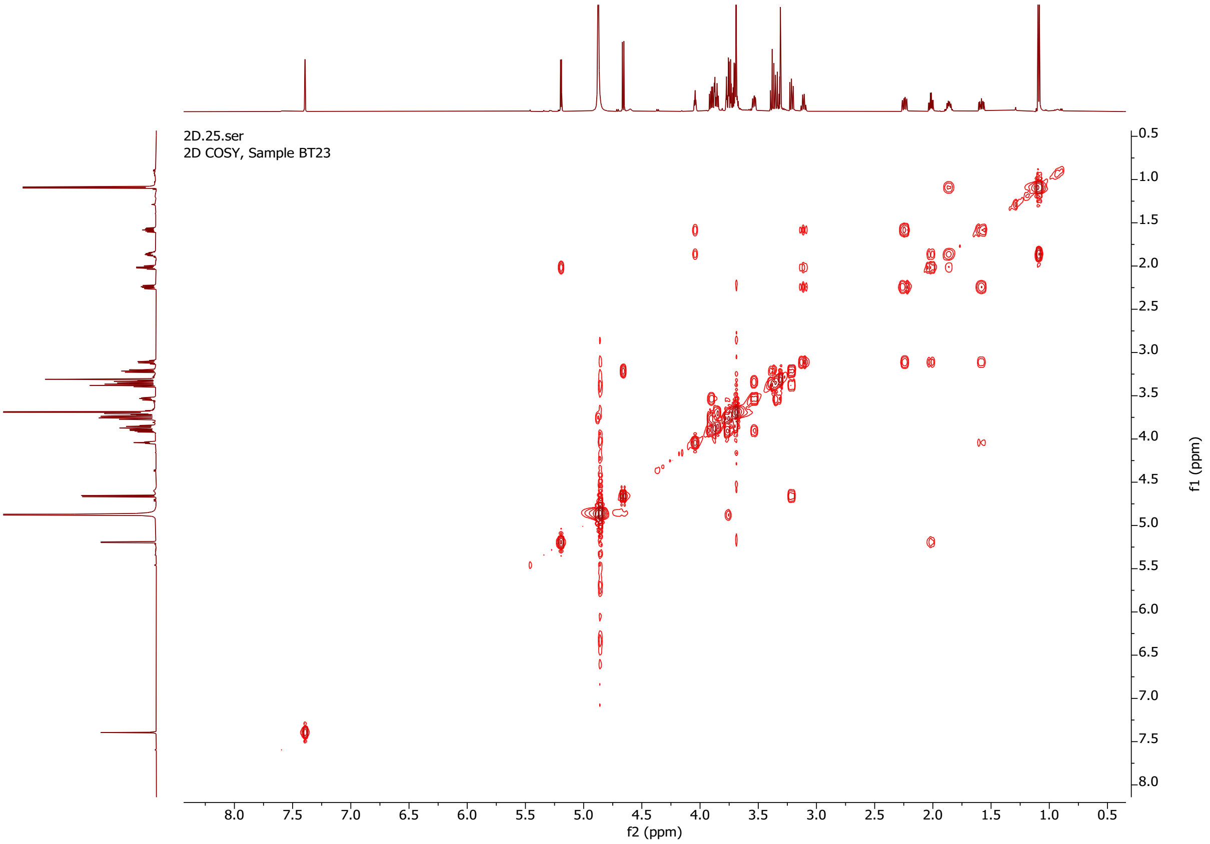

Figure S6. HMBC spectrum of compound **1** (600 MHz, CD_3OD)

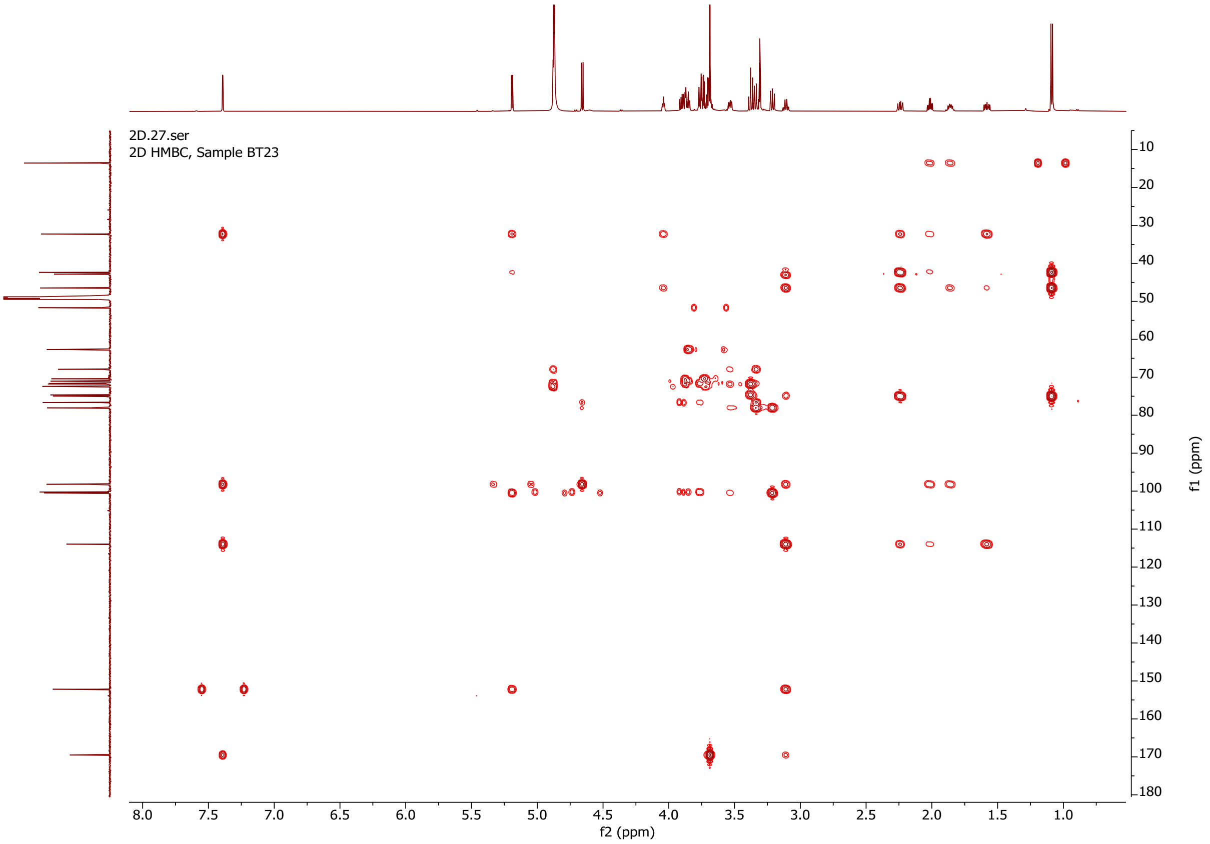

Figure S7. ROESY spectrum of compound **1** (600 MHz, CD_3OD)

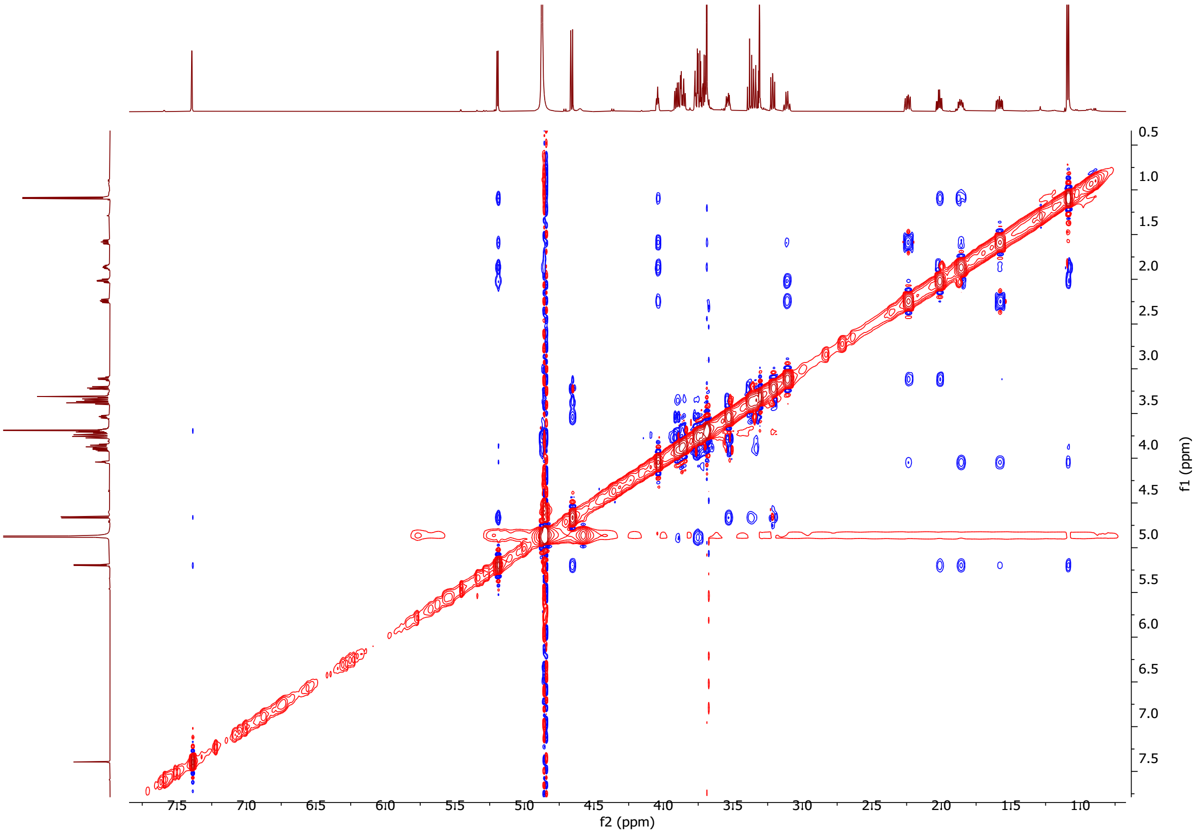

Figure S8. HR-ESI-MS data of compound **1**

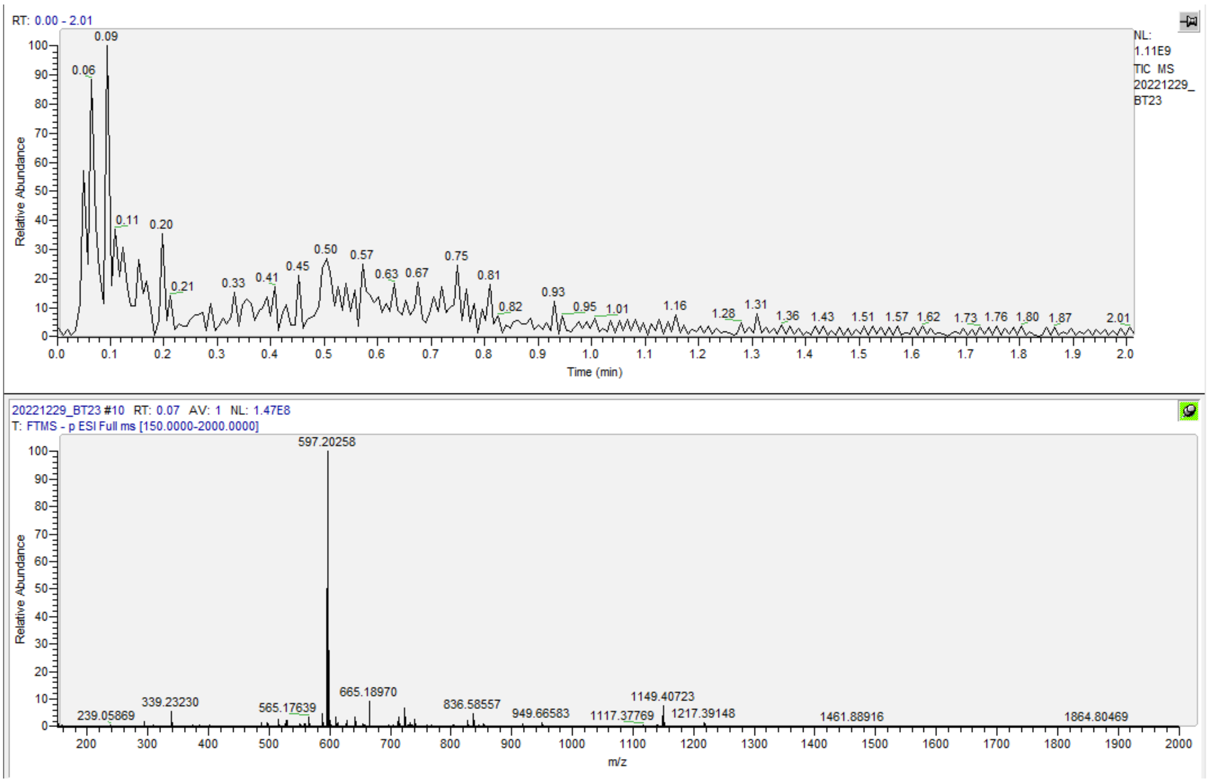

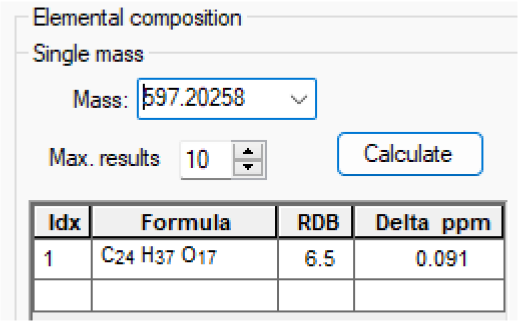

Idx	Formula	RDB	Delta ppm
1	$C_{24}H_{37}O_{17}$	6.5	0.091

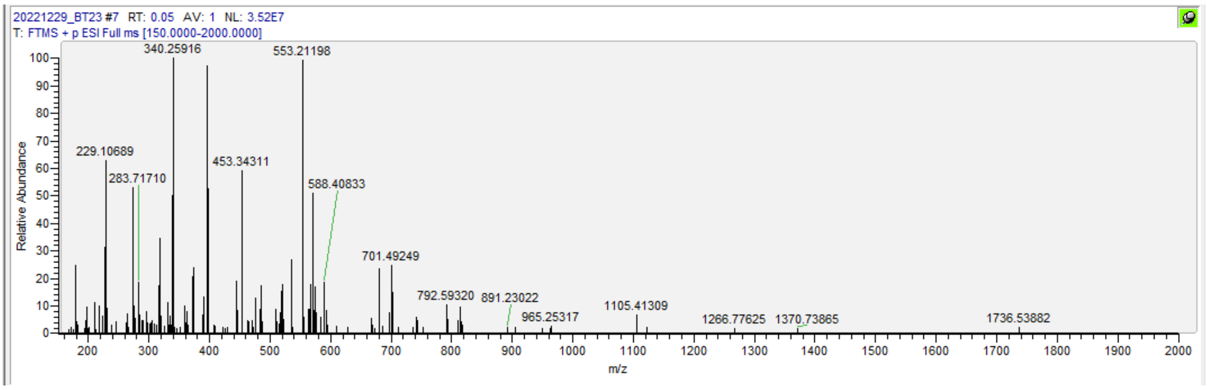

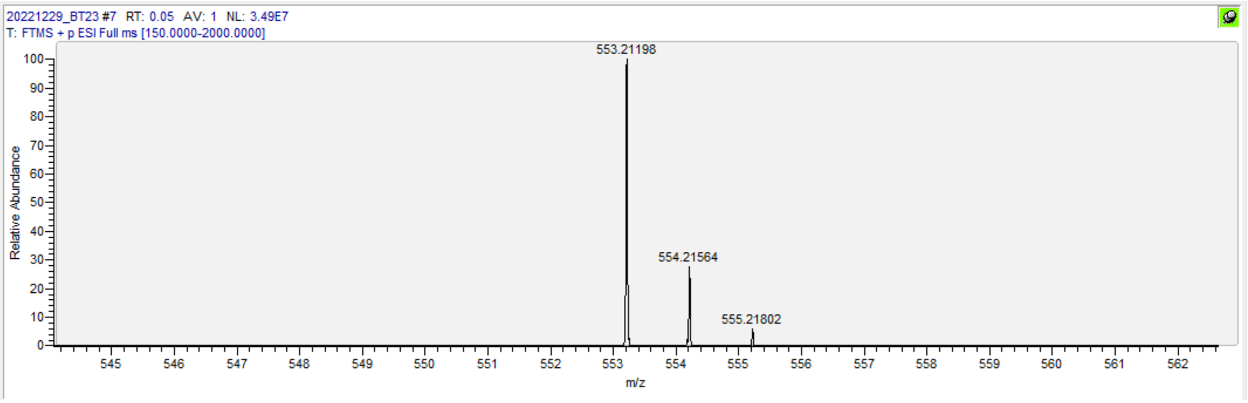

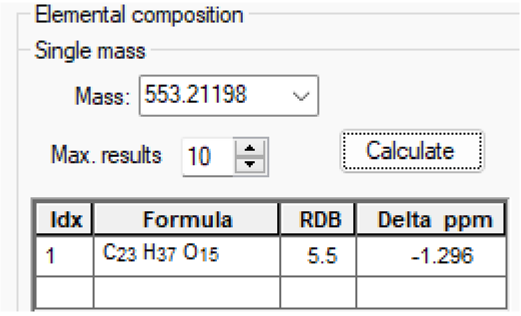

Idx	Formula	RDB	Delta ppm
1	$C_{23}H_{37}O_{15}$	5.5	-1.296

Figure S9. UV spectrum (in MeOH) of compound **1**

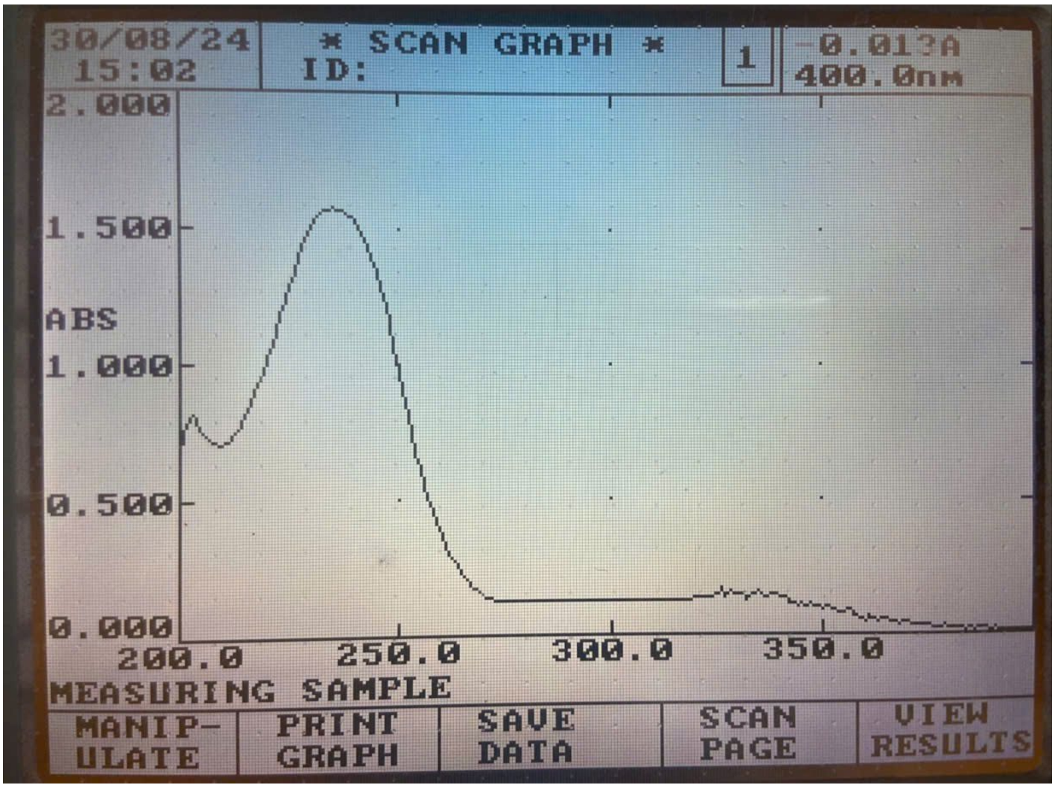

Figure S10. IR (ATR) spectrum of compound **1**

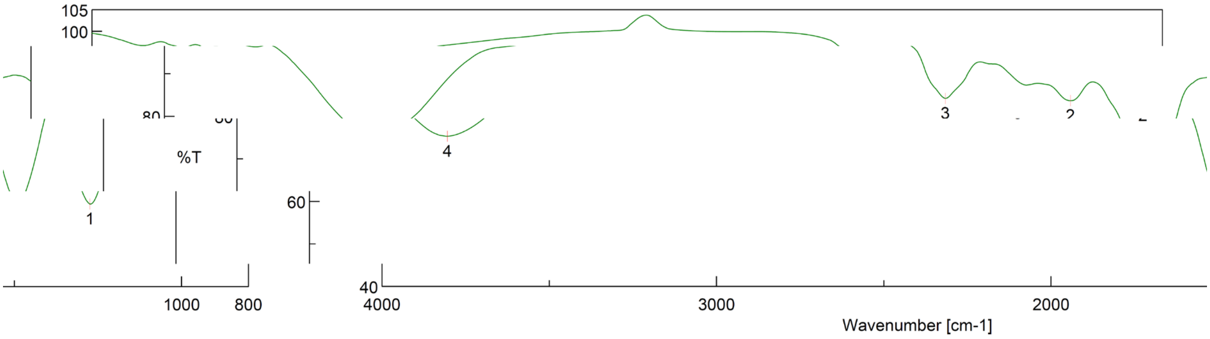

[Result of Peak Picking]

No.	Position	Intensity	No.	Position	Intensity	No.	Position	Intensity
1	1056.8	59.4014	2	1292.07	83.6159	3	1666.2	84.176
4	3370.96	75.2843						

Figure S11. ^{1}H-NMR spectrum of compound **2** (600 MHz, CD_3OD)

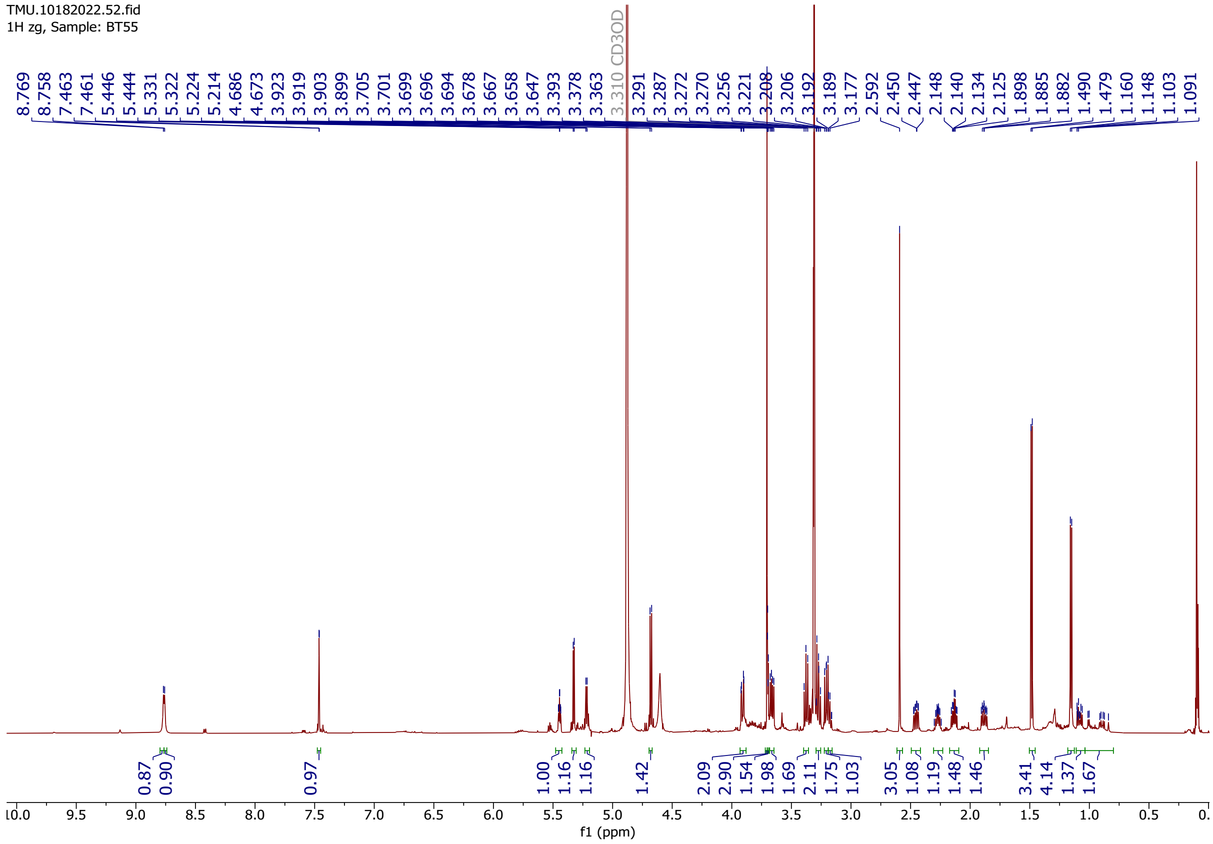

Figure S12. ^{13}C-NMR spectrum of compound **2** (150 MHz, CD_3OD)

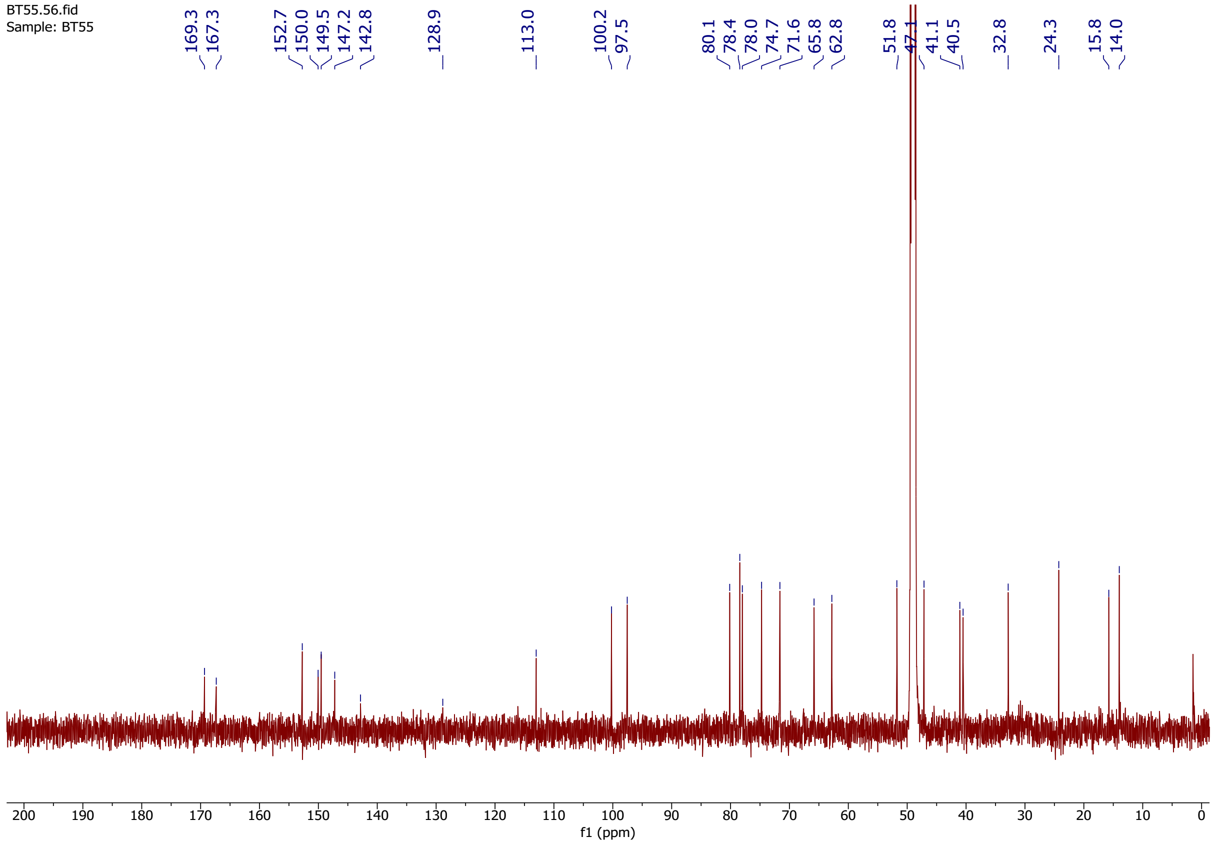

Figure S13. DEPT-NMR spectrum of compound **2** (150 MHz, CD_3OD)

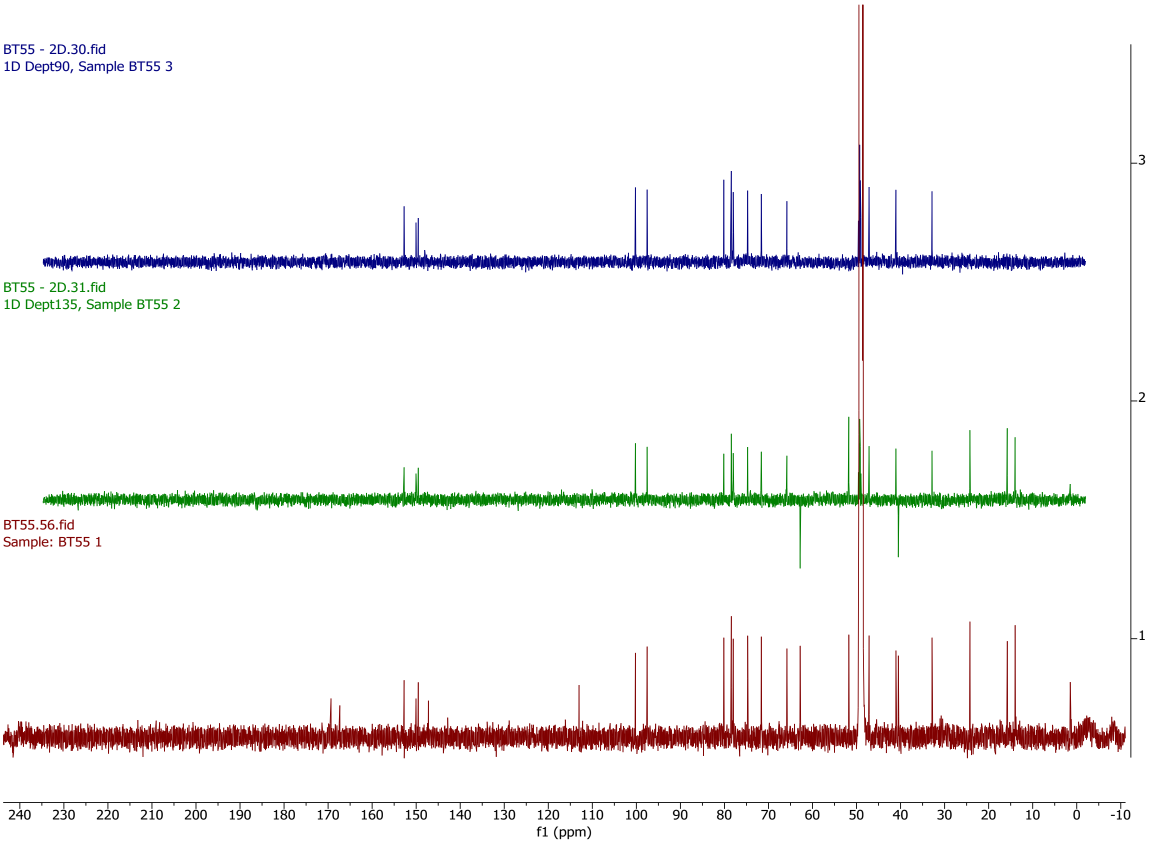

Figure S14. HSQC spectrum of compound **2** (600 MHz, CD_3OD)

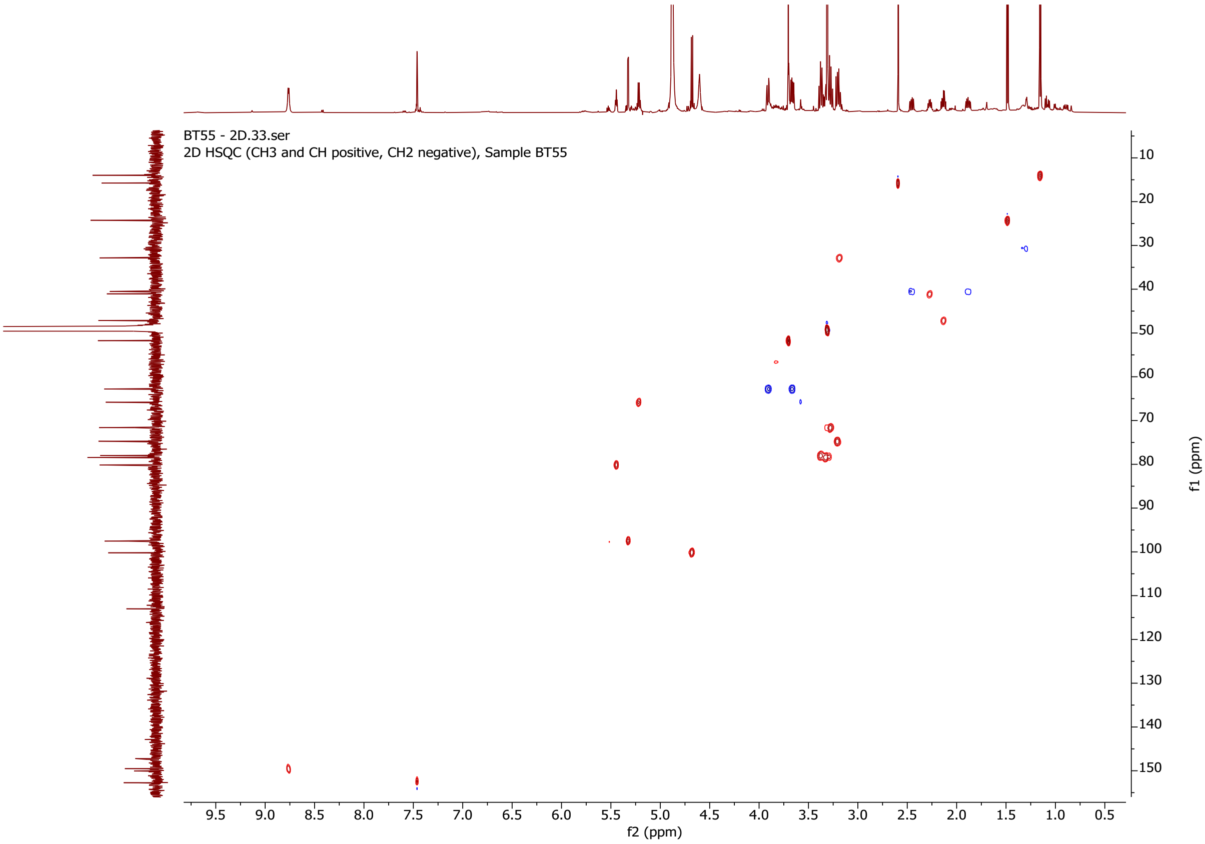

Figure S15. COSY spectrum of compound **2** (600 MHz, CD_3OD)

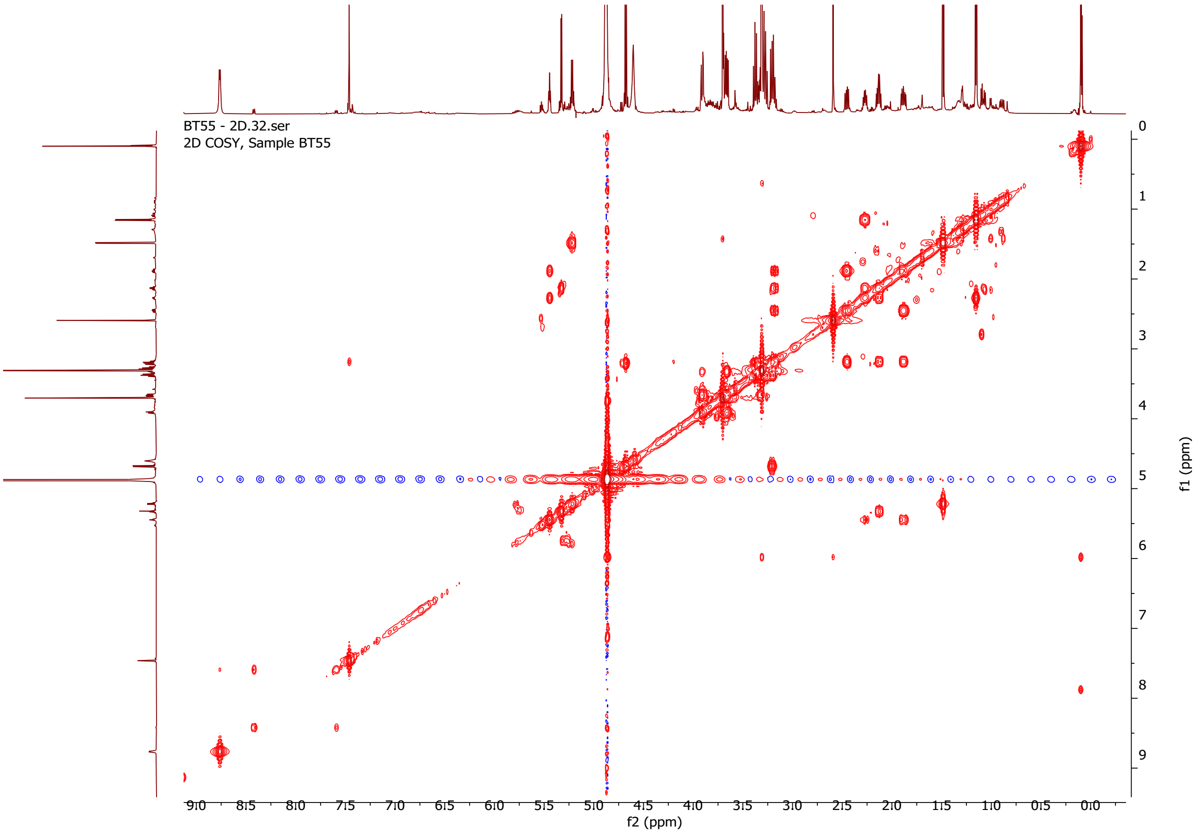

Figure S16. HMBC spectrum of compound **2** (600 MHz, CD_3OD)

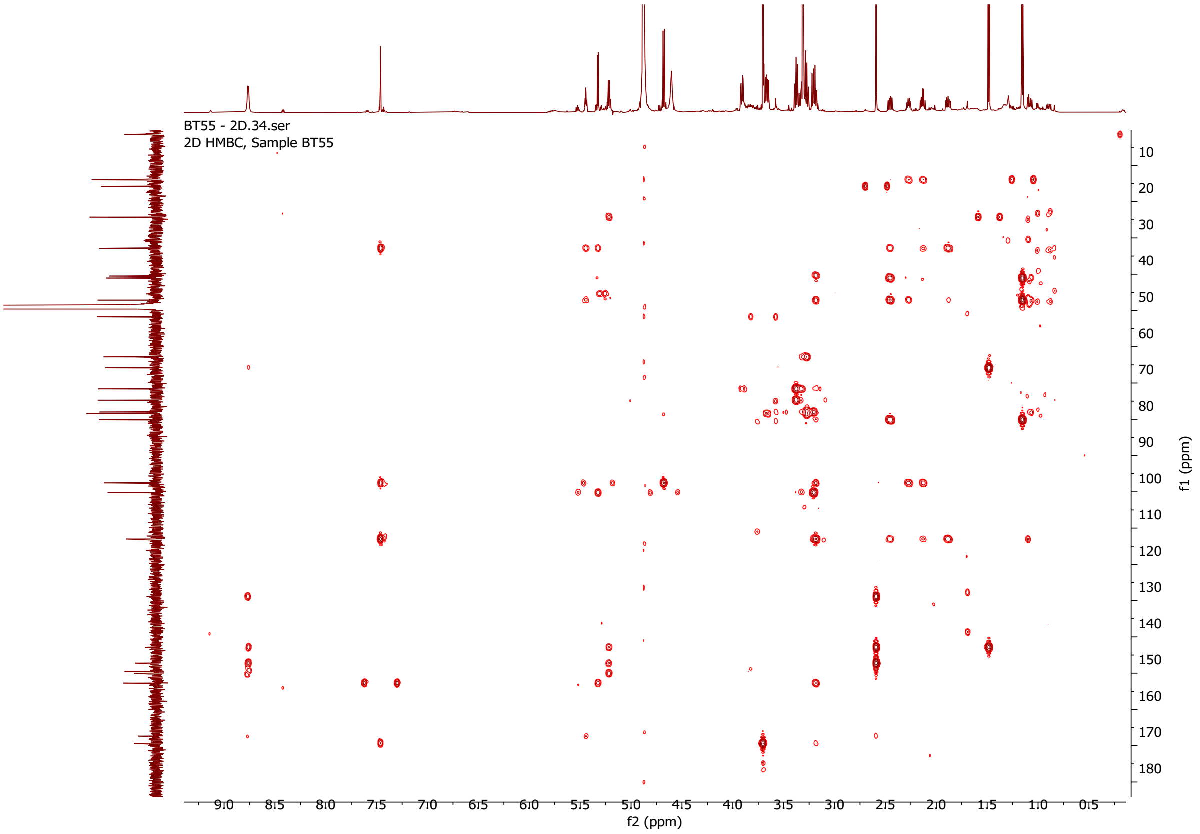

Figure S17. ROESY spectrum of compound **2** (600 MHz, CD_3OD)

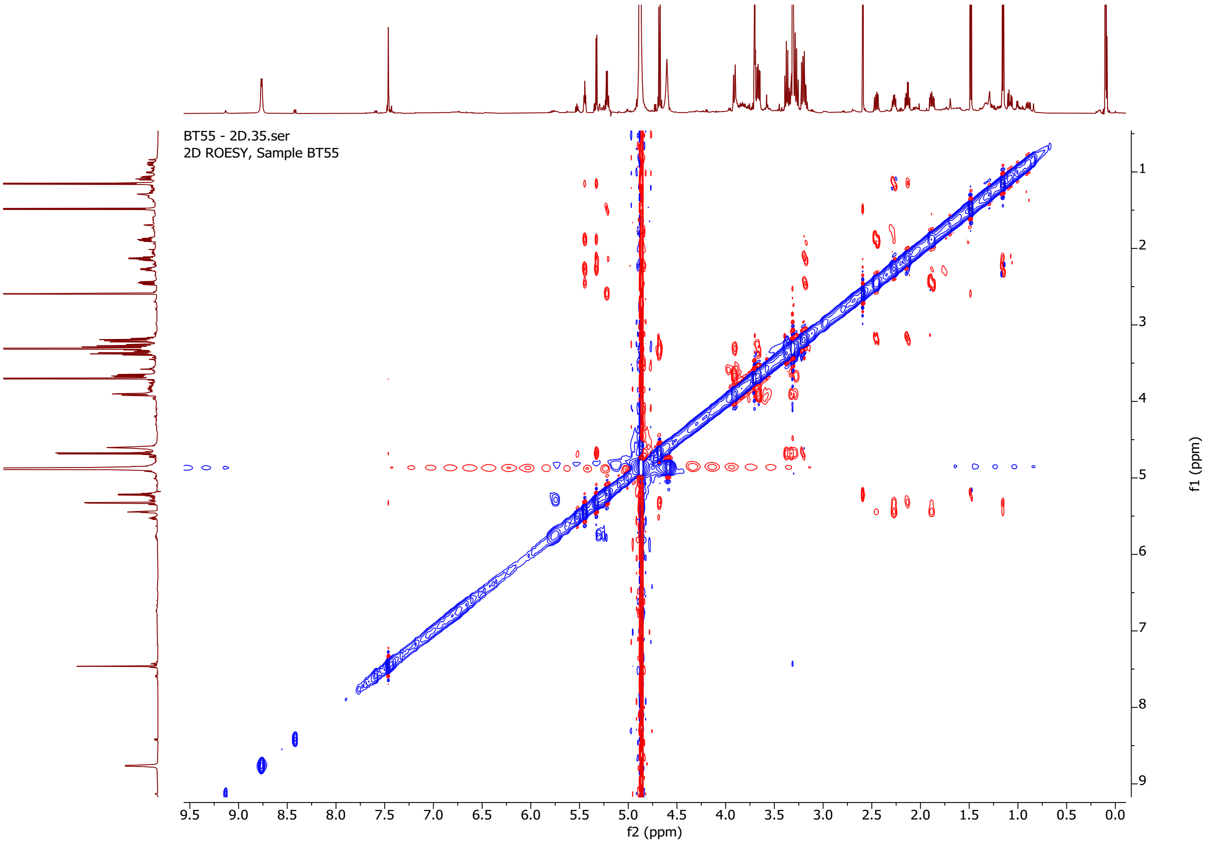

Figure S18. HR-ESI-MS data of compound **2**

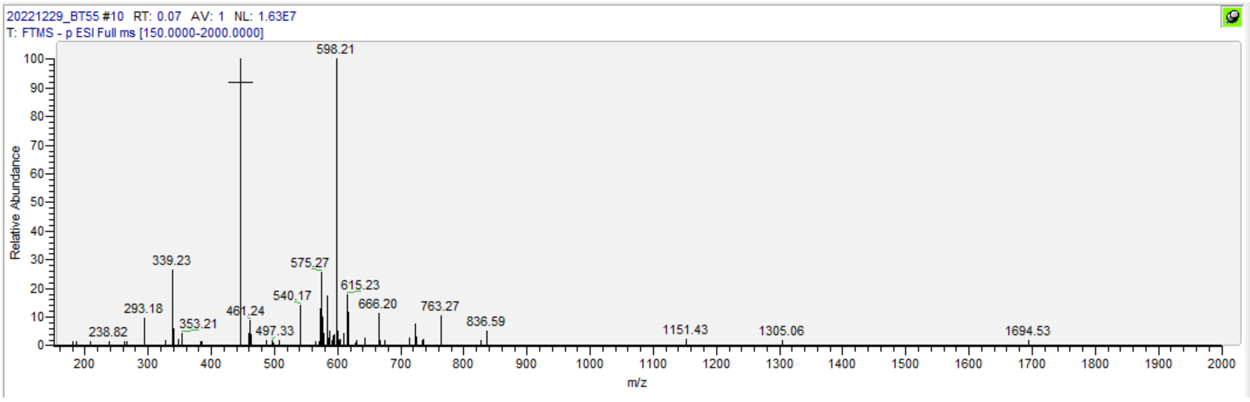

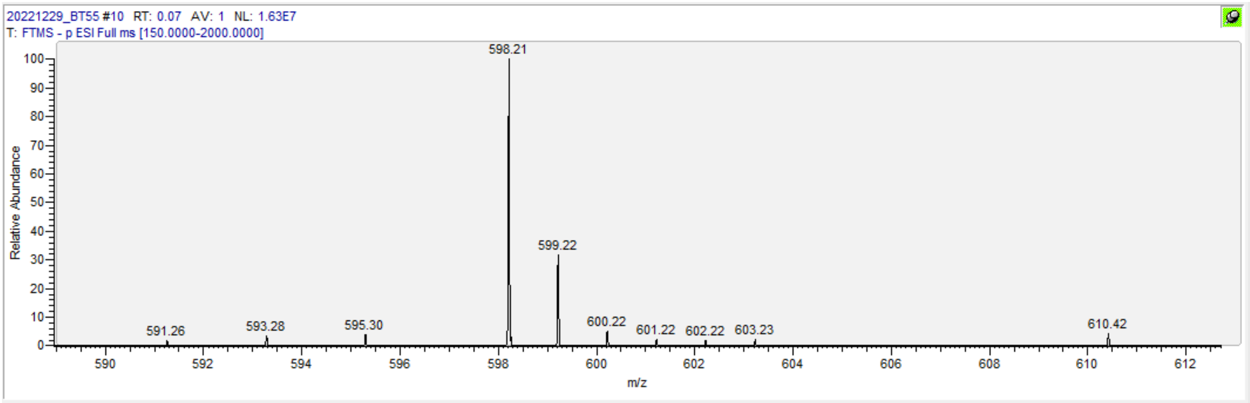

Single mass

Mass: 598.21350

Max. results 10 Calculate

Idx	Formula	RDB	Delta ppm
1	$C_{27}H_{36}O_{14}N$	10.5	0.784

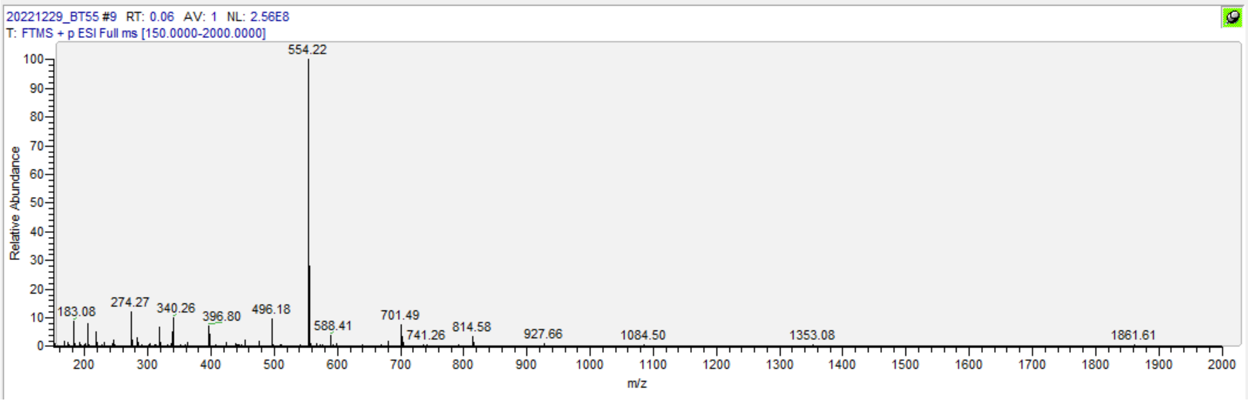

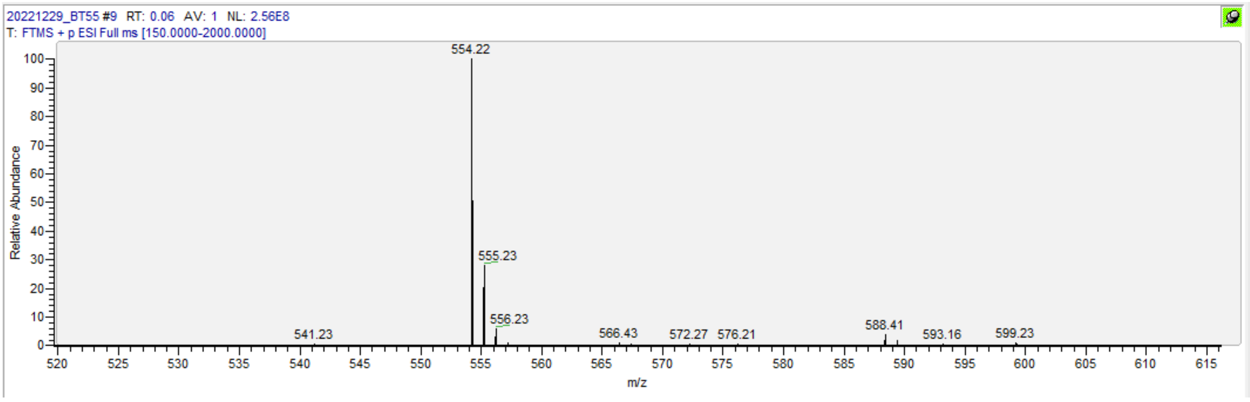

Elemental composition

Single mass

Mass: 554.22278

Max. results 10 Calculate

Idx	Formula	RDB	Delta ppm
1	$C_{26}H_{36}O_{12}N$	9.5	-0.761

Figure S19. UV spectrum (in MeOH) of compound **2**

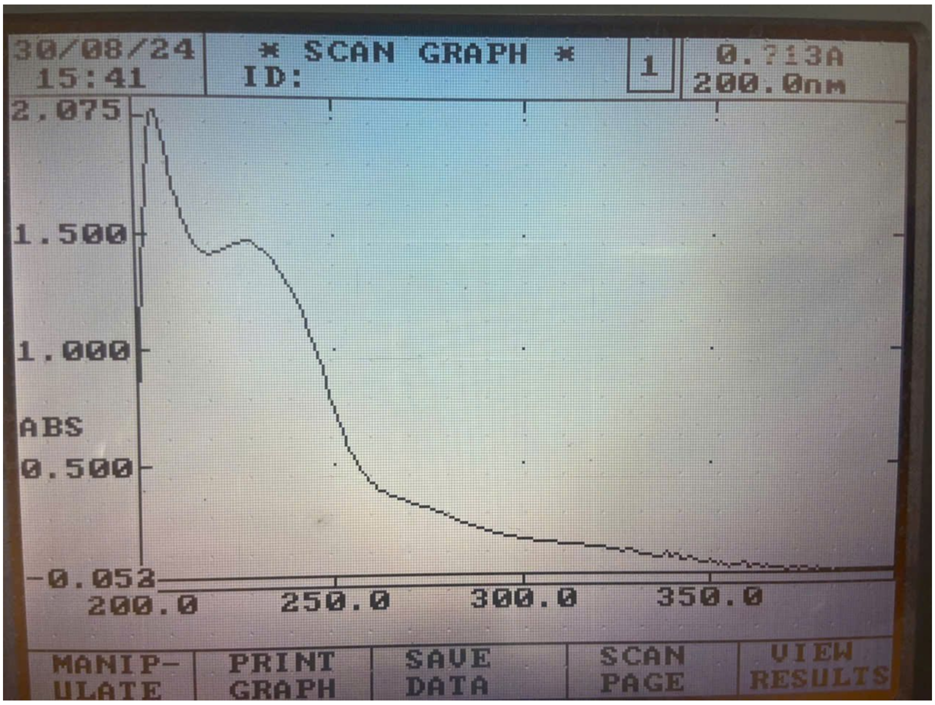

Figure S20. IR (ATR) spectrum of compound **2**

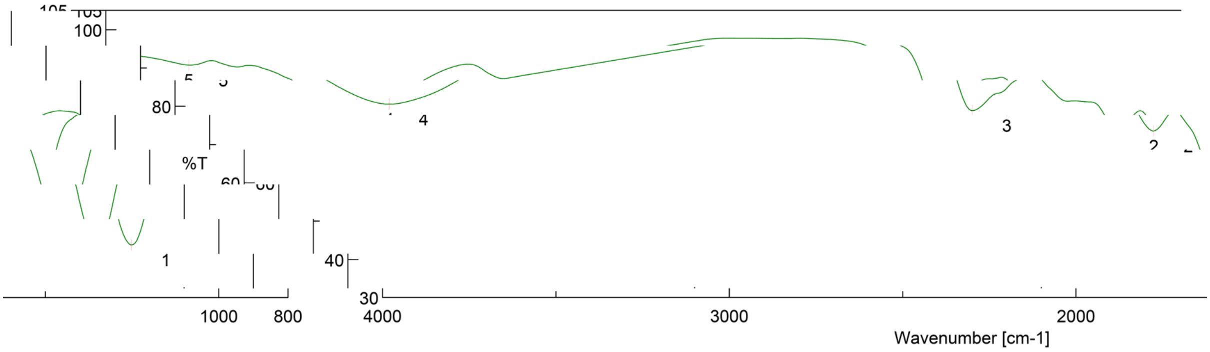

[Result of Peak Picking]

No.	Position	Intensity	No.	Position	Intensity	No.	Position	Intensity
1	1052.94	43.7718	2	1276.65	73.4753	3	1700.91	78.8069
4	3382.53	80.4887	5	3860.79	90.6817			

Figure S21. ^{1}H-NMR spectrum of compound **3** (600 MHz, CD_3OD)

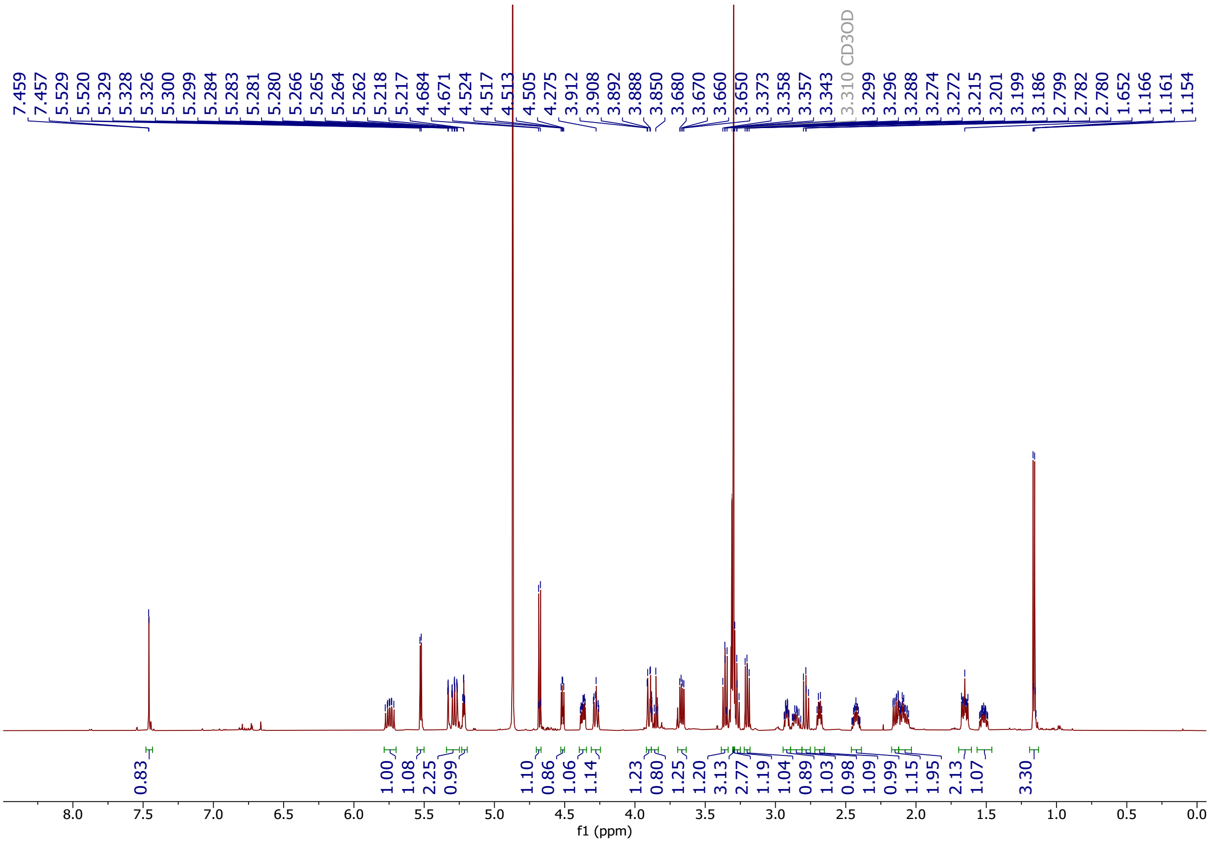

Figure S22. ^{13}C-NMR spectrum of compound **3** (150 MHz, CD_3OD)

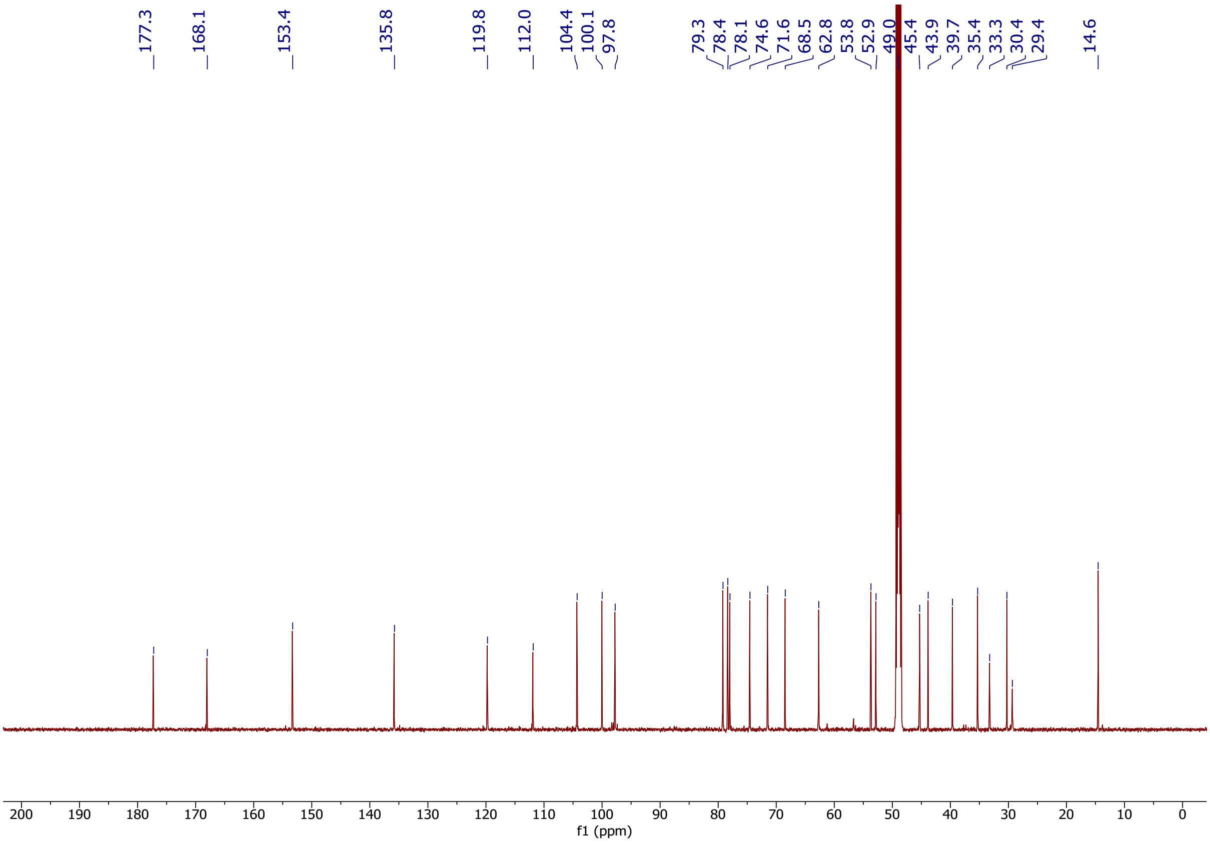

Figure S23. DEPT-NMR spectrum of compound **3** (150 MHz, CD_3OD)

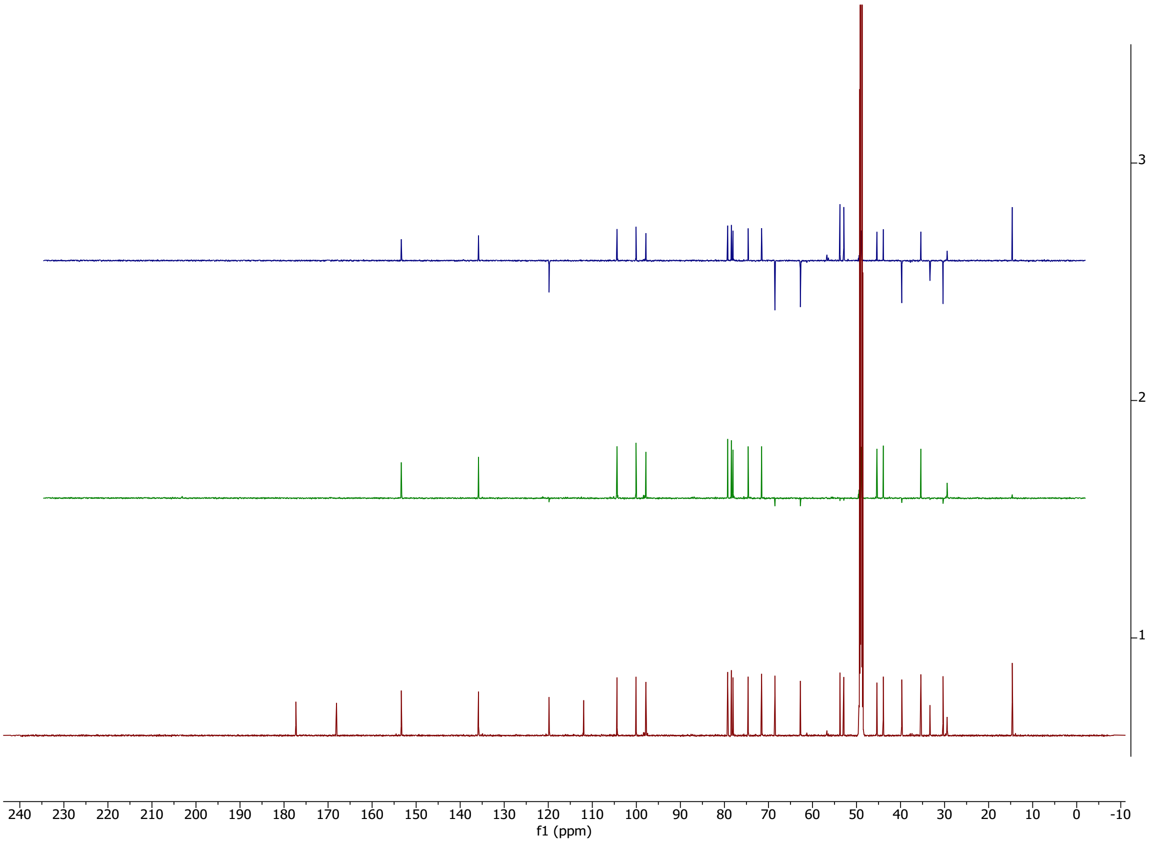

Figure S24. HSQC spectrum of compound **3** (600 MHz, CD_3OD)

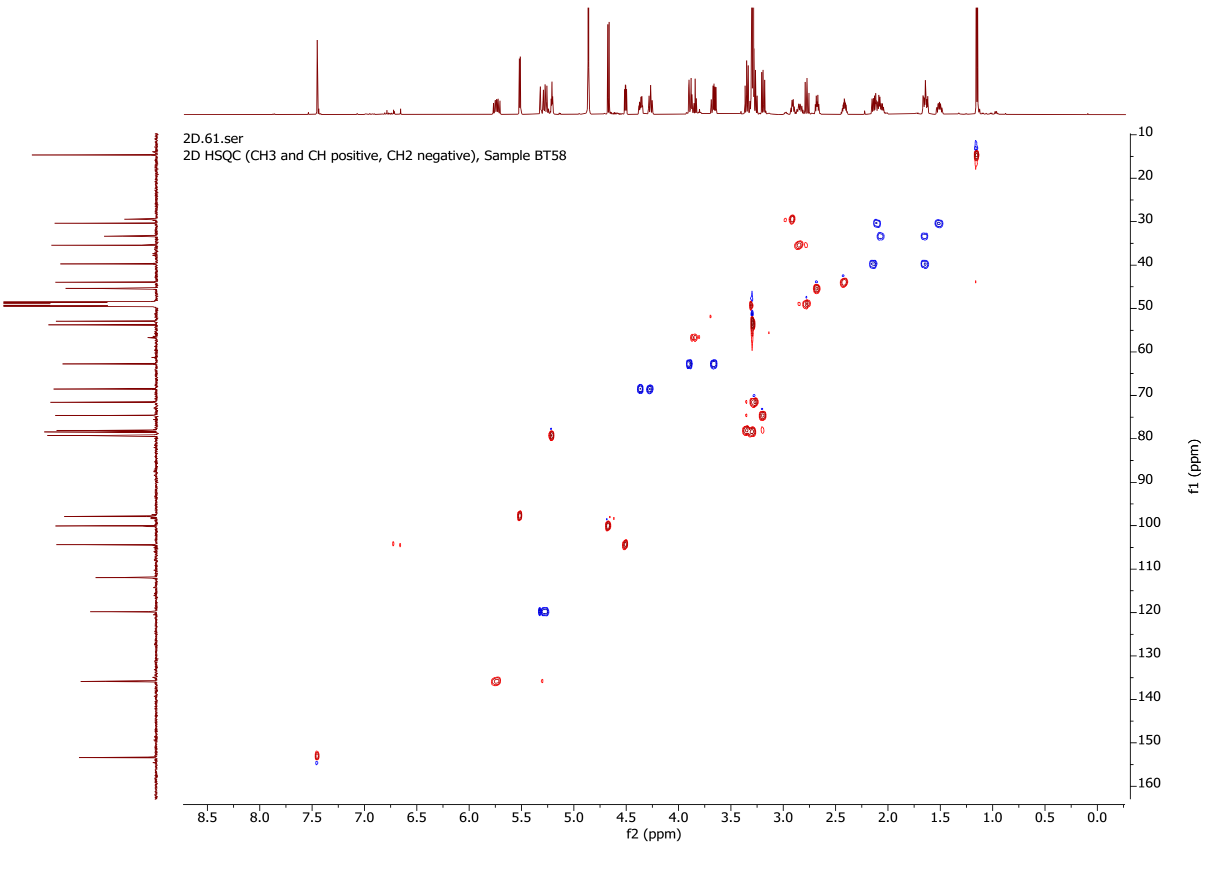

Figure S25. COSY spectrum of compound **3** (600 MHz, CD_3OD)

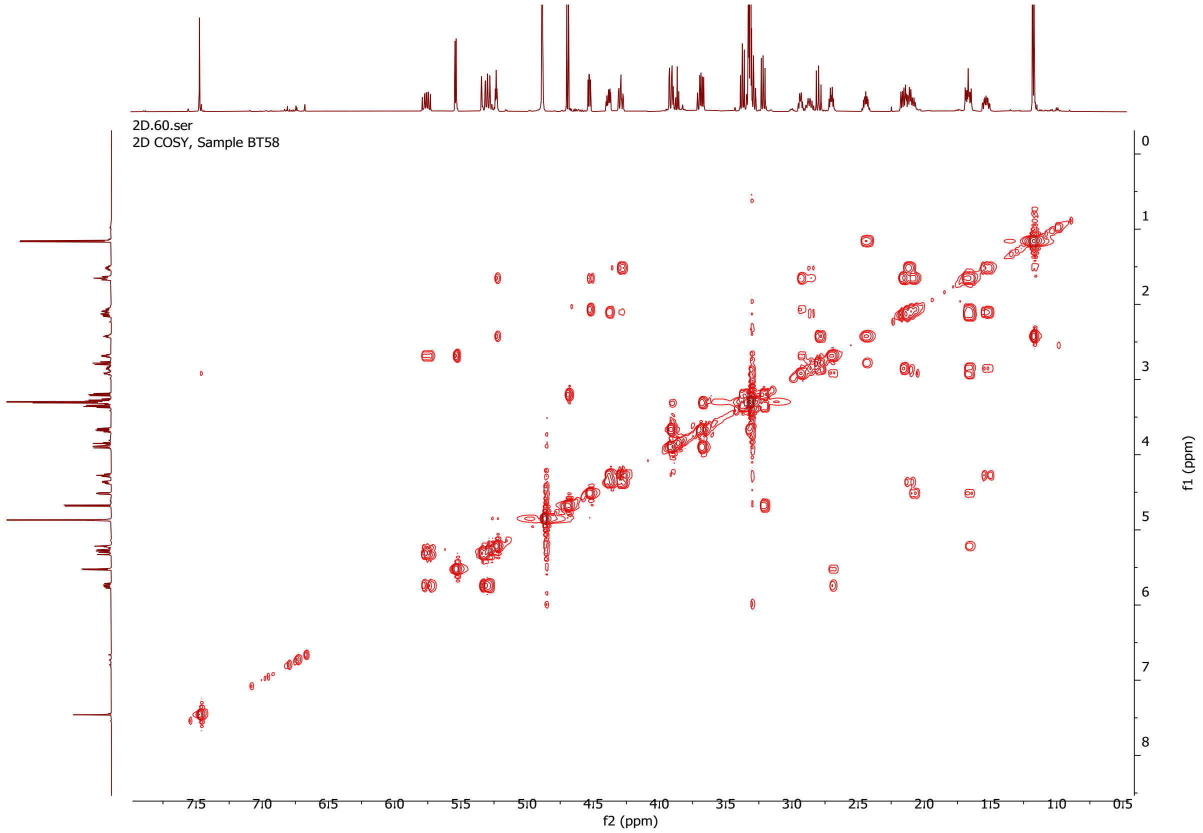

Figure S26. HMBC spectrum of compound **3** (600 MHz, CD_3OD)

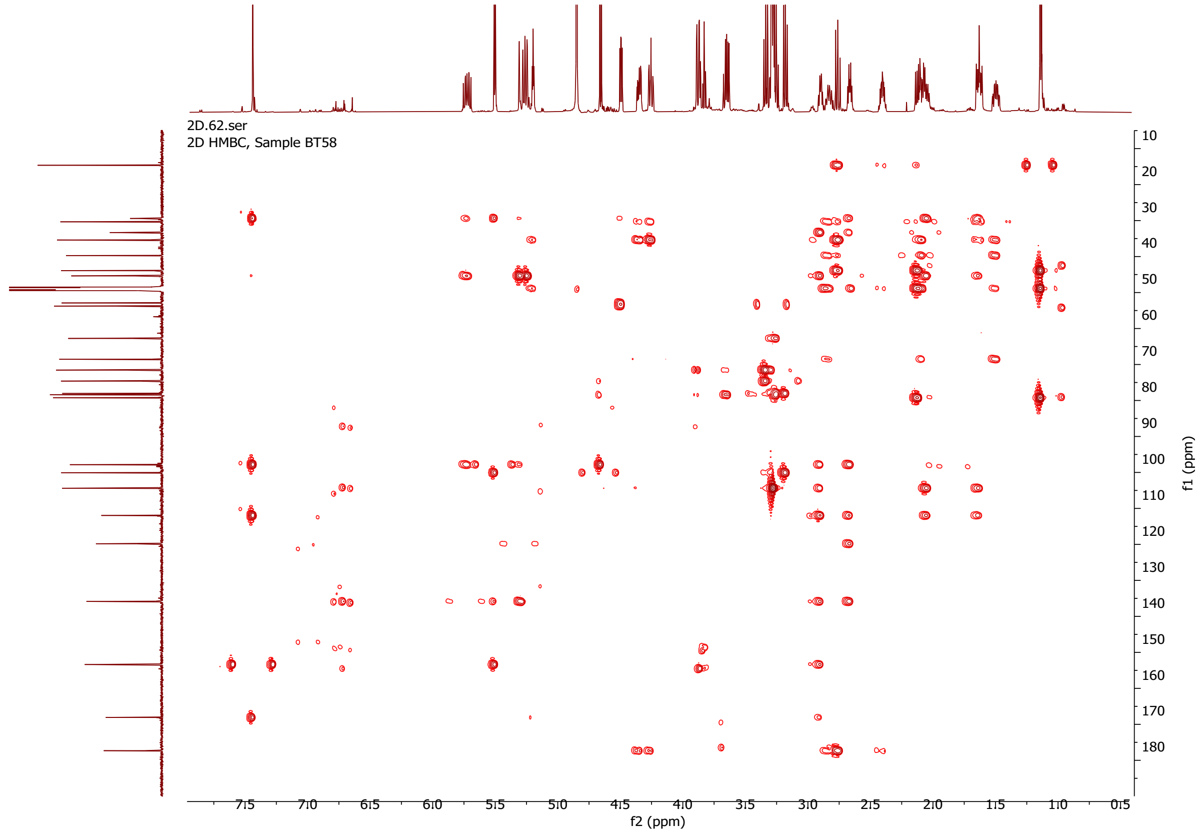

Figure S27. ROESY spectrum of compound **3** (600 MHz, CD_3OD)

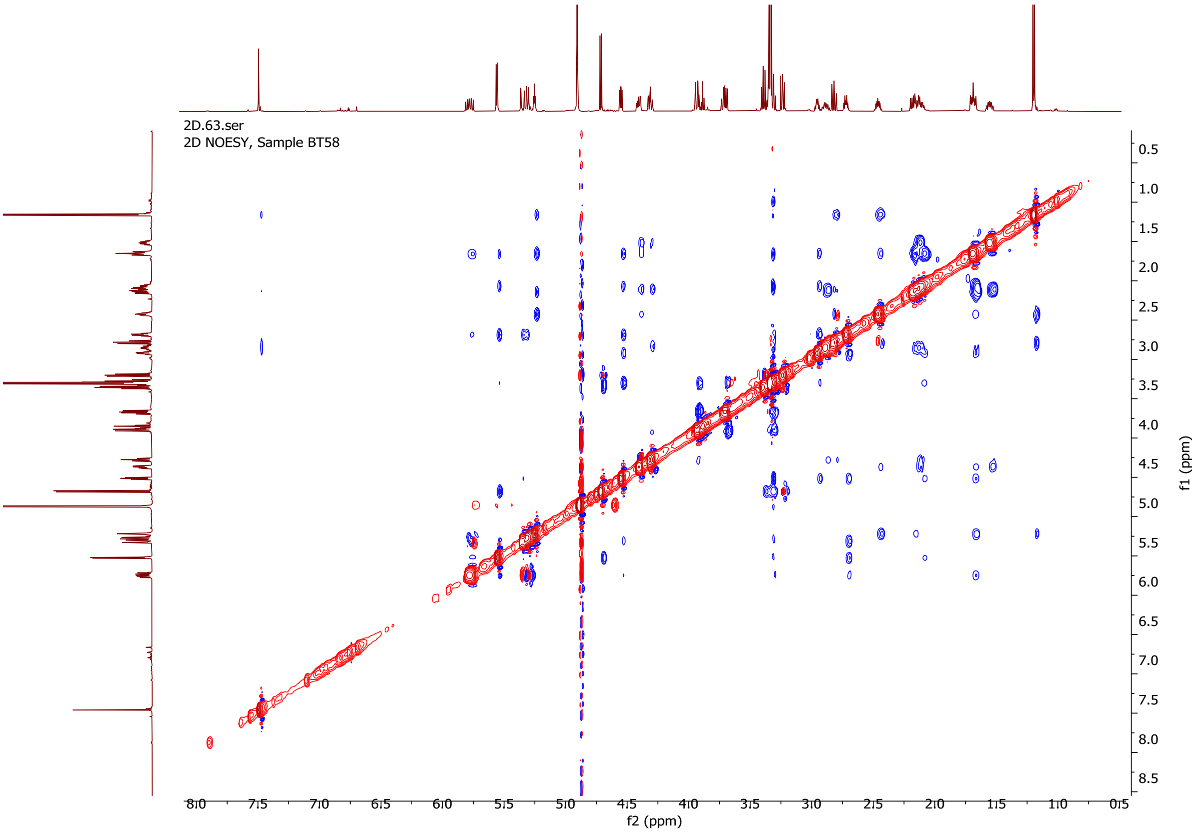

Figure S28. HR-ESI-MS data of compound **3**

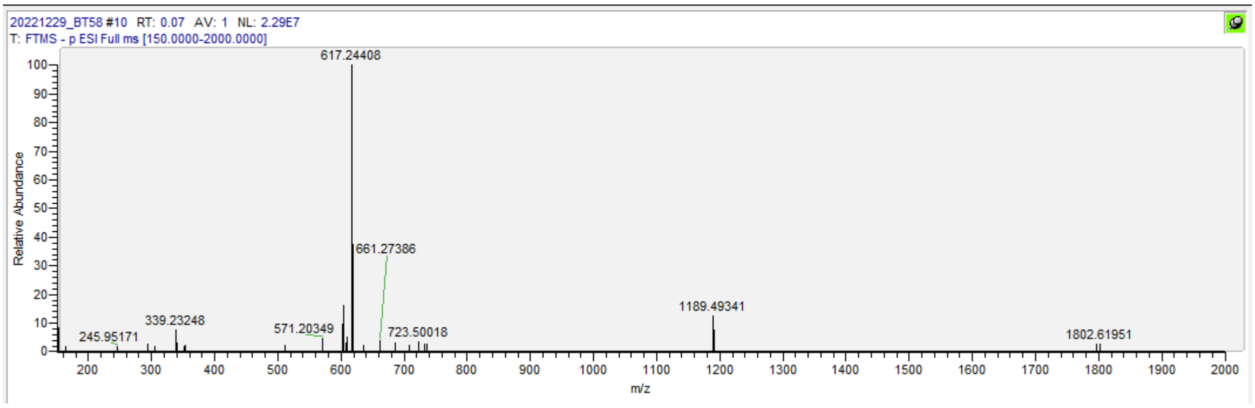

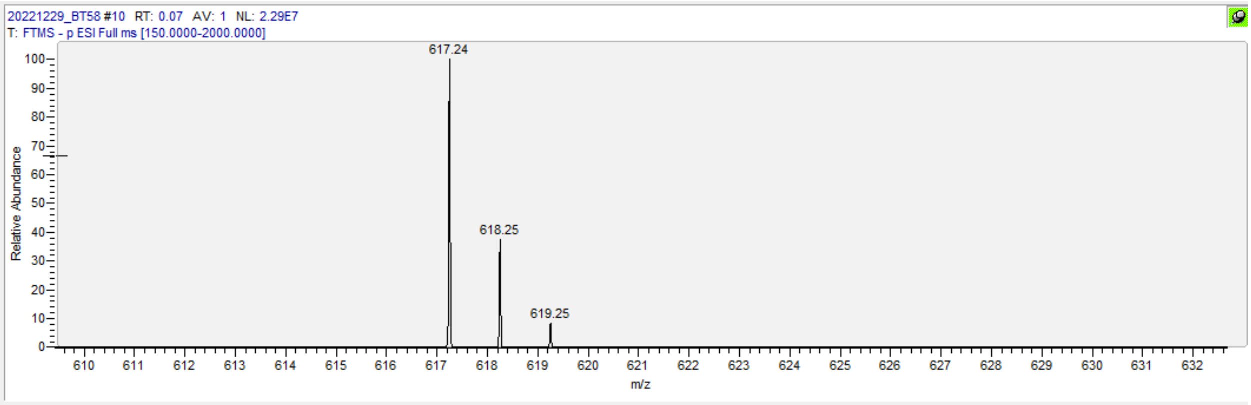

Elemental composition

Single mass

Mass: 617.24408

Max. results 10 Calculate

Idx	Formula	RDB	Delta ppm
1	$C_{28}H_{41}O_{15}$	8.5	0.135

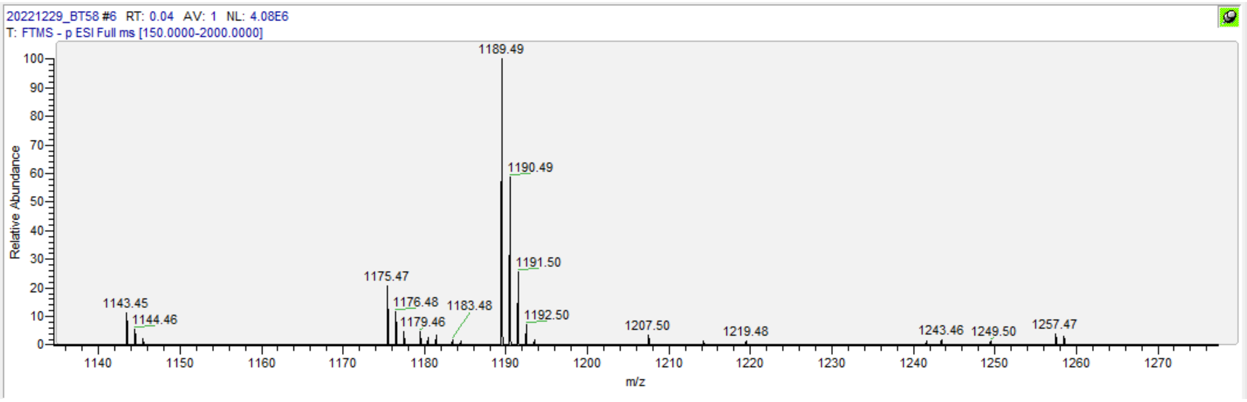

Elemental composition

Single mass

Mass: 1189.48987

Max. results 10 Calculate

Idx	Formula	RDB	Delta ppm
1	$C_{55}H_{81}O_{28}$	15.5	-0.856

Figure S29. UV spectrum (in MeOH) of compound **3**

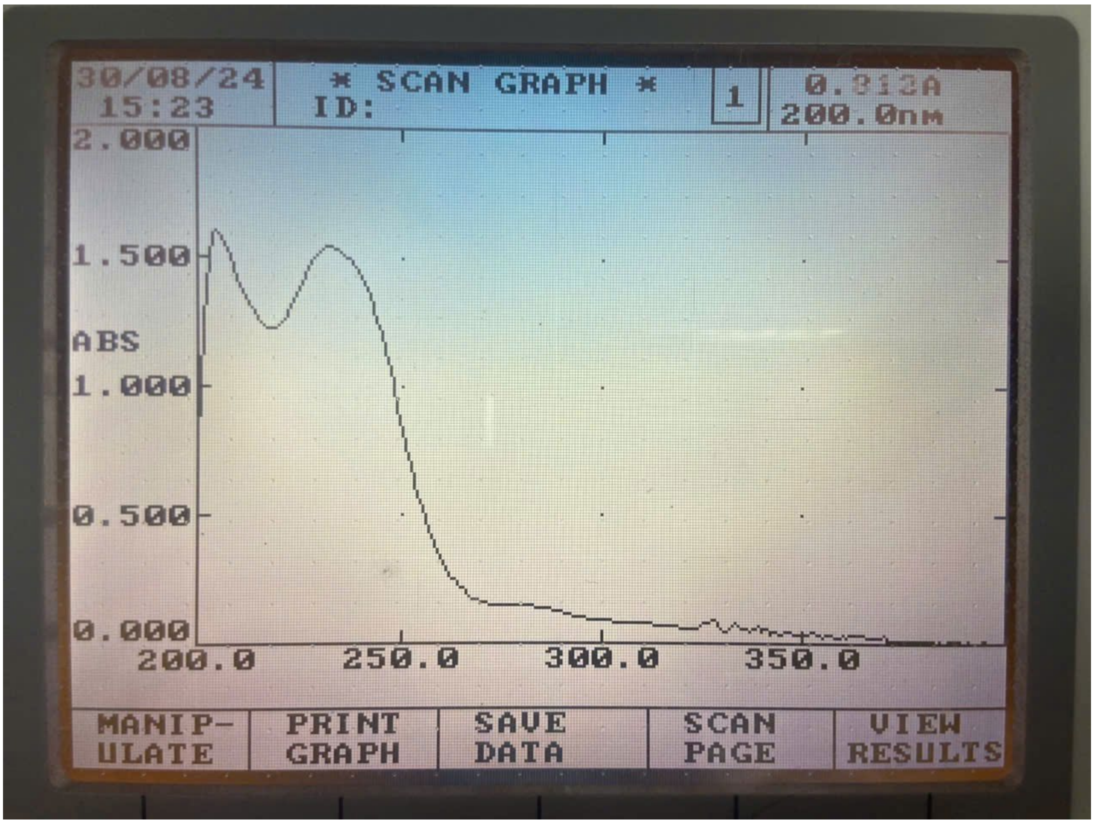

Figure S30. IR (ATR) spectrum of compound **3**

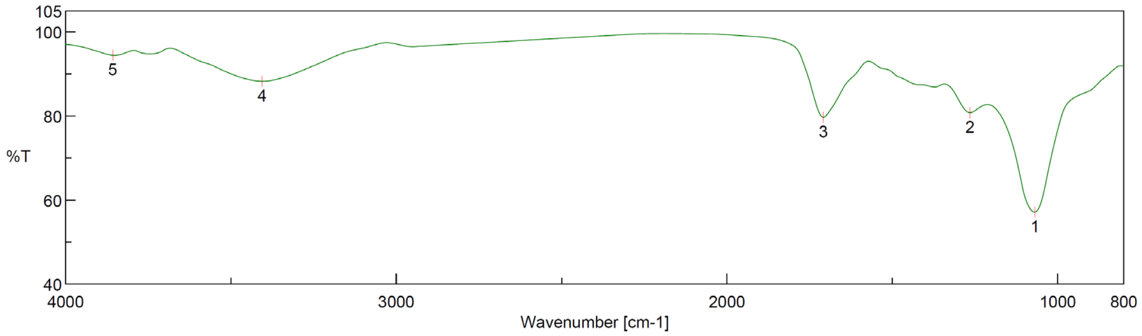

[Result of Peak Picking]

No.	Position	Intensity	No.	Position	Intensity	No.	Position	Intensity
1	1068.37	57.1121	2	1265.07	80.7537	3	1708.62	79.6302
4	3405.67	88.2207	5	3856.93	94.4132			

Figure S31. ^{1}H-NMR spectrum of compound **4** (600 MHz, CD_3OD)

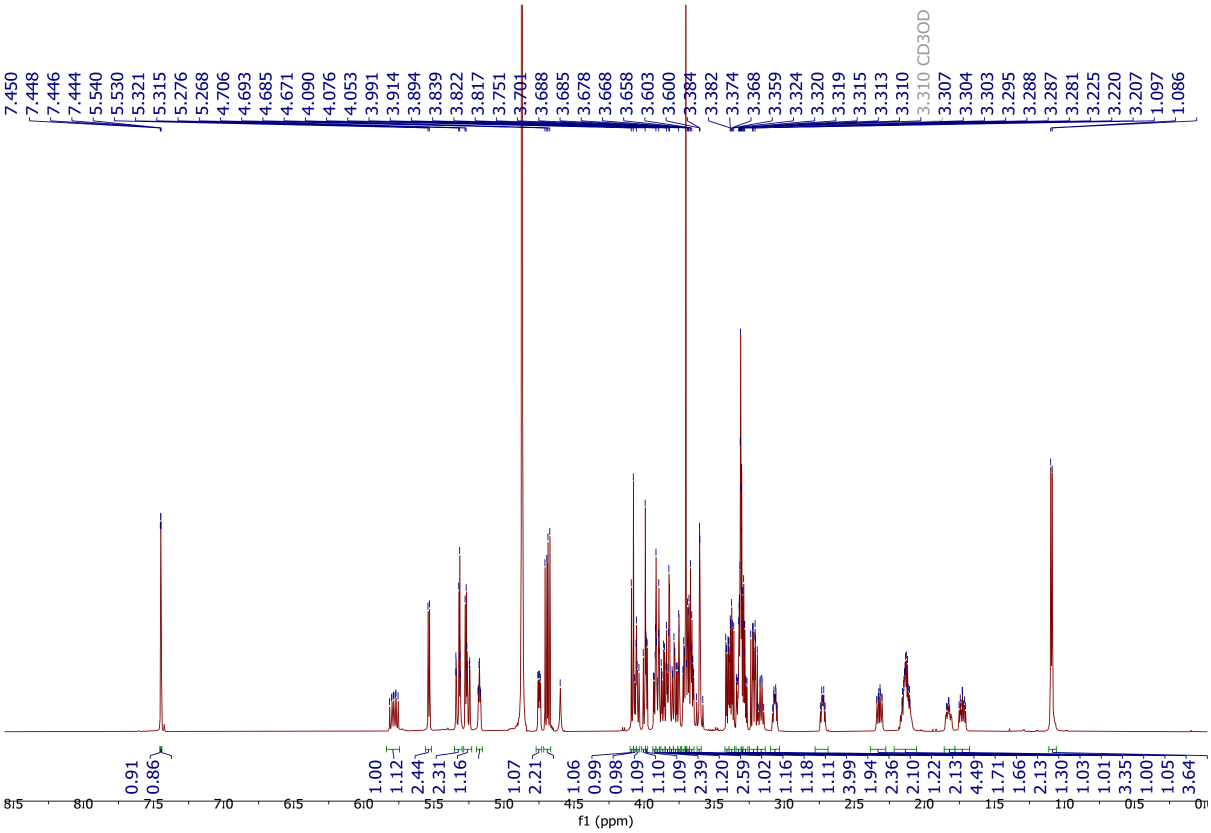

Figure S32. ^{13}C-NMR spectrum of compound **4** (125 MHz, CD_3OD)

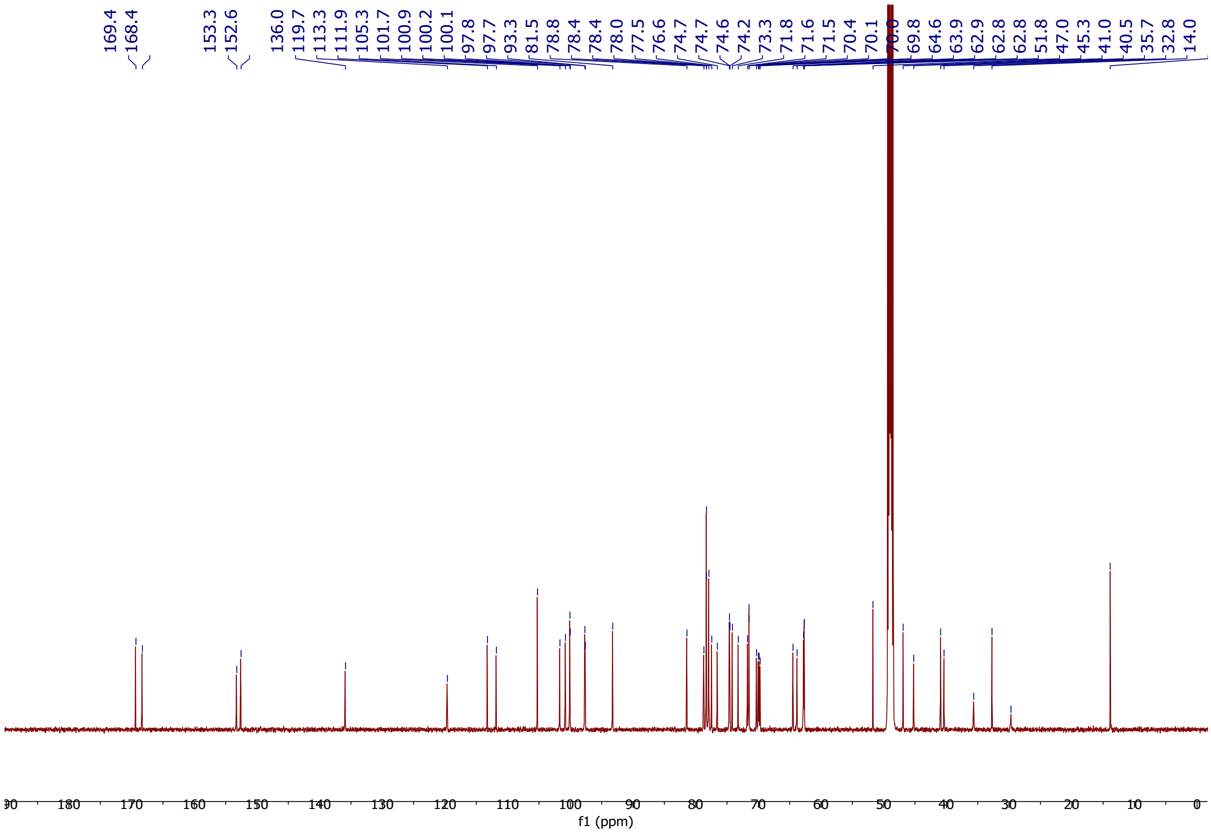

Figure S33. DEPT-NMR spectrum of compound **4** (125 MHz, CD_3OD)

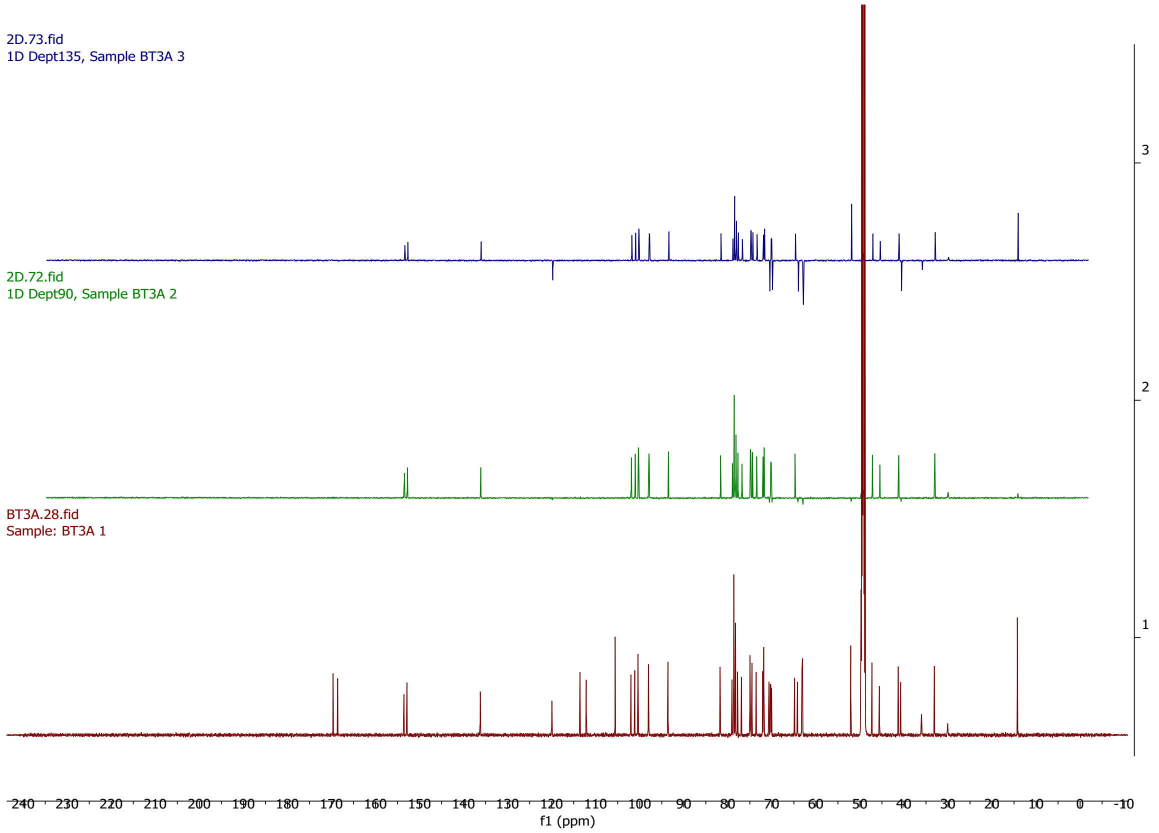

Figure S34. HSQC spectrum of compound **4** (600 MHz, CD_3OD)

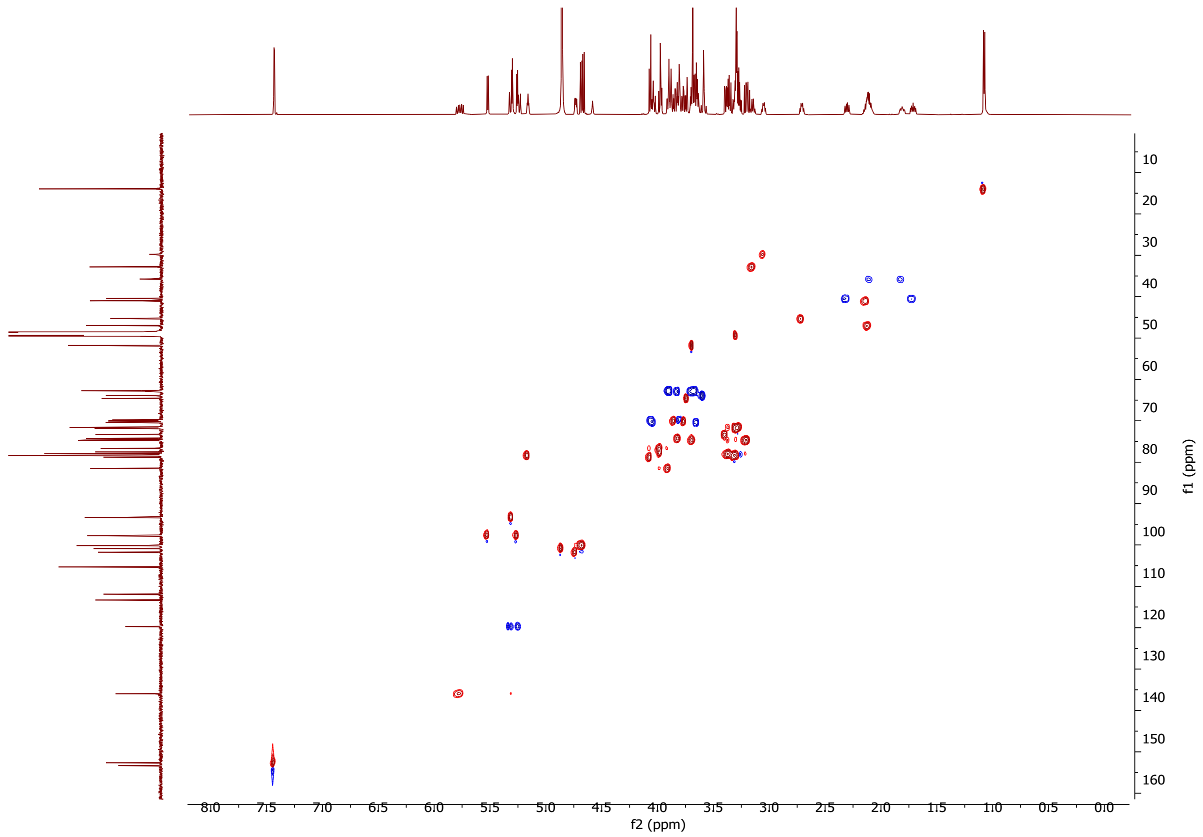

Figure S35. COSY spectrum of compound **4** (600 MHz, CD_3OD)

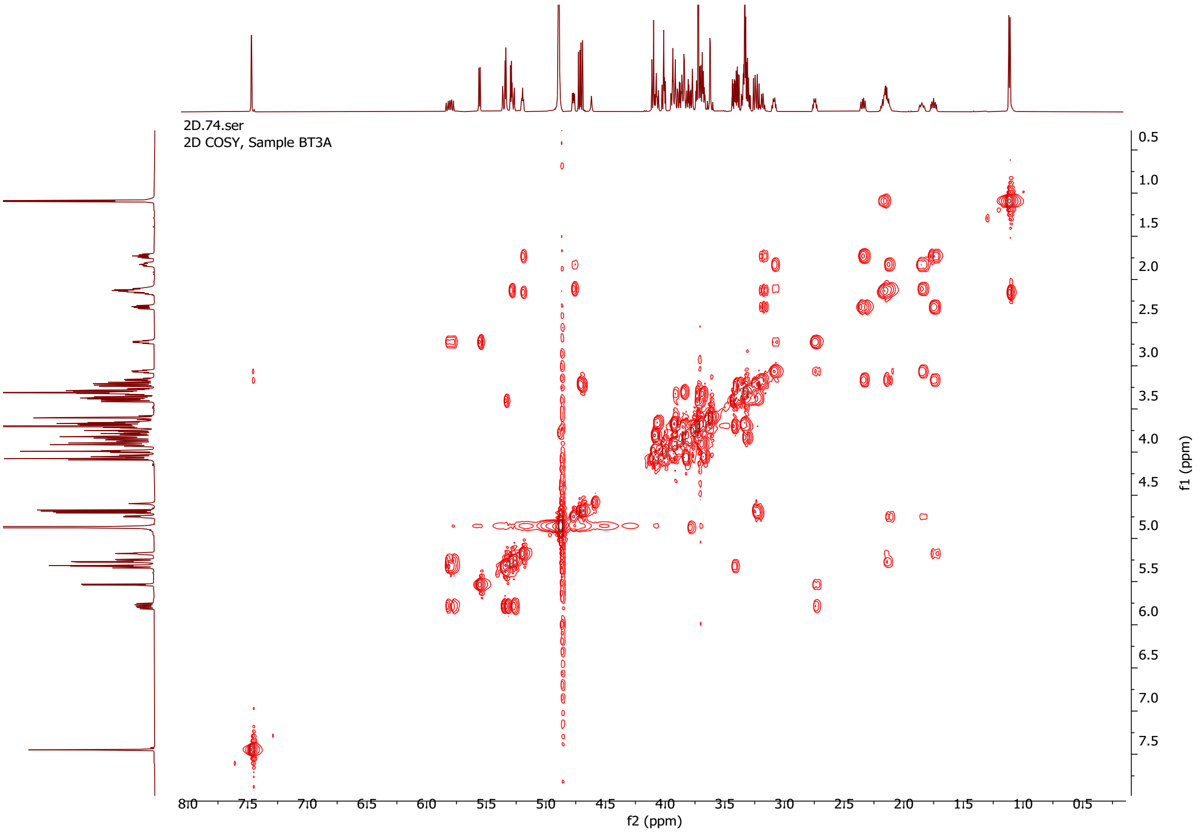

Figure S36. HMBC spectrum of compound **4** (600 MHz, CD_3OD)

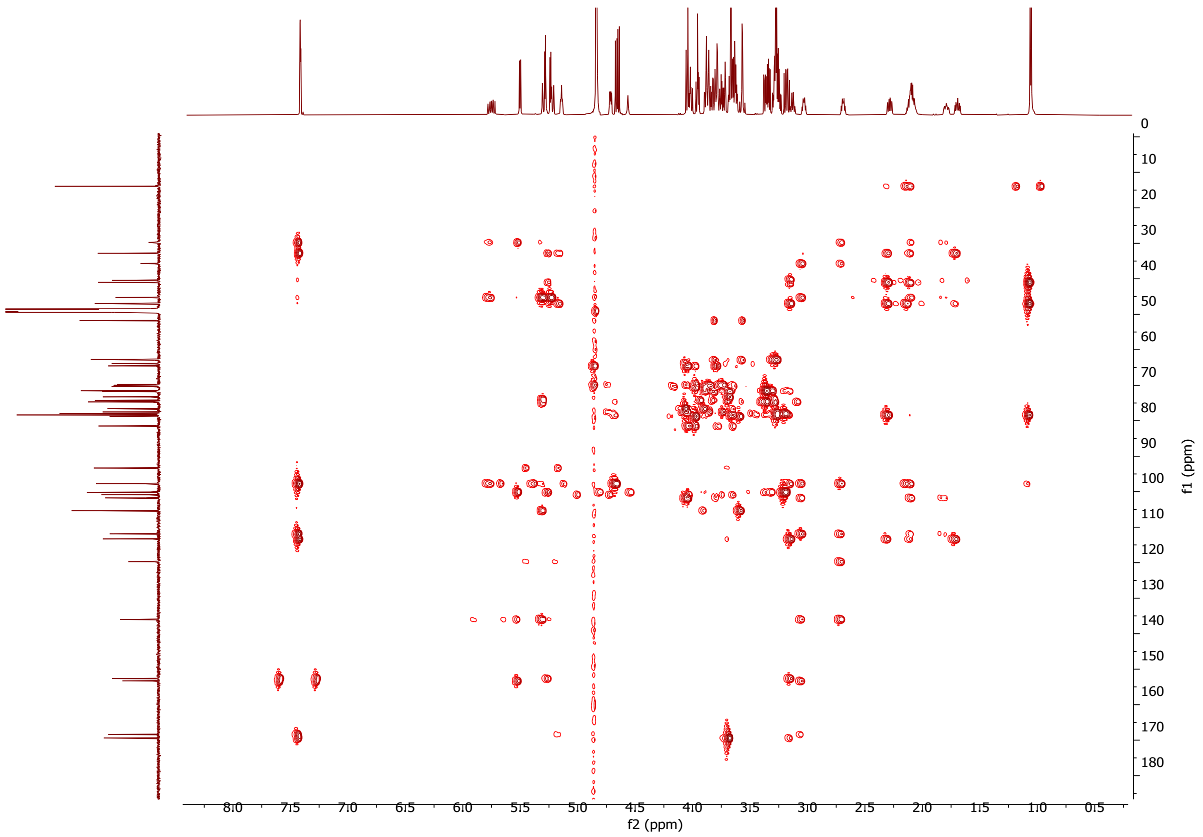

Figure S37. ROESY spectrum of compound **4** (600 MHz, CD_3OD)

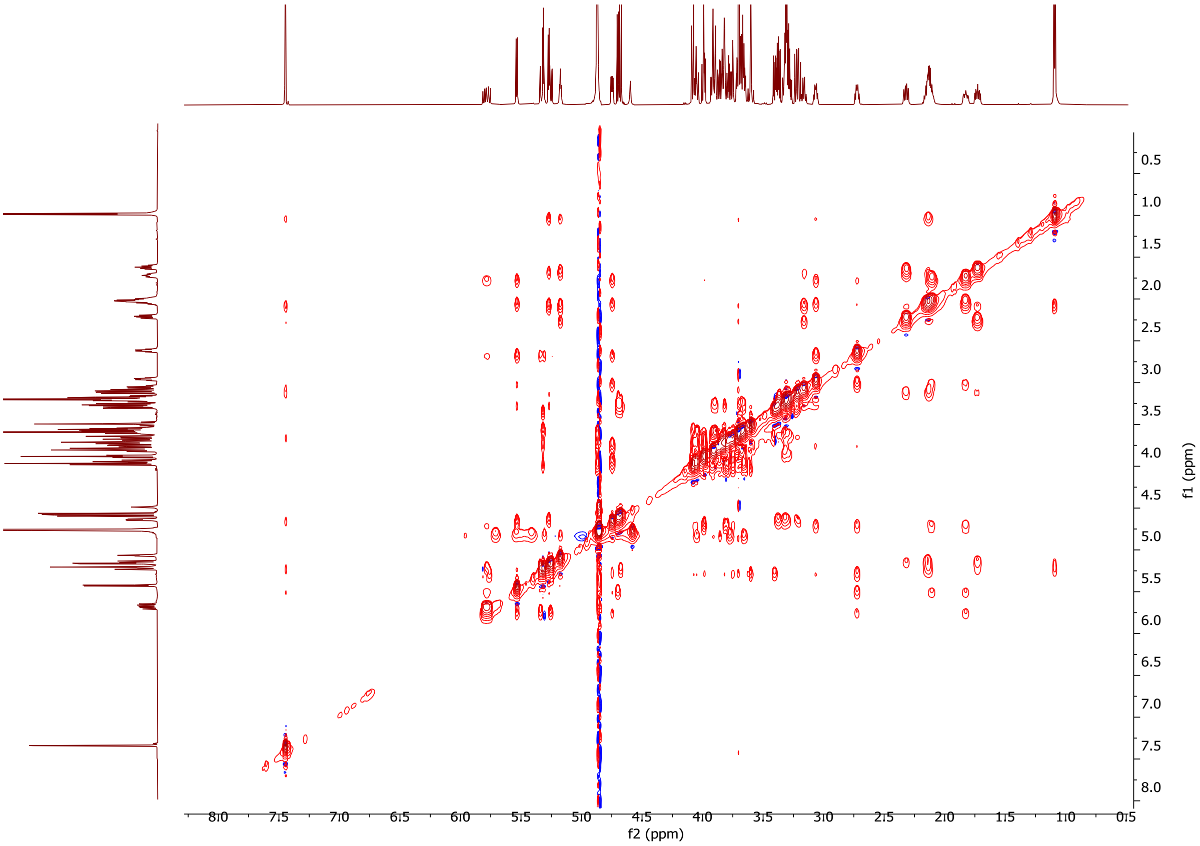

Figure S38. 2D-TOCSY spectrum of compound **4** (600 MHz, CD_3OD)

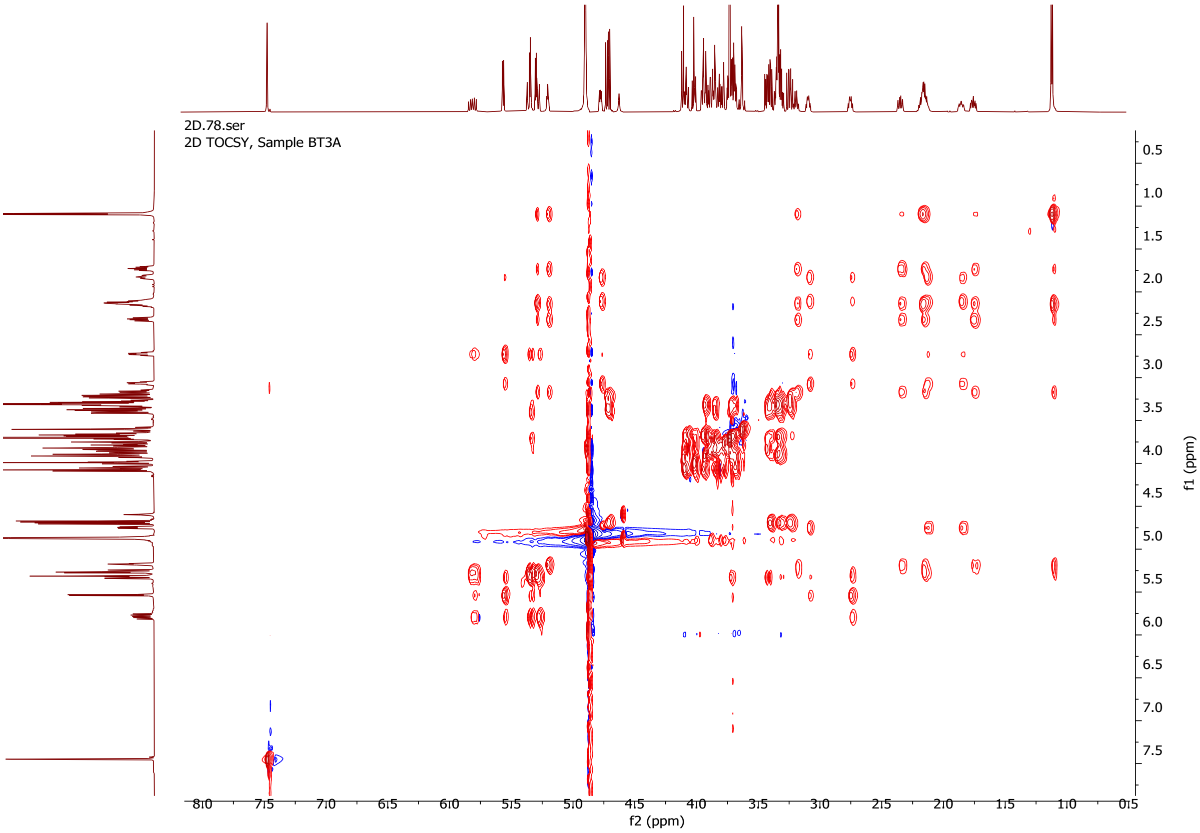

Figure S39. HR-ESI-MS data of compound **4**

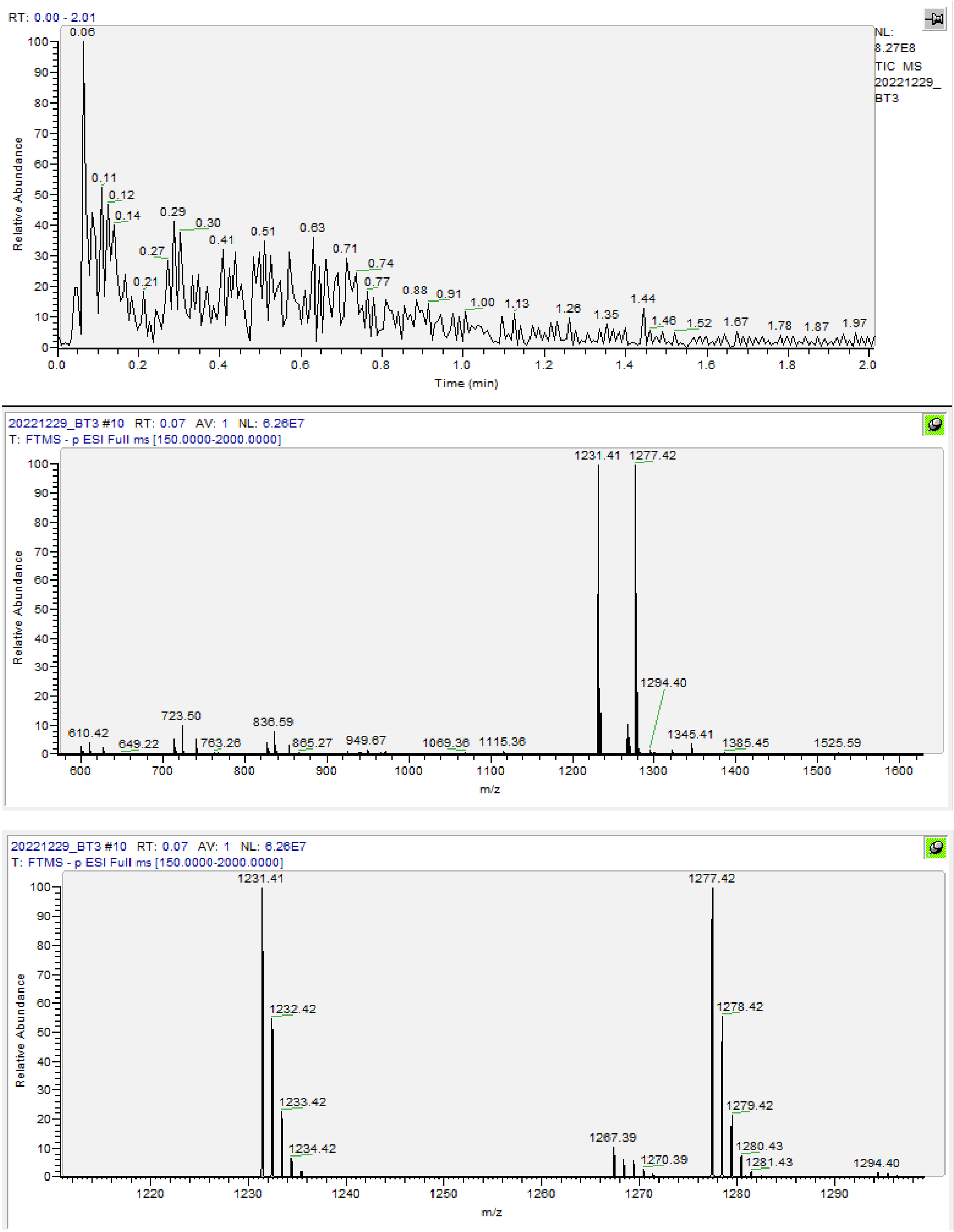

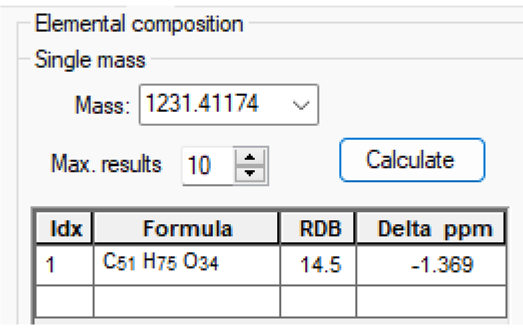

Elemental composition

Single mass

Mass: 1231.41174

Max. results 10

Calculate

Idx	Formula	RDB	Delta ppm
1	$C_{51}H_{75}O_{34}$	14.5	-1.369

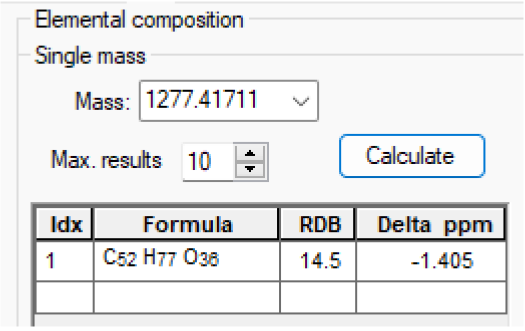

Elemental composition

Single mass

Mass: 1277.41711

Max. results 10

Calculate

Idx	Formula	RDB	Delta ppm
1	$C_{52}H_{77}O_{36}$	14.5	-1.405

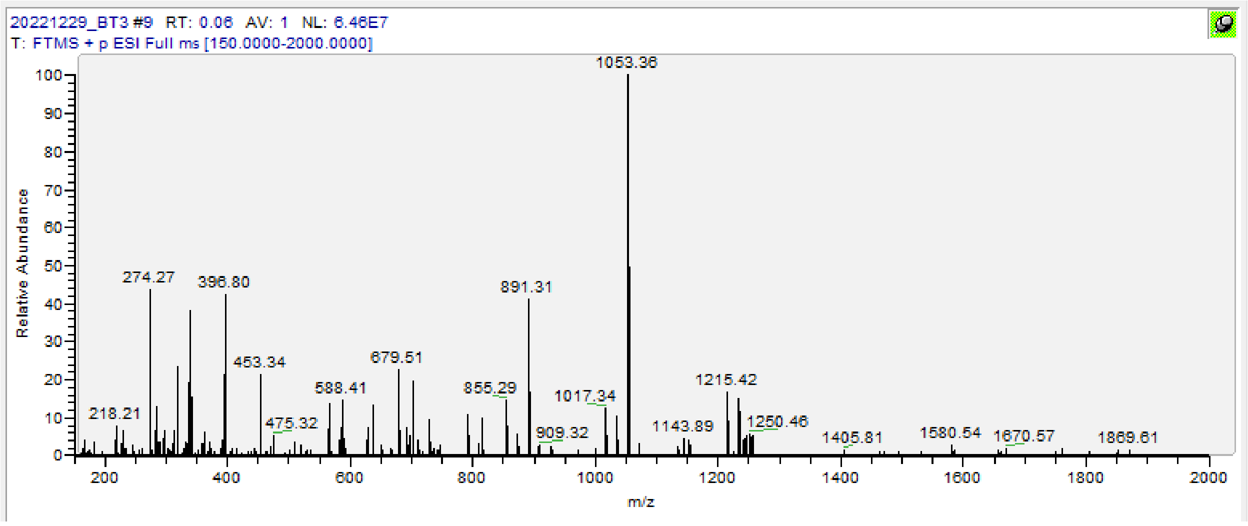

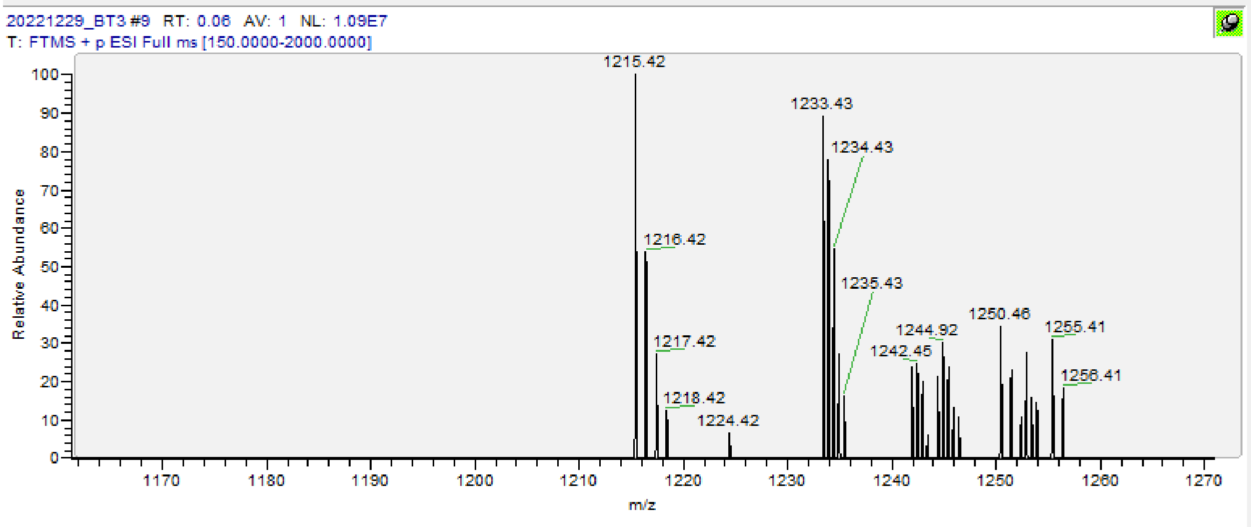

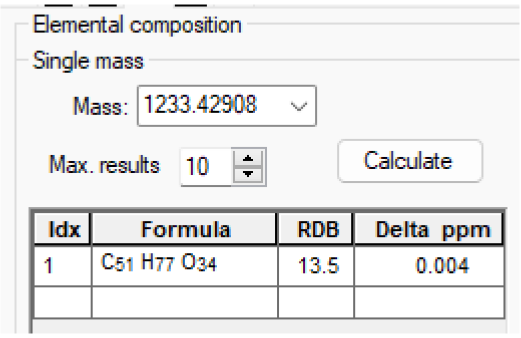

Elemental composition

Single mass

Mass: 1233.42908

Max. results 10

Calculate

Idx	Formula	RDB	Delta ppm
1	$C_{51}H_{77}O_{34}$	13.5	0.004

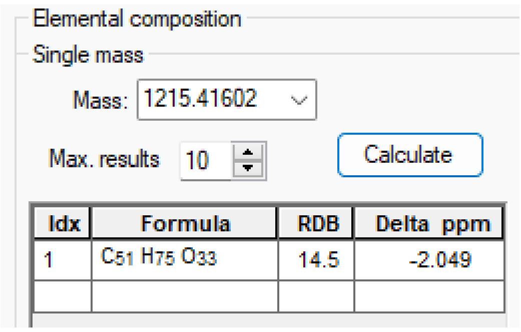

Elemental composition

Single mass

Mass: 1215.41602

Max. results 10

Calculate

Idx	Formula	RDB	Delta ppm
1	$C_{51}H_{75}O_{33}$	14.5	-2.049

Figure S40. UV spectrum (in MeOH) of compound **4**

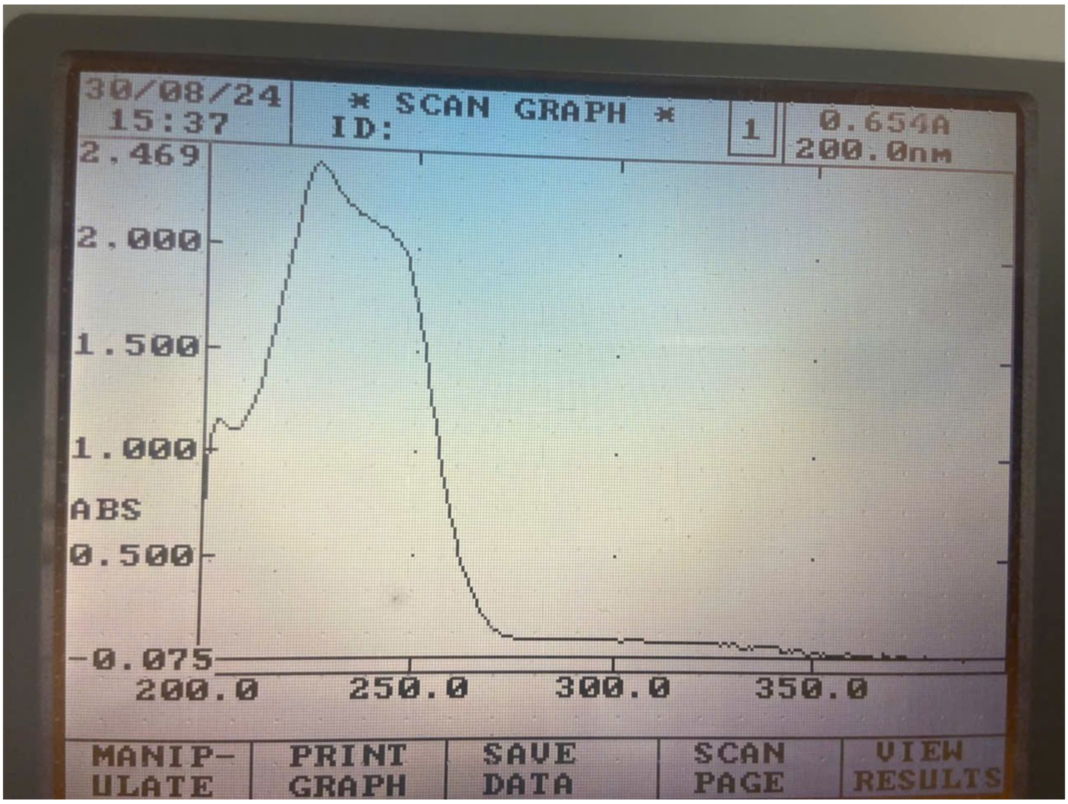

Figure S41. IR (ATR) spectrum of compound **4**

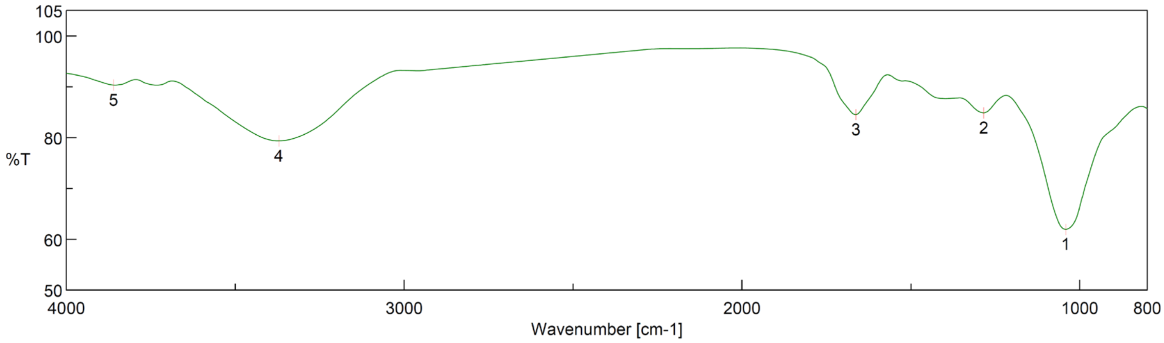

[Result of Peak Picking]

No.	Position	Intensity	No.	Position	Intensity	No.	Position	Intensity
1	1041.37	61.9155	2	1284.36	84.8439	3	1662.34	84.4989
4	3370.96	79.3098	5	3860.79	90.3065			

Figure S42. ^{1}H-NMR spectrum of compound **5** (600 MHz, CD_3OD)

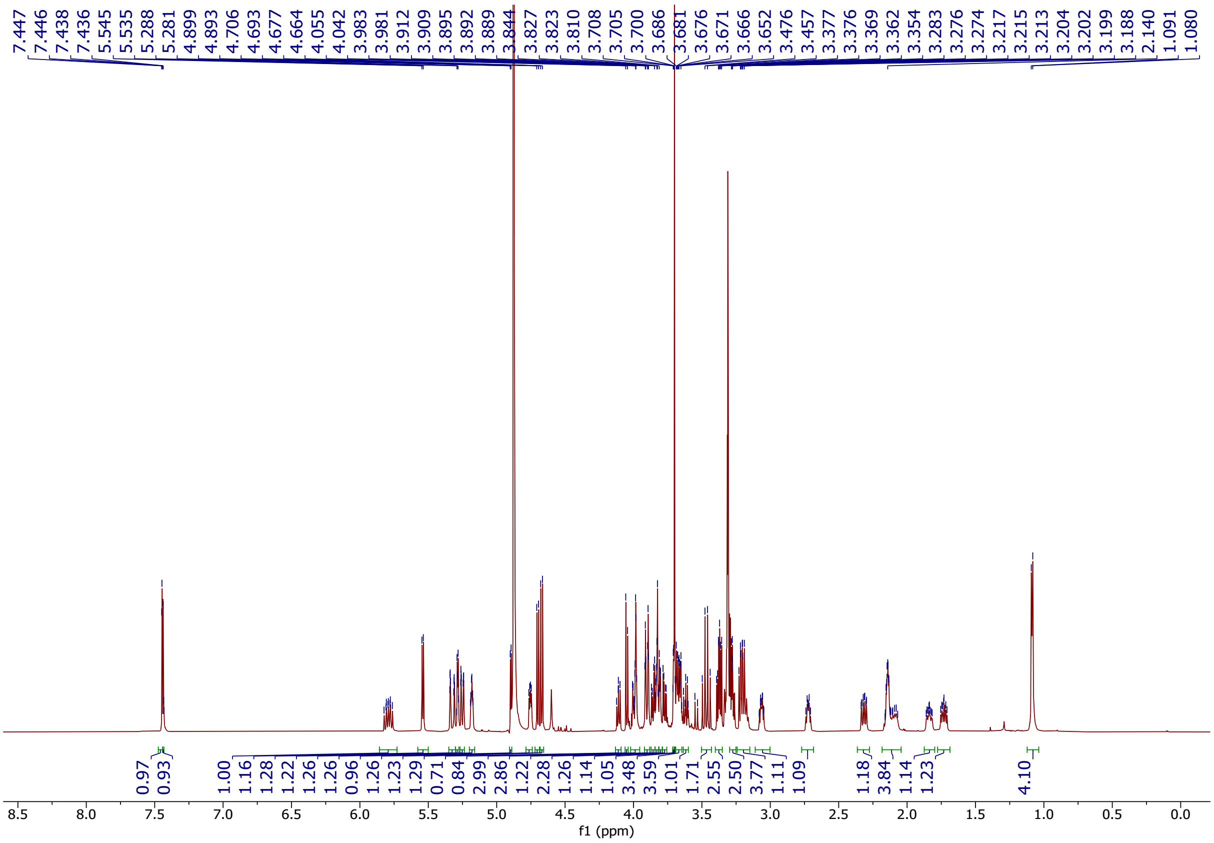

Figure S43. ^{13}C-NMR spectrum of compound **5** (125 MHz, CD_3OD)

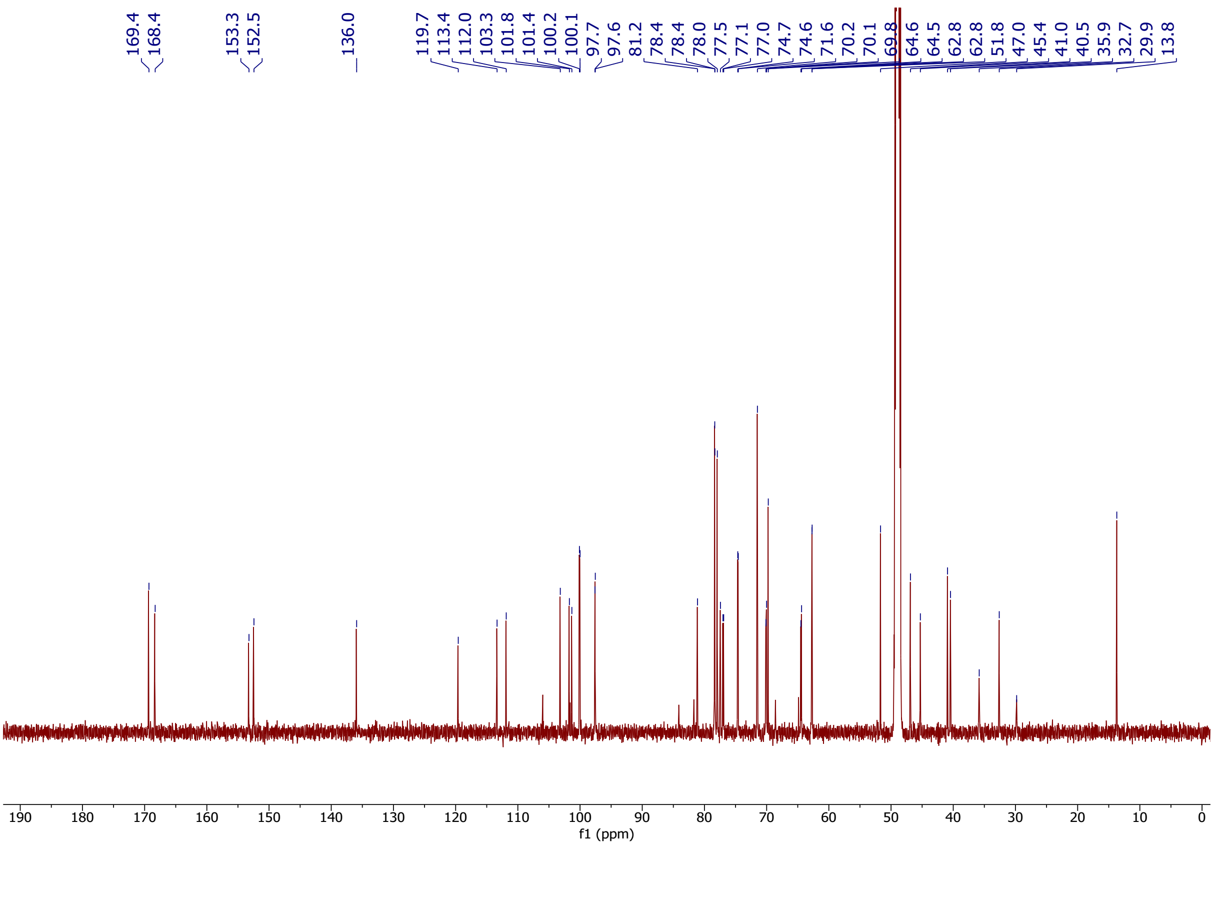

Figure S44. DEPT-NMR spectrum of compound **5** (125 MHz, CD_3OD)

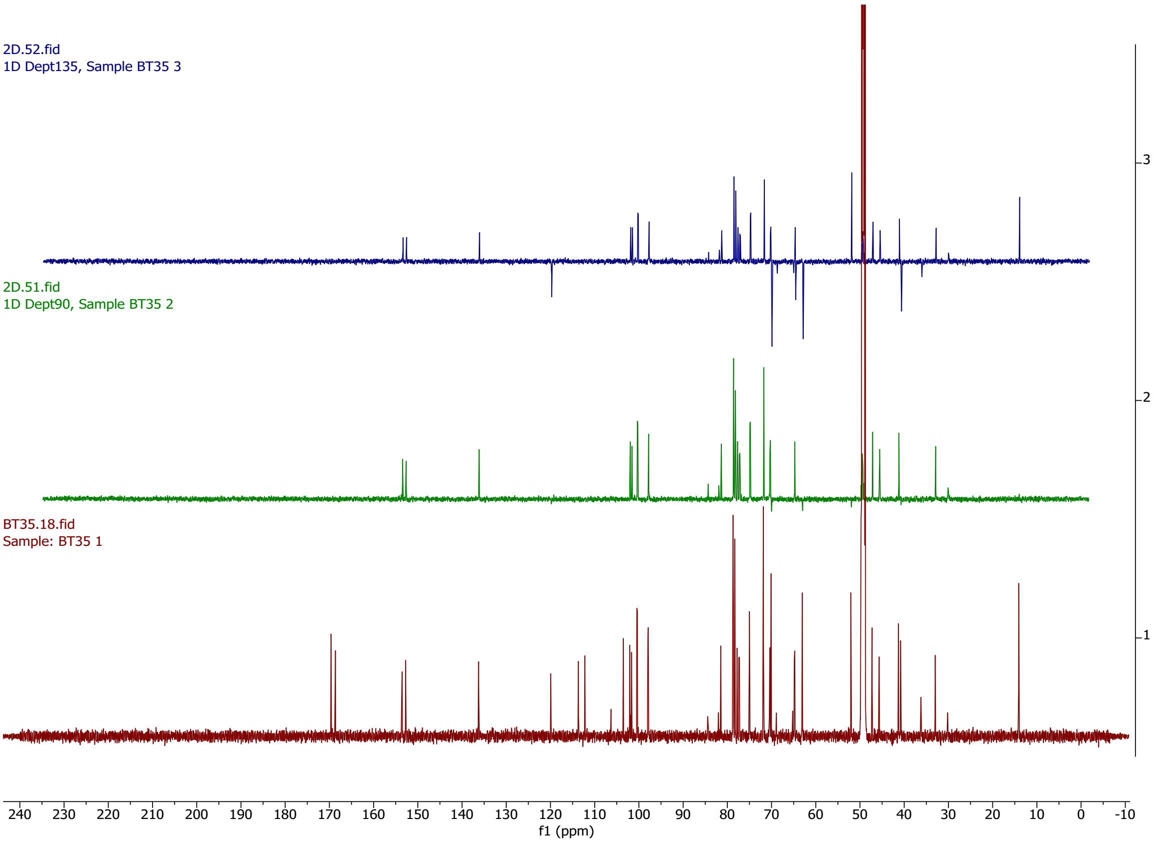

Figure S45. HSQC spectrum of compound **5** (600 MHz, CD_3OD)

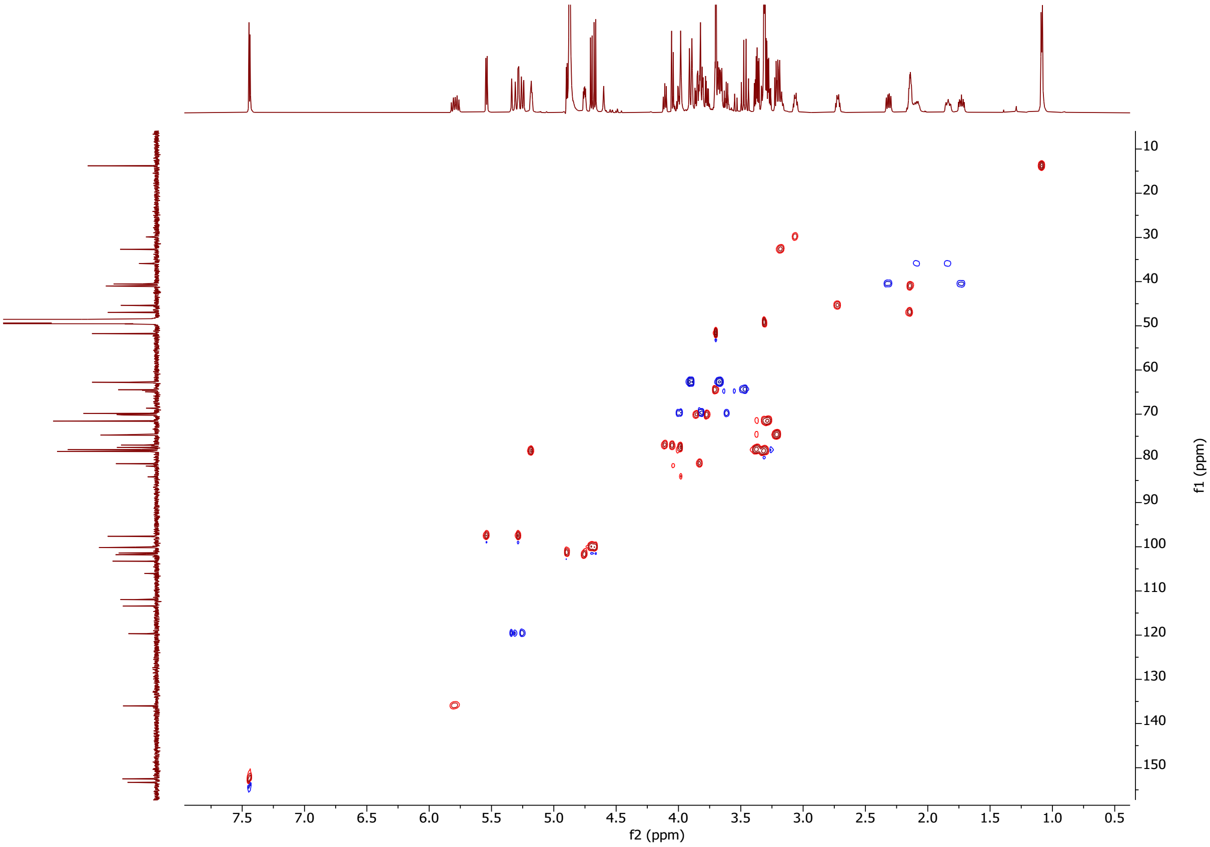

Figure S46. COSY spectrum of compound **5** (600 MHz, CD_3OD)

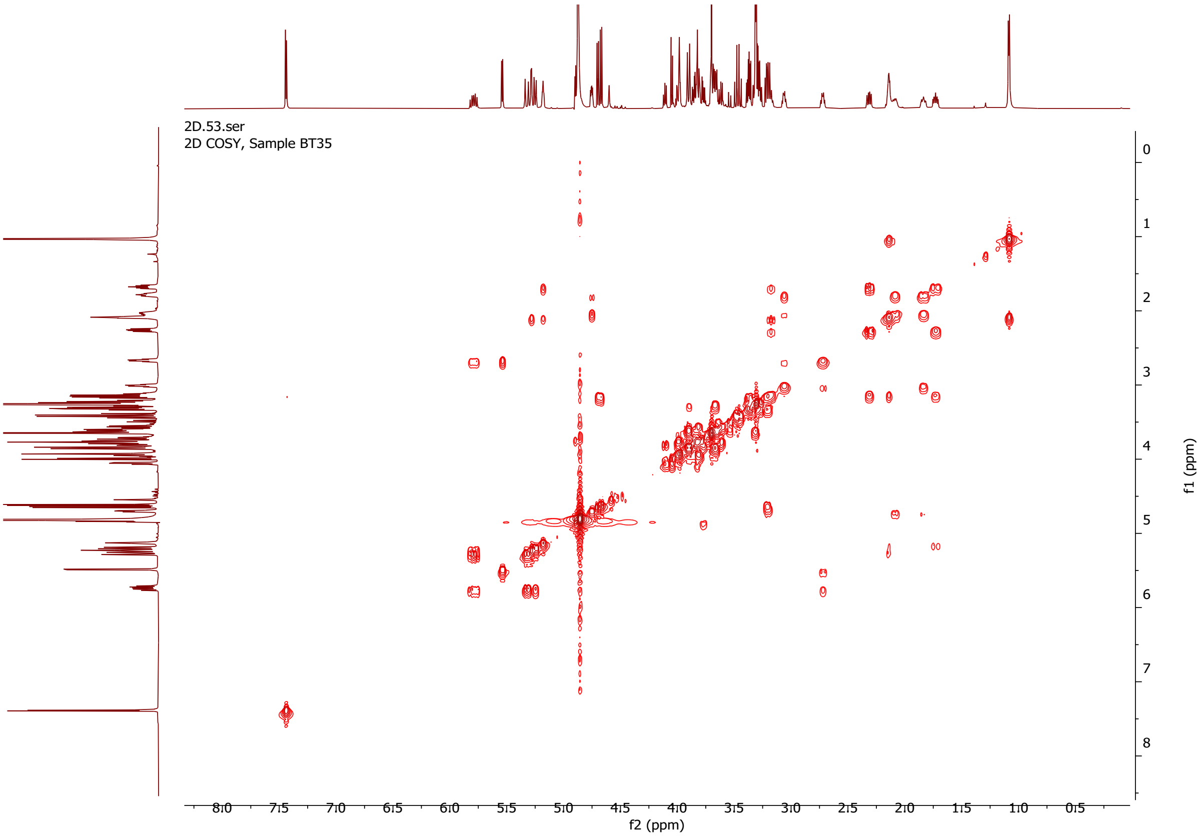

Figure S47. HMBC spectrum of compound **5** (600 MHz, CD_3OD)

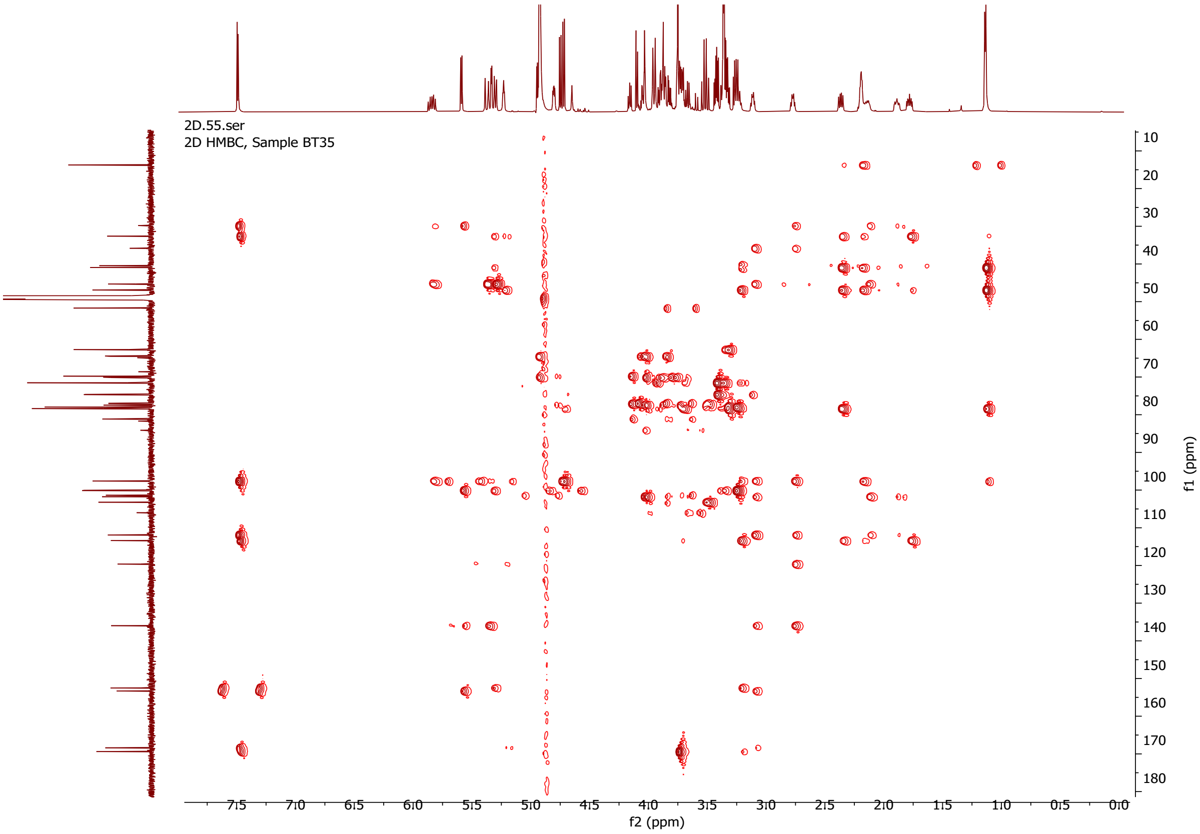

Figure S48. ROESY spectrum of compound **5** (600 MHz, CD_3OD)

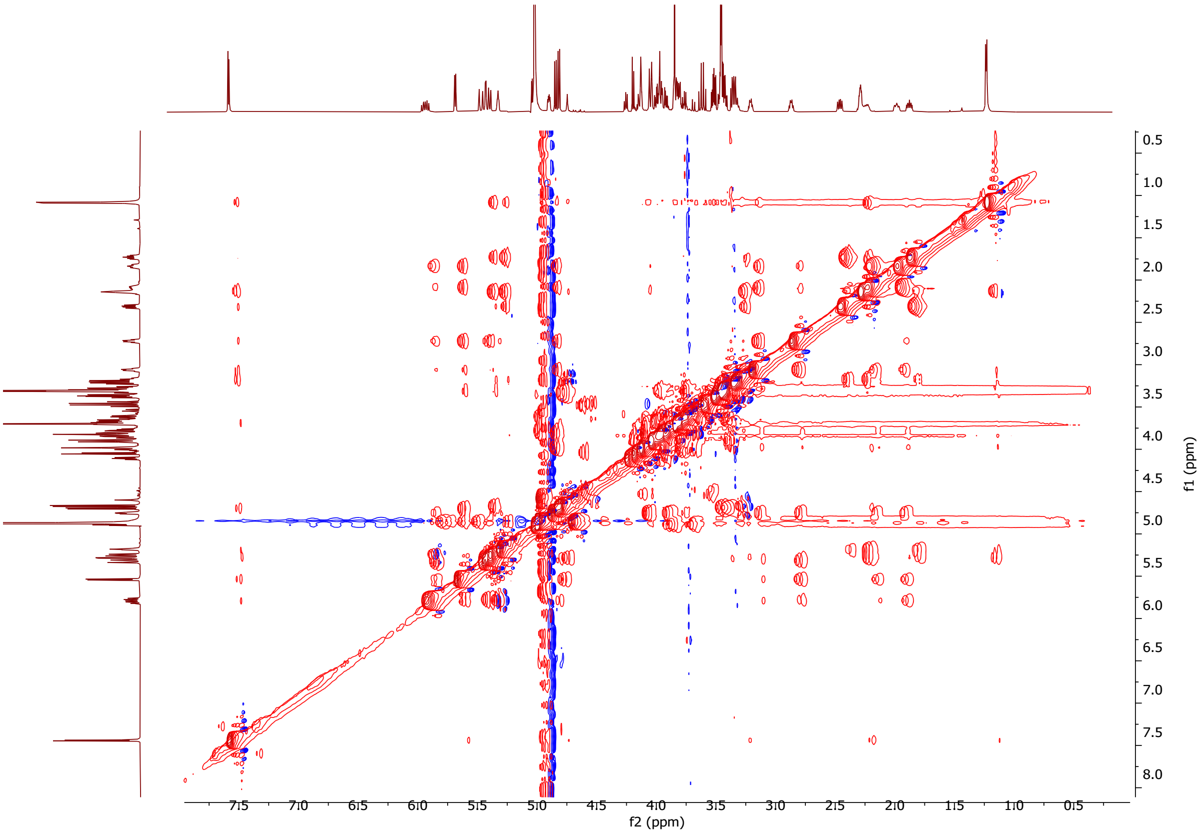

Figure S49. HR-ESI-MS data of compound **5**

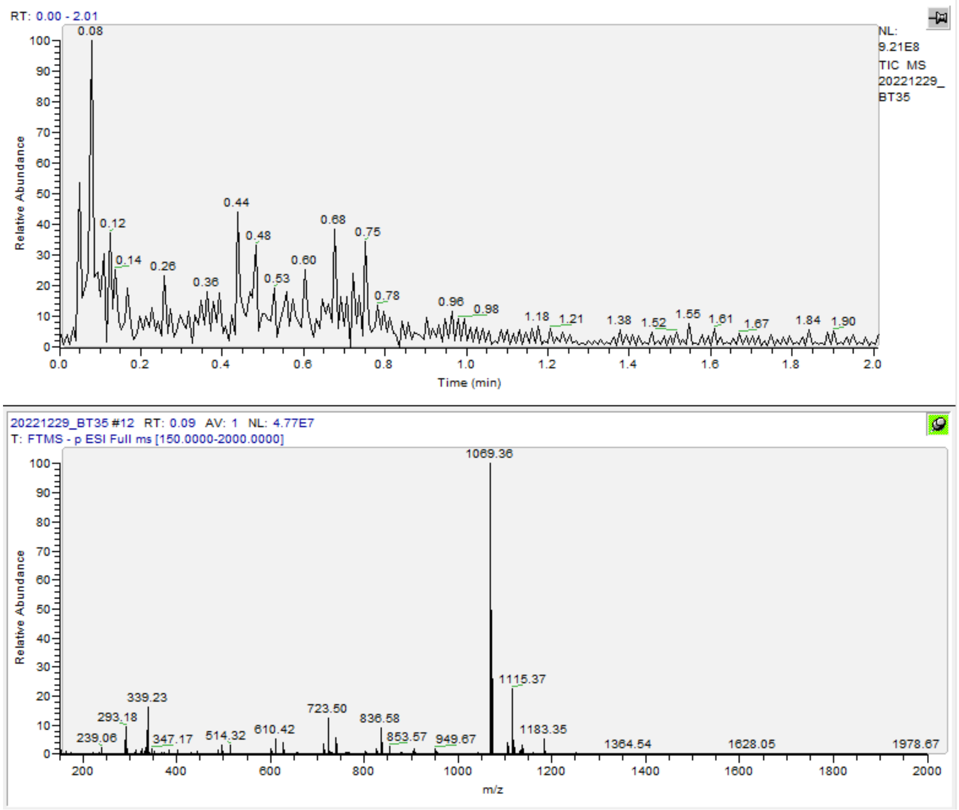

Elemental composition

Single mass

Mass: 1069.35974

Max. results 10

Calculate

Idx	Formula	RDB	Delta ppm
1	$C_{45}H_{65}O_{29}$	13.5	-0.806

Elemental composition

Single mass

Mass: 1115.36523

Max. results 10

Calculate

Idx	Formula	RDB	Delta ppm
1	$C_{46}H_{67}O_{31}$	13.5	-0.763

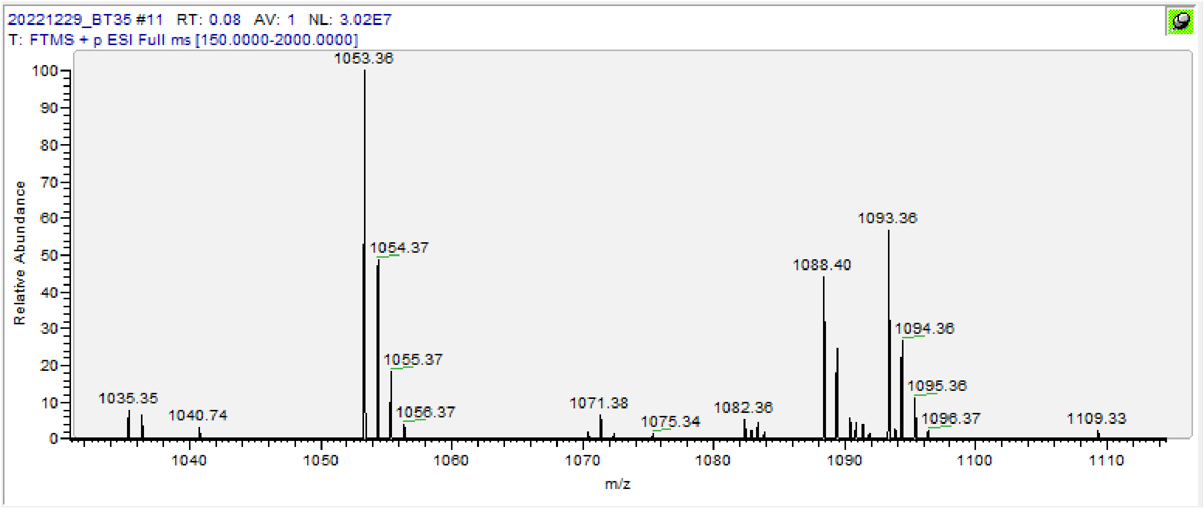

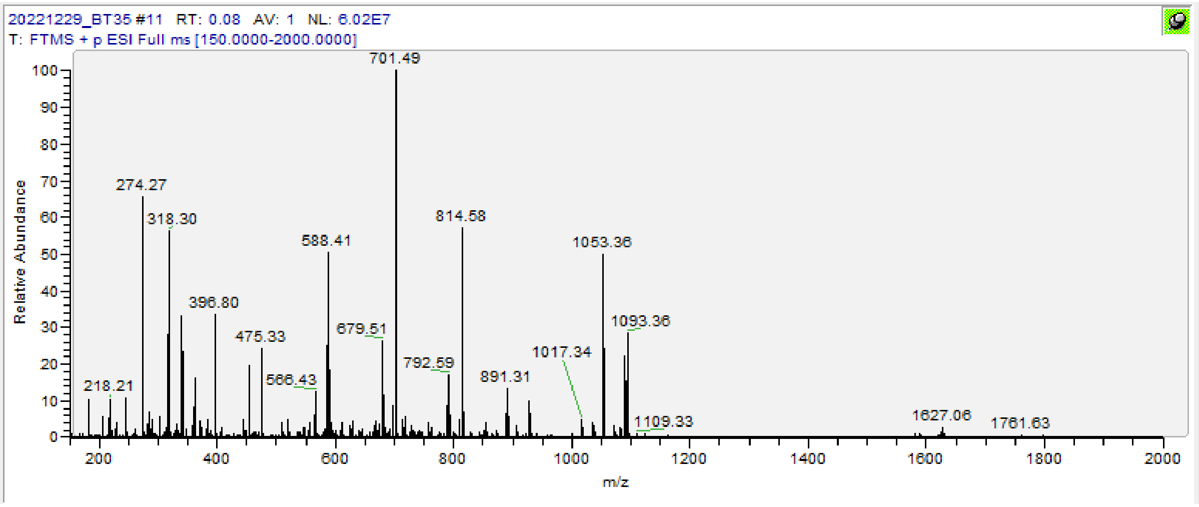

Elemental composition

Single mass

Mass: 1053.36401

Max. results 10

Calculate

Idx	Formula	RDB	Delta ppm
1	$C_{45}H_{65}O_{28}$	13.5	-1.592

Elemental composition

Single mass

Mass: 1093.35693

Max. results 10

Calculate

Idx	Formula	RDB	Delta ppm
1	$C_{45}H_{66}O_{29}Na$	12.5	-1.159

Figure S50. UV spectrum (in MeOH) of compound **5**

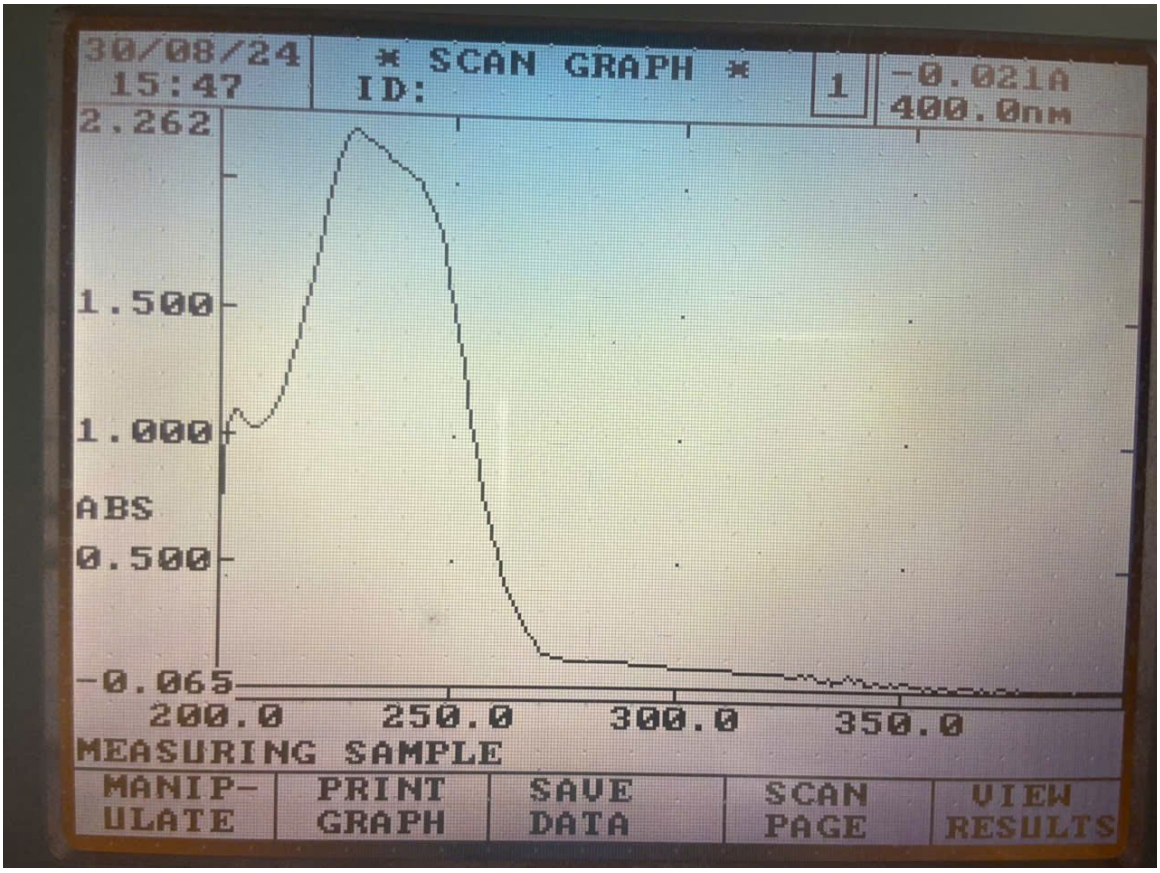

Figure S51. IR (ATR) spectrum of compound **5**

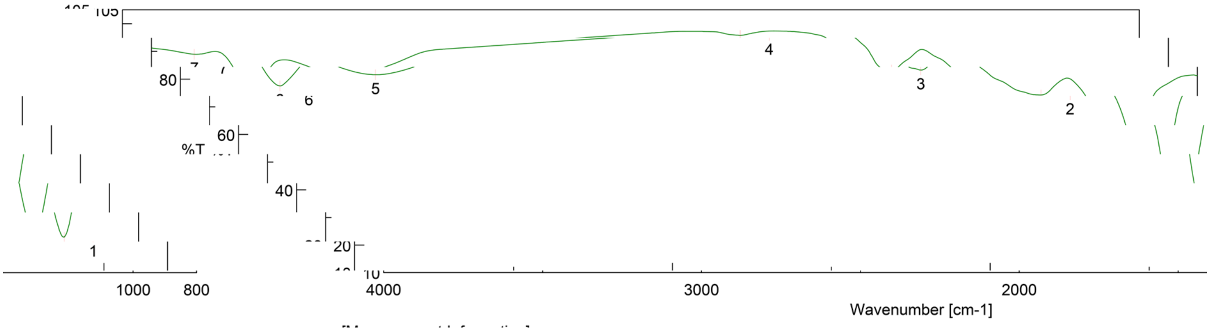

[Result of Peak Picking]

No.	Position	Intensity	No.	Position	Intensity	No.	Position	Intensity
1	1033.66	22.8809	2	1292.07	74.2009	3	1670.05	83.2218
4	2055.75	95.7342	5	3386.39	81.5131	6	3687.23	77.4809
7	3864.65	88.9228						

Figure S52. ^{1}H-NMR spectrum of compound **6** (600 MHz, CD3OD)

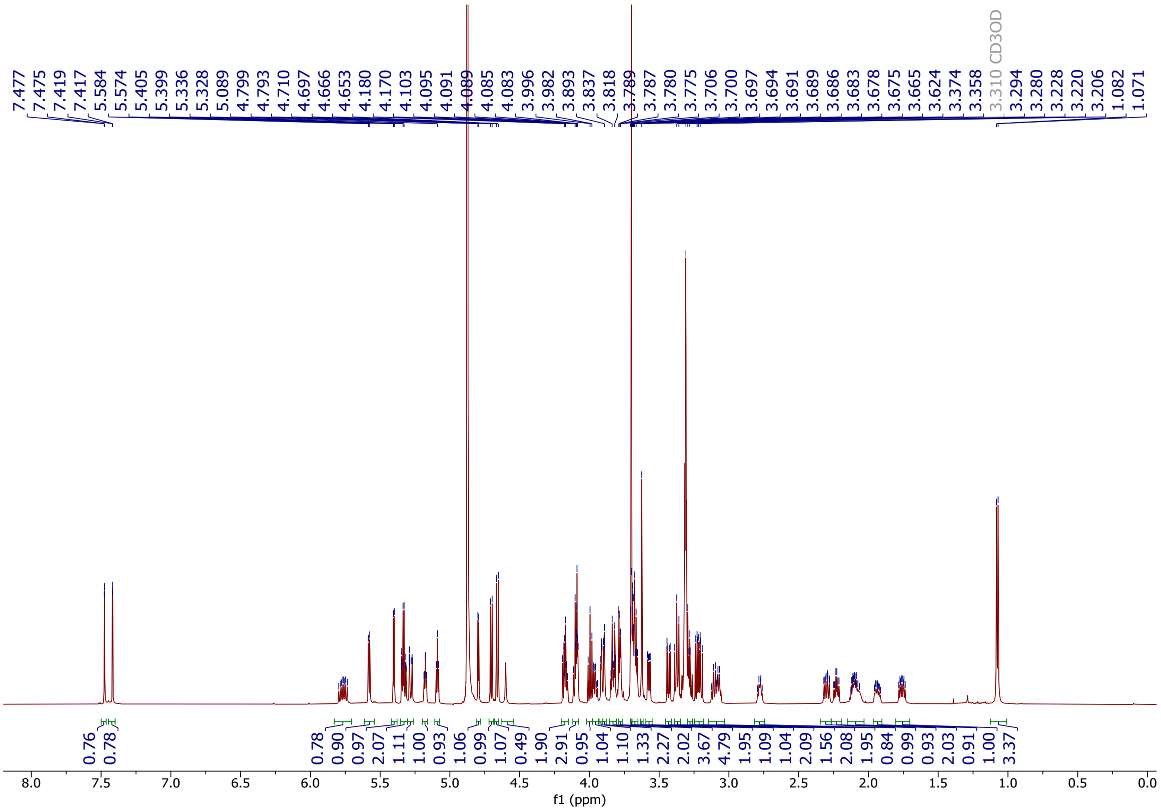

Figure S53. ^{13}C-NMR spectrum of compound **6** (125 MHz, CD3OD)

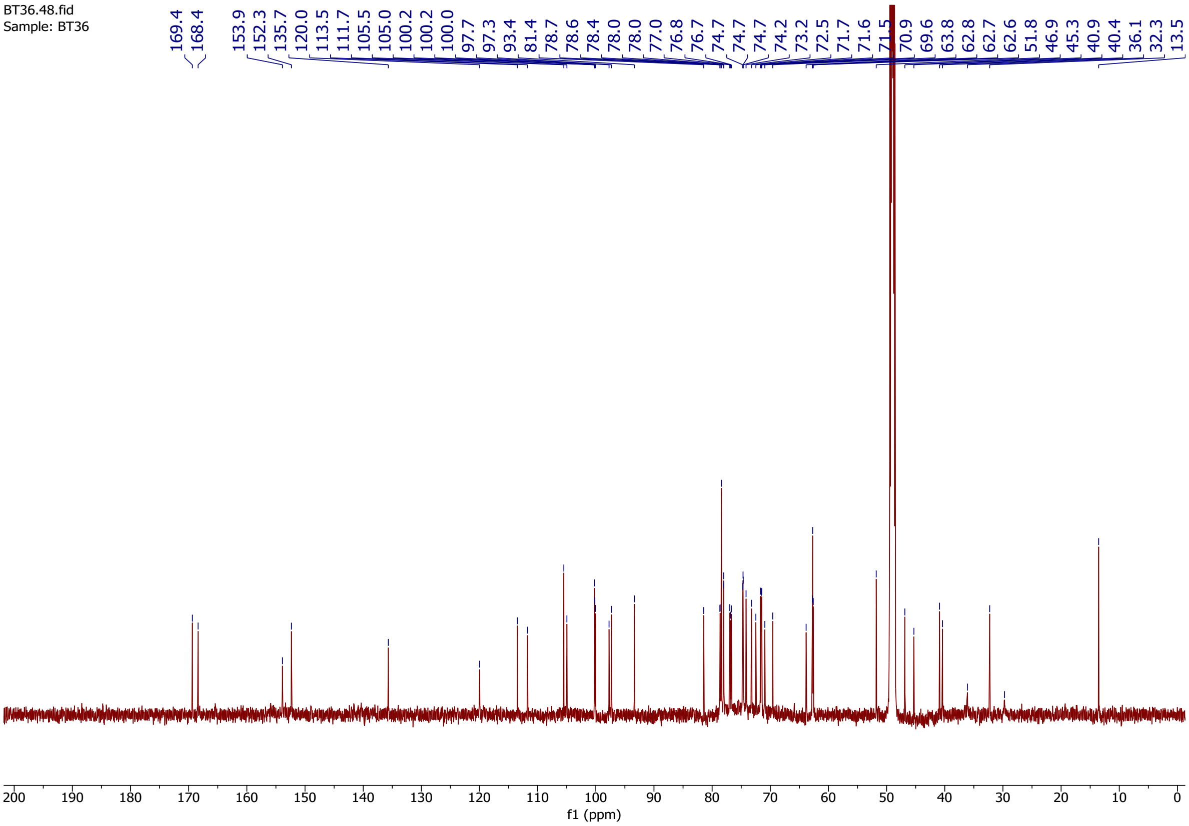

Figure S54. DEPT-NMR spectrum of compound **6** (125 MHz, CD_3OD)

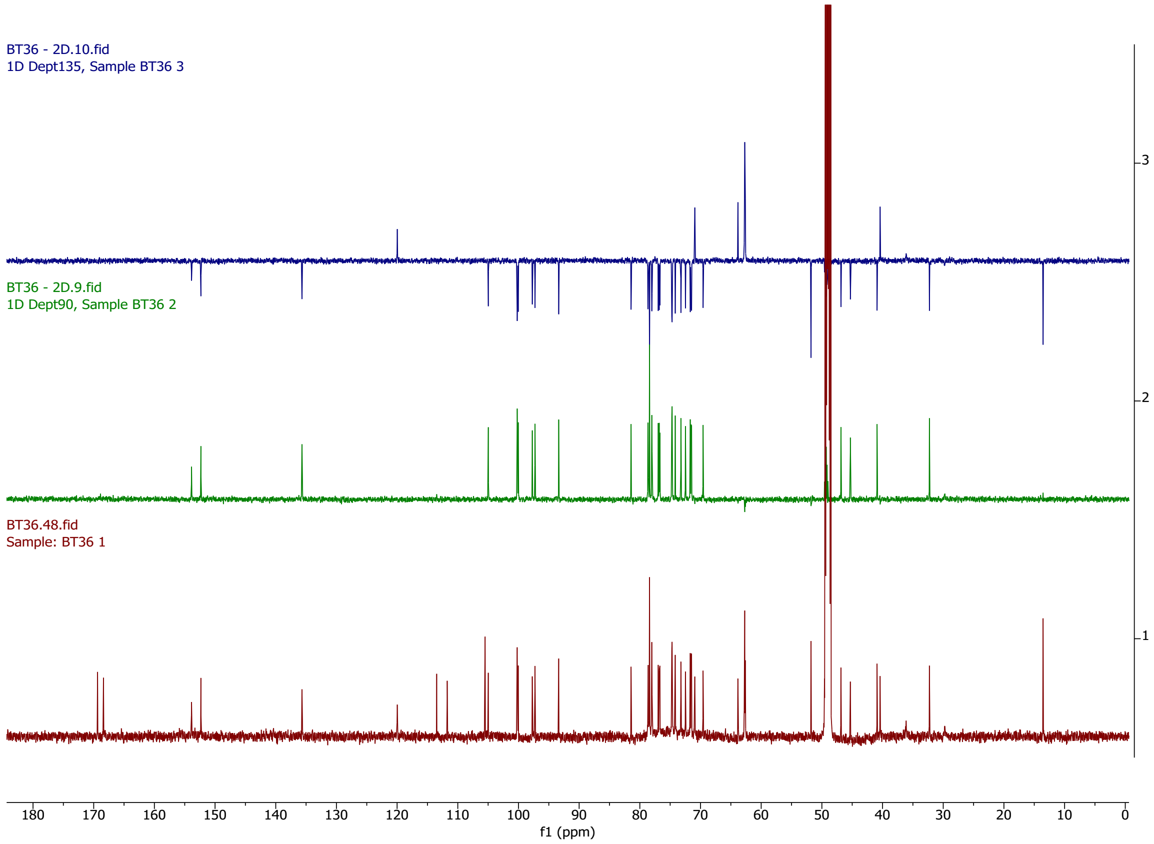

Figure S55. HSQC spectrum of compound **6** (600 MHz, CD_3OD)

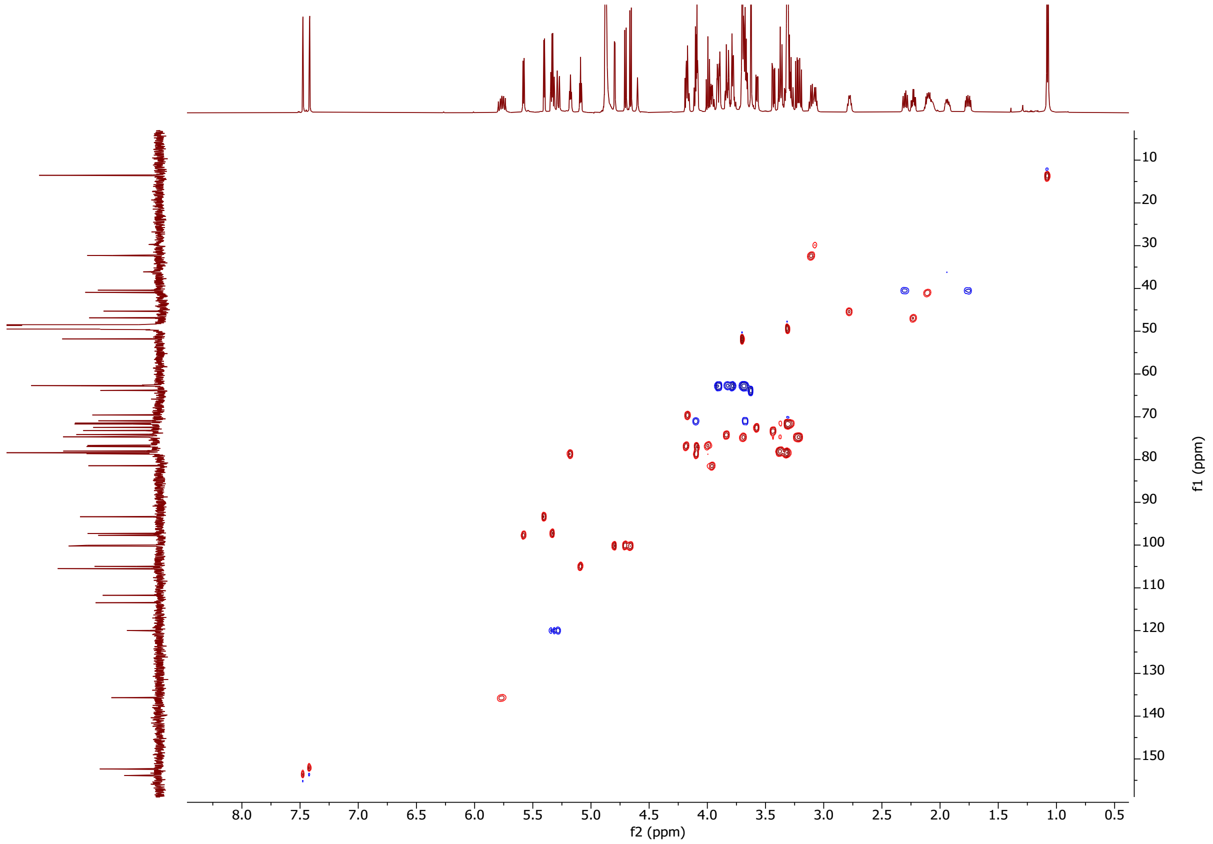

Figure S56. COSY spectrum of compound **6** (600 MHz, CD3OD)

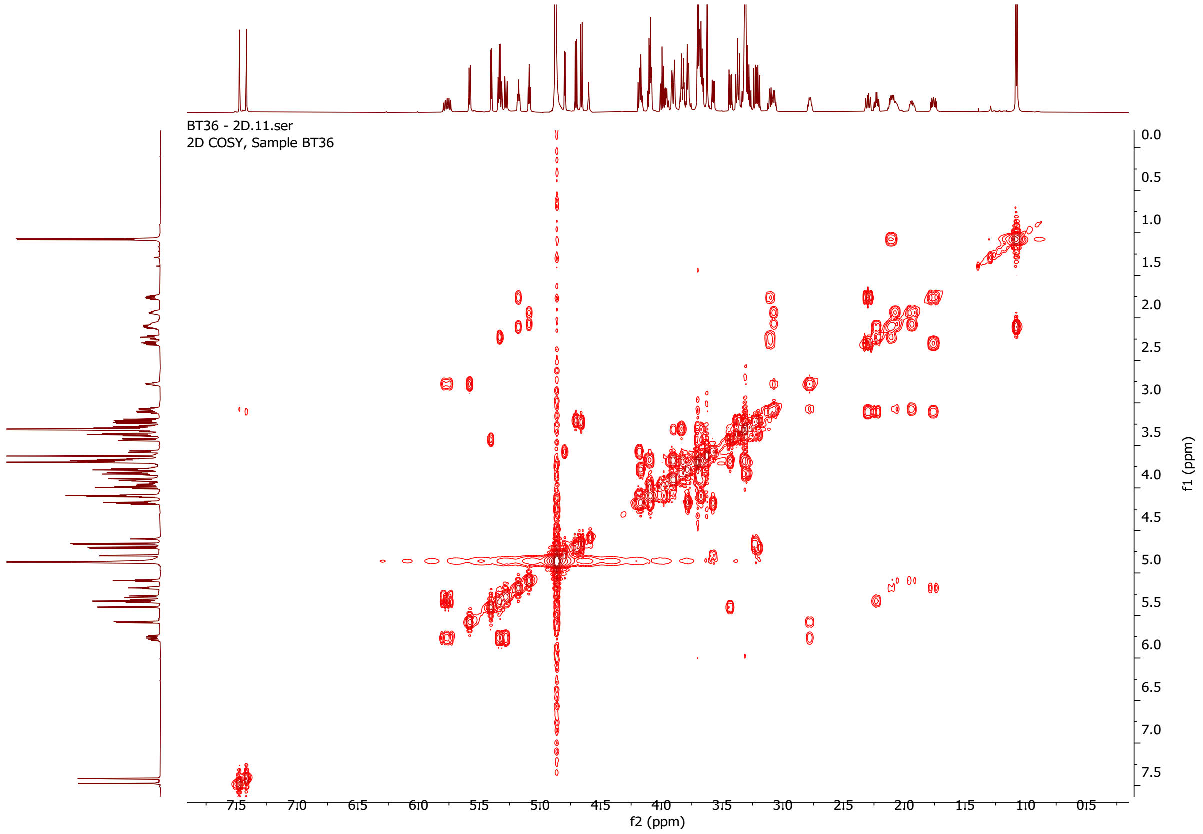

Figure S57. HMBC spectrum of compound **6** (600 MHz, CD3OD)

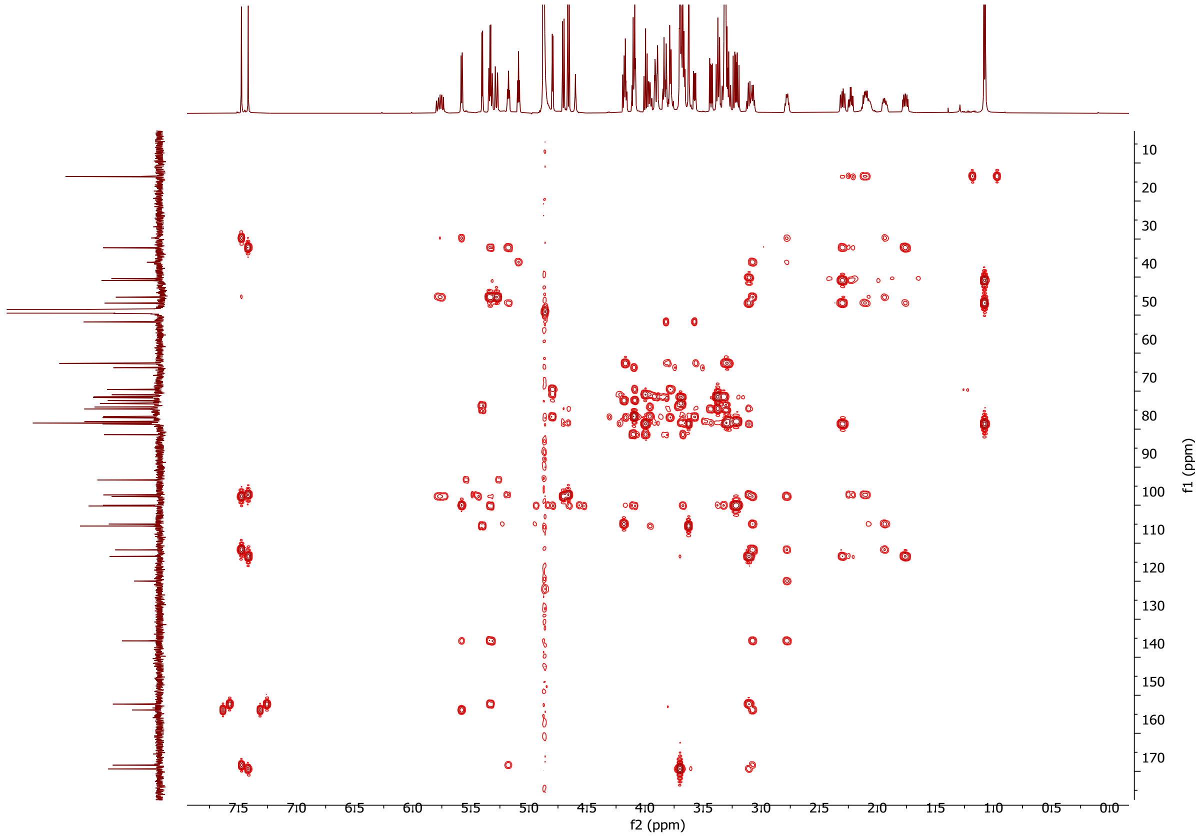

Figure S58. ROESY spectrum of compound **6** (600 MHz, CD_3OD)

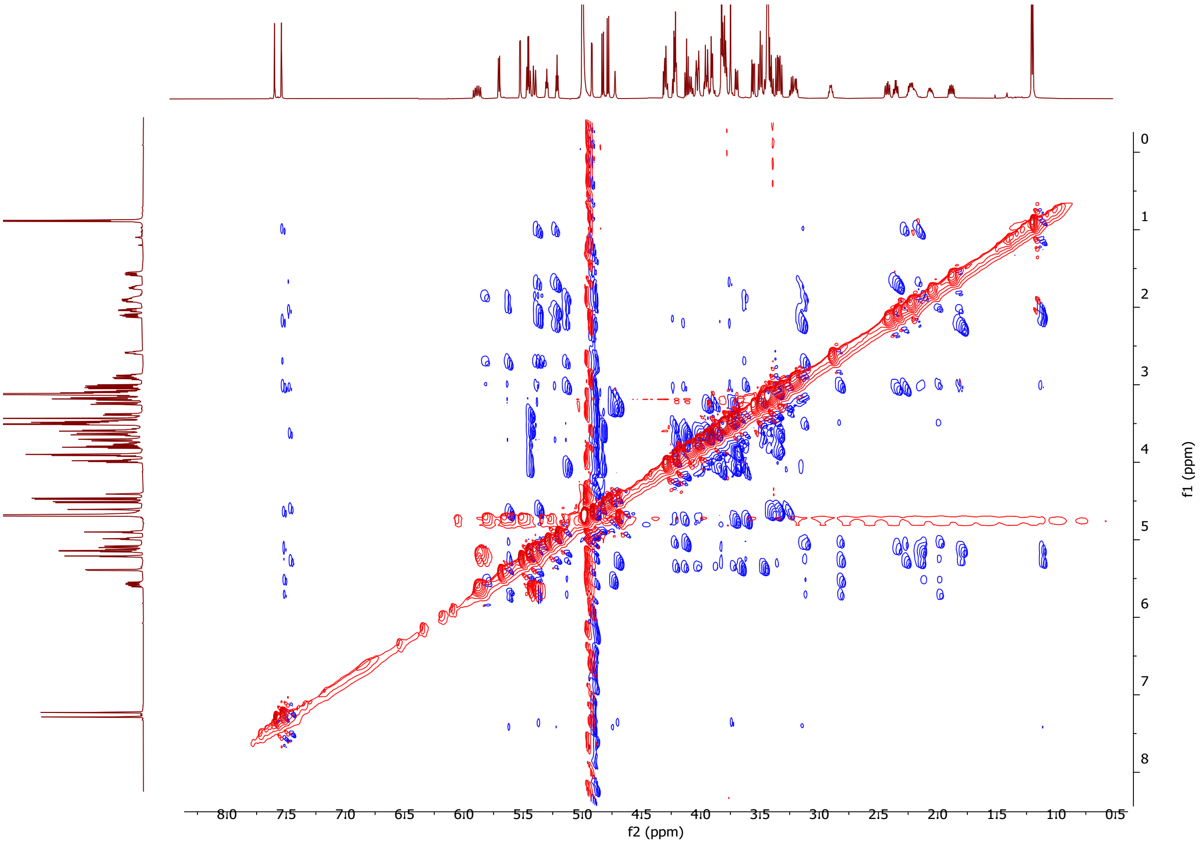

Figure S59. HR-ESI-MS data of compound **6**

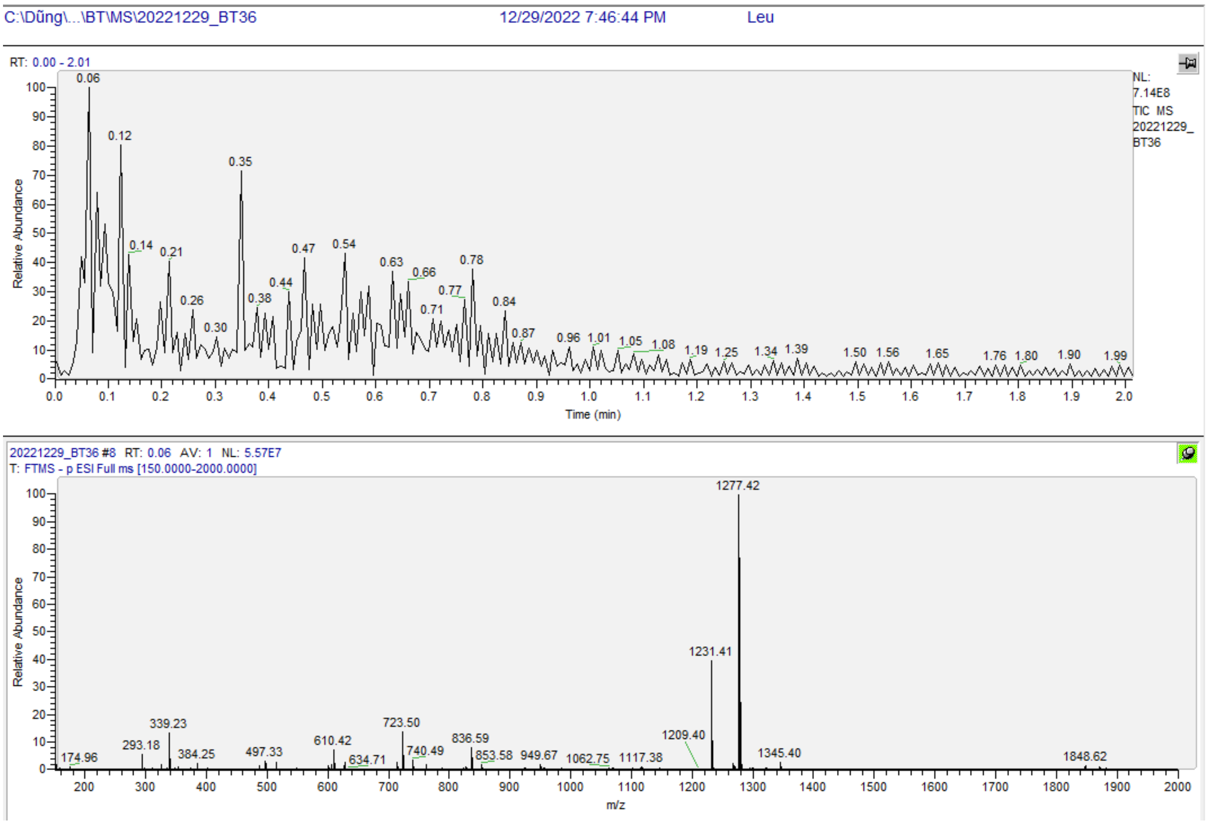

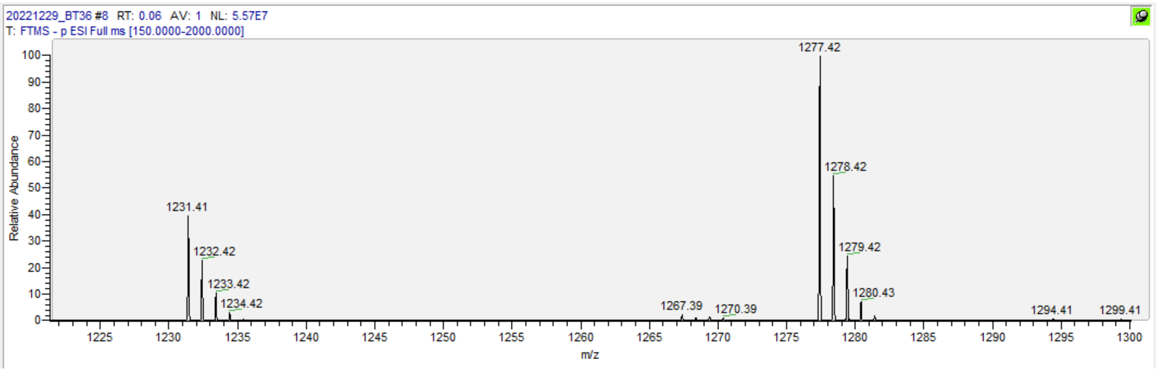

Elemental composition

Single mass

Mass: 1231.41235

Max. results 10 Calculate

Idx	Formula	RDB	Delta ppm
1	$C_{51}H_{75}O_{34}$	14.5	-0.873

Elemental composition

Single mass

Mass: 1277.41797

Max. results 10 Calculate

Idx	Formula	RDB	Delta ppm
1	$C_{52}H_{77}O_{36}$	14.5	-0.732

Figure S60. UV spectrum (in MeOH) of compound **6**

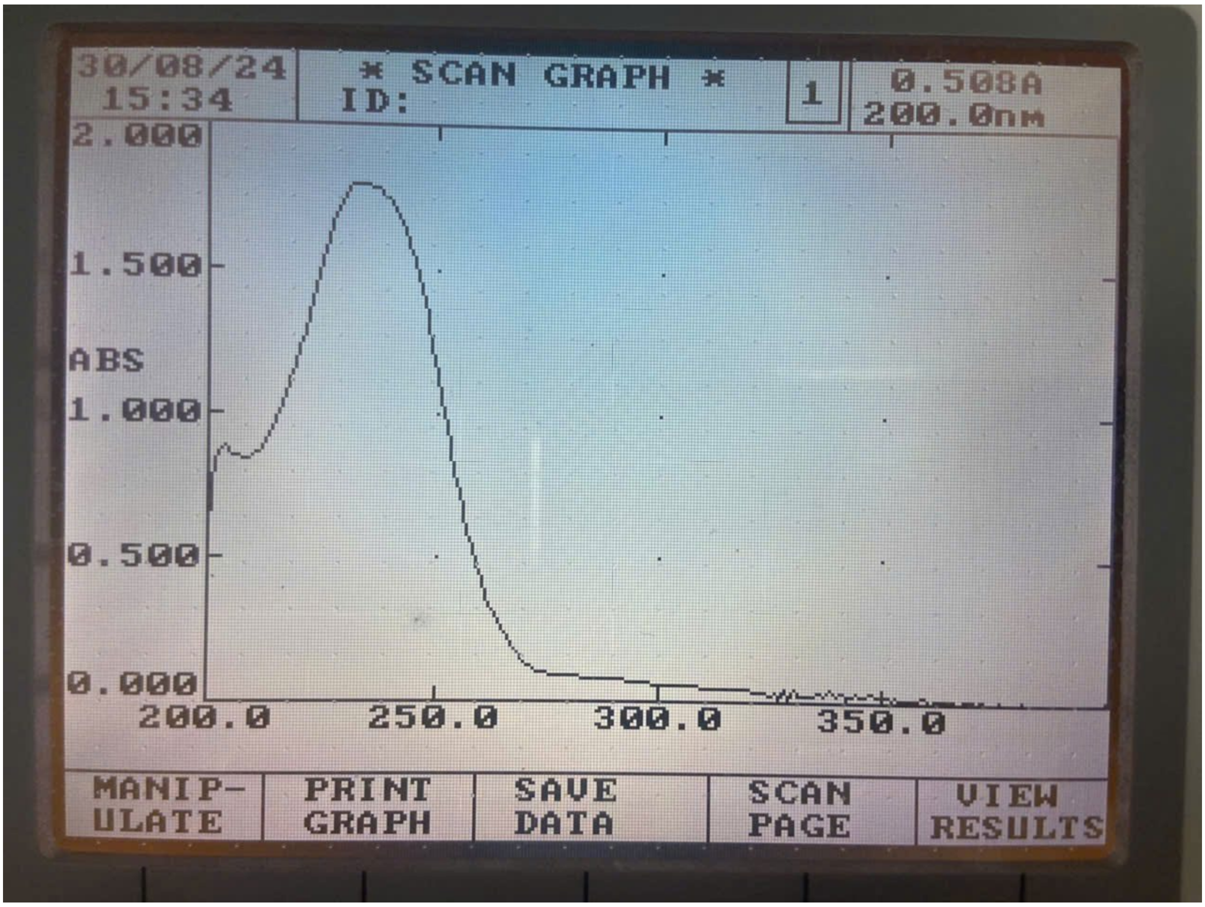

Figure S61. IR (ATR) spectrum of compound **6**

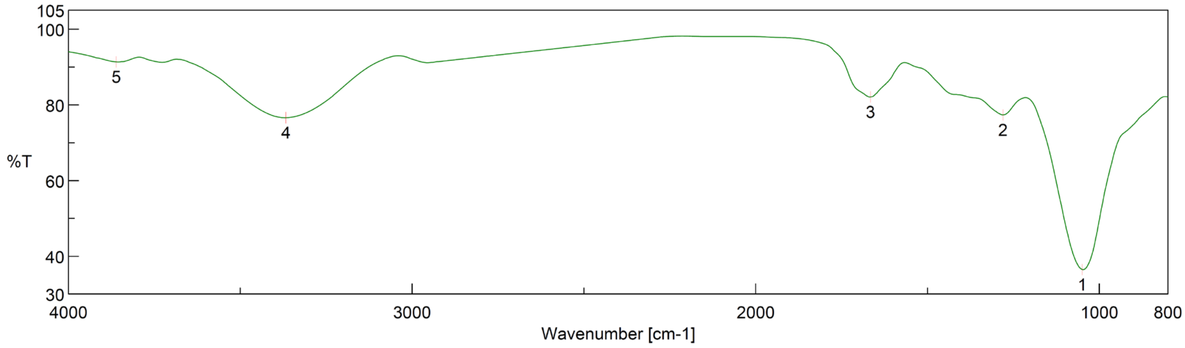

[Result of Peak Picking]

No.	Position	Intensity	No.	Position	Intensity	No.	Position	Intensity
1	1049.09	36.4331	2	1280.5	77.3054	3	1666.2	82.0186
4	3367.1	76.5612	5	3860.79	91.2864			

Figure S62. ^{1}H-NMR spectrum of compound **7** (600 MHz, CD_3OD)

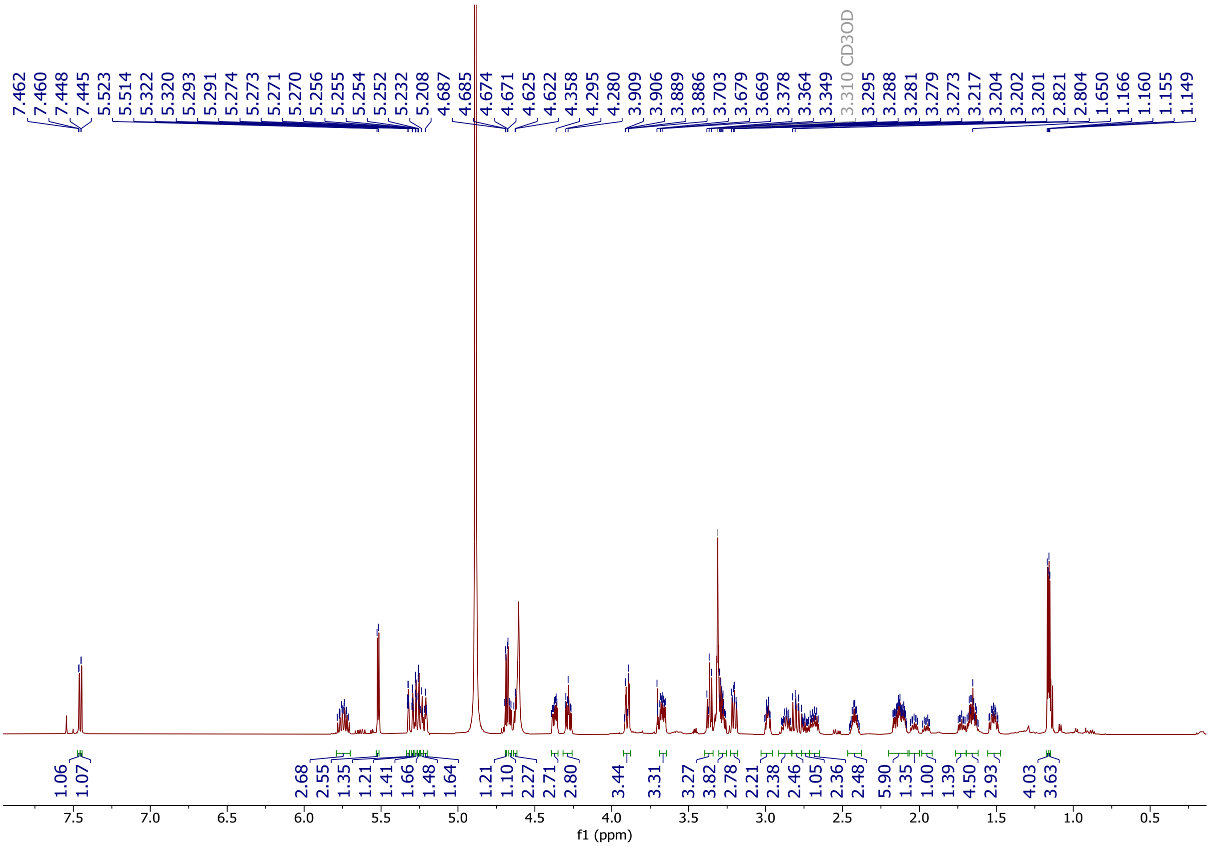

Figure S63. ^{13}C-NMR spectrum of compound **7** (125 MHz, CD_3OD)

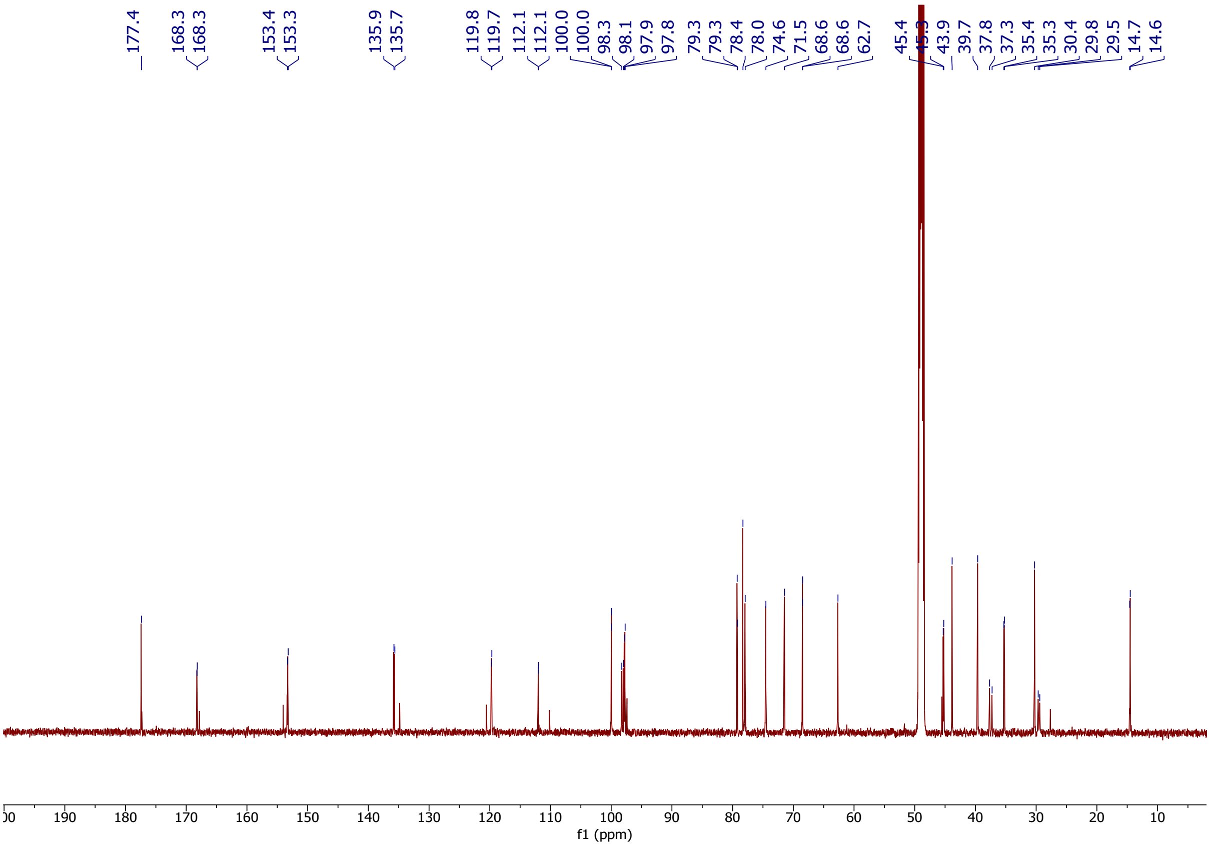

Figure S64. DEPT-NMR spectrum of compound **7** (125 MHz, CD_3OD)

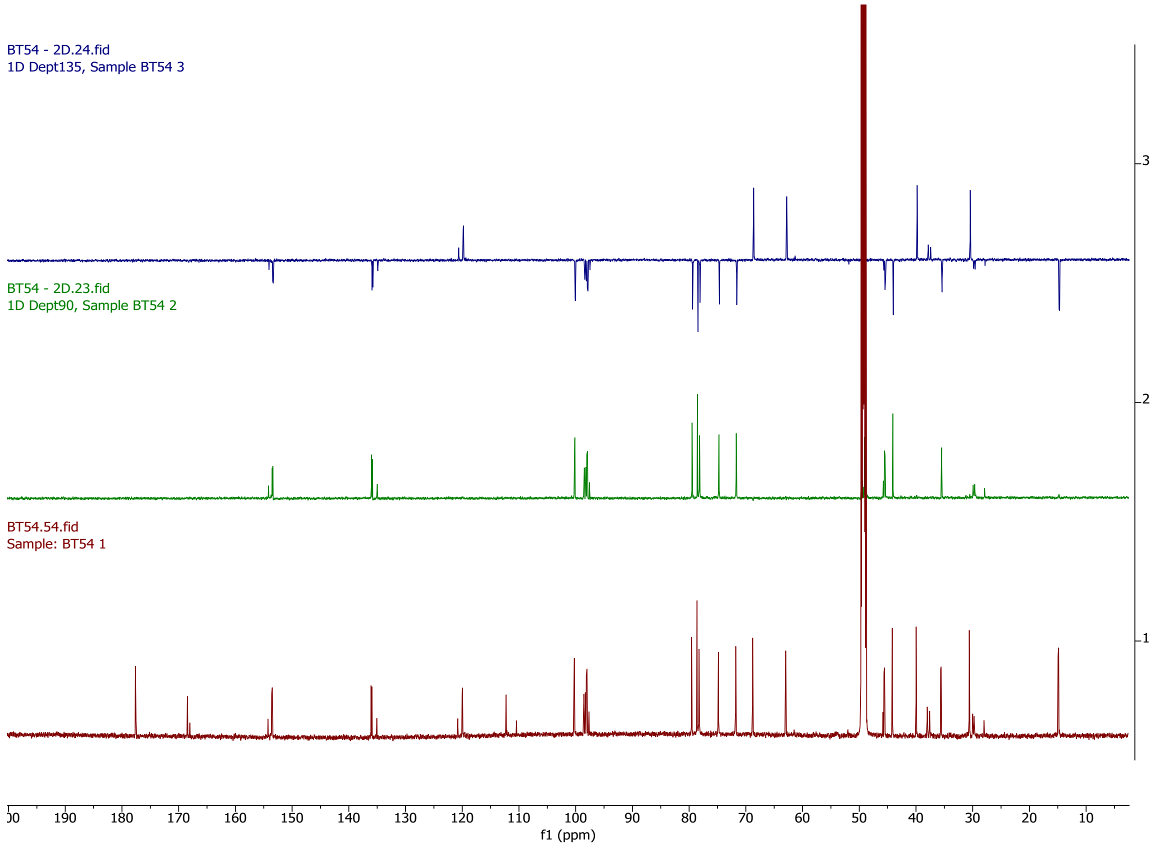

Figure S65. HSQC spectrum of compound **7** (600 MHz, CD_3OD)

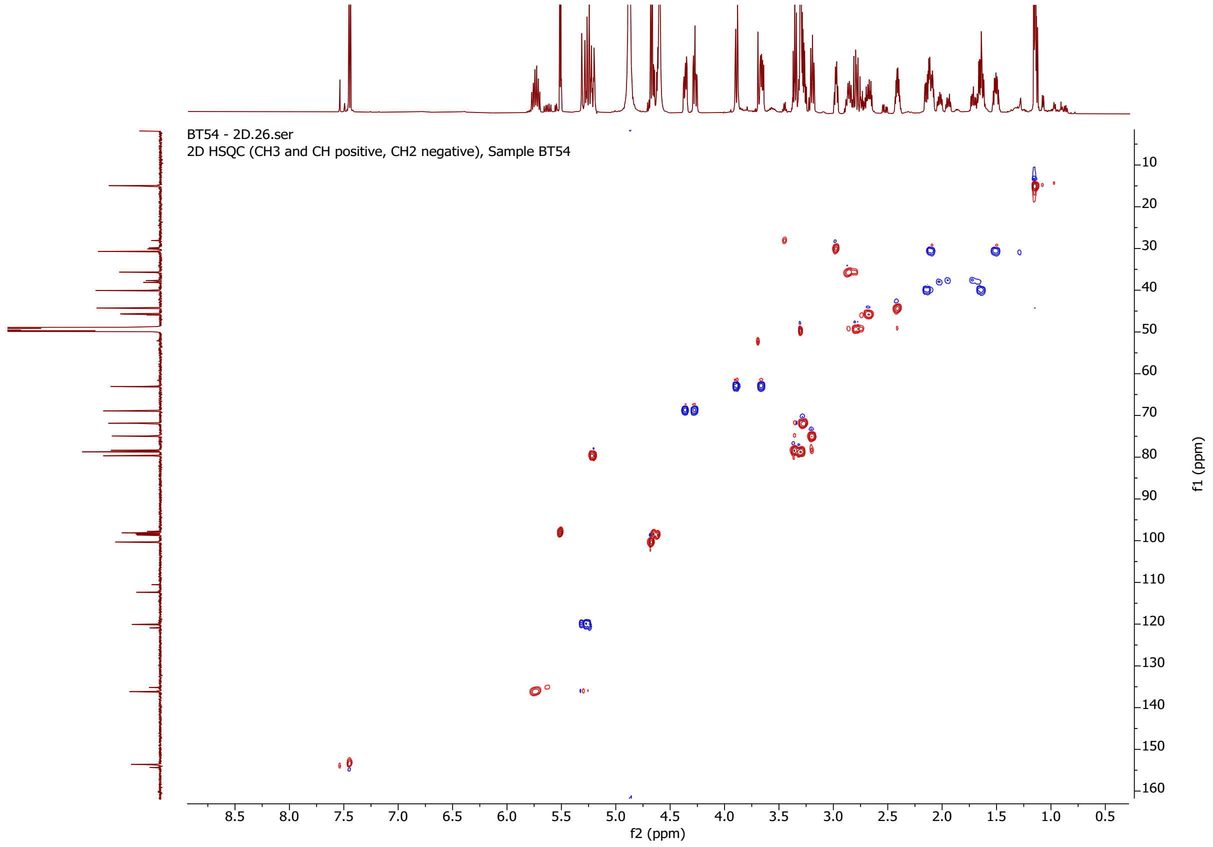

Figure S66. COSY spectrum of compound **7** (600 MHz, CD_3OD)

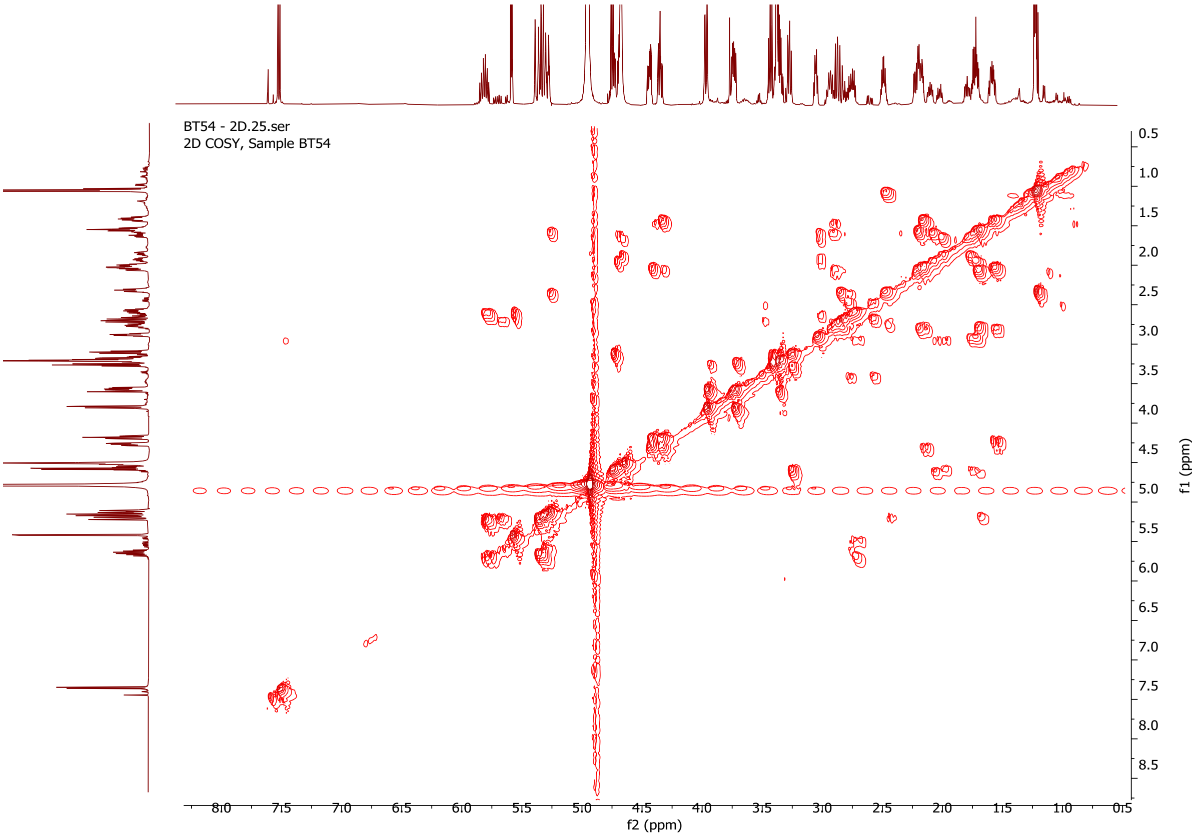

Figure S67. HMBC spectrum of compound **7** (600 MHz, CD_3OD)

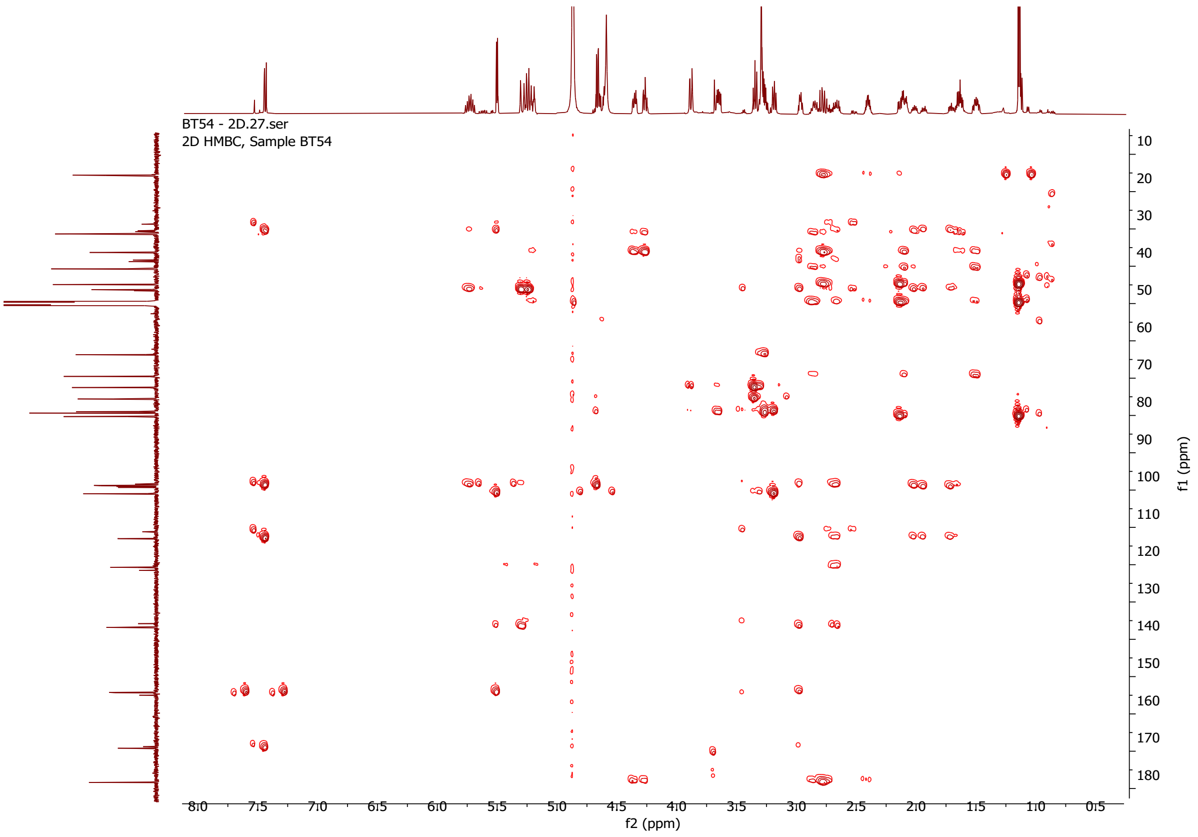

Figure S68. ROESY spectrum of compound **7** (600 MHz, CD_3OD)

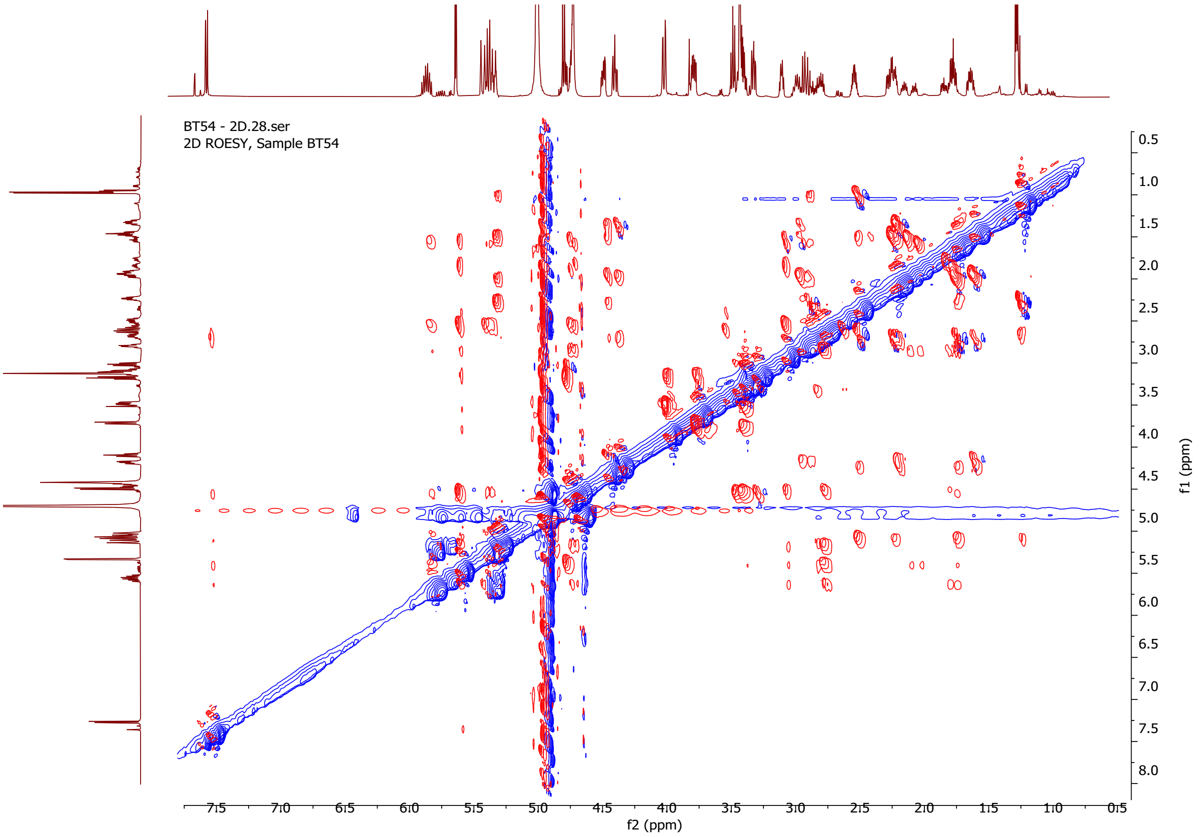

Figure S69. HR-ESI-MS data of compound **7** (600 MHz, CD_3OD)

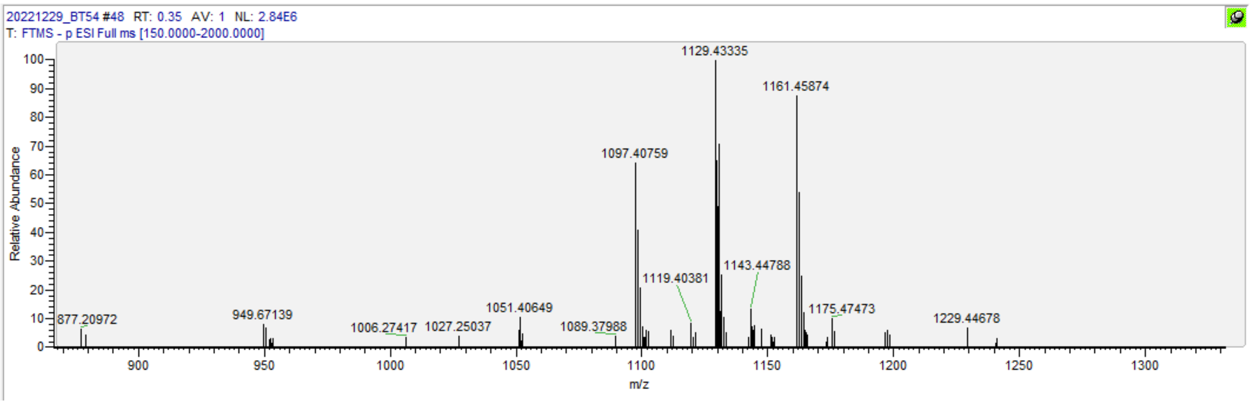

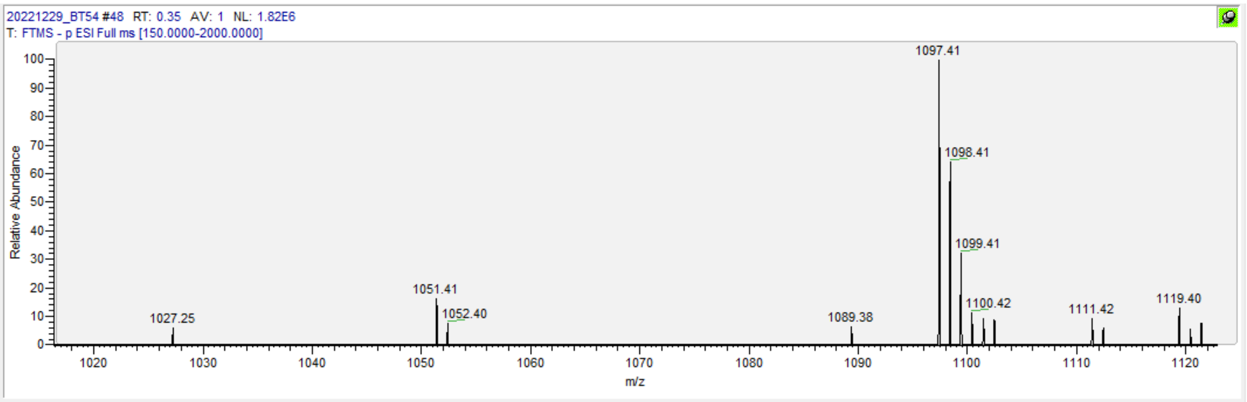

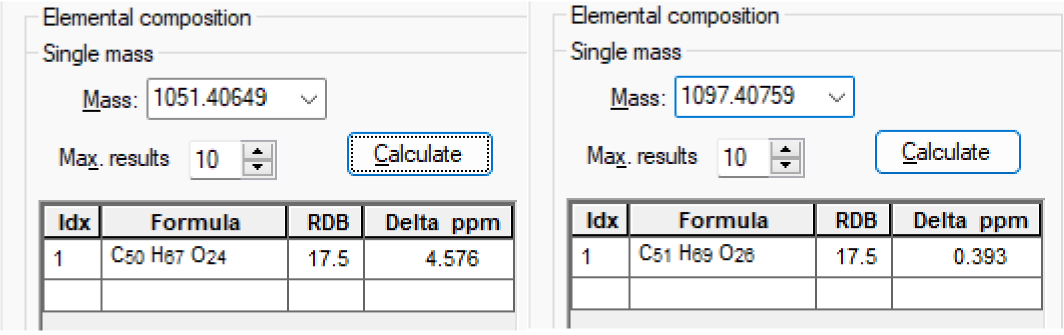

Figure S70. UV spectrum (in MeOH) of compound **7**

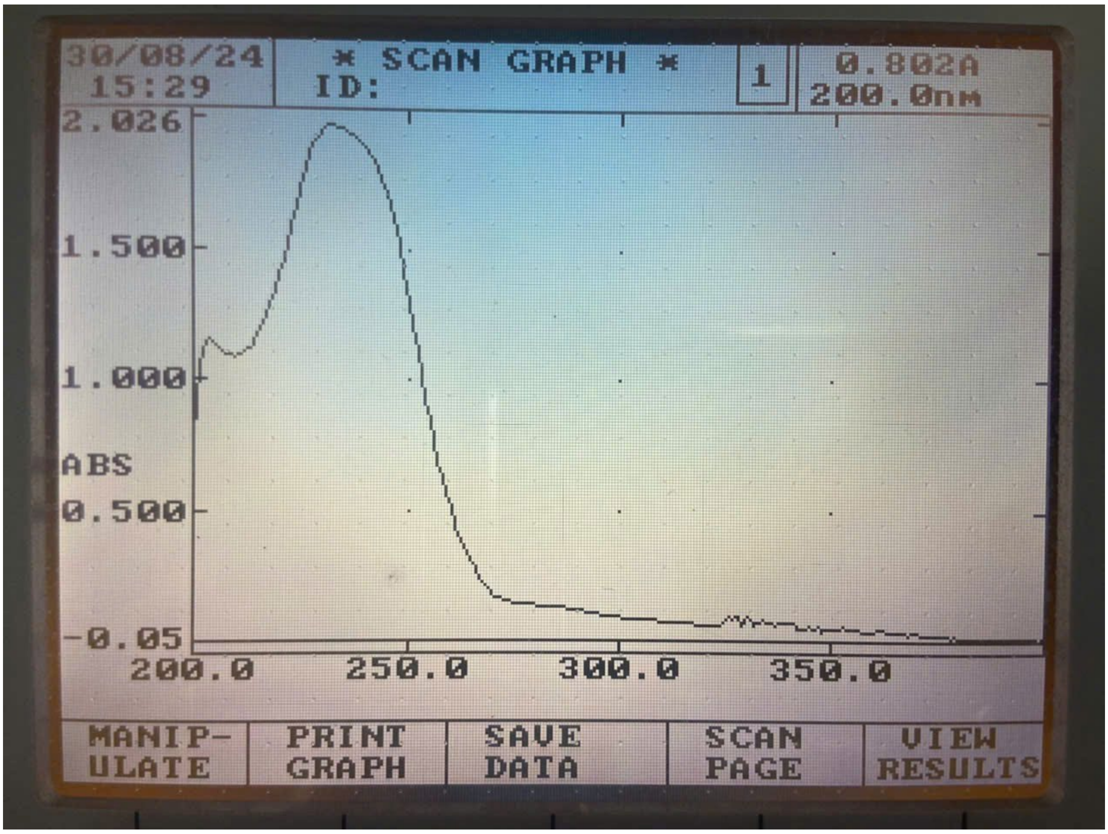

Figure S71. IR (ATR) spectrum of compound **7**

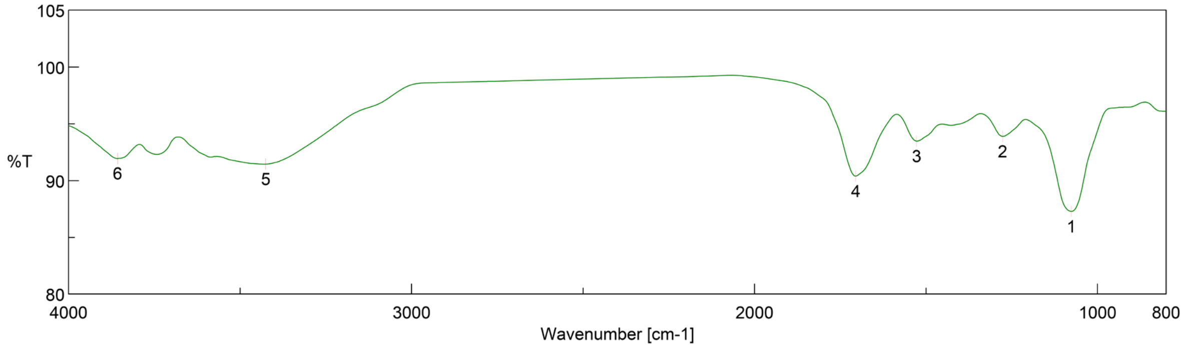

[Result of Peak Picking]

No.	Position	Intensity	No.	Position	Intensity	No.	Position	Intensity
1	1076.08	87.2785	2	1276.65	93.8814	3	1527.35	93.4629
4	1704.76	90.3848	5	3424.96	91.4303	6	3856.93	91.9336

Figure S72. ^{1}H-NMR spectrum of compound **8** (600 MHz, CD_3OD)

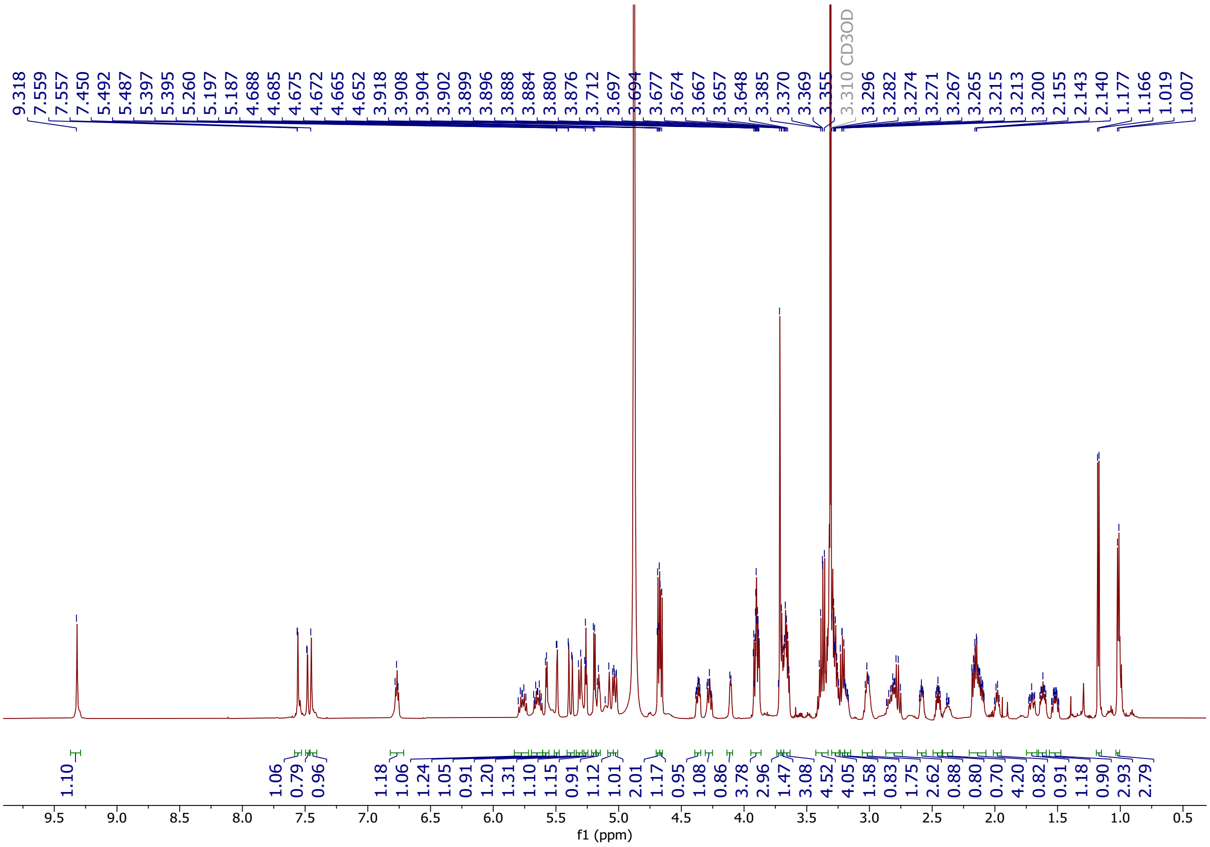

Figure S73. ^{13}C-NMR spectrum of compound **8** (125 MHz, CD_3OD)

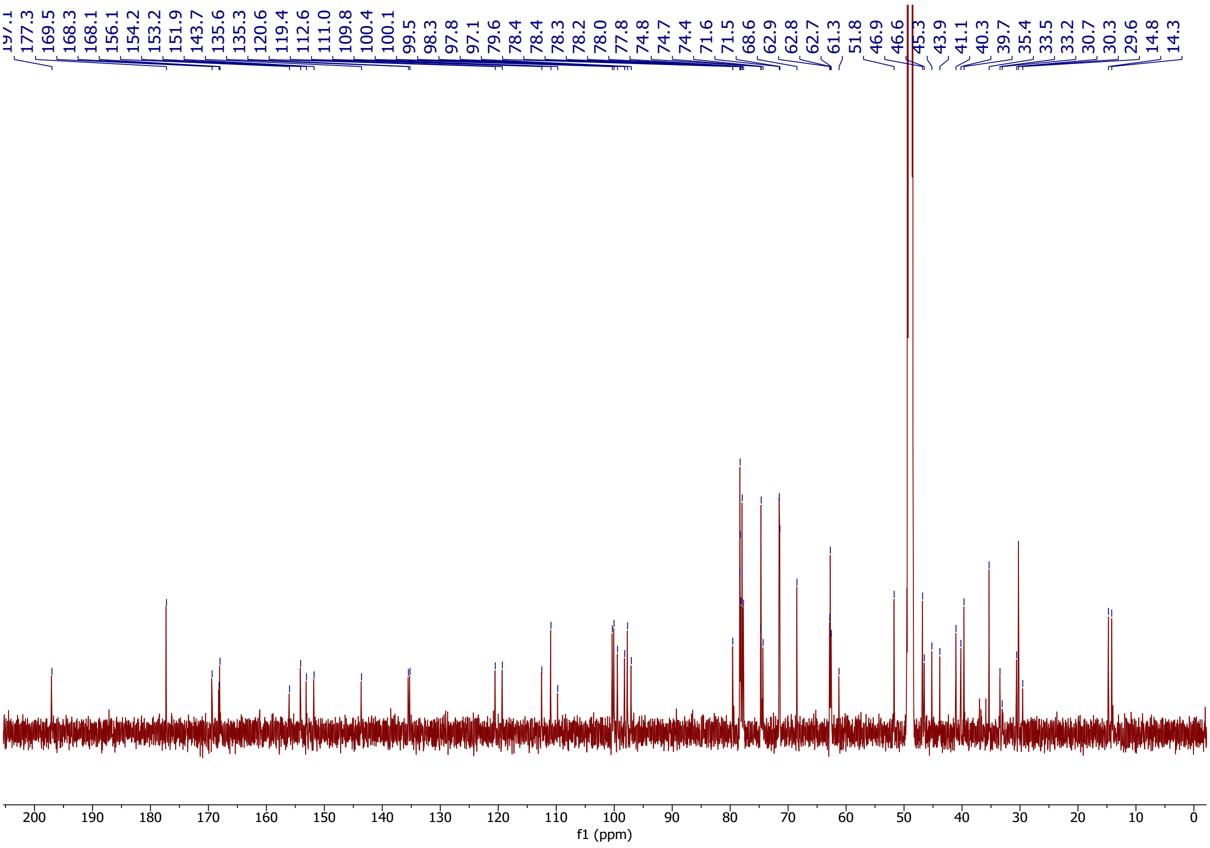

Figure S74. DEPT-NMR spectrum of compound **8** (125 MHz, CD_3OD)

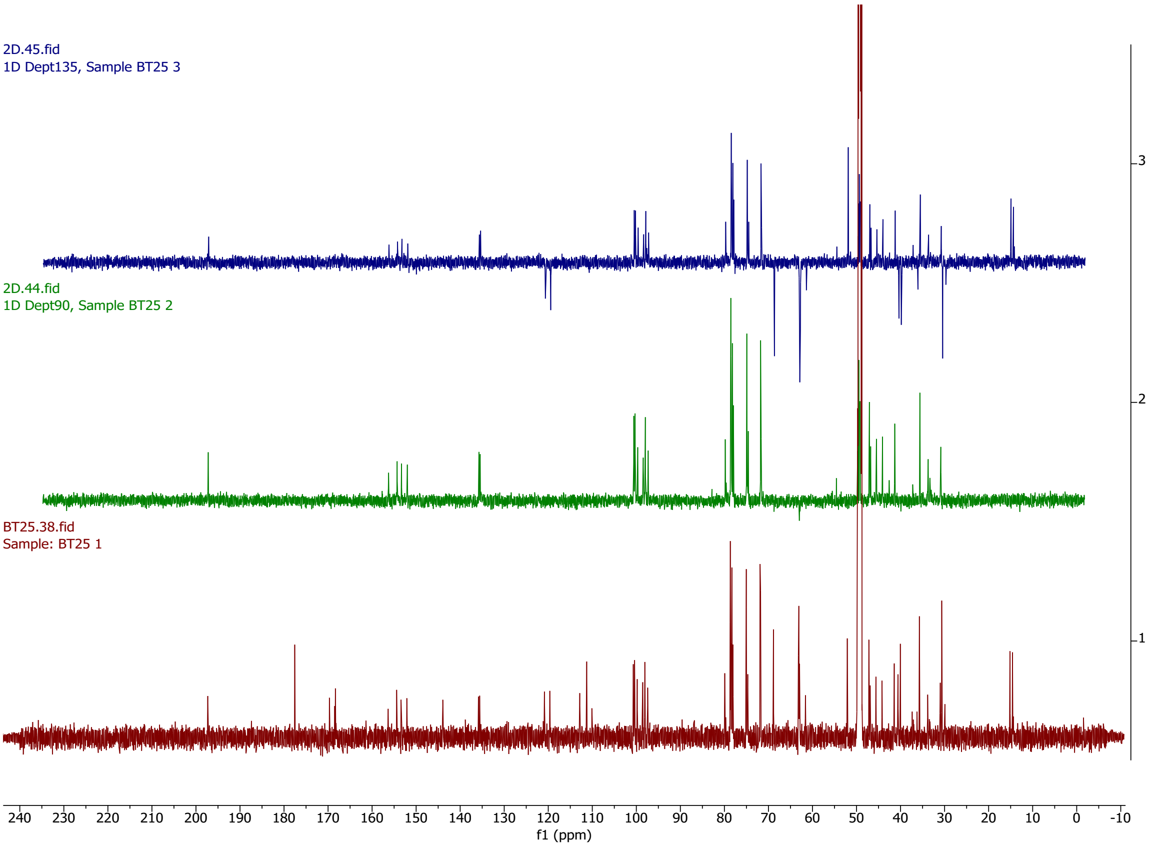

Figure S75. HSQC spectrum of compound **8** (600 MHz, CD_3OD)

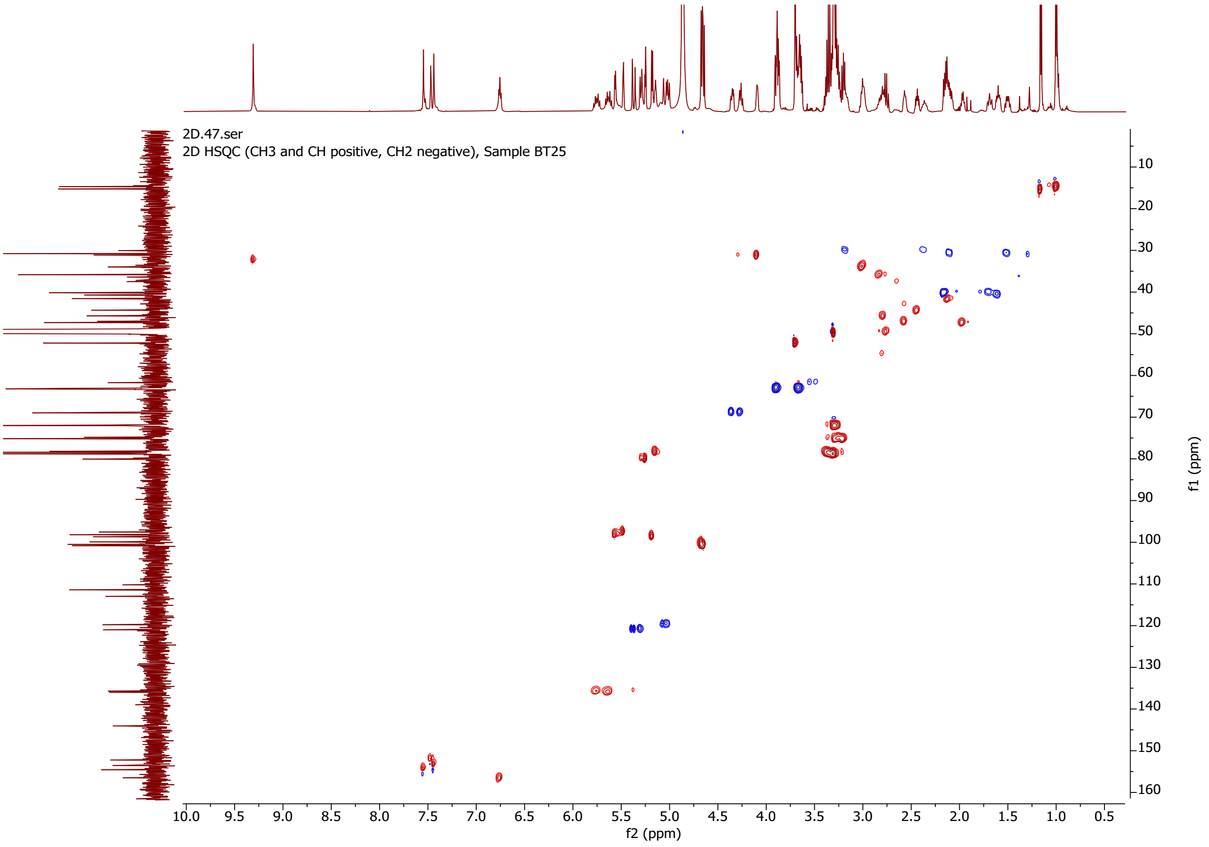

Figure S76. COSY spectrum of compound **8** (600 MHz, CD_3OD)

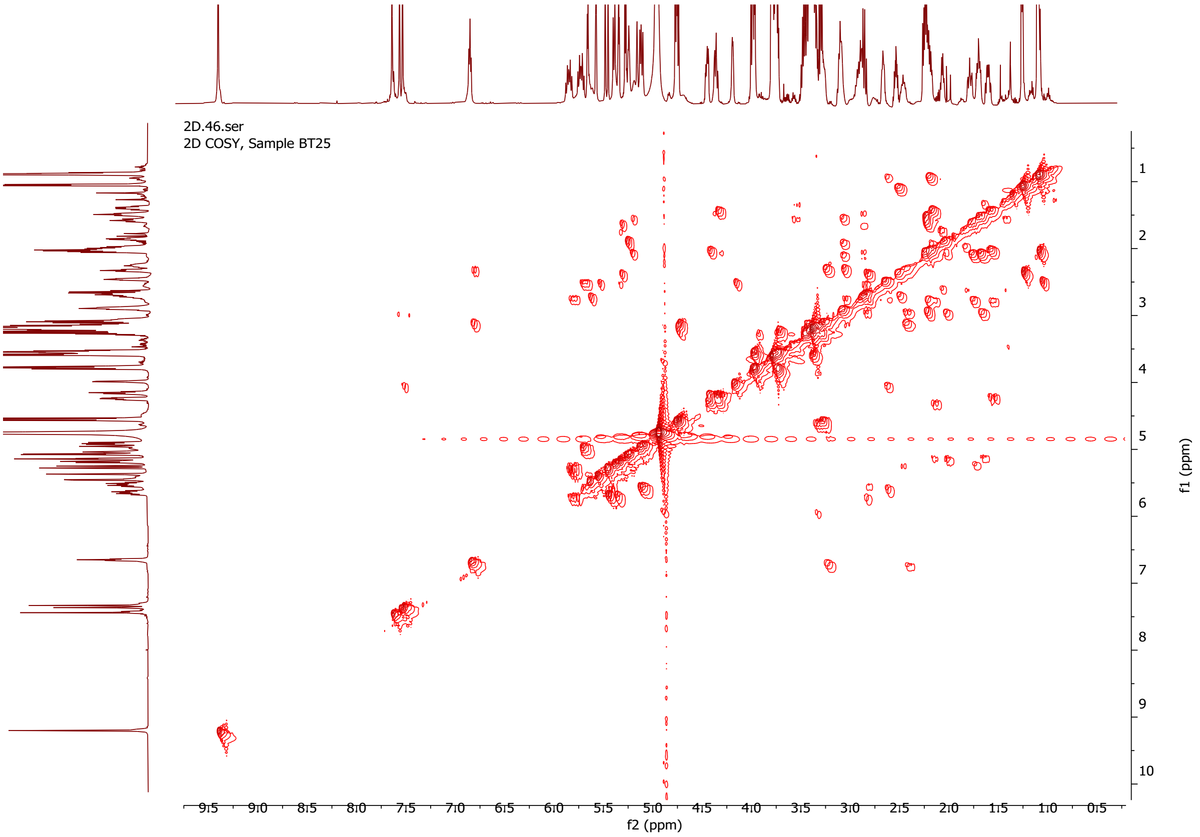

Figure S77. HMBC spectrum of compound **8** (600 MHz, CD_3OD)

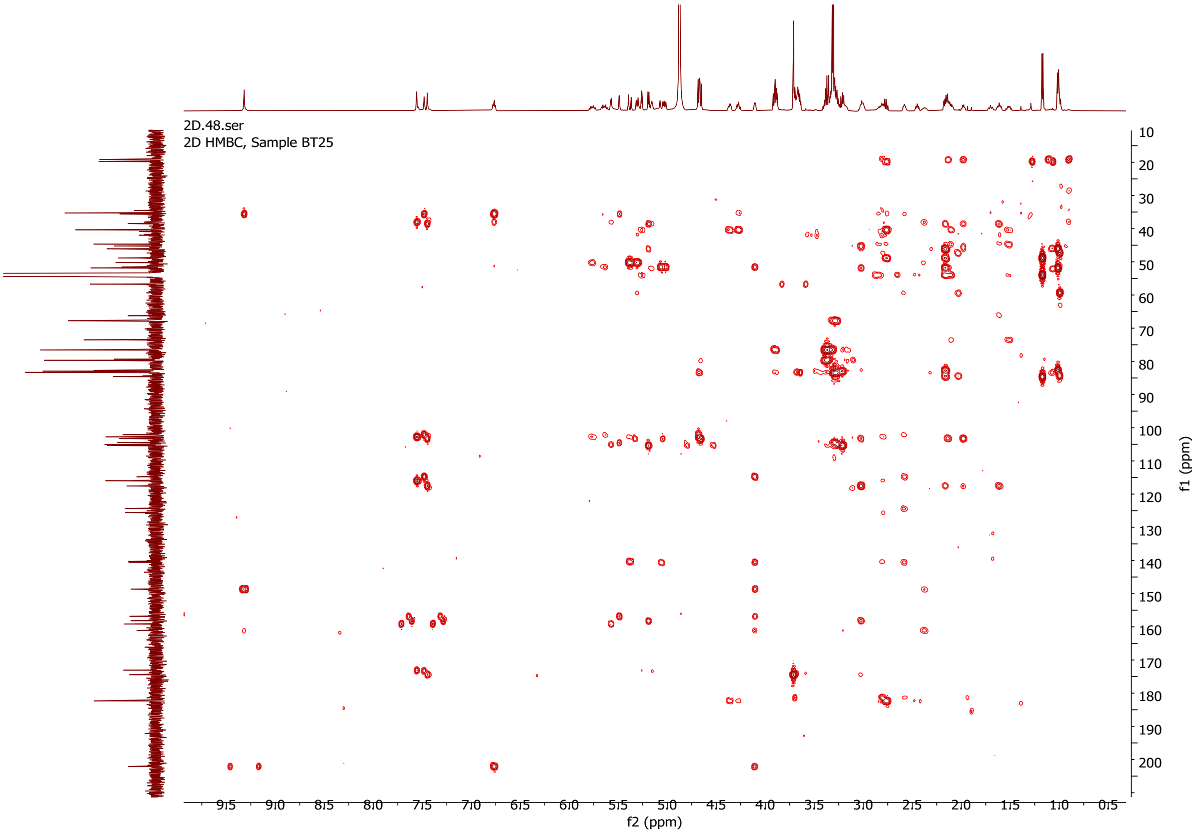

Figure S78. ROESY spectrum of compound **8** (600 MHz, CD_3OD)

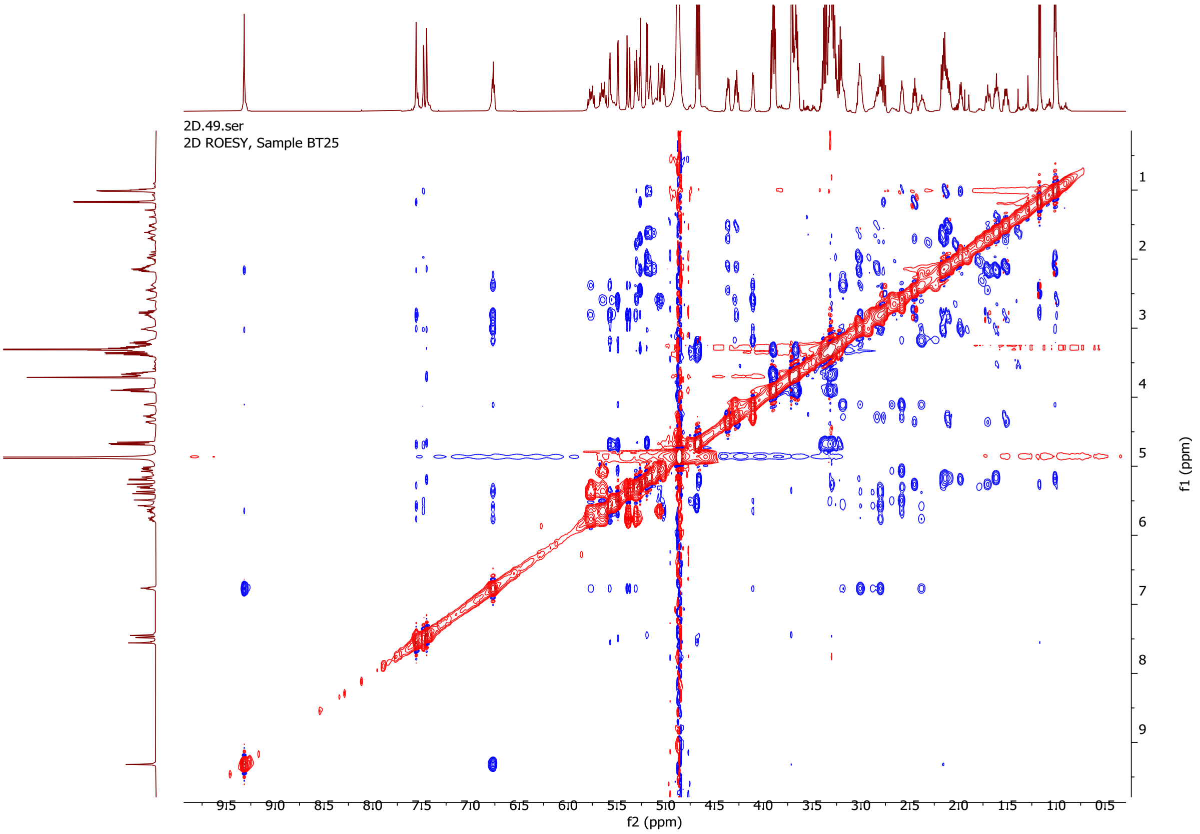

Figure S79. HR-ESI-MS data of compound **8**

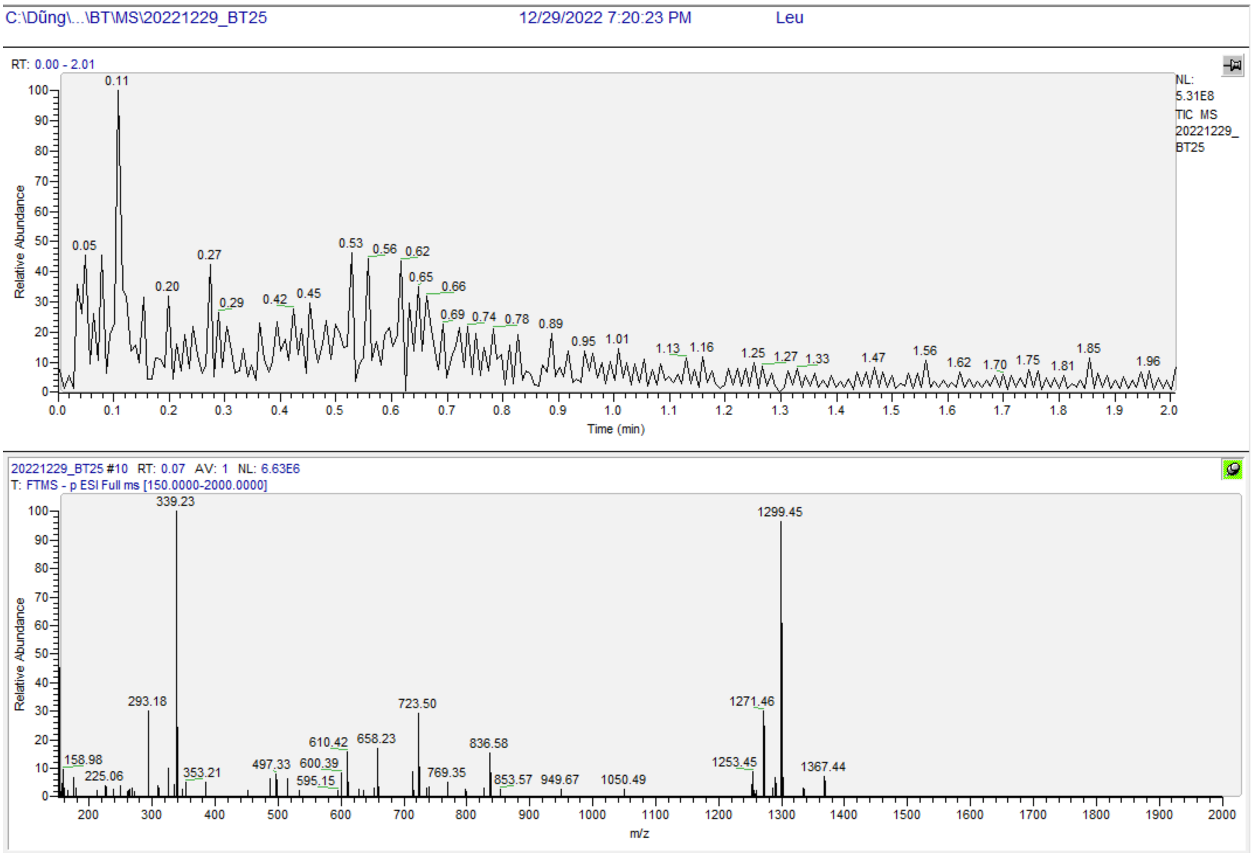

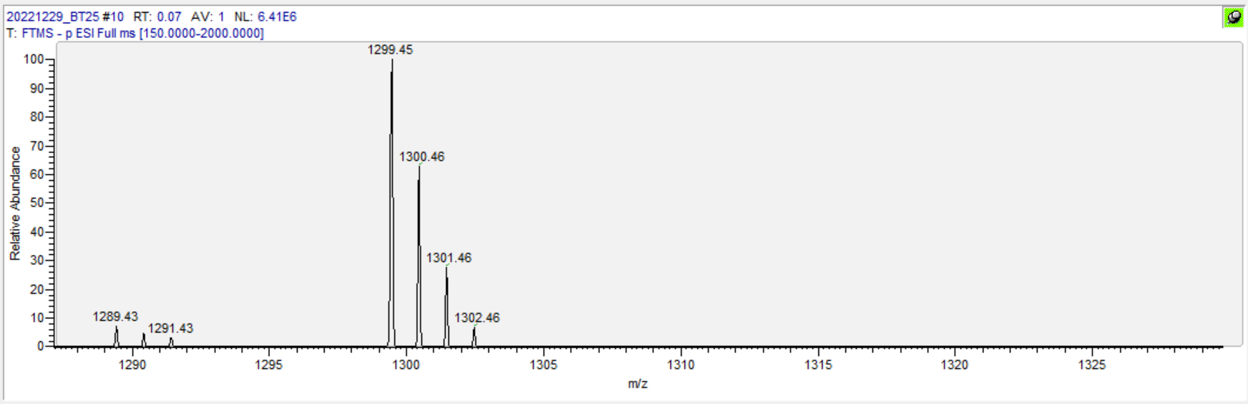

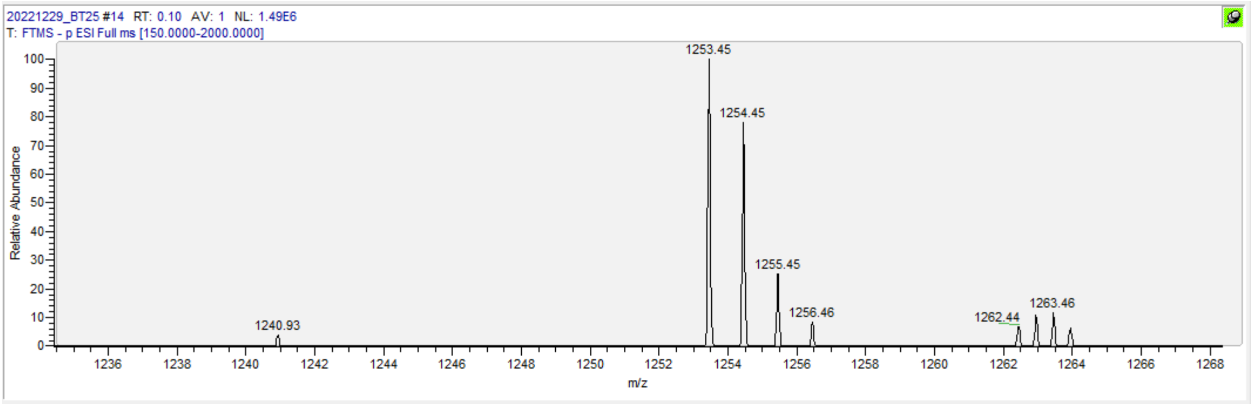

Elemental composition

Single mass

Mass: 1253.44897

Max. results 10

Calculate

Idx	Formula	RDB	Delta ppm
1	$C_{58}H_{77}O_{30}$	20.5	-0.357

Elemental composition

Single mass

Mass: 1299.45386

Max. results 10

Calculate

Idx	Formula	RDB	Delta ppm
1	$C_{59}H_{79}O_{32}$	20.5	-0.797

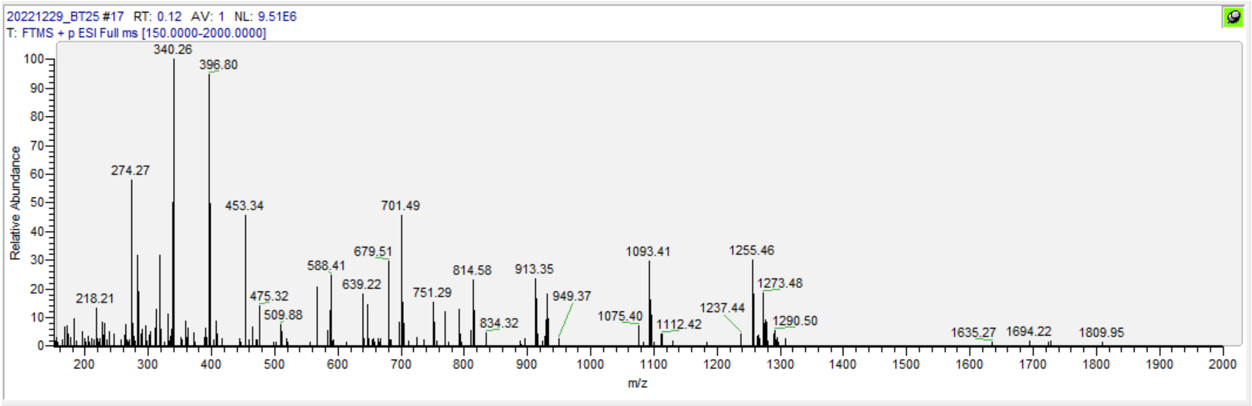

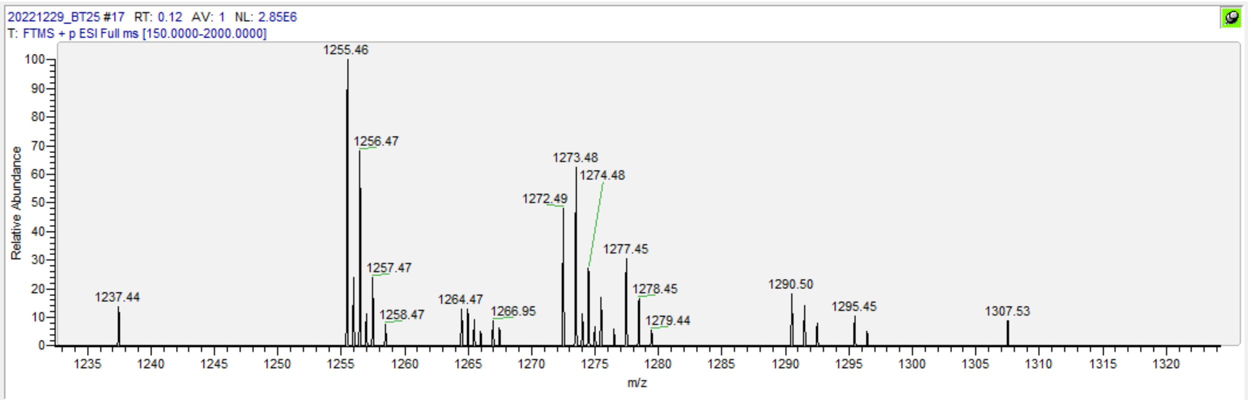

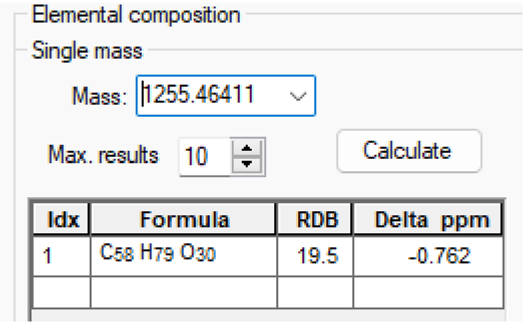
Elemental composition
Single mass
Mass: 1255.46411
Max. results 10
Calculate

Idx	Formula	RDB	Delta ppm
1	C58 H79 O30	19.5	-0.762

Figure S80. UV spectrum (in MeOH) of compound **8**

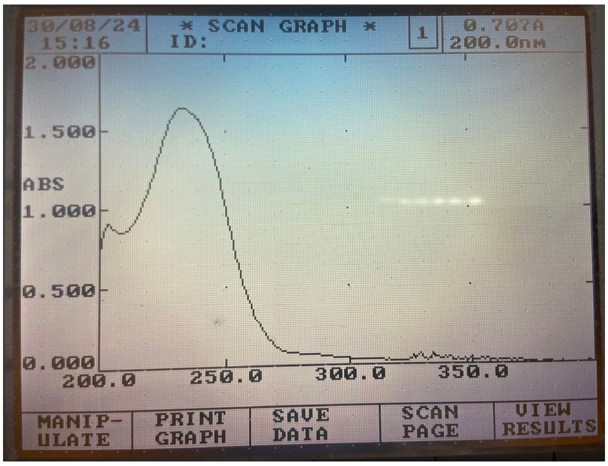

Figure S81. IR (ATR) spectrum of compound **8**

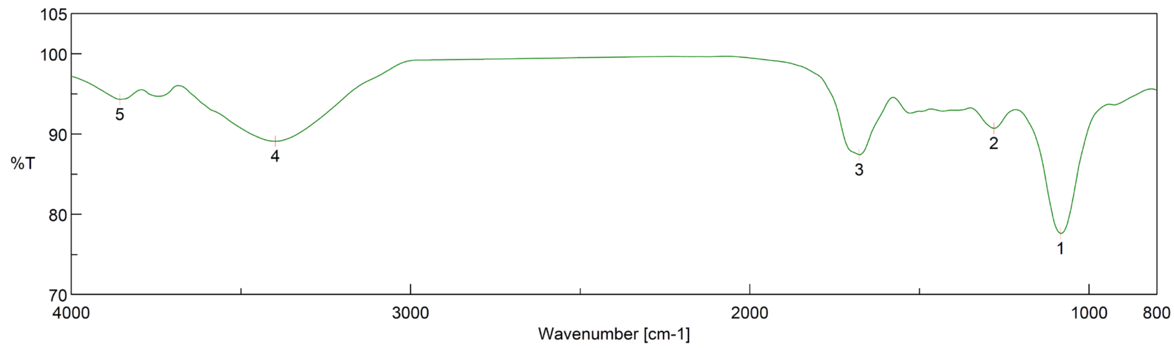

[Result of Peak Picking]

No.	Position	Intensity	No.	Position	Intensity	No.	Position	Intensity
1	1083.8	77.6119	2	1280.5	90.6918	3	1677.77	87.4178
4	3397.96	89.0859	5	3856.93	94.3105			

Figure S82. ^{1}H-NMR spectrum of compound **9** (600 MHz, CD_3OD)

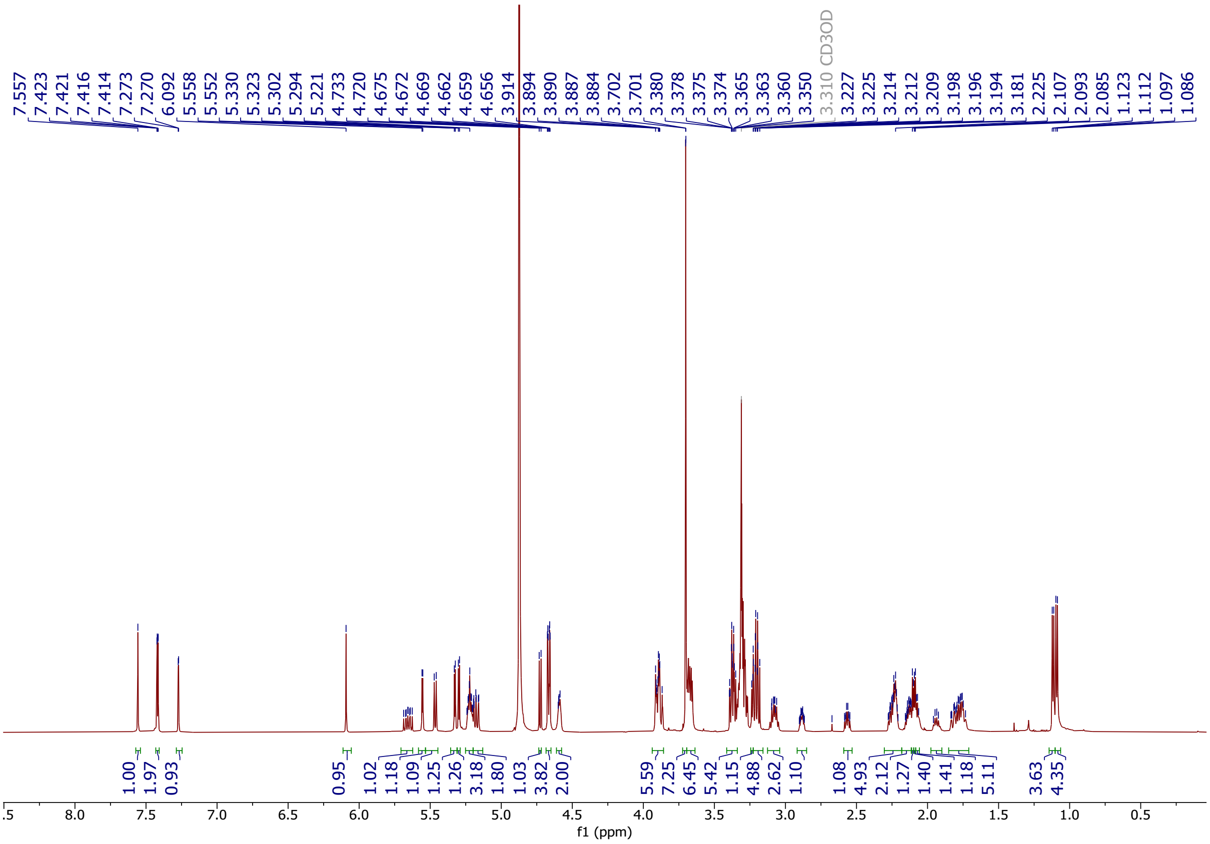

Figure S83. ^{13}C-NMR spectrum of compound **9** (125 MHz, CD_3OD)

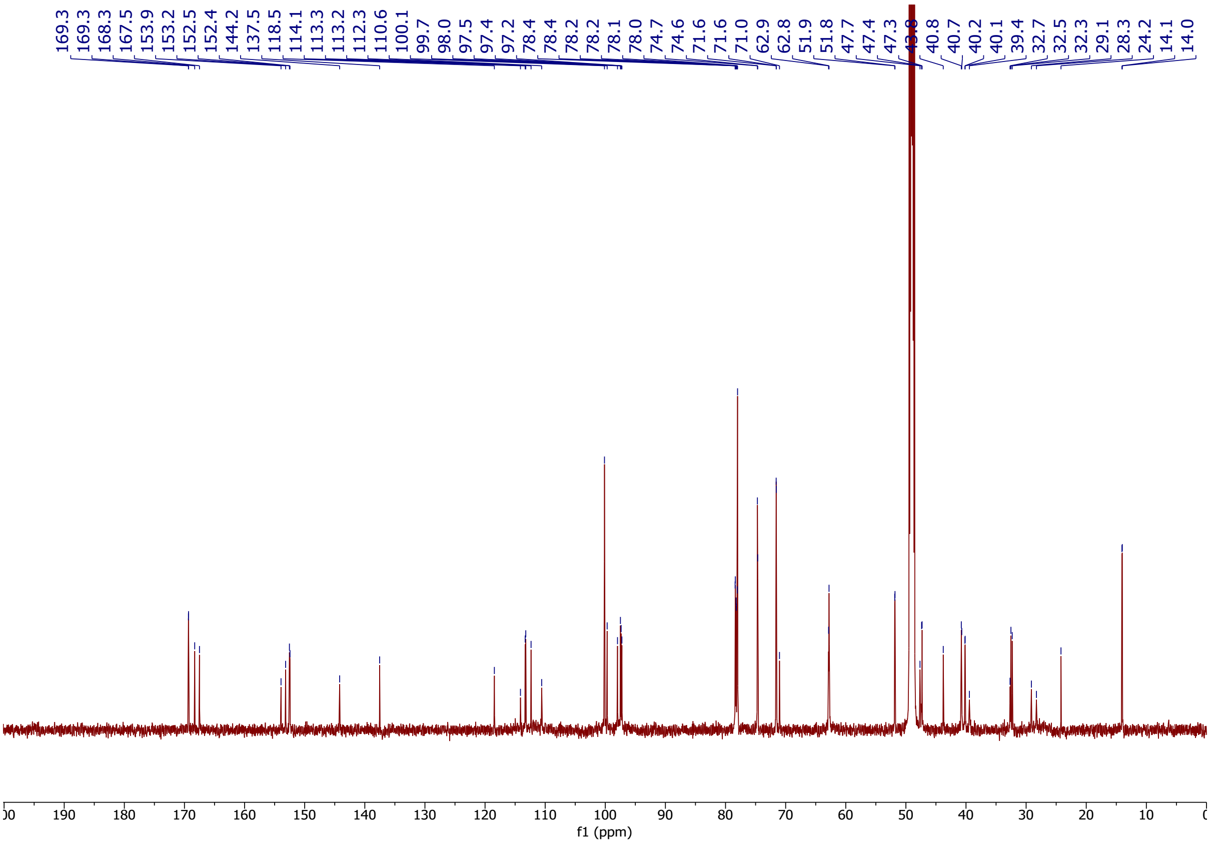

Figure S84. DEPT-NMR spectrum of compound **9** (125 MHz, CD_3OD)

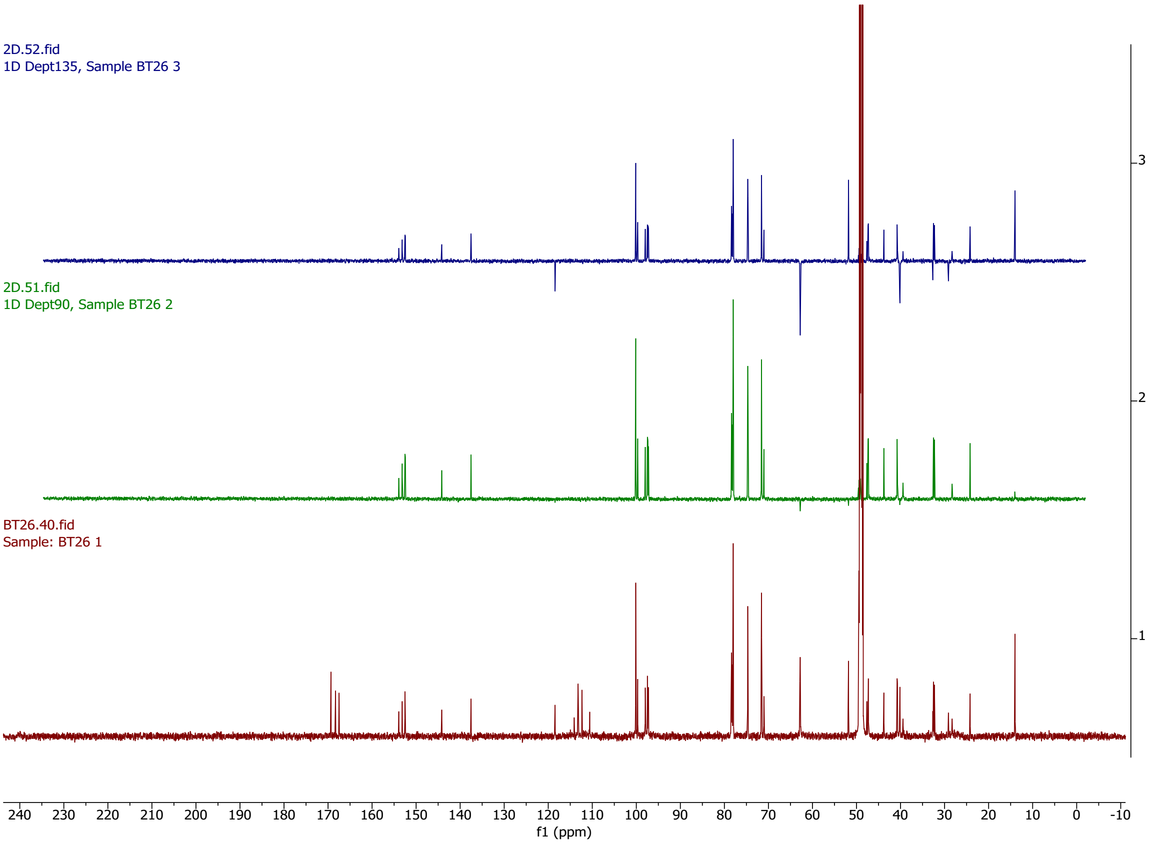

Figure S85. HSQC spectrum of compound **9** (600 MHz, CD_3OD)

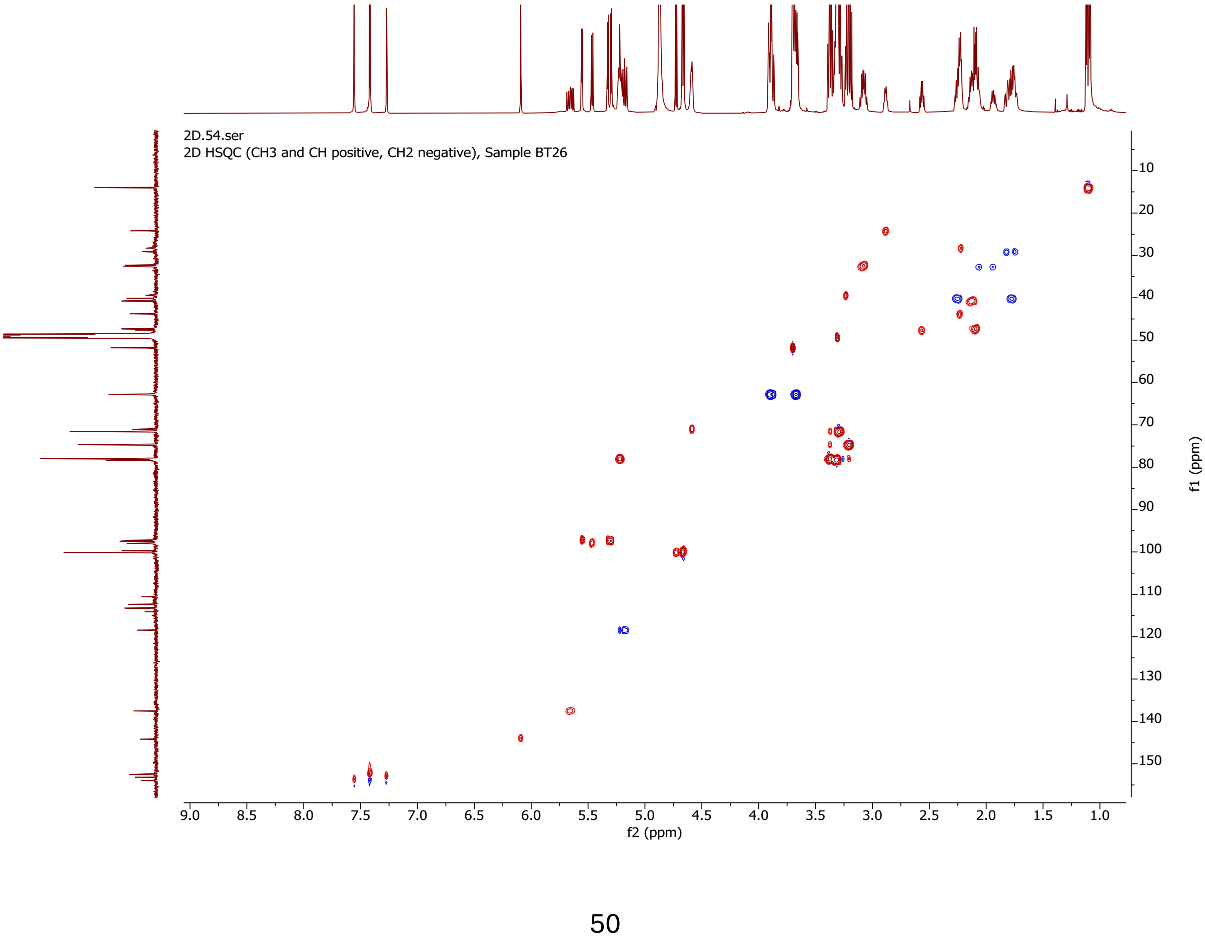

Figure S86. COSY spectrum of compound **9** (600 MHz, CD_3OD)

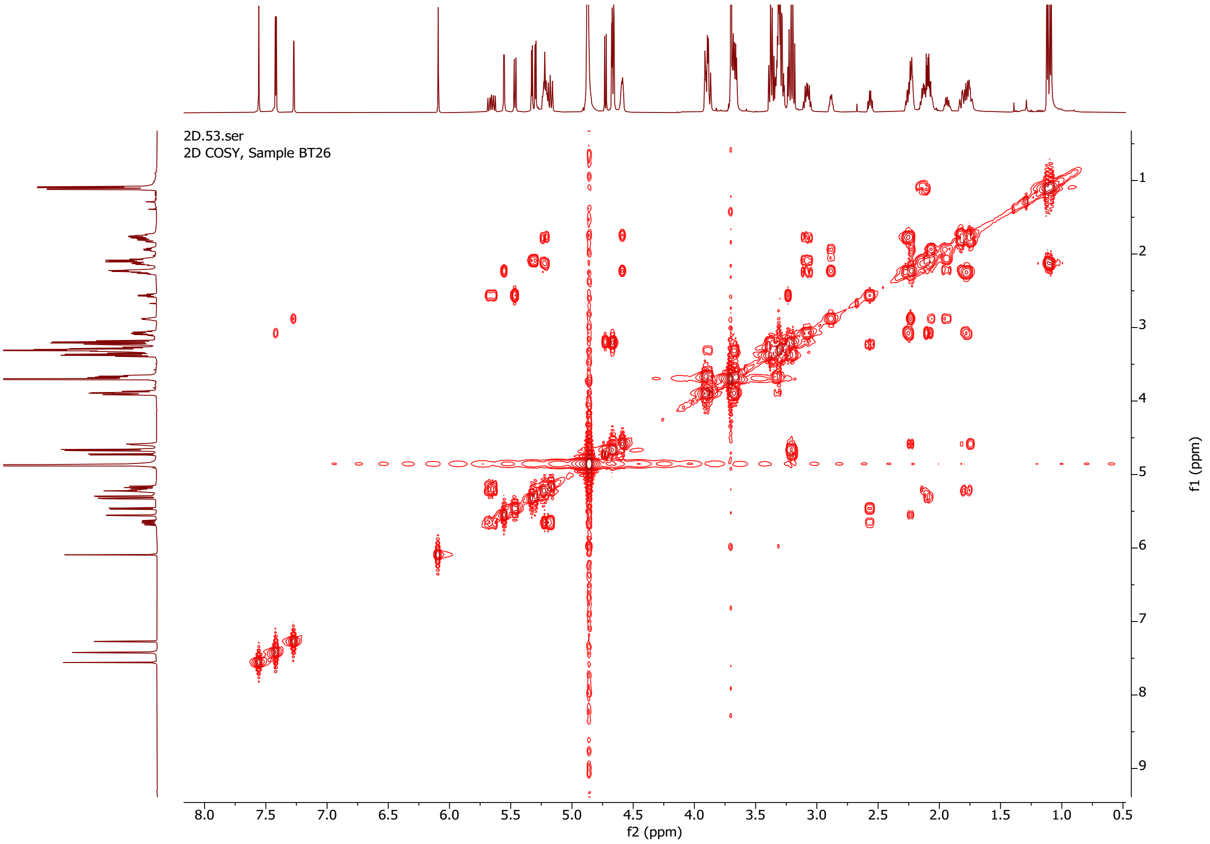

Figure S87. HMBC spectrum of compound **9** (600 MHz, CD_3OD)

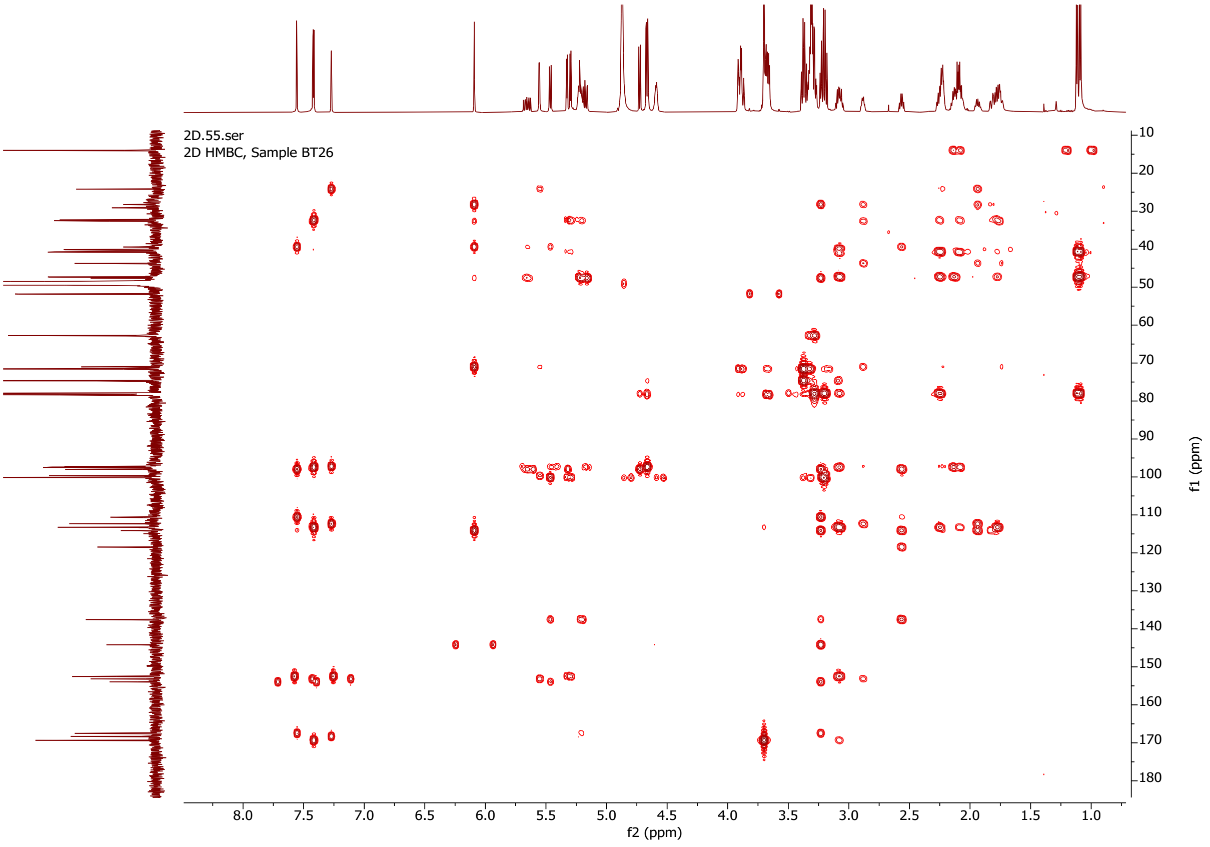

Figure S88. ROESY spectrum of compound **9** (600 MHz, CD_3OD)

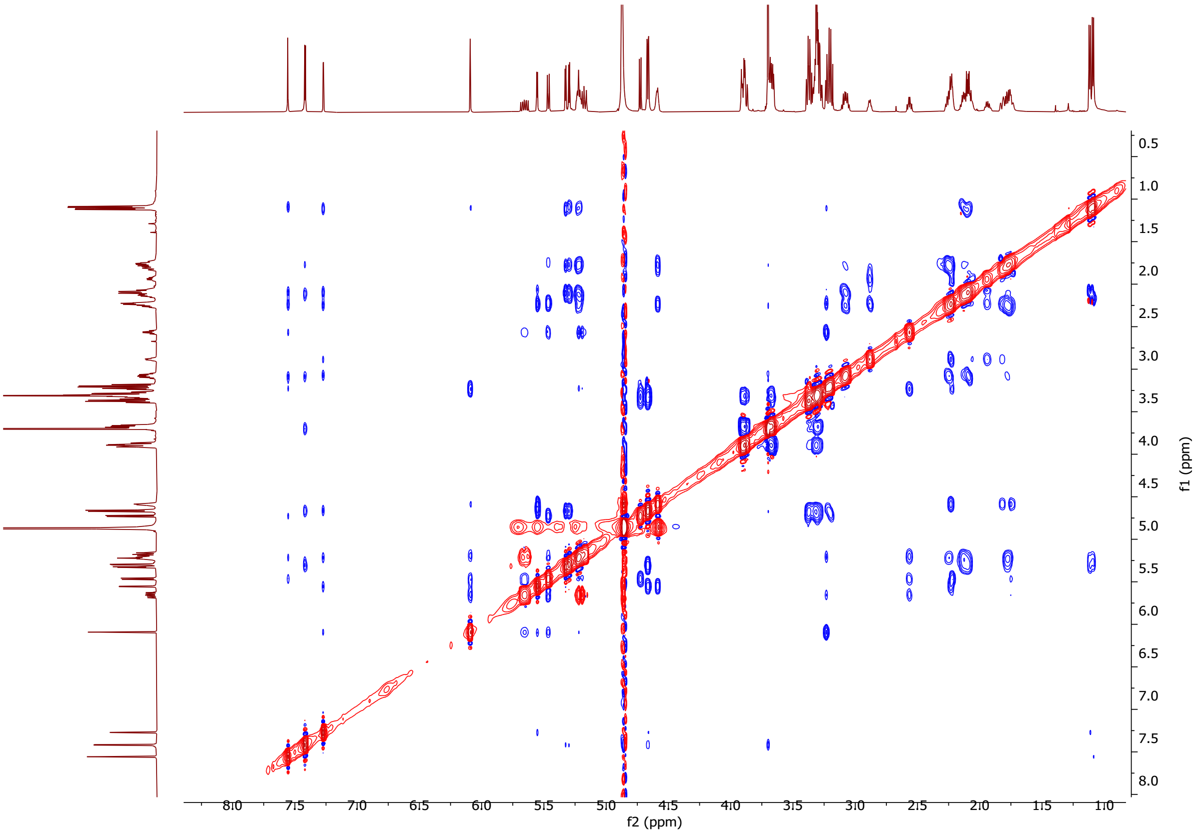

Figure S89. HR-ESI-MS data of compound **9**

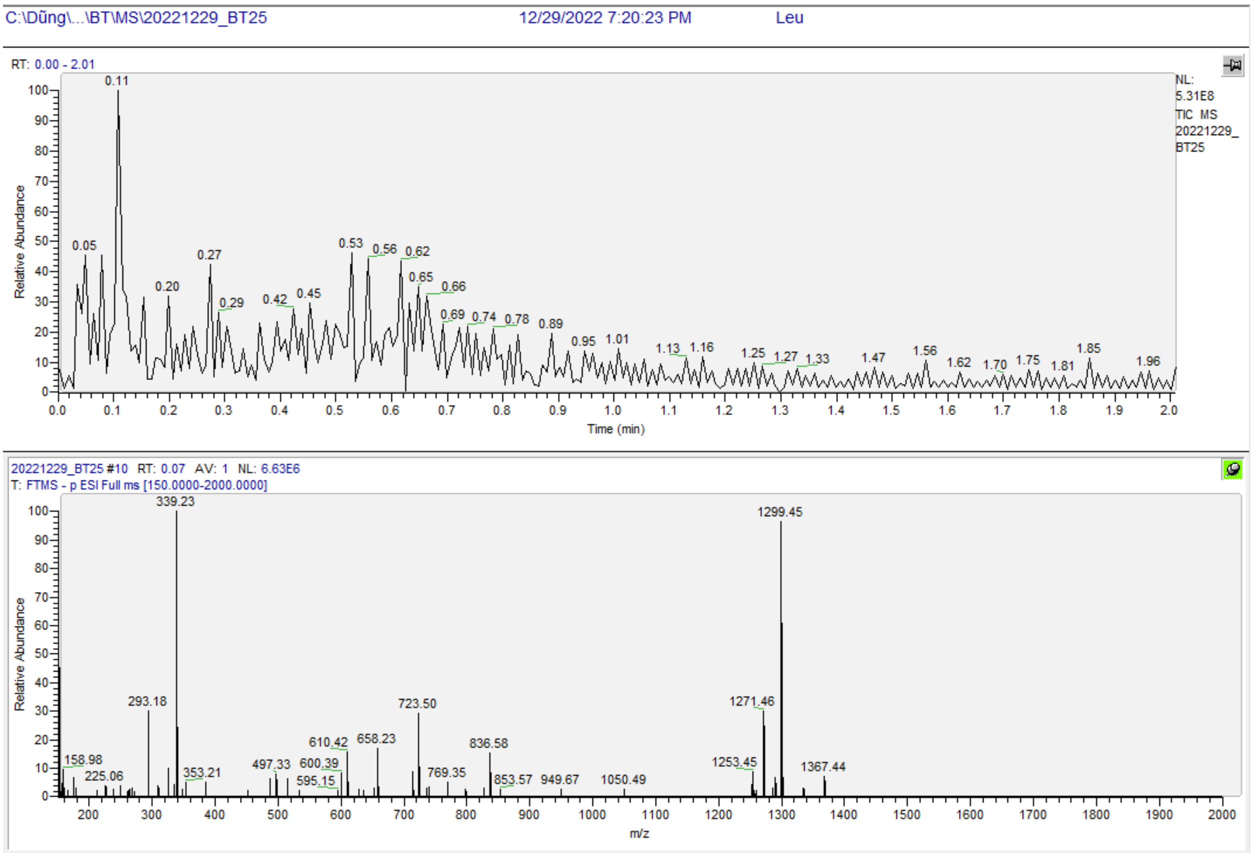

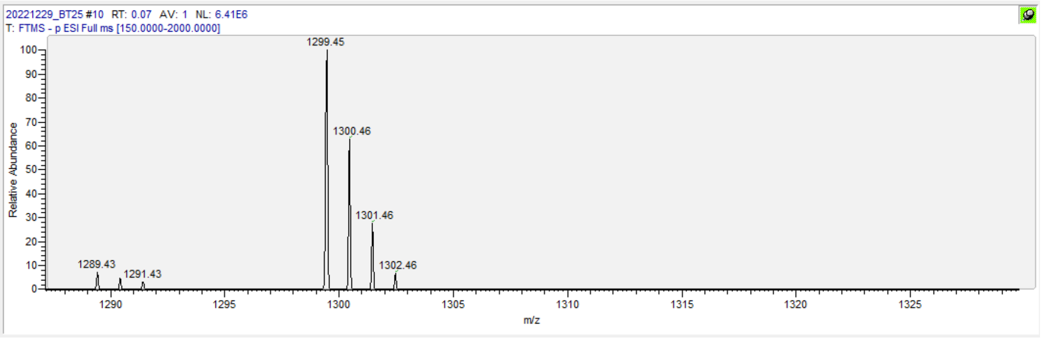

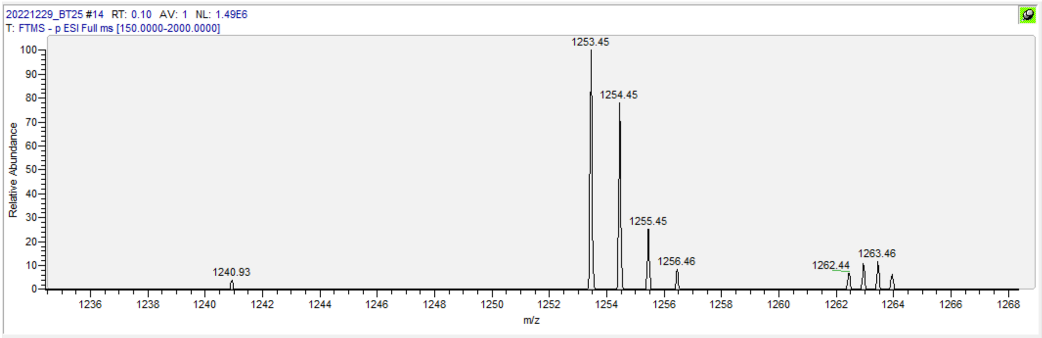

Elemental composition

Single mass

Mass: 1299.45386

Max. results 10 Calculate

Idx	Formula	RDB	Delta ppm
1	$C_{59}H_{79}O_{32}$	20.5	-0.797

Elemental composition

Single mass

Mass: 1253.44897

Max. results 10 Calculate

Idx	Formula	RDB	Delta ppm
1	$C_{58}H_{77}O_{30}$	20.5	-0.357

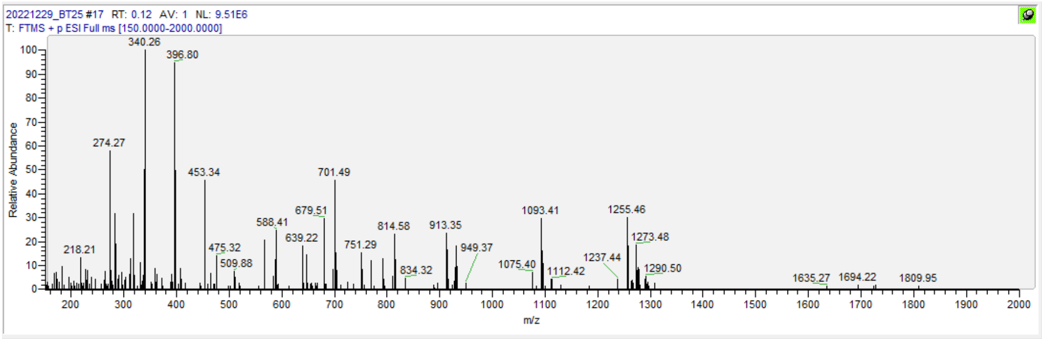

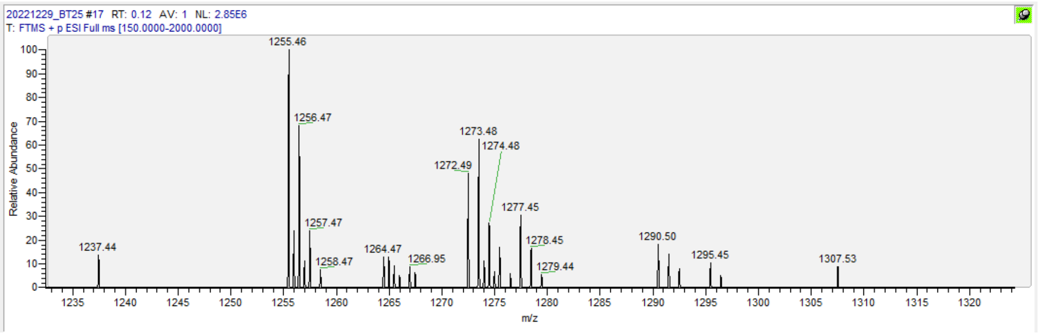

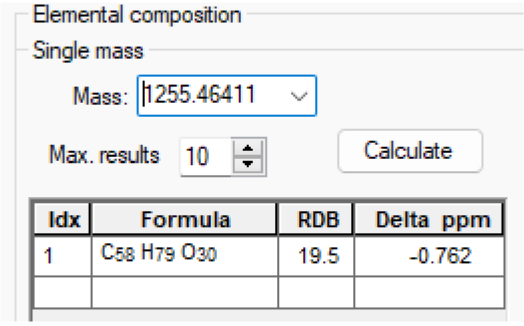

Figure S90. UV spectrum (in MeOH) of compound **9**

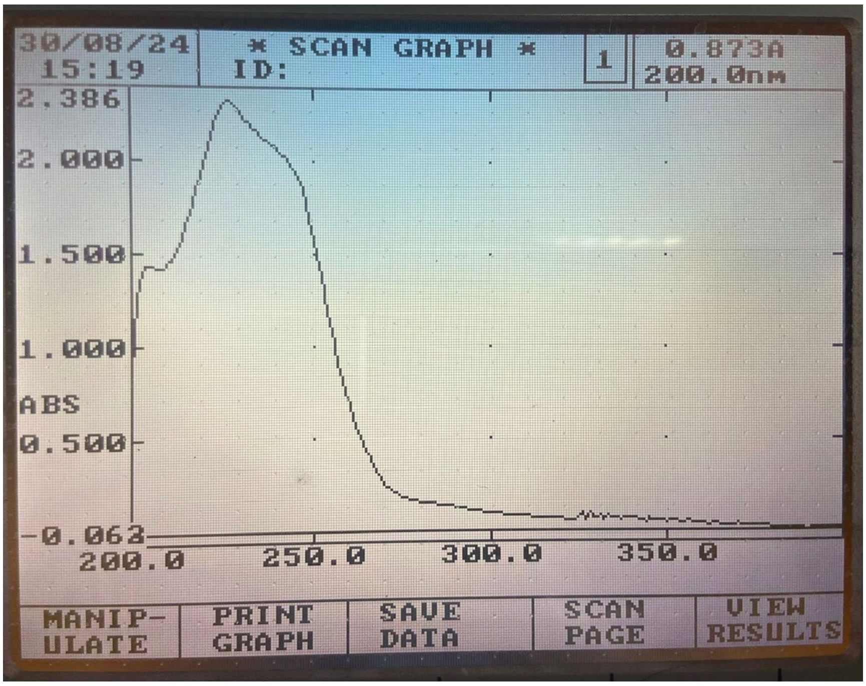

Figure S91. IR (ATR) spectrum of compound **9**

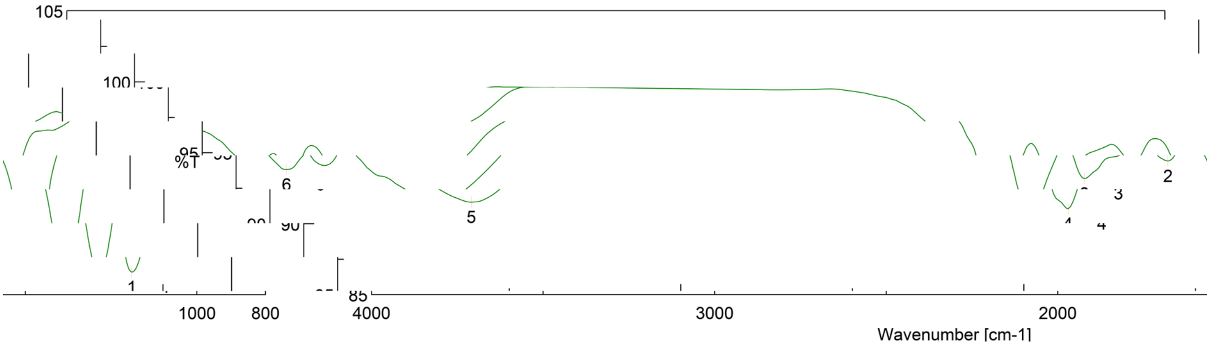

[Result of Peak Picking]

No.	Position	Intensity	No.	Position	Intensity	No.	Position	Intensity
1	1091.51	86.6023	2	1284.36	94.4109	3	1527.35	93.1742
4	1673.91	91.0567	5	3413.39	91.5097	6	3853.08	93.814

Figure S92. ^{1}H-NMR spectrum of compound **10** (600 MHz, CD_3OD)

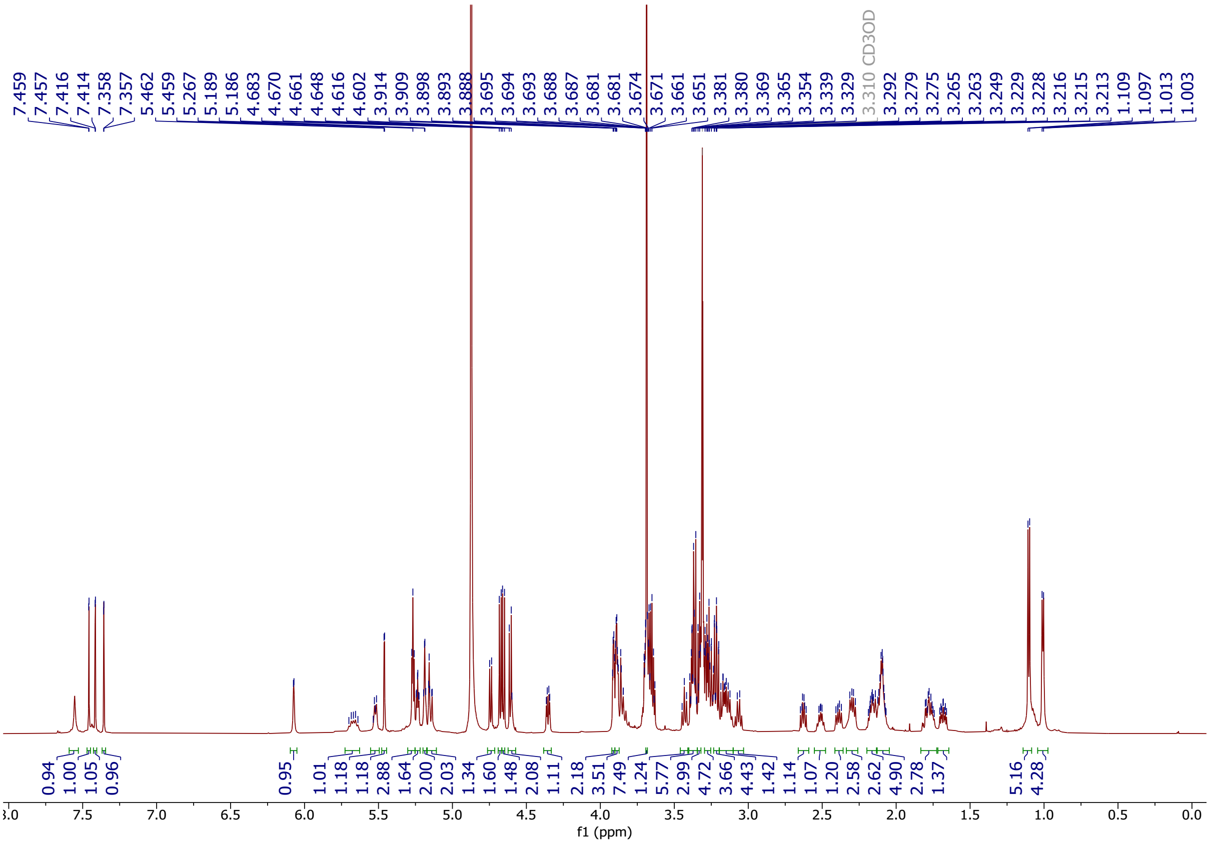

Figure S93. ^{13}C-NMR spectrum of compound **10** (600 MHz, CD_3OD)

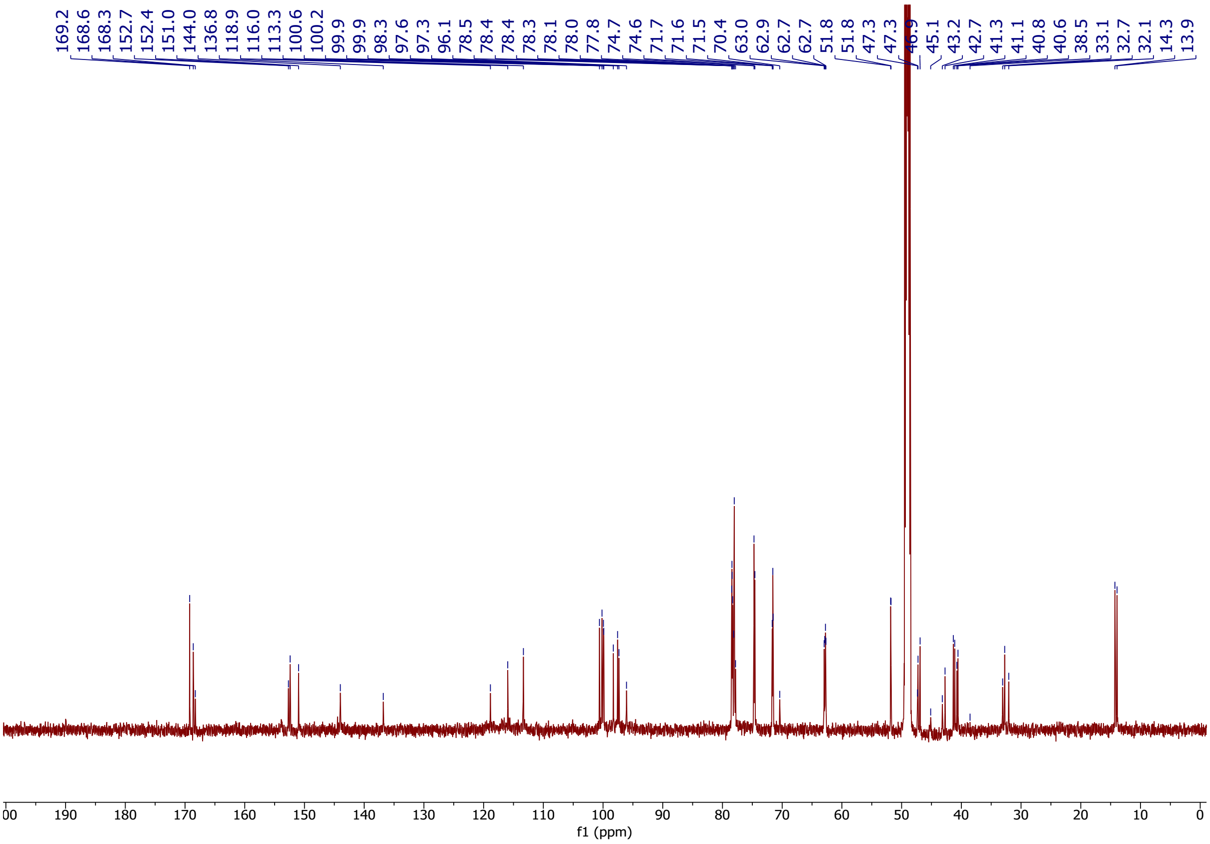

Figure S94. DEPT-NMR spectrum of compound **10** (600 MHz, CD_3OD)

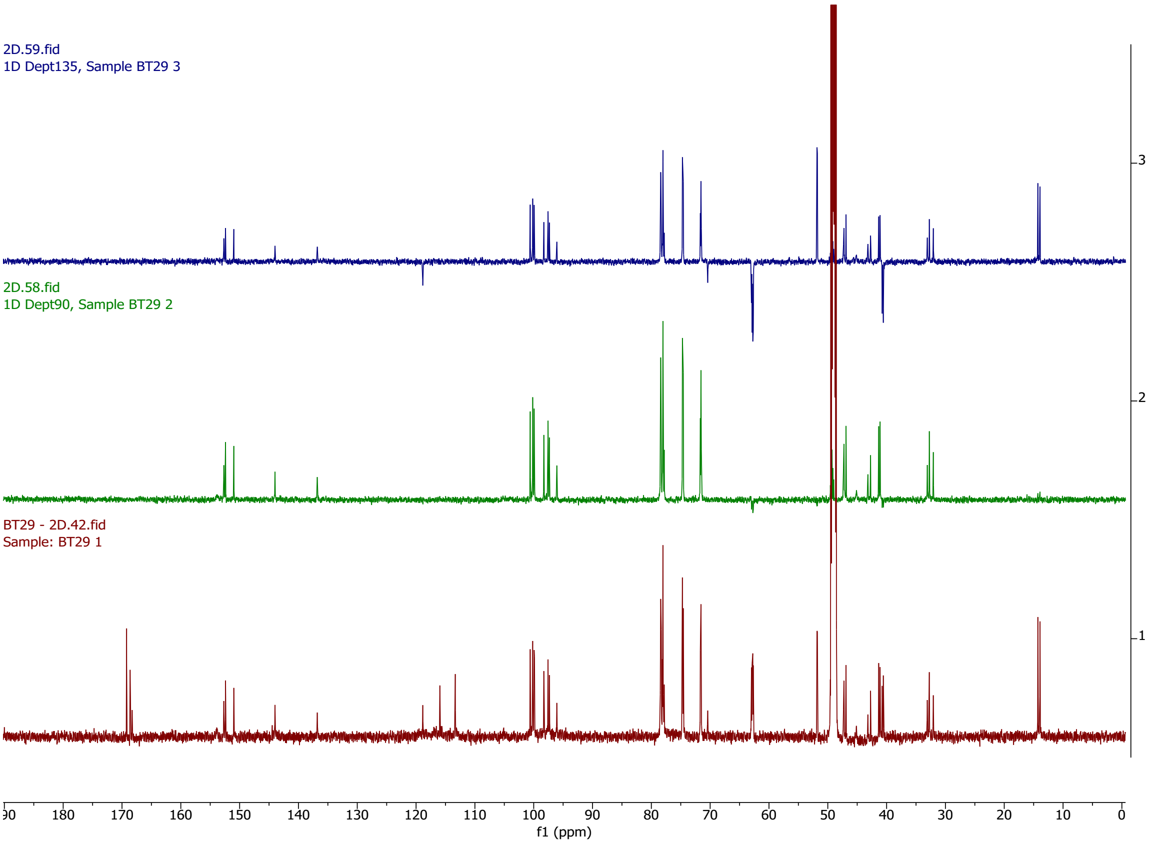

Figure S95. HSQC spectrum of compound **10** (600 MHz, CD_3OD)

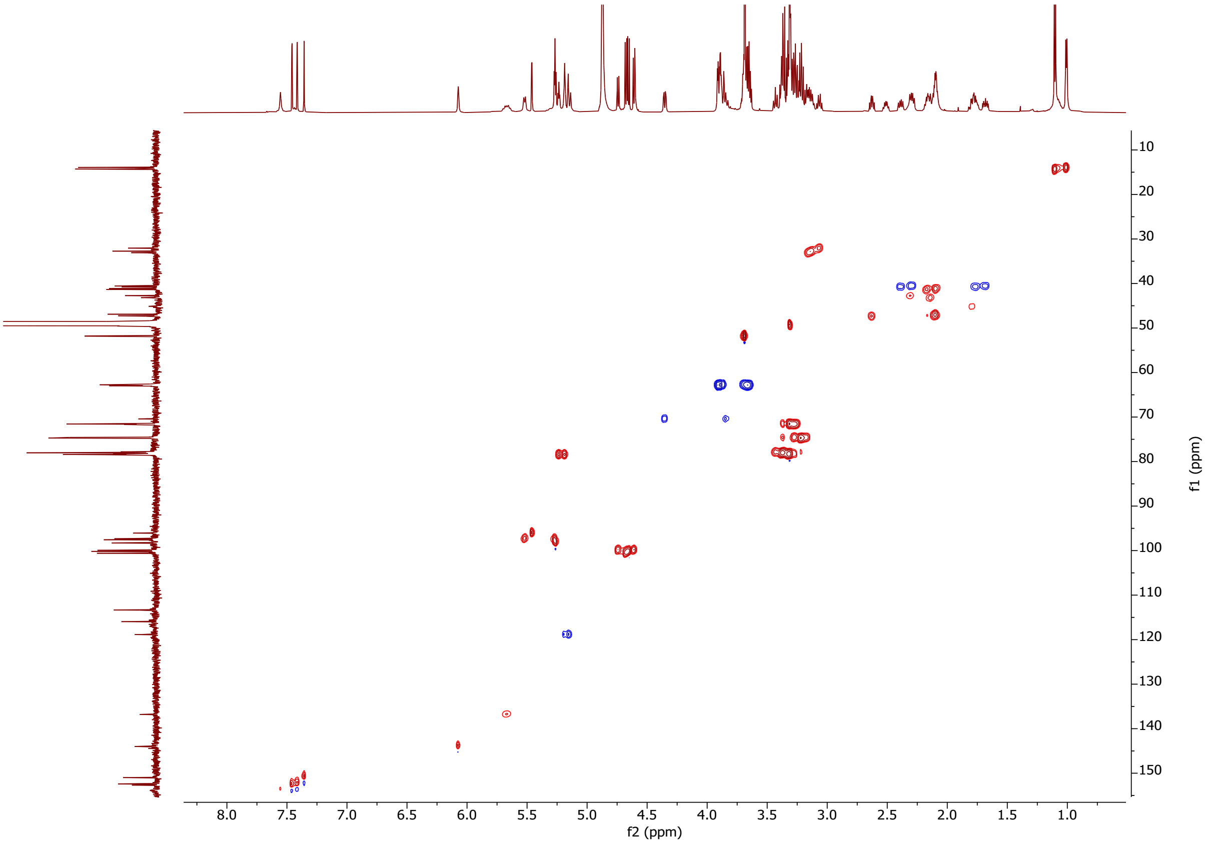

Figure S96. COSY spectrum of compound **10** (600 MHz, CD_3OD)

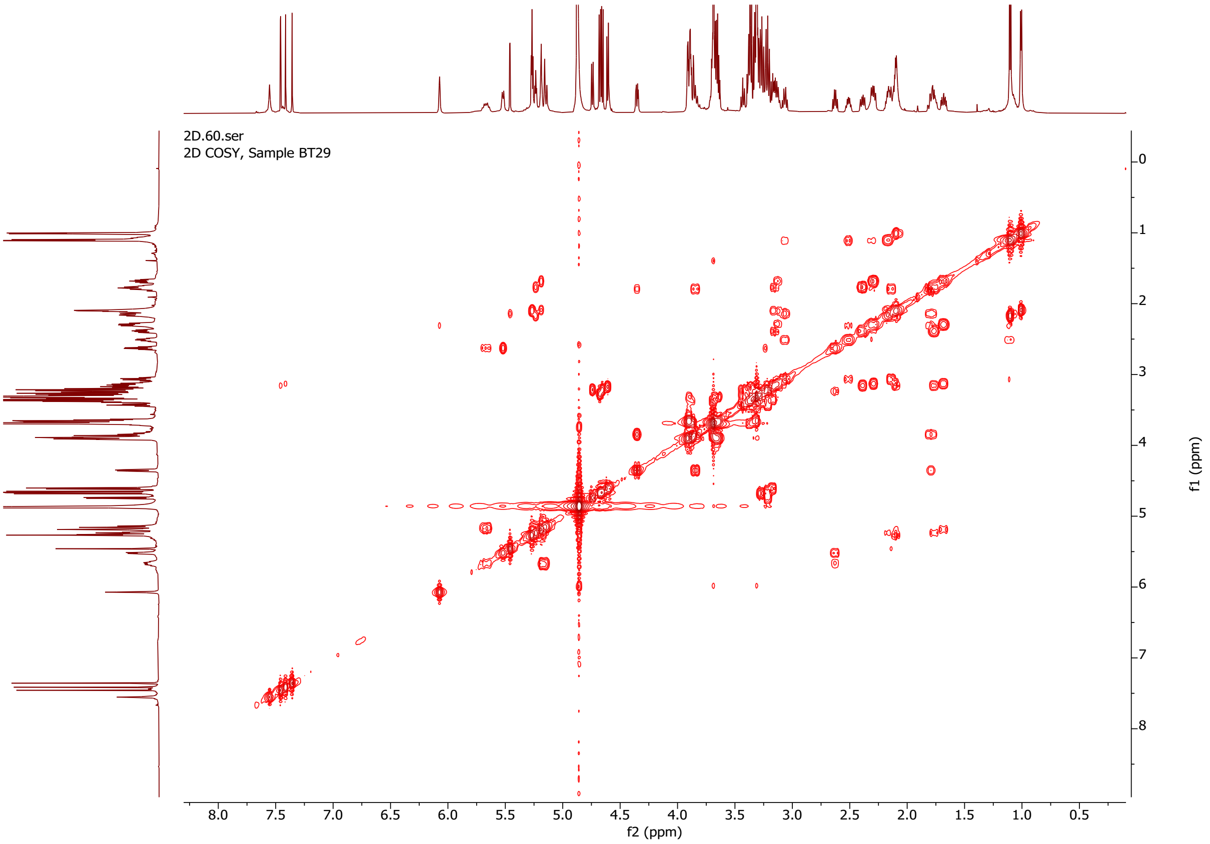

Figure S97. HMBC spectrum of compound **10** (600 MHz, CD_3OD)

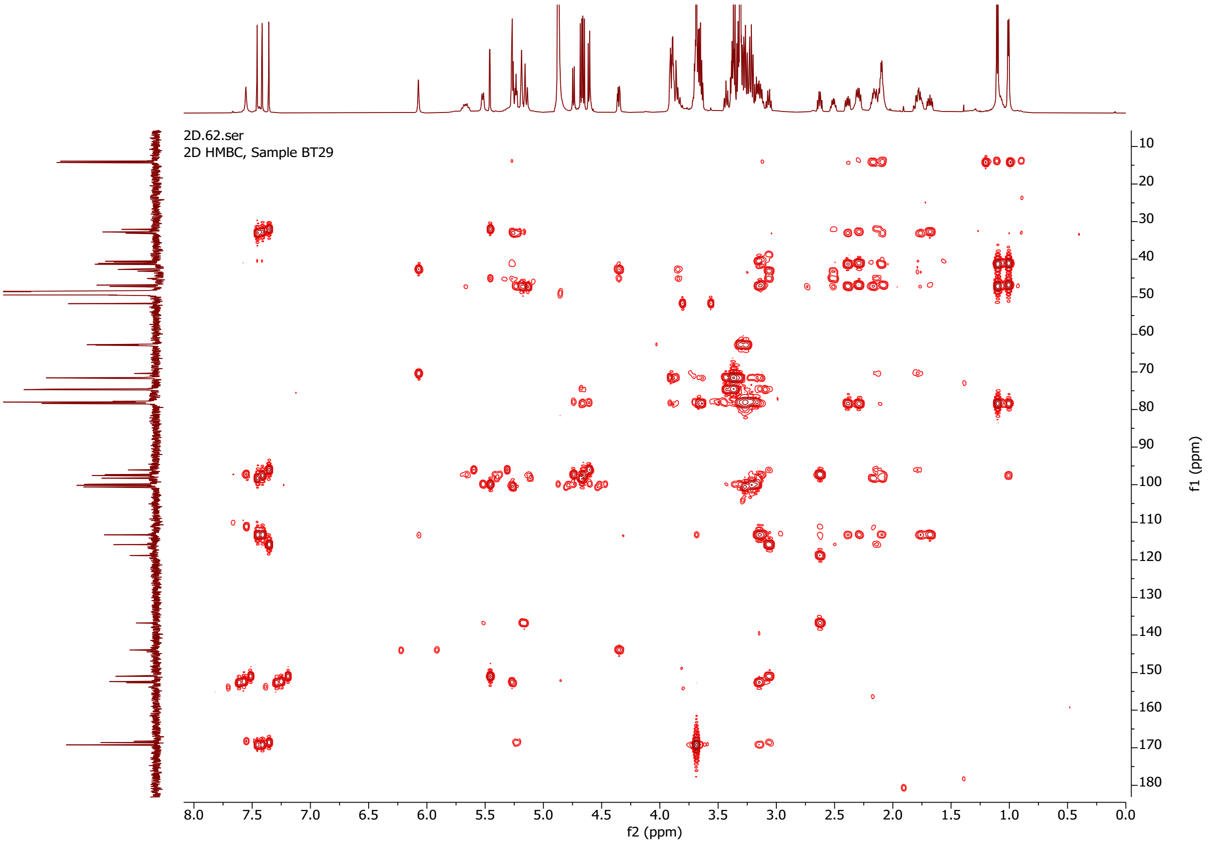

Figure S98. ROESY spectrum of compound **10** (600 MHz, CD_3OD)

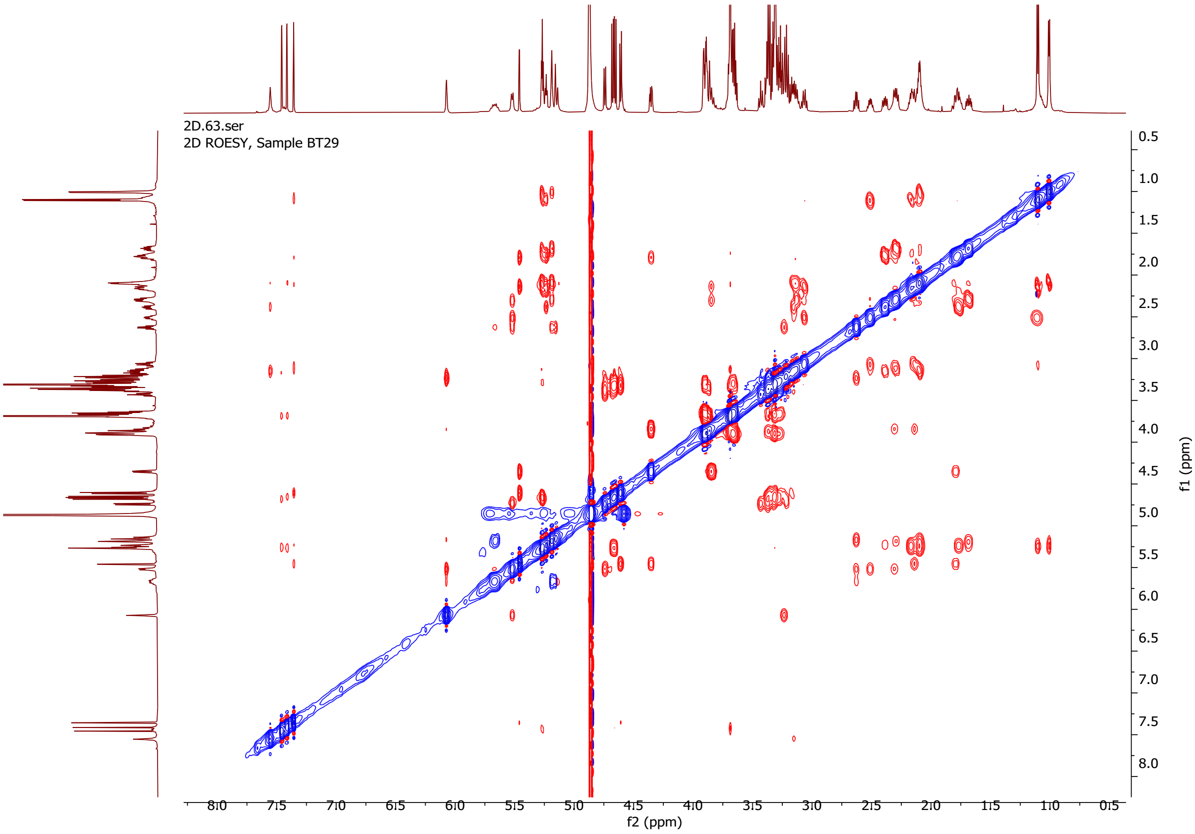

Figure S99. HR-ESI-MS data of compound **10**

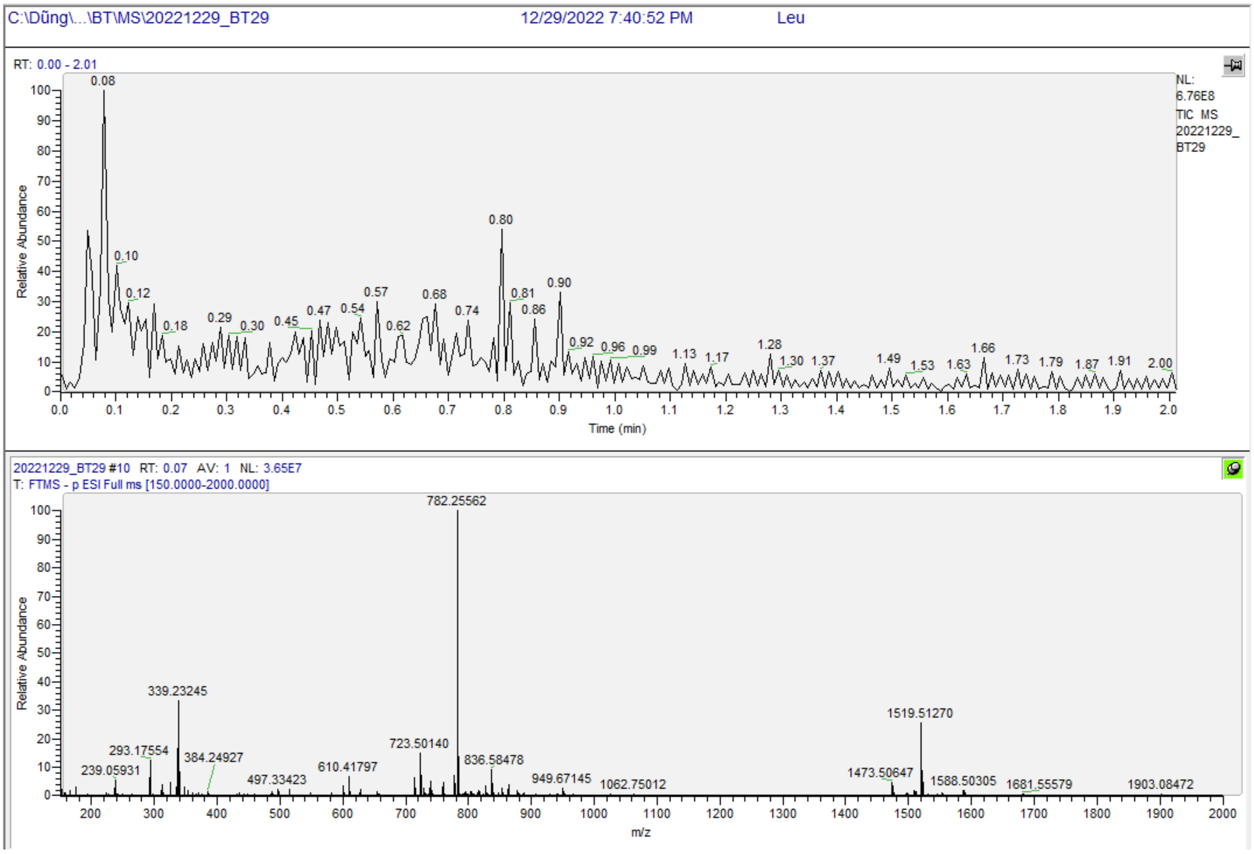

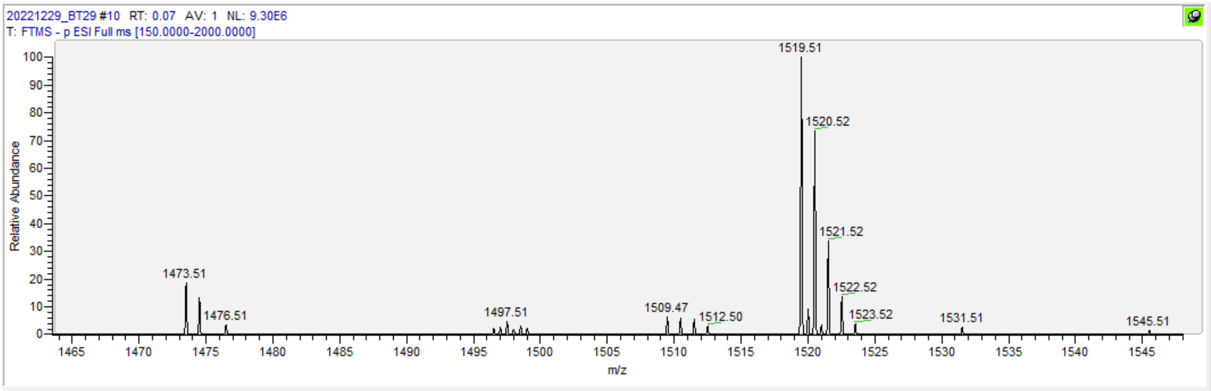

Elemental composition

Single mass

Mass: 1473.50647

Max. results 10 Calculate

Idx	Formula	RDB	Delta ppm
1	$C_{66} H_{89} O_{37}$	22.5	-0.848

Elemental composition

Single mass

Mass: 1519.51270

Max. results 10 Calculate

Idx	Formula	RDB	Delta ppm
1	$C_{67} H_{91} O_{39}$	22.5	-0.328

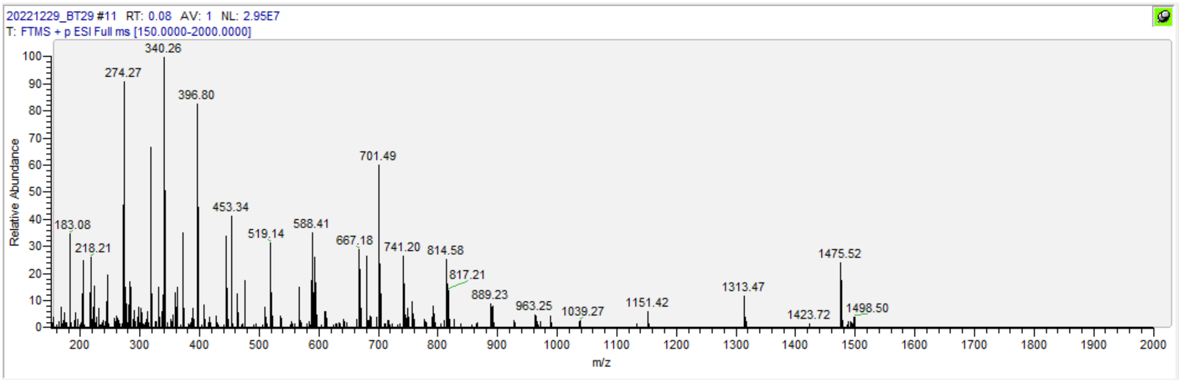

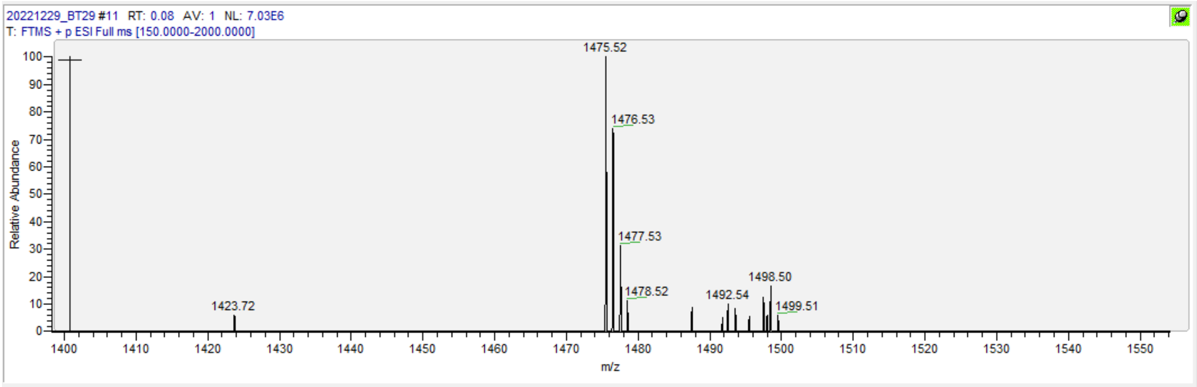

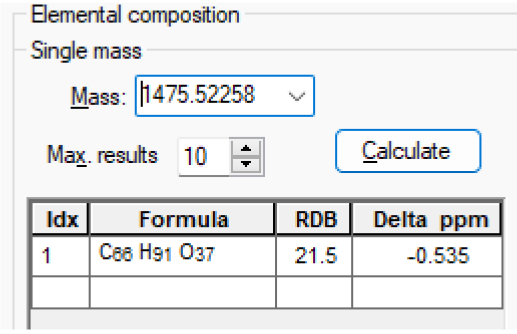

Figure S100. UV spectrum (in MeOH) of compound **10**

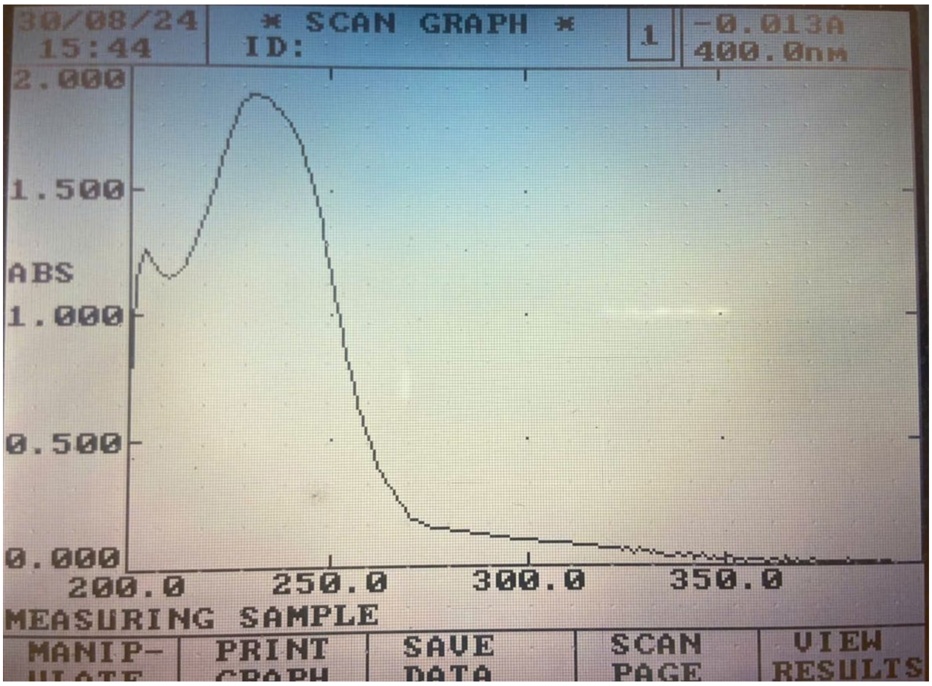

Figure S101. IR (ATR) spectrum of compound **10**

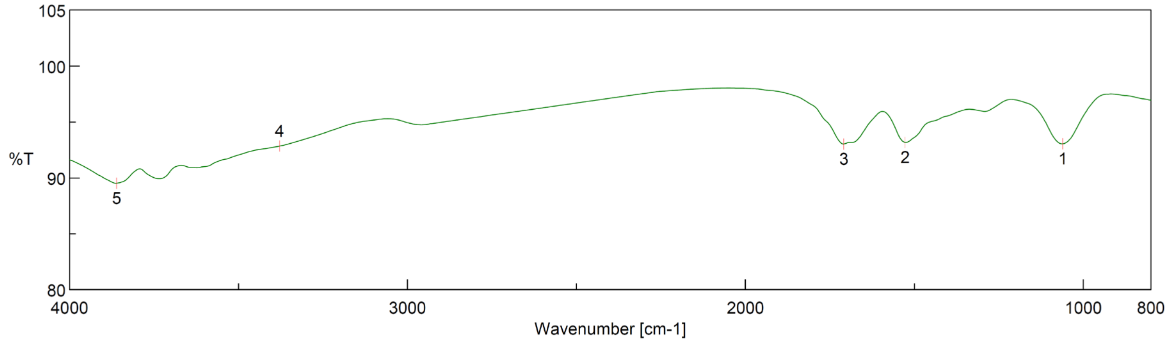

[Result of Peak Picking]

No.	Position	Intensity	No.	Position	Intensity	No.	Position	Intensity
1	1060.66	93.0322	2	1527.35	93.1584	3	1708.62	93.0186
4	3378.67	92.8327	5	3860.79	89.5067			

S2. UHPLC-MS chromatogram for monosaccharide identification of compounds 1–10

Figure S102. UHPLC-MS chromatogram of D-glucose-NAIM (4.92 min), D-galactose-NAIM (5.12 min), D-fructose-NAIM (5.84 min)

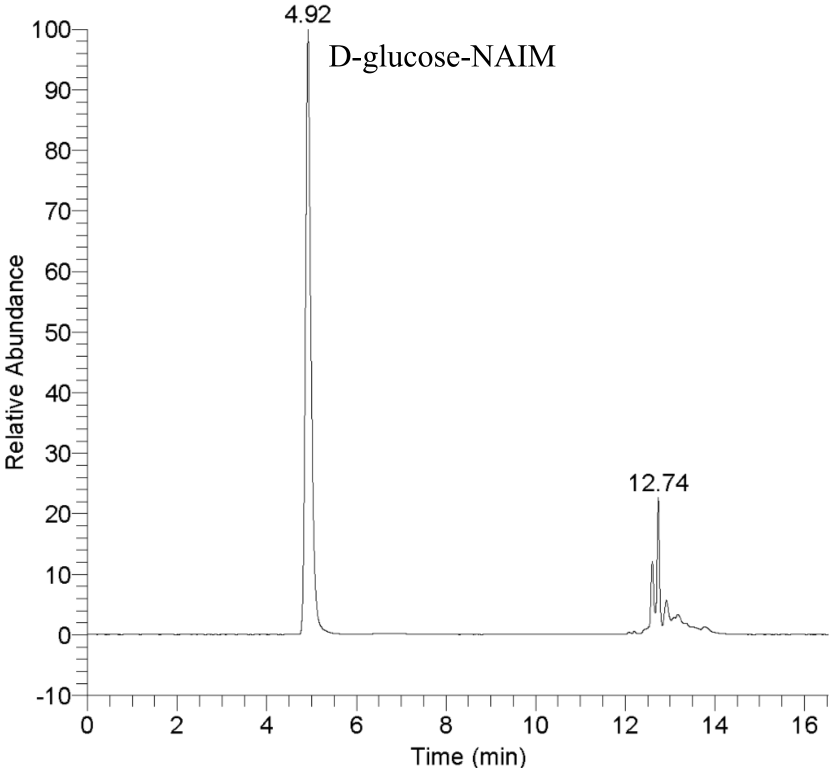

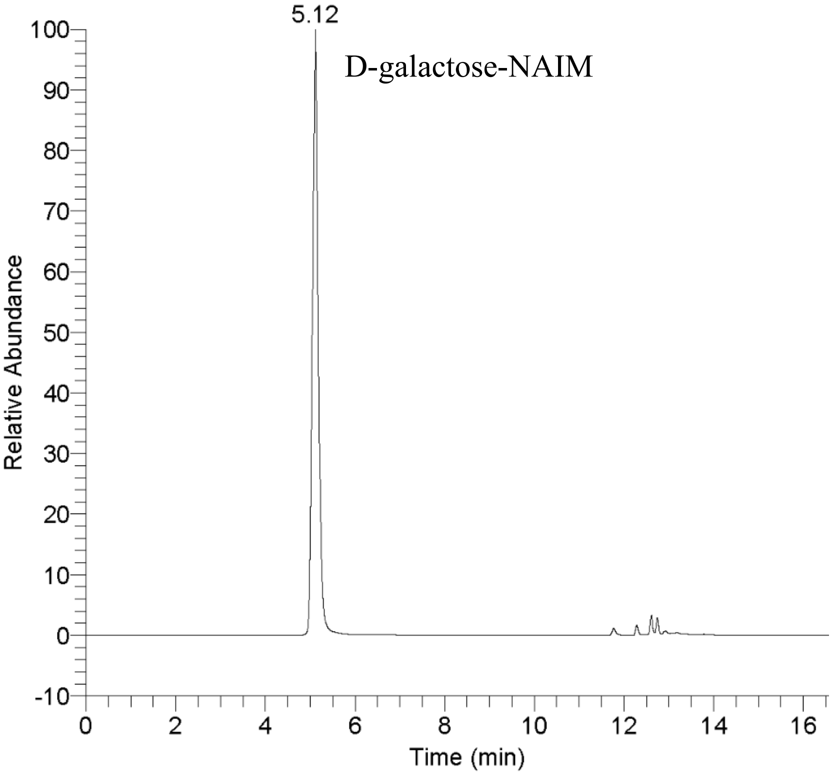

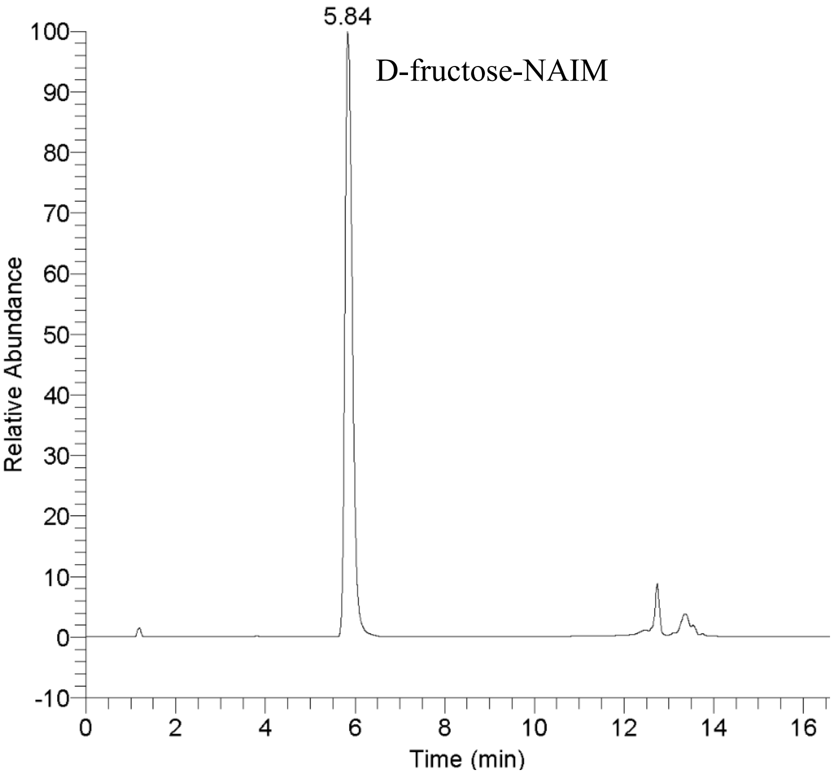

Figure S103. UHPLC-MS chromatogram of compound **1**'s sugar – NAIM

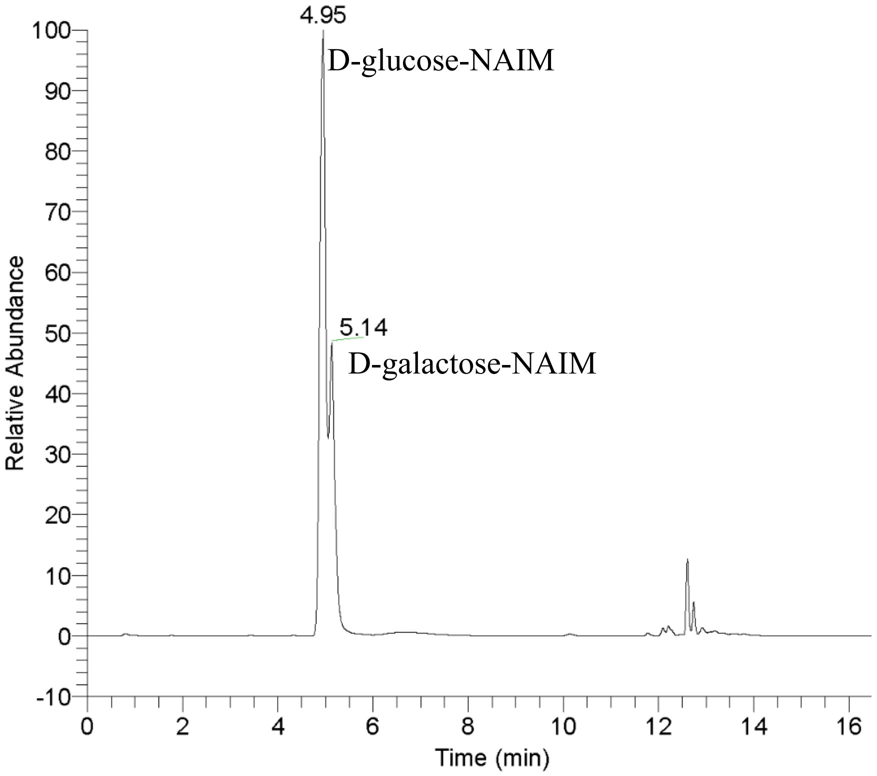

Figure S104. UHPLC-MS chromatogram of compound **2**’s sugar – NAIM

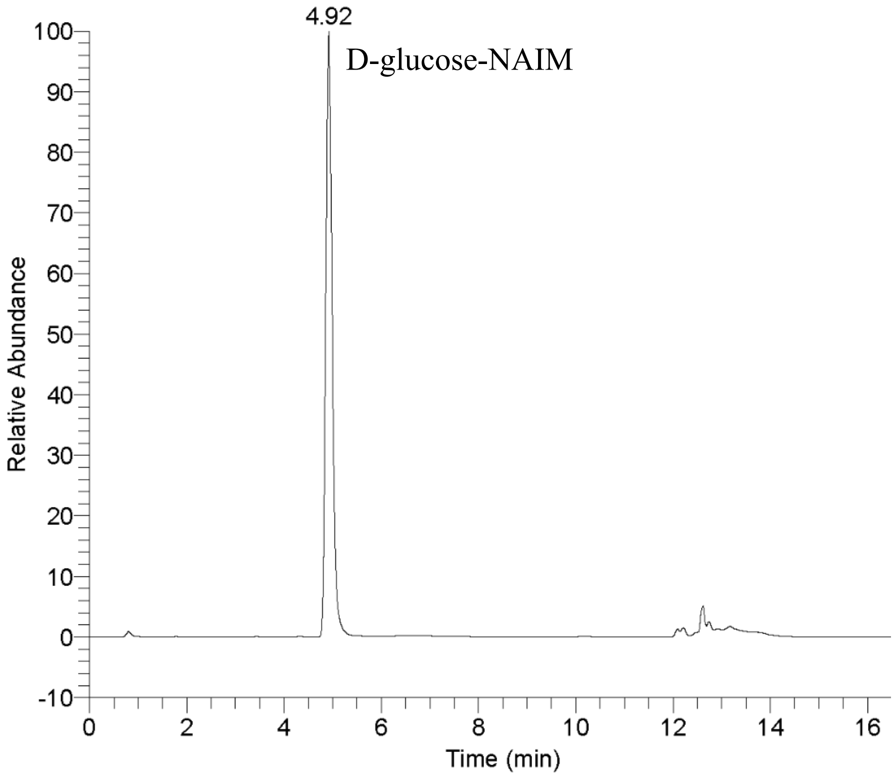

Figure S105. UHPLC-MS chromatogram of compound **3**’s sugar – NAIM

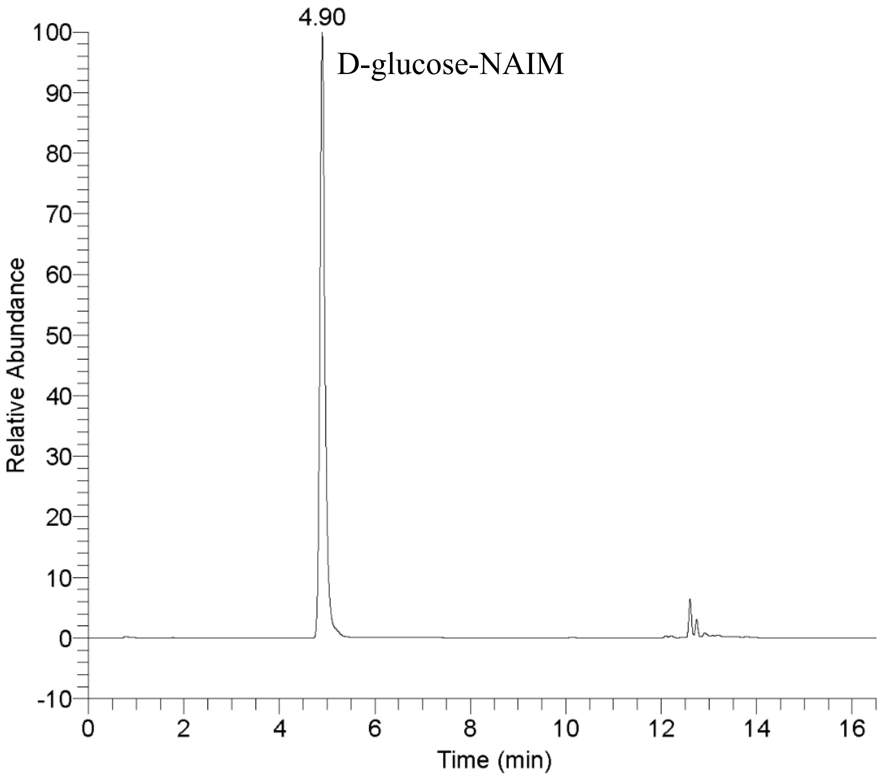

Figure S106. UHPLC-MS chromatogram of compound **4**'s sugar – NAIM

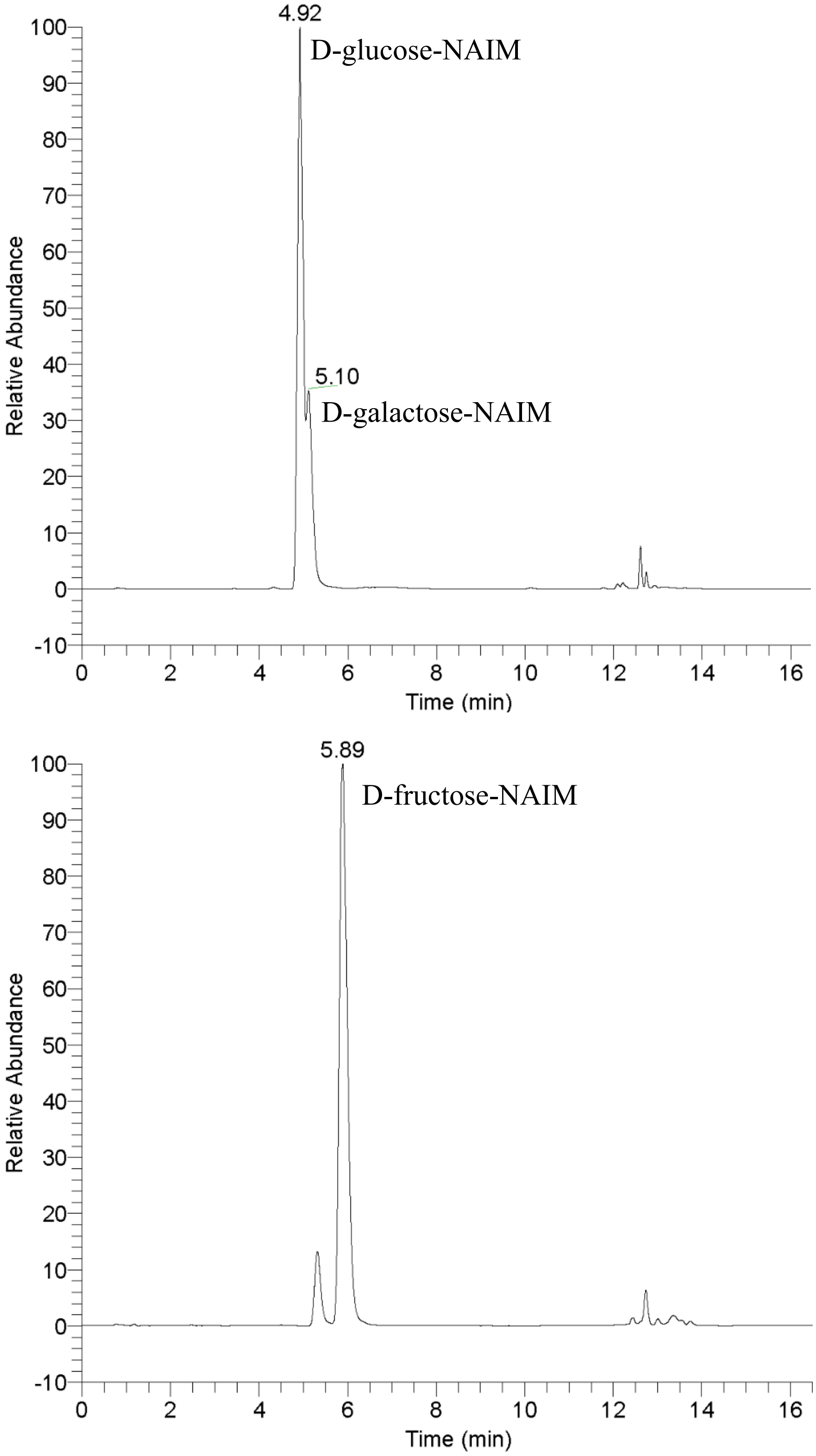

Figure S107. UHPLC-MS chromatogram of compound **5**'s sugar – NAIM

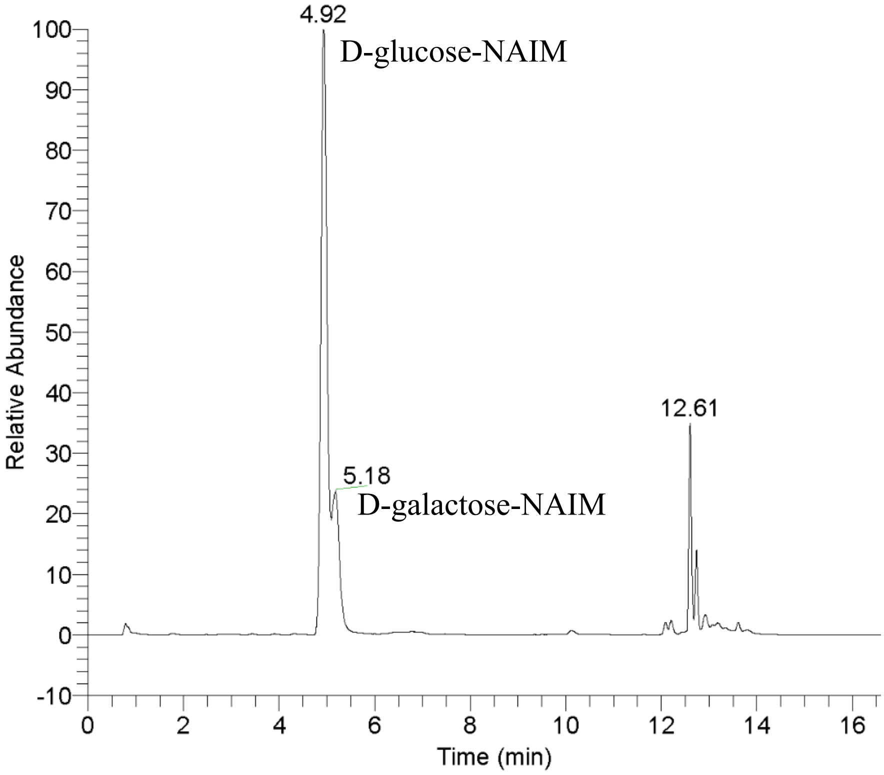

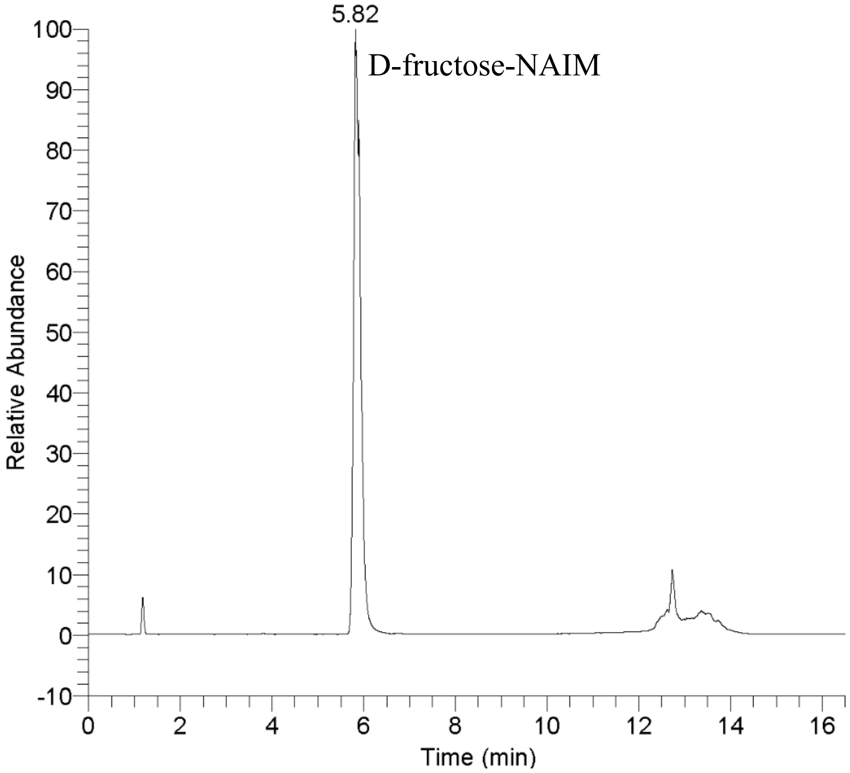

Figure S108. UHPLC-MS chromatogram of compound **6**'s sugar – NAIM

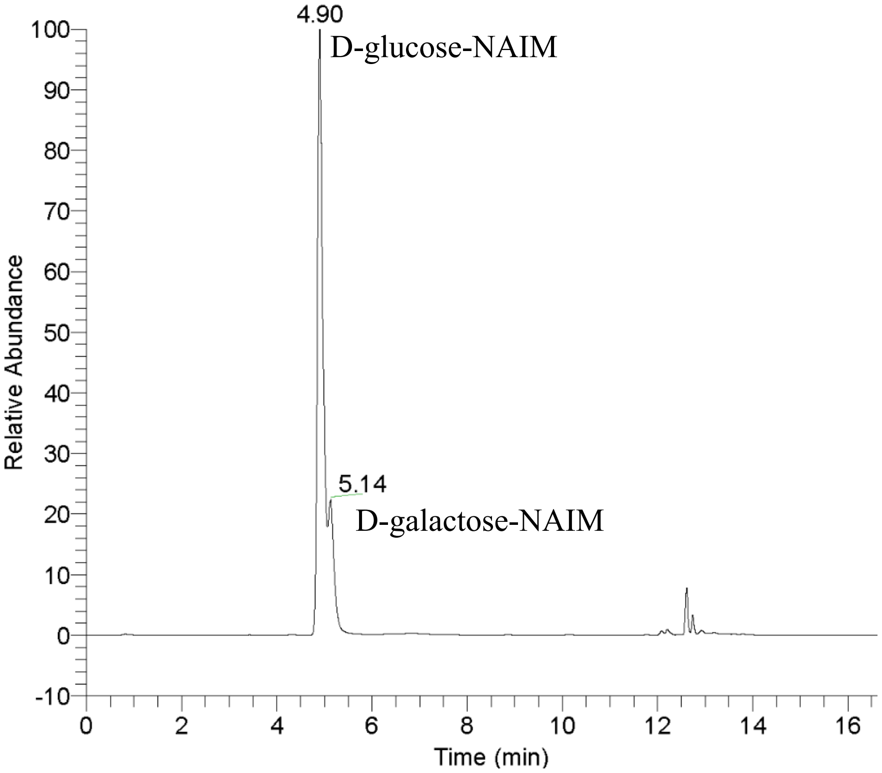

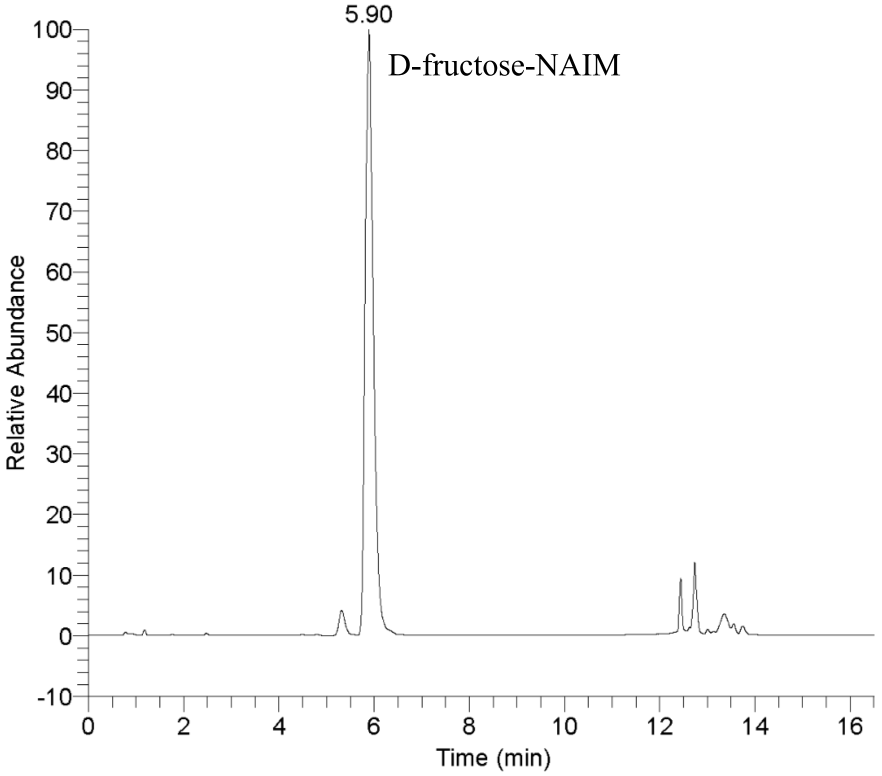

Figure S109. UHPLC-MS chromatogram of compound **7**'s sugar – NAIM

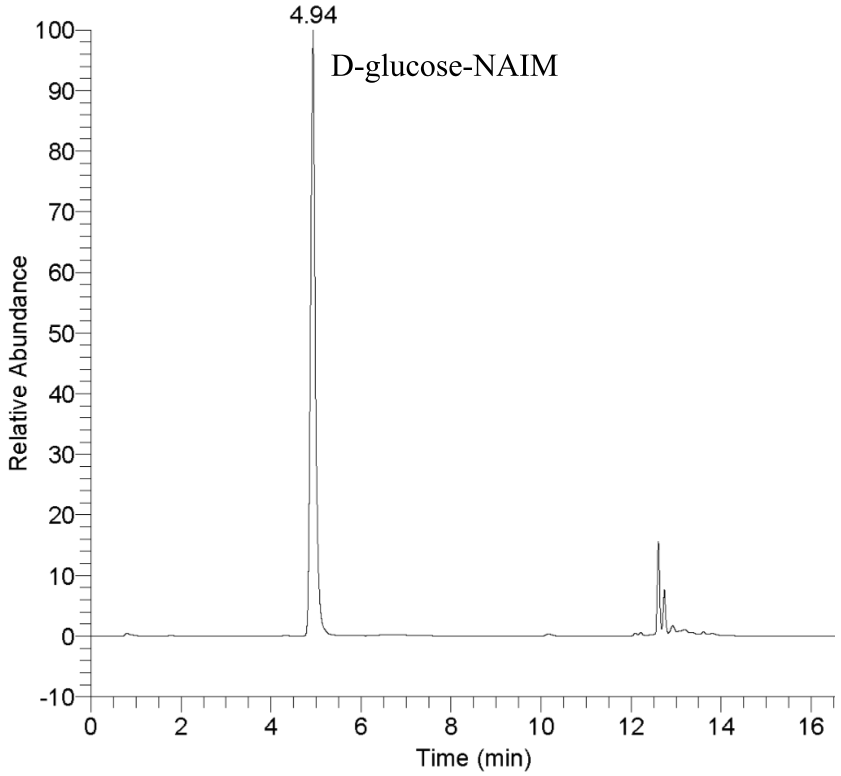

Figure S110. UHPLC-MS chromatogram of compound **8**'s sugar – NAIM

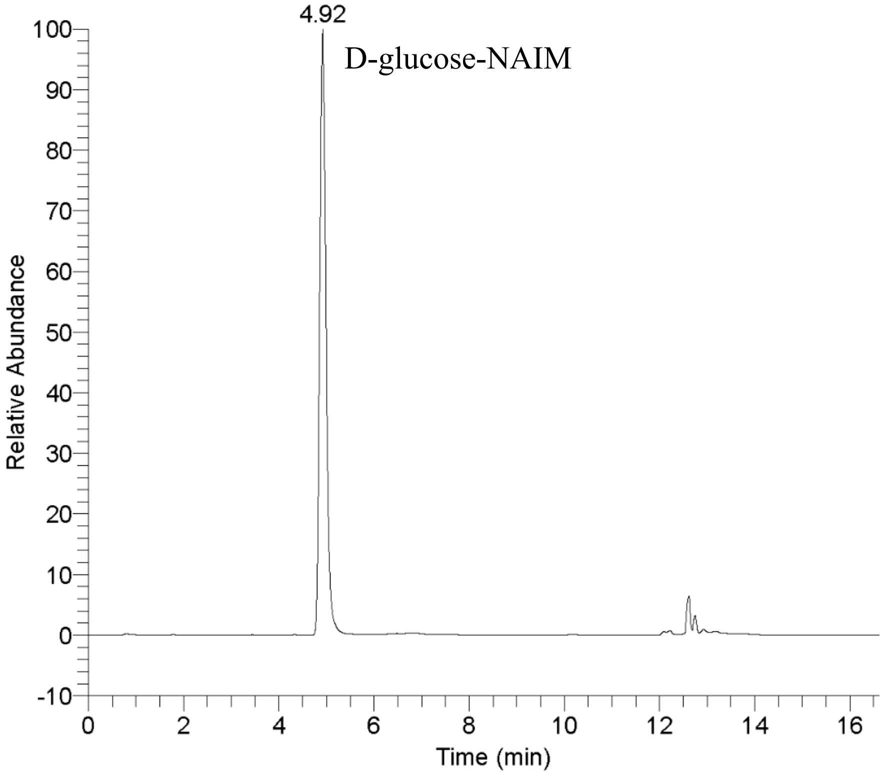

Figure S111. UHPLC-MS chromatogram of compound **9**'s sugar – NAIM

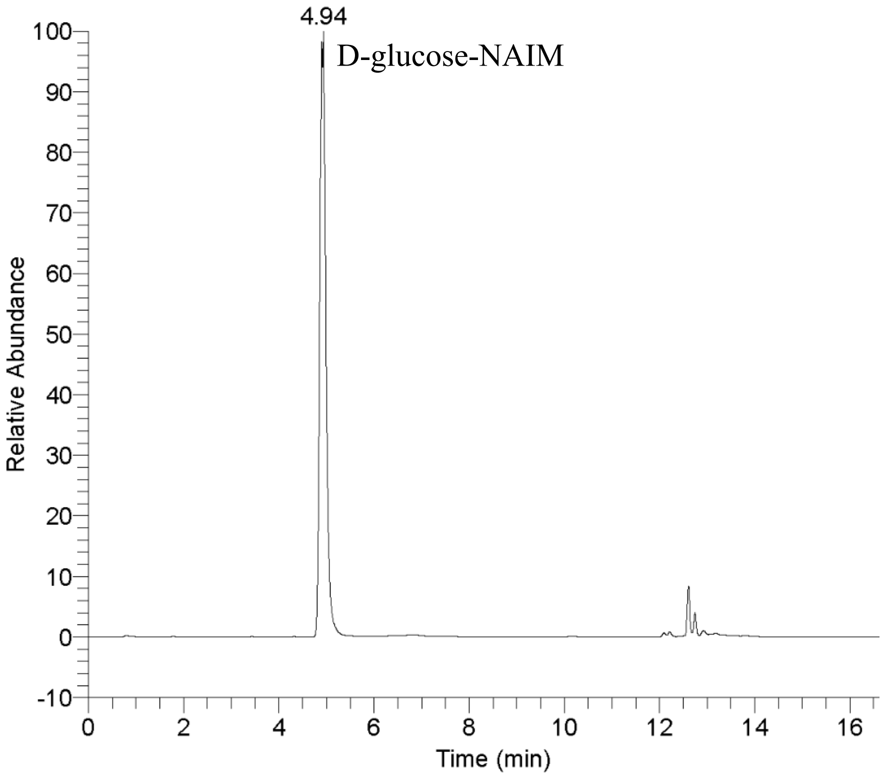

Figure S112. UHPLC-MS chromatogram of compound **10**'s sugar – NAIM

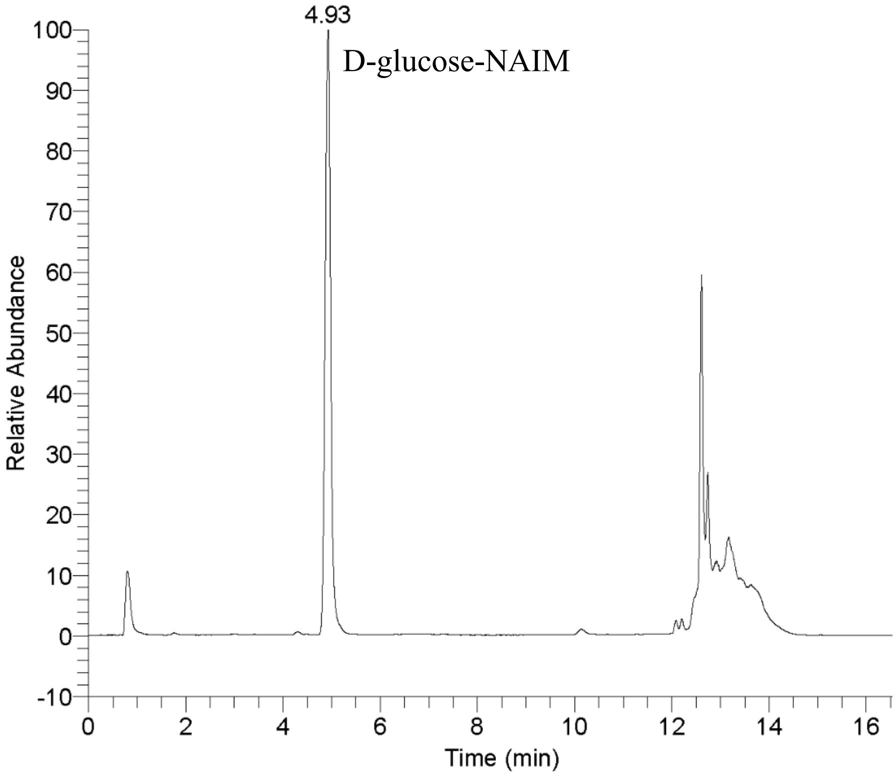

S3. NMR calculation and DP4+ analysis of compound 1

Figure S113. The energies and equilibrium populations of compound **1**'s stable conformers

No	Conformer	Structure	Gibb free energy (Hartree)	Population (%)
1	Cp1_(1*R*,5*R*,7*R*,8*S*,9*R*)-cf1	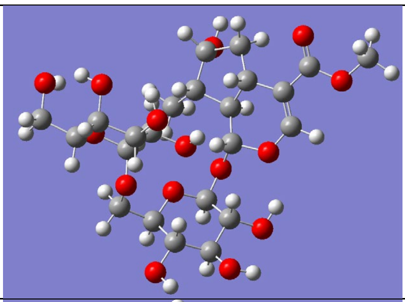	-2026.563172	59.2
2	Cp1_(1*R*,5*R*,7*R*,8*S*,9*R*)-cf2	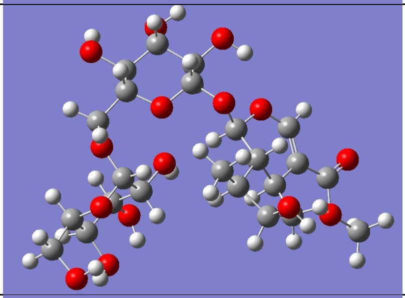	-2026.562819	40.8
3	Cp1_(1*S*,5*S*,7*S*,8*R*,9*S*)	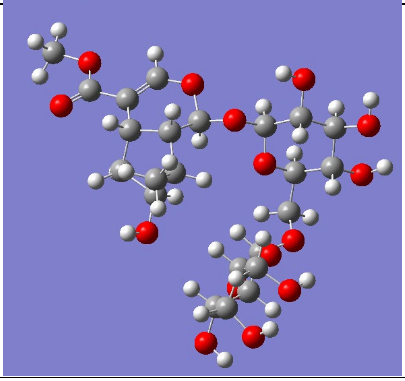	-2025.945710	100

Figure S114. Cartesian Coordinates of compound **1**'s stable conformers

Cp1_(1*R*,5*R*,7*R*,8*S*,9*R*)-cf1							
Atom	X	Y	Z	Atom	X	Y	Z
C	2.908017	-1.33398	0.269687	C	-3.28404	1.593852	1.042025
C	2.343074	-0.39014	1.34699	C	0.925263	-0.8062	3.510915
C	1.70441	0.81087	0.691569	O	-3.17985	0.696571	-0.06519
O	2.714228	1.484814	-0.15759	H	0.872618	0.539734	0.032922
C	3.686504	0.75732	-0.75012	H	4.320839	1.378564	-1.3714
C	3.864616	-0.57632	-0.61754	H	4.416789	-2.38645	1.443056
C	3.386829	-2.54018	1.105365	H	3.359841	-3.47074	0.53675
C	2.457688	-2.56105	2.358797	H	1.885788	-3.497	2.421421
C	1.493341	-1.34952	2.200636	H	0.658078	-1.69515	1.574808
O	3.205123	-2.37964	3.566626	H	3.824731	-3.11886	3.633101
H	2.062399	-1.67586	-0.34687	H	7.287558	-0.2493	-3.33836
H	3.173784	-0.01856	1.963319	H	7.390046	-1.68109	-2.26806
C	4.893293	-1.27492	-1.39963	H	6.269052	-1.68863	-3.65075
O	5.005219	-2.49138	-1.46107	H	0.23277	3.351985	2.137105
O	5.723229	-0.43488	-2.06795	H	0.489809	2.634612	-0.83578
C	6.7283	-1.0644	-2.88062	H	-0.8408	4.993157	0.562265
O	1.327341	1.762393	1.642043	H	-1.9876	2.690365	-1.08107
C	0.260793	2.626033	1.312753	H	-2.30449	3.298253	1.892951
C	0.440874	3.368068	-0.02068	H	2.353213	3.597509	-0.08957
C	-0.77689	4.253031	-0.25252	H	-1.82812	-0.44332	1.002274
C	-2.04095	3.400545	-0.24457	H	-1.46355	-2.03571	-0.81581
C	-2.13408	2.596066	1.060825	H	-3.44379	-0.38086	-2.43535
O	-0.92892	1.852317	1.288708	H	-4.89981	-2.32201	-2.30509
O	1.592739	4.195474	-0.01156	H	-5.15559	-0.8746	-0.44102
C	-2.56502	-0.53929	0.201911	H	-6.28571	-3.1074	-0.17552
C	-1.91975	-1.07069	-1.07888	H	-5.89097	-2.32373	1.369401
C	-2.98254	-1.33149	-2.15122	H	-4.01476	-3.68345	1.404258
C	-4.04789	-2.2925	-1.6074	H	-0.69898	-0.47513	-2.43311

C	-4.57453	-1.78661	-0.25137	H	-2.18579	-2.78598	-3.12858
O	-3.5093	-1.4971	0.67308	H	-3.8036	-4.04824	-0.77595
C	-5.46527	-2.79149	0.473813	H	0.134604	5.377528	-1.53573
O	-4.7374	-3.97725	0.827489	H	-3.07246	4.79198	-1.0984
O	-0.93214	-0.16201	-1.5448	H	-4.22188	2.142747	0.927333
O	-2.36263	-1.85105	-3.32577	H	-3.3168	1.042512	1.987942
O	-3.4295	-3.57785	-1.54552	H	0.401312	-1.59665	4.05875
O	-0.71525	4.91322	-1.51064	H	1.718804	-0.42298	4.156665
O	-3.2064	4.211002	-0.33513	H	0.218422	0.003476	3.315971
Cp1_(1*R*,5*R*,7*R*,8*S*,9*R*)-cf2							
Atom		Y	Z	Atom	X	Y	Z
C	3.054684	-0.9627	0.224864	C	-3.38256	1.35892	1.103435
C	2.43061	-0.01578	1.268932	C	1.142296	-0.43966	3.511875
C	1.645474	1.073494	0.577712	O	-3.24116	0.433592	0.024116
O	2.549411	1.799707	-0.34513	H	0.824733	0.683062	-0.0333
C	3.563696	1.137922	-0.93916	H	4.114113	1.772414	-1.62517
C	3.885724	-0.16091	-0.74814	H	4.706261	-1.76504	1.404224
C	3.69019	-2.05283	1.11511	H	3.749615	-3.01637	0.606795
C	2.808285	-2.09571	2.401434	H	2.340036	-3.08037	2.535851
C	1.71736	-1.00186	2.212435	H	0.901205	-1.46975	1.643506
O	3.569556	-1.76874	3.568976	H	4.259346	-2.44024	3.658508
H	2.228952	-1.43323	-0.33054	H	6.056081	-3.71125	-2.07442
H	3.239713	0.475999	1.827075	H	5.94821	-2.45057	-3.34246
C	4.973602	-0.69173	-1.58603	H	7.095934	-2.25472	-1.99652
O	5.703978	-0.02868	-2.31081	H	-0.02773	3.528631	1.950563
O	5.07984	-2.03783	-1.48582	H	0.17739	2.668742	-0.98793
C	6.115228	-2.64329	-2.28028	H	-1.32347	4.961653	0.345964
O	1.204991	2.03243	1.492187	H	-2.30108	2.470763	-1.12221
C	0.042328	2.763064	1.165384	H	-2.55669	3.197697	1.830392
C	0.090138	3.440709	-0.21257	H	1.96479	3.856196	-0.38565
C	-1.2198	4.186632	-0.4314	H	-1.71472	-0.50359	1.052721

C	-2.38944	3.215322	-0.3198	H	-1.22821	-2.0814	-0.75081
C	-2.34523	2.475039	1.025624	H	-3.46225	-0.71886	-2.31133
O	-1.06054	1.872738	1.236724	H	-4.66069	-2.82006	-2.08679
O	1.150748	4.378118	-0.30311	H	-5.0188	-1.3782	-0.23613
C	-2.46546	-0.70924	0.286884	H	-5.84717	-3.72854	0.114931
C	-1.81097	-1.18612	-1.01074	H	-5.49061	-2.86796	1.627912
C	-2.87576	-1.59918	-2.03183	H	-3.45677	-3.98119	1.6003
C	-3.79111	-2.67188	-1.42708	H	-0.73181	-0.47387	-2.42769
C	-4.32032	-2.2062	-0.05754	H	-1.94552	-2.96513	-3.0191
O	-3.26271	-1.76586	0.814807	H	-3.29704	-4.36618	-0.57903
C	-5.04689	-3.29841	0.722407	H	-0.4789	5.31858	-1.81366
O	-4.16175	-4.37603	1.063354	H	-3.59612	4.444527	-1.19298
O	-0.96324	-0.17334	-1.53453	H	-4.37757	1.802447	1.019143
O	-2.24477	-2.06352	-3.22346	H	-3.31004	0.846088	2.068777
O	-3.01702	-3.8703	-1.37223	H	0.724455	-1.2449	4.125453
O	-1.27798	4.778342	-1.72343	H	1.913518	0.061904	4.101237
O	-3.63424	3.900621	-0.39254	H	0.347546	0.279053	3.300216
Cp1_(1S,5S,7S,8R,9S)							
Atom	X	Y	Z	Atom	X	Y	Z
C	-3.54856	-1.17404	0.788305	C	2.036524	1.382543	-2.27337
C	-2.45648	-0.24292	1.388228	C	-1.29639	-1.96349	2.874356
C	-2.16495	0.907871	0.430753	O	3.018038	0.696589	-1.49648
O	-3.35903	1.667026	0.128876	H	-1.77439	0.554509	-0.52962
C	-4.4326	0.931726	-0.22785	H	-5.20195	1.53354	-0.6976
C	-4.56139	-0.39626	-0.01734	H	-3.04975	-3.28698	0.447884
C	-2.77409	-2.30769	0.043768	H	-2.99867	-2.3324	-1.02552
C	-1.27075	-2.0351	0.299582	H	-0.86326	-1.45639	-0.53545
C	-1.22374	-1.15152	1.574518	H	-0.30664	-0.55362	1.58896
O	-0.44592	-3.1954	0.333448	H	-0.78959	-3.79343	1.012284
H	-4.09422	-1.63012	1.620412	H	-8.37661	-0.25162	-2.18563
H	-2.78542	0.205505	2.332	H	-7.60677	-1.86796	-2.23429

C	-5.77285	-1.11064	-0.44932	H	-8.42041	-1.3196	-0.74883
O	-6.00334	-2.28067	-0.17712	H	-1.15302	3.074421	-0.58861
O	-6.61844	-0.35223	-1.1875	H	0.839798	3.04791	1.735509
C	-7.83113	-0.99927	-1.61121	H	0.624602	4.969048	-0.62853
O	-1.25934	1.789687	1.029149	H	2.757716	2.868259	-0.03583
C	-0.49848	2.57235	0.141276	H	0.603747	2.951611	-2.20051
C	0.263067	3.599762	0.979272	H	-1.16186	4.07022	2.214422
C	1.222639	4.371834	0.07869	H	1.542419	-0.63773	-0.93351
C	2.108094	3.423258	-0.72765	H	4.362835	-1.50999	-1.71067
C	1.236732	2.408562	-1.47972	H	1.939777	-3.22934	-0.98295
O	0.410323	1.713814	-0.5338	H	3.148231	-3.83625	1.020548
O	-0.60771	4.546671	1.580009	H	1.809967	-1.89554	1.234011
C	2.637362	-0.53349	-0.93525	H	3.360675	-2.30982	3.172122
C	3.278636	-1.69532	-1.69432	H	2.902154	-0.60179	2.998055
C	3.01638	-3.02395	-0.9757	H	4.929575	-0.32981	1.906328
C	3.504817	-2.95006	0.471821	H	3.007032	-2.58804	-3.37163
C	2.891285	-1.71028	1.147696	H	4.581877	-4.02492	-1.48629
O	3.129101	-0.50806	0.398093	H	5.245035	-2.41134	1.178325
C	3.458284	-1.42629	2.536293	H	1.526703	5.845854	1.30462
O	4.857252	-1.11331	2.473965	H	3.298348	4.868449	-1.19261
O	2.743225	-1.72745	-3.01016	H	2.581294	1.914677	-3.05717
O	3.635398	-4.0896	-1.69452	H	1.352707	0.675087	-2.75232
O	4.930071	-2.94516	0.424454	H	-0.43501	-2.63208	2.967182
O	2.08786	5.219472	0.825228	H	-2.20453	-2.57368	2.935261
O	2.877897	4.144166	-1.6798	H	-1.29	-1.29664	3.741733

Figure S115. Comparison of the Calculated and Experimental Proton and Carbon Resonances for compound **1**

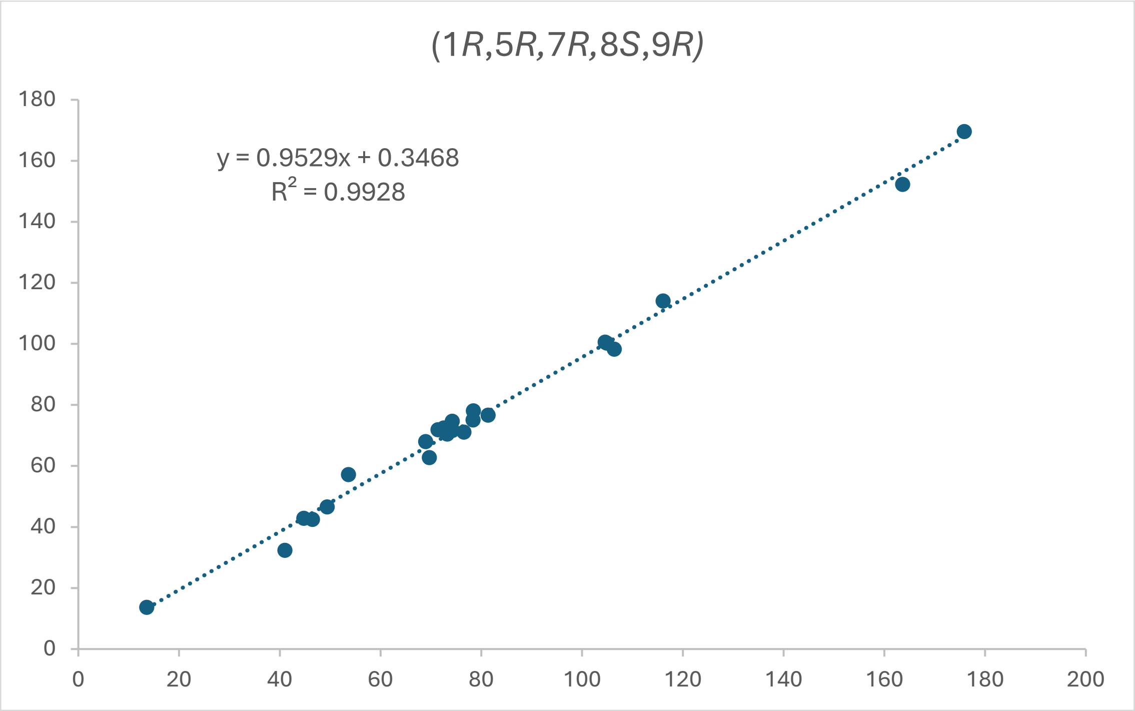

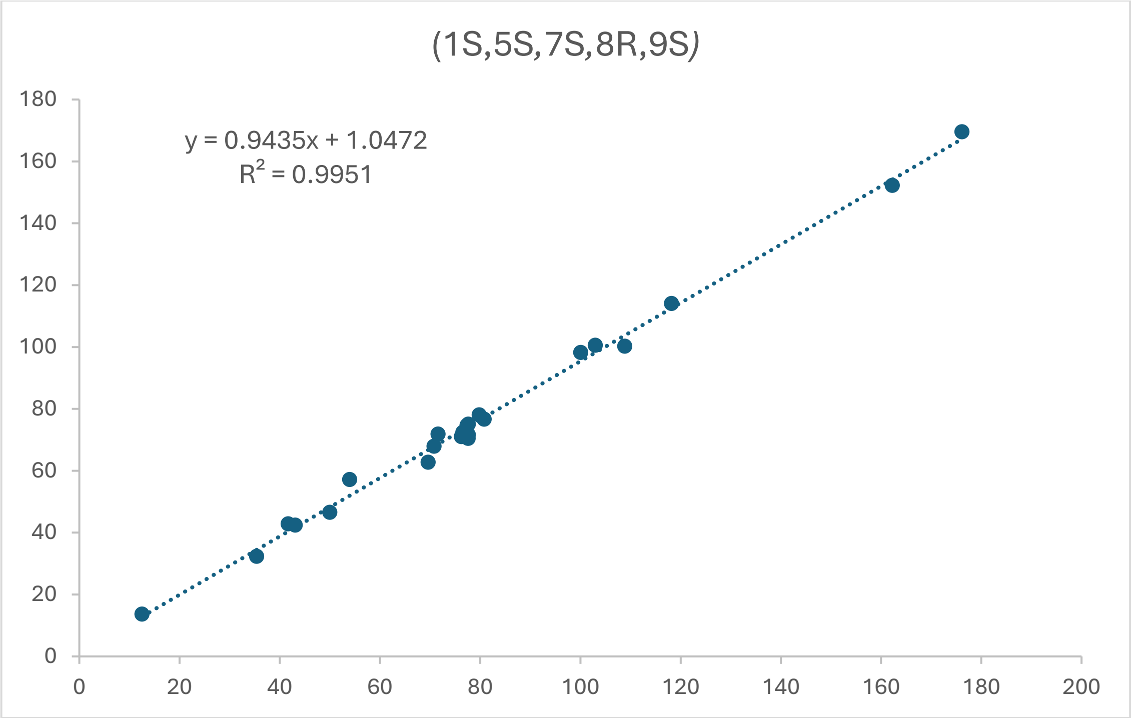

Figure S116. Assignment of the relative stereochemistry of **2** using DP4+ and representing the DP4+ probabilities for each candidate structure

Functional	Solvent?	Basis Set	Type of Data
mPW1PW91	PCM	6-311+G(d,p)	Shielding Tensors

		DP4+	0.02%	99.98%	-	-	-
**Nuclei**	**sp2?**	**Experimental**	**Isomer 1**	**Isomer 2**	**Isomer 3**	**Isomer 4**	**Isomer 5**
C	x	169.5	12.1	11.8			
C		57.1	134.4	134.0			
C		100.5	83.4	85.0			
C		74.6	113.8	110.6			
C		32.3	147.0	152.6			
C		78	109.6	108.2			
C		71.8	116.6	116.4			
C		76.6	106.7	107.2			
C		100.2	83.1	79.1			
C		70.4	114.8	110.4			
C		71.6	113.7	110.4			
C		71	111.45	111.73			
C		72.4	115.44	111.45			
C		46.5	138.57	137.99			
C		62.7	118.31	118.38			
C		67.9	119.05	117.21			
C		13.6	174.40	175.49			
C		98.2	81.58	87.94			
C	x	152.2	24.35	25.73			
C	x	114	71.89	69.82			
C		42.8	143.24	146.31			
C		75	109.64	110.36			
C		42.4	141.51	144.91			

(1*R*,5*R*,7*R*,8*S*,9*R*) (1S,5S,7S,8R,9S)

Functional	Solvent?	Basis Set	Type of Data
mPW1PW91	PCM	6-311+G(d,p)	Shielding Tensors

	Isomer 1	Isomer 2	Isomer 3	Isomer 4	Isomer 5	Isomer 6
sDP4+ (H data)	-	-	-	-	-	-
sDP4+ (C data)	1.15%	98.85%	-	-	-	-
sDP4+ (all data)	1.15%	98.85%	-	-	-	-
uDP4+ (H data)	-	-	-	-	-	-
uDP4+ (C data)	1.94%	98.06%	-	-	-	-
uDP4+ (all data)	1.94%	98.06%	-	-	-	-
DP4+ (H data)	-	-	-	-	-	-
DP4+ (C data)	0.02%	99.98%	-	-	-	-
DP4+ (all data)	0.02%	99.98%	-	-	-	-

S4. NMR calculation and DP4+ analysis of compound 2

Figure S117. The energies and equilibrium populations of compound **2**'s stable conformers

No	Conformer	Structure	Gibb free energy (Hartree)	Population (%)
1	Cp2_7*R*-7"*R*	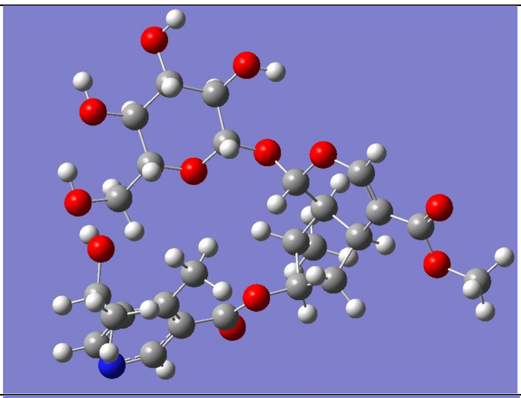	-1968.799838	100
2	Cp2_7*R*-7"S	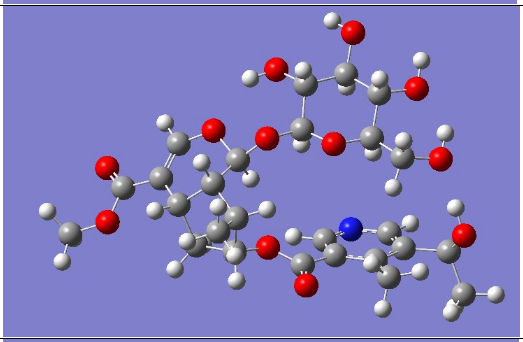	-1968.802234	100
3	Cp2_7*S*-7"*R*	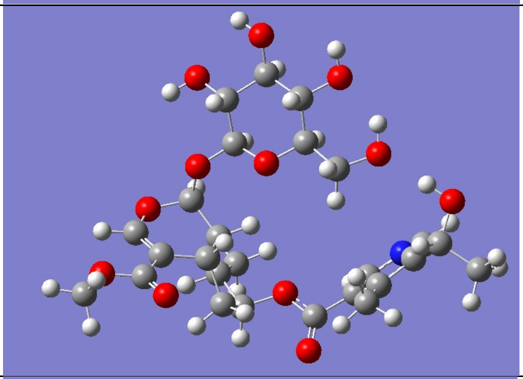	-1968.795654	100
4	Cp2_7*S*-7"*S*	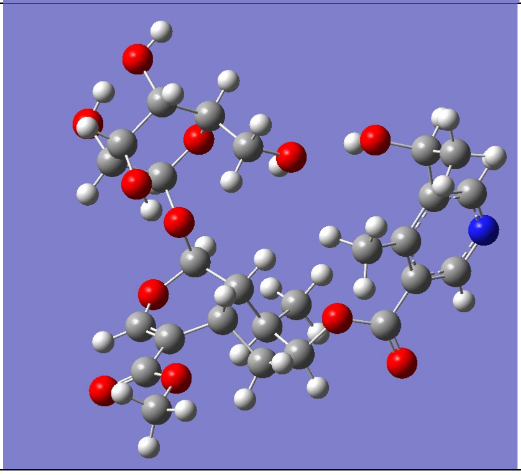	-1968.789006	100

Figure S118. Cartesian Coordinates of compound **2**'s stable conformers

Cp2_7*R*-7"*R*							
Atom	X	Y	Z	Atom	X	Y	Z
C	-1.57941	-3.03853	-0.69821	H	4.535408	-0.64246	-1.16214
C	-2.42097	-3.92026	-1.39423	H	3.172735	1.211086	-1.85073
N	-3.65865	-4.23716	-1.01598	C	-1.19493	-1.49546	1.318756
C	-4.10578	-3.63239	0.091908	O	-4.67344	0.629475	-0.12852
C	-3.37249	-2.74125	0.882214	H	-2.05083	-4.37786	-2.30682
C	-2.04521	-2.43666	0.498026	H	-5.13023	-3.868	0.378164
C	-4.08742	-2.17096	2.112568	H	-5.14676	-2.44982	2.002126
C	-3.59437	-2.7847	3.425213	H	-2.53312	-2.58298	3.592344
O	-3.98972	-0.75471	2.233553	H	-4.16108	-2.36136	4.259757
C	-0.26248	-2.79326	-1.37213	H	-3.7417	-3.86831	3.418626
O	-0.0568	-3.09624	-2.53738	H	-4.33359	-0.33474	1.417122
O	0.669582	-2.2189	-0.59115	H	2.200709	-2.70828	-1.88732
C	1.943299	-1.8841	-1.22104	H	3.64166	-2.54308	0.018853
C	2.973302	-1.68741	-0.09403	H	2.442358	-1.56183	0.854525
C	3.738993	-0.38984	-0.4544	H	0.807342	-0.22225	-2.05836
C	2.692401	0.471144	-1.20176	H	4.265181	2.113605	1.918277
C	1.852104	-0.54468	-2.01538	H	1.278623	0.572974	0.45737
C	4.350719	0.35985	0.70232	H	-0.14177	2.232821	0.866224
C	3.806263	1.515254	1.138993	H	-2.46993	1.548248	0.874716
O	2.669725	2.067579	0.6713	H	-3.08007	3.387061	-1.49645
C	1.849041	1.245011	-0.19526	H	-1.88196	4.081084	1.218681
O	0.993924	2.130683	-0.8583	H	-0.58377	4.056797	-1.54919
C	-0.22269	2.420205	-0.21558	H	1.245558	4.547194	-0.19174
O	-1.21203	1.573973	-0.78748	H	-1.74788	6.128518	0.150538
C	-2.50625	1.760633	-0.20481	H	-4.53086	4.257775	0.096935
C	-2.96737	3.209906	-0.4164	H	-2.9853	-0.22916	-0.87947
C	-1.93871	4.197197	0.124839	H	-3.65378	1.053947	-1.90157

C	-0.56136	3.89173	-0.46189	H	7.521424	-2.63419	0.818361
O	0.372051	4.748246	0.175406	H	8.095075	-1.00386	1.289411
O	-2.38978	5.501662	-0.21277	H	7.140289	-1.97174	2.438568
O	-4.22574	3.350485	0.24326	H	1.790107	-1.46001	-3.98141
C	-3.4554	0.755536	-0.86323	H	2.287094	0.243535	-3.99866
C	5.590829	-0.08262	1.362985	H	3.423294	-1.00515	-3.46937
O	6.143384	0.478975	2.298631	H	-0.34834	-2.03	1.758656
O	6.072628	-1.21935	0.806369	H	-0.77559	-0.71244	0.684214
C	7.286943	-1.73274	1.382848	H	-1.78267	-1.02661	2.102111
C	2.370908	-0.70034	-3.45147	H	-4.98726	1.536844	0.040824
Cp2_7*R*-7"*R*							
Atom	X	Y	Z	Atom	X	Y	Z
C	1.314924	-2.25078	-0.54771	H	-4.75193	-0.75807	1.374716
C	0.9647	-1.7431	-1.80858	H	-3.17816	0.856092	2.154917
N	1.847885	-1.3814	-2.73996	C	3.126143	-2.99017	1.106132
C	3.139309	-1.51409	-2.41168	O	4.868558	0.976488	0.865918
C	3.622031	-2.02222	-1.20078	H	-0.08354	-1.63368	-2.06688
C	2.683708	-2.42118	-0.22124	H	3.853938	-1.19359	-3.16903
C	5.144613	-2.12814	-1.0611	H	5.566345	-1.56725	-1.91
C	5.642129	-3.57101	-1.17811	H	5.349553	-4.00111	-2.14003
O	5.658923	-1.59532	0.153221	H	5.236323	-4.19961	-0.38102
C	0.22378	-2.58045	0.418662	H	6.733724	-3.58636	-1.10697
O	0.342774	-3.27283	1.414431	H	5.380225	-0.66047	0.244224
O	-0.94635	-1.99624	0.067291	H	-1.99995	-3.125	1.444479
C	-2.06882	-2.13575	0.991955	H	-4.07335	-2.7296	0.343307
C	-3.36324	-1.91666	0.181295	H	-3.12508	-1.90504	-0.88532
C	-3.95835	-0.55166	0.650242	H	-0.96808	-0.7168	2.227241
C	-2.80205	0.152288	1.40486	H	-4.40676	2.190839	-1.46962
C	-2.00775	-1.00743	2.05009	H	-1.52534	0.31055	-0.35755
C	-4.54266	0.330789	-0.42583	H	0.187736	1.479599	-0.62021

C	-3.95775	1.497286	-0.76759	H	2.598407	1.201051	-0.42773
O	-2.77855	1.938282	-0.28166	H	2.822641	3.670499	1.368503
C	-1.96064	0.952358	0.415895	H	1.708725	3.413992	-1.45733
O	-0.97536	1.673548	1.08096	H	0.244532	3.960942	1.168666
C	0.202883	1.977981	0.362169	H	-1.53712	3.690416	-0.3989
O	1.268136	1.48579	1.153446	H	1.224544	5.628826	-1.01362
C	2.550942	1.69239	0.556548	H	4.221547	4.296087	-0.38425
C	2.799411	3.197053	0.375674	H	3.274443	0.01422	1.693448
C	1.678659	3.831866	-0.43872	H	3.625324	1.588718	2.429673
C	0.319887	3.495749	0.174222	H	-7.87015	-2.47569	-0.71347
O	-0.66899	3.992153	-0.71187	H	-8.37147	-0.78311	-1.01816
O	1.92176	5.23219	-0.47181	H	-7.49522	-1.68608	-2.27749
O	4.062071	3.34712	-0.2756	H	-2.0482	-2.28599	3.807566
C	3.583329	1.039648	1.478496	H	-2.61691	-0.63546	4.106189
C	-5.82103	0.012762	-1.08829	H	-3.65807	-1.79291	3.267691
O	-6.36179	0.68054	-1.95841	H	2.557913	-2.55264	1.928961
O	-6.35142	-1.13945	-0.6155	H	2.923994	-4.06573	1.142322
C	-7.60412	-1.53872	-1.20074	H	4.186848	-2.81199	1.26281
C	-2.62229	-1.45679	3.383515	H	5.032708	1.843915	0.452914
Cp2_7S-7"*R*							
Atom	X	Y	Z	Atom	X	Y	Z
C	2.535661	-2.35523	-0.77164	H	-1.66128	-0.74578	1.0534
C	2.959076	-1.82465	-1.99742	H	-0.82139	-0.18758	-1.0656
N	4.053422	-1.07479	-2.14567	C	2.89786	-2.65151	1.744277
C	4.729365	-0.78153	-1.02649	O	3.280238	2.106998	0.688041
C	4.400396	-1.24647	0.250107	H	2.382628	-2.02871	-2.89644
C	3.288244	-2.10039	0.392792	H	5.579986	-0.11385	-1.13487
C	5.234126	-0.76471	1.435781	H	4.624791	-0.84812	2.346625
C	6.501929	-1.59886	1.62823	H	7.06898	-1.22085	2.483933
O	5.643673	0.587637	1.256148	H	6.25893	-2.65004	1.808213

C	1.234828	-3.10202	-0.74288	H	7.133964	-1.53831	0.736823
O	1.039329	-4.20073	-0.25614	H	4.836687	1.126596	1.127033
O	0.287873	-2.35222	-1.33253	H	-1.14174	-3.80035	-1.77379
C	-1.10439	-2.79871	-1.34003	H	-2.55134	-3.40821	0.128206
C	-1.71312	-2.7098	0.066927	H	-1.00177	-3.00502	0.837629
C	-2.2017	-1.23036	0.235276	H	-2.88566	-2.09391	-2.25482
C	-1.84549	-0.5624	-1.12043	H	-5.57948	-0.37008	-0.1643
C	-1.85708	-1.72532	-2.14258	H	-2.56474	1.008468	-2.45488
C	-3.69019	-1.13047	0.495606	H	-1.01904	2.4961	-1.68461
C	-4.50553	-0.4108	-0.29923	H	1.283535	2.568322	-0.95818
O	-4.1099	0.339509	-1.36054	H	0.483664	3.797381	1.730224
C	-2.72224	0.633867	-1.43824	H	0.010949	4.81556	-1.10503
O	-2.51949	1.706105	-0.50553	H	-1.97617	3.732441	0.948026
C	-1.33744	2.440081	-0.62999	H	-3.38099	4.078032	-0.83433
O	-0.31737	1.80829	0.147477	H	-1.17862	6.383548	0.12653
C	0.941157	2.485778	0.085032	H	1.924023	5.448361	0.945095
C	0.779106	3.894243	0.674679	H	1.916376	0.609113	0.478582
C	-0.31494	4.657783	-0.06457	H	1.661865	1.60747	1.923766
C	-1.60825	3.845582	-0.08213	H	-5.75951	-1.94566	3.868461
O	-2.5385	4.551918	-0.88774	H	-7.22469	-2.20609	2.872326
O	-0.48023	5.905226	0.596397	H	-5.93661	-3.44923	2.931384
O	2.037268	4.561779	0.572039	H	-1.91239	-0.68211	-4.05236
C	1.947314	1.631521	0.862117	H	-0.27549	-1.00616	-3.45738
C	-4.23199	-1.82436	1.677834	H	-1.25193	-2.31457	-4.144
O	-3.53978	-2.42422	2.487785	H	2.5776	-1.84594	2.41484
O	-5.57744	-1.73016	1.796546	H	2.089514	-3.37544	1.664793
C	-6.15168	-2.37836	2.945263	H	3.747543	-3.14746	2.222671
C	-1.28856	-1.41135	-3.52633	H	3.263465	3.06503	0.864255
Cp2_7S-7"S							
C	4.12857	-0.95109	-0.41701	H	-0.95593	-1.41411	1.222261

C	5.181705	-0.76338	-1.32145	H	0.103094	-0.44324	-0.64223
N	5.927173	0.341923	-1.381	C	2.822337	-0.16199	1.638075
C	5.600973	1.315585	-0.52283	O	0.802093	2.287281	-0.06392
C	4.600574	1.235611	0.453397	H	5.403144	-1.55021	-2.03758
C	3.84972	0.044683	0.54906	H	6.182211	2.233623	-0.60492
C	4.403011	2.467213	1.338545	H	4.823134	3.315694	0.774349
C	5.169484	2.37854	2.661438	H	4.987223	3.283028	3.249375
O	3.041986	2.739826	1.65221	H	6.243791	2.295351	2.475491
C	3.315606	-2.18667	-0.6535	H	4.854501	1.513885	3.252073
O	3.749762	-3.19638	-1.17987	H	2.491893	2.648568	0.853001
O	2.026522	-2.00541	-0.31206	H	1.511758	-3.8532	-1.1174
C	1.011252	-2.95356	-0.75979	H	-0.43192	-4.16344	0.27559
C	0.042834	-3.18739	0.402598	H	0.566704	-3.20339	1.35966
C	-1.02205	-2.03583	0.324602	H	-0.54892	-2.9832	-2.20334
C	-0.60423	-1.22368	-0.9369	H	-4.20404	-2.63888	-1.01641
C	0.160559	-2.23596	-1.82214	H	-1.48347	-0.10779	-2.58942
C	-2.43977	-2.54851	0.174722	H	-2.82666	1.582008	-2.45551
C	-3.21159	-2.22357	-0.88148	H	-0.86767	4.31177	-0.35187
O	-2.87105	-1.37257	-1.87897	H	-2.34842	3.962357	1.774135
C	-1.75802	-0.50567	-1.60934	H	-4.6257	3.067978	1.130566
O	-2.18514	0.549906	-0.7673	H	-4.6207	1.295106	-0.69543
C	-2.65075	1.756211	-1.3904	H	-4.19255	4.018806	-1.24568
O	-1.66073	2.758734	-1.33798	H	-2.92264	0.868703	1.037919
C	-1.37184	3.383321	-0.05998	H	-2.65694	5.625649	0.098637
C	-2.63471	3.753166	0.735401	H	-0.7928	1.632182	1.108285
C	-3.70129	2.635533	0.733303	H	-0.01067	3.144439	1.591477
C	-3.96704	2.173182	-0.71215	H	-2.00971	-4.46573	4.072961
O	-4.61389	3.171339	-1.48058	H	-3.70346	-4.00683	3.712483
O	-3.31828	1.56454	1.586647	H	-2.99074	-5.4406	2.934085
O	-3.28843	4.895302	0.161216	H	1.527218	-2.4125	-3.50158

C	-0.35204	2.558632	0.733162	H	0.260792	-1.21	-3.74205
C	-3.06378	-3.4283	1.184783	H	1.617482	-0.85217	-2.66589
O	-4.17223	-3.93791	1.102829	H	1.835595	0.165801	1.299834
O	-2.25822	-3.60297	2.257601	H	2.748838	-1.2153	1.908916
C	-2.78397	-4.43534	3.307678	H	3.067511	0.421326	2.522667
C	0.936139	-1.64182	-2.99733	H	0.48863	2.053796	-0.95081
C	4.12857	-0.95109	-0.41701	H	-0.95593	-1.41411	1.222261

Figure S119. Comparison of the Calculated and Experimental Proton and Carbon Resonances for compound **2**

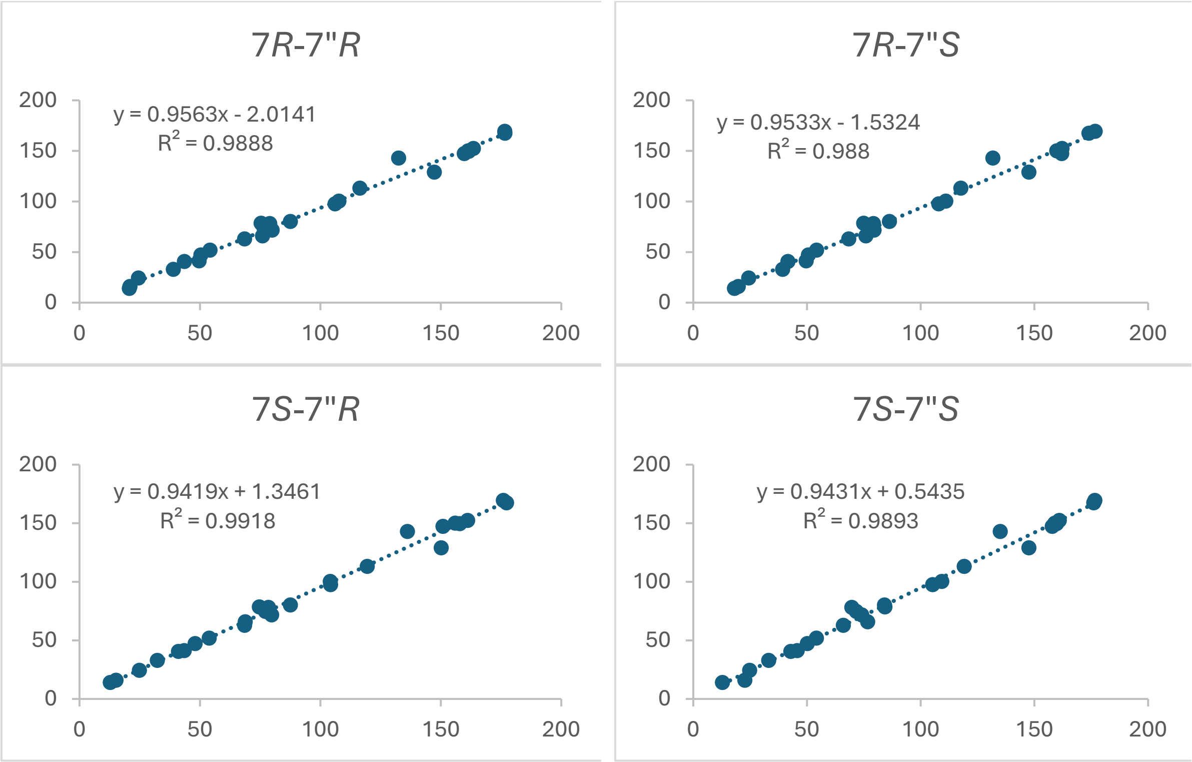

Figure S120. Assignment of the relative stereochemistry of **2** using DP4+ and representing the DP4+ probabilities for each candidate structure

Functional		Solvent?		Basis Set		Type of Data	
mPW1PW91		PCM		6-311+G(d,p)		Shielding Tensors	
		DP4+	0.00%	0.00%	100.00%	0.00%	-
Nuclei	sp2?	Experimental	Isomer 1	Isomer 2	Isomer 3	Isomer 4	Isomer 5
C	x	167.3	11.3	14.0	10.7	11.9	
C		80.1	100.4	101.7	100.4	103.9	
C		40.5	144.4	146.4	146.8	145.1	
C		32.8	149.0	148.7	155.6	154.8	
C		47.1	137.6	137.3	139.9	137.9	
C		41.1	138.3	138.4	144.5	142.3	
C	x	113	71.6	70.3	68.5	68.9	
C	x	152.3	24.5	25.8	26.8	27.0	
C	x	142.8	55.5	56.2	51.8	53.0	
C		97.5	81.9	80.0	83.7	82.7	
C		100.2	80.2	77.0	83.8	78.7	
C		78.4	112.57	113.07	113.33	103.70	
C		71.6	107.95	108.47	108.19	113.92	
C		78	108.98	108.75	109.49	118.29	
C		74.7	111.51	111.36	110.79	116.28	
C		150	26.60	28.16	32.01	28.97	
C		62.8	119.38	119.59	119.42	121.96	
C	x	169.3	11.44	11.28	12.00	11.36	
C		51.8	133.80	133.78	134.12	133.83	
C		14	167.27	169.83	175.16	175.17	
C		15.8	167.02	168.14	172.78	165.27	
C	x	149.5	26.39	27.14	30.09	28.22	
C	x	128.9	40.66	40.41	37.79	40.42	
C	x	147.2	28.21	25.96	37.05	30.08	
C		65.8	111.95	112.08	119.16	111.29	
C		24.3	163.4508	163.5716	163.1089	163.1935	
			(7*R*-7"*R*)	(7*R*-7"*S*)	(7*S*-7"*R*)	(7*S*-7"*S*)	

Functional	Solvent?		Basis Set		Type of Data	
mPW1PW91	PCM		6-311+G(d,p)		Shielding Tensors	
	Isomer 1	Isomer 2	Isomer 3	Isomer 4	Isomer 5	Isomer 6
sDP4+ (H data)	-	-	-	-	-	-
sDP4+ (C data)	0.00%	0.00%	100.00%	0.00%	-	-
sDP4+ (all data)	0.00%	0.00%	100.00%	0.00%	-	-
uDP4+ (H data)	-	-	-	-	-	-
uDP4+ (C data)	0.00%	0.00%	99.73%	0.27%	-	-
uDP4+ (all data)	0.00%	0.00%	99.73%	0.27%	-	-
DP4+ (H data)	-	-	-	-	-	-
DP4+ (C data)	0.00%	0.00%	100.00%	0.00%	-	-
DP4+ (all data)	0.00%	0.00%	100.00%	0.00%	-	-

S5. NMR calculation and DP4+ analysis of compound 3

Figure S121. The energies and equilibrium populations of compound **3**'s stable conformers

No	Conformer	Structure	Gibb free energy (Hartree)	Population (%)
1	Cp3_7b*R*_cf1	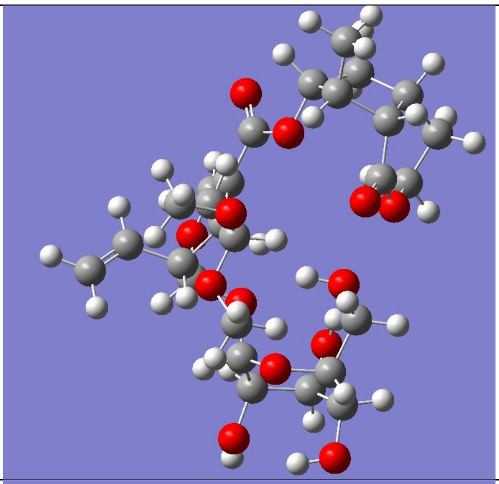	-2030.915114	5
2	Cp3_7b*R*_cf2	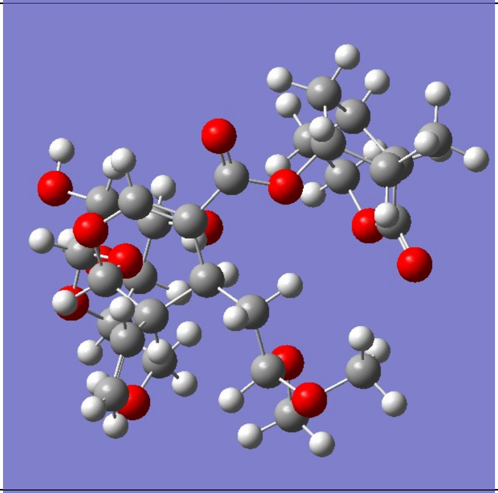	-2030.917893	95
3	Cp3_7bS	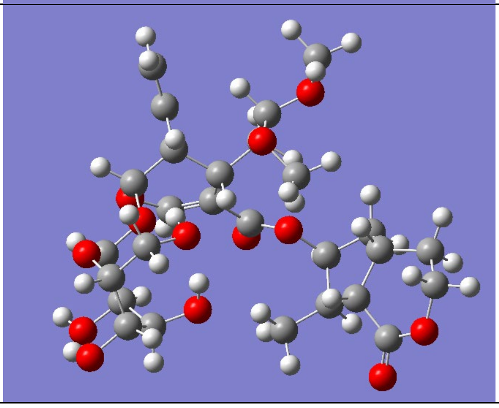	-2030.923615	100

Figure S122. Cartesian Coordinates of compound **3**’s stable conformers

Cp3_7b*R*_cf1							
Atom	X	Y	Z	Atom	X	Y	Z
C	-4.16609	-2.81223	0.106684	H	-4.7359	-1.98566	-1.8498
C	-4.09074	-1.72686	-1.00279	H	-5.13403	-3.31428	0.023259
C	-2.72347	-1.50208	-1.64949	H	-0.91851	-3.92218	-0.41549
O	-1.67203	-2.28093	-1.33337	H	-1.42862	-2.63911	0.684627
C	-1.7107	-3.20201	-0.20938	H	-3.03926	-4.54277	0.7941
C	-3.06593	-3.86303	-0.06465	H	-3.2664	-4.474	-0.95277
C	-4.12349	-2.03108	1.455893	H	-5.03103	-2.22091	2.035787
C	-4.04514	-0.53591	1.100912	H	-3.28447	-2.31999	2.093066
C	-4.60452	-0.42484	-0.32546	H	-4.5573	0.101469	1.823769
O	-2.56839	-0.6538	-2.51028	H	-4.20326	0.458394	-0.82968
O	-2.65645	-0.08827	1.023235	H	-6.51801	-0.29831	-1.3466
C	-6.13735	-0.34373	-0.32202	H	-6.47525	0.554378	0.204237
C	-2.04157	0.228867	2.188689	H	-6.5969	-1.20942	0.166079
C	-0.67815	0.771604	1.973527	H	0.044973	0.644136	-0.03157
O	-2.56043	0.046344	3.27975	H	1.614524	2.424277	0.048385
C	-0.19782	1.436439	0.689224	H	2.976354	1.835053	2.101581
C	1.130608	2.191582	1.000233	H	-0.14511	0.088307	3.923617
C	2.080122	1.28801	1.797596	H	3.865411	1.343674	0.170814
O	1.496933	0.837962	3.013393	H	4.865481	-0.42851	1.724968
C	0.173588	0.547642	2.99517	H	5.727285	-2.43537	0.363154
O	2.470292	0.113493	1.081057	H	4.111797	-3.22697	-1.43227
C	3.571428	0.290503	0.16589	H	3.112192	-1.24852	-2.70006
C	4.781888	-0.53366	0.637707	H	6.712661	-0.25401	0.379146
C	4.711438	-2.03364	0.265867	H	2.96442	-2.6912	0.859273
C	4.322795	-2.17225	-1.22447	H	5.757624	-0.96203	-1.69599
C	3.078106	-1.34443	-1.60979	H	1.647146	-2.92762	-1.79738
O	3.17936	0.029702	-1.15065	H	0.928872	-1.31632	-1.70325

O	5.900724	0.082233	-0.02663	H	1.460814	-1.34408	0.54751
O	3.899025	-2.78123	1.145863	H	-2.24725	2.034832	0.233342
O	5.423216	-1.8039	-2.05359	H	-1.1321	3.385627	0.415023
C	1.713998	-1.95968	-1.29181	H	0.3445	3.420686	2.680571
O	1.462219	-2.20969	0.095163	H	1.322892	5.546123	2.009464
C	-1.23448	2.372806	0.016741	H	2.059472	4.758136	0.505805
C	0.936551	3.481009	1.768326	H	-1.17113	1.379191	-1.90789
C	1.466384	4.651386	1.410927	H	-3.04838	4.858549	-2.54365
C	-1.10822	2.40024	-1.50666	H	-2.7908	4.570562	-0.80815
O	-2.18683	3.06566	-2.14494	H	-1.40909	4.988028	-1.85838
C	-2.35707	4.44242	-1.80763	H	0.594422	1.726773	-3.45297
O	0.13991	2.973938	-1.8395	H	-0.21946	3.255495	-3.88149
C	0.507971	2.793548	-3.20547	H	1.480253	3.271475	-3.33897
Cp3_7b*R*_cf2							
Atom	X	Y	Z	Atom	X	Y	Z
C	-3.2038	-2.9299	0.12807	H	-4.7718	-2.4521	-1.3237
C	-3.9691	-1.9215	-0.7963	H	-3.6068	-3.9348	-0.0325
C	-3.1708	-1.2836	-1.9307	H	-1.9987	-3.9257	-2.136
O	-2.0323	-1.8605	-2.3577	H	-0.4491	-3.0609	-1.9671
C	-1.5067	-3.0473	-1.7022	H	-1.1972	-2.0824	0.17632
C	-1.7078	-2.9734	-0.2024	H	-1.2373	-3.8435	0.2678
C	-3.511	-2.4613	1.57039	H	-4.3973	-2.9734	1.95923
C	-3.8327	-0.9693	1.45107	H	-2.6952	-2.6566	2.27084
C	-4.61	-0.8409	0.13434	H	-4.365	-0.5693	2.31536
O	-3.5599	-0.2851	-2.5096	H	-4.4663	0.15442	-0.2914
O	-2.5979	-0.2042	1.28615	H	-6.5328	-0.3196	1.02294
C	-6.1141	-1.0672	0.34188	H	-6.3271	-2.0582	0.75703
C	-1.9034	0.08721	2.41245	H	-6.6428	-0.9877	-0.6129
C	-0.5957	0.72436	2.12183	H	-0.0847	0.64217	0.04102
O	-2.3037	-0.2078	3.52936	H	1.56078	2.3567	0.01422

C	-0.2329	1.40855	0.80967	H	3.068	1.72649	1.89925
C	1.12507	2.14962	0.99625	H	0.08038	0.06109	4.03275
C	2.11434	1.22797	1.71182	H	4.02861	-0.3452	2.04861
O	1.63931	0.86123	3.01446	H	2.59139	-2.3725	1.59621
C	0.32746	0.53127	3.08802	H	3.05227	-3.5268	-0.6426
O	2.31586	0.058	0.94217	H	3.70132	-1.9789	-2.5289
C	3.60388	-0.5594	1.06411	H	5.34729	-0.1522	-1.6845
C	3.39428	-2.0711	0.9145	H	4.48251	-3.6056	1.46932
C	3.02216	-2.4369	-0.5348	H	1.49495	-1.2234	-0.3476
C	4.07878	-1.8591	-1.5078	H	5.50117	-2.6699	-0.4813
C	4.35645	-0.3638	-1.2729	H	2.32779	0.35053	-1.6628
O	4.5187	-0.0308	0.13874	H	3.44391	0.47262	-3.0258
O	4.6405	-2.6676	1.29052	H	3.96541	1.95164	-0.7134
O	1.69821	-2.0223	-0.8585	H	-2.2405	1.77715	0.12231
O	5.28165	-2.6184	-1.4284	H	-1.5476	3.14693	0.9899
C	3.35879	0.59015	-1.9403	H	0.46771	3.40756	2.70943
O	3.66725	1.94738	-1.6354	H	1.46266	5.50341	1.96649
C	-1.3273	2.35443	0.26706	H	2.10597	4.68231	0.43831
C	1.01187	3.44694	1.76718	H	-0.0964	3.70237	-0.9256
C	1.55278	4.5998	1.37116	H	0.18209	1.62016	-3.8043
C	-0.9499	3.02465	-1.0571	H	-0.6177	3.21021	-3.685
O	-0.6134	2.00587	-1.9745	H	0.98252	2.95411	-2.9316
C	0.01549	2.4863	-3.1612	H	-3.8291	3.17117	-0.9085
O	-1.9619	3.88408	-1.554	H	-3.754	3.94383	-2.5071
C	-3.2284	3.27541	-1.8209	H	-3.115	2.29355	-2.2896
Cp3_7b*S*							
Atom	X	Y	Z	Atom	X	Y	Z
C	-4.206	-0.2283	-0.1995	H	-2.727	-1.7637	-0.6634
C	-3.6025	-1.6647	-0.0072	H	-3.4897	0.35029	-0.791
C	-4.509	-2.8147	-0.4172	H	-4.6134	-1.1312	-2.7319

O	-5.3321	-2.6294	-1.4735	H	-6.3727	-1.3548	-2.6534
C	-5.46	-1.3063	-2.0576	H	-6.3644	-0.509	-0.294
C	-5.5323	-0.2547	-0.9628	H	-5.755	0.7229	-1.4023
C	-4.26	0.36431	1.22267	H	-5.2012	0.08734	1.71316
C	-3.1174	-0.3133	1.97531	H	-4.1856	1.45479	1.24088
C	-3.1476	-1.7712	1.47571	H	-3.1957	-0.2416	3.05947
O	-4.504	-3.9041	0.1243	H	-3.956	-2.2572	2.03525
O	-1.8693	0.32966	1.5751	H	-1.0322	-2.1685	1.11297
C	-1.8664	-2.5764	1.69281	H	-2.0235	-3.6109	1.3768
C	-0.9333	0.54182	2.53156	H	-1.5799	-2.5809	2.74909
C	0.32633	1.09343	1.97414	H	0.41311	0.98541	-0.1516
O	-1.0998	0.25396	3.70805	H	2.08803	2.63001	-0.5356
C	0.45789	1.76701	0.61464	H	3.93169	1.81054	0.96931
C	1.87181	2.41891	0.51585	H	1.34193	0.35921	3.70049
C	2.91611	1.40627	0.99117	H	4.52308	-0.3974	1.14908
O	2.7027	1.05933	2.36947	H	2.43037	-1.8348	1.57272
C	1.41552	0.82319	2.72393	H	2.33853	-3.8546	0.01629
O	2.81652	0.25136	0.18513	H	2.95926	-3.3397	-2.3995
C	3.84485	-0.749	0.36437	H	5.05862	-1.8607	-2.4746
C	3.1828	-2.0666	0.81107	H	4.64052	-2.5257	2.0689
C	2.51485	-2.8372	-0.3483	H	1.39595	-1.5804	-1.3562
C	3.50426	-2.9518	-1.532	H	4.82864	-3.6987	-0.3318
C	4.14525	-1.6034	-1.9282	H	3.15242	-1.2651	-3.7977
O	4.63944	-0.8668	-0.7781	H	3.94417	0.16841	-3.1207
O	4.18554	-2.9499	1.32772	H	2.22669	0.26962	-1.6087
O	1.25694	-2.3012	-0.7053	H	-1.628	2.27801	0.33137
O	4.5222	-3.9037	-1.233	H	-0.6913	3.56612	1.06724
C	3.33704	-0.7105	-2.8729	H	1.68626	3.66683	2.34691
O	2.05762	-0.2983	-2.3827	H	2.63303	5.71676	1.43557
C	-0.6615	2.78218	0.30329	H	2.87796	4.90144	-0.207

C	2.01585	3.69844	1.30985	H	0.32633	4.18808	-1.0375
C	2.53337	4.82651	0.82194	H	-2.086	2.063	-2.7324
C	-0.5066	3.47357	-1.056	H	-0.725	1.06231	-3.3107
O	-0.157	2.59768	-2.1198	H	-1.2971	0.89791	-1.6376
C	-1.129	1.60811	-2.4565	H	-2.6186	5.57409	-2.4388
O	-1.7142	4.15859	-1.2949	H	-0.8832	5.87436	-2.152
C	-1.637	5.10287	-2.3609	H	-1.3914	4.61877	-3.3125

Figure S123. Comparison of the Calculated and Experimental Proton and Carbon Resonances for compound **3**

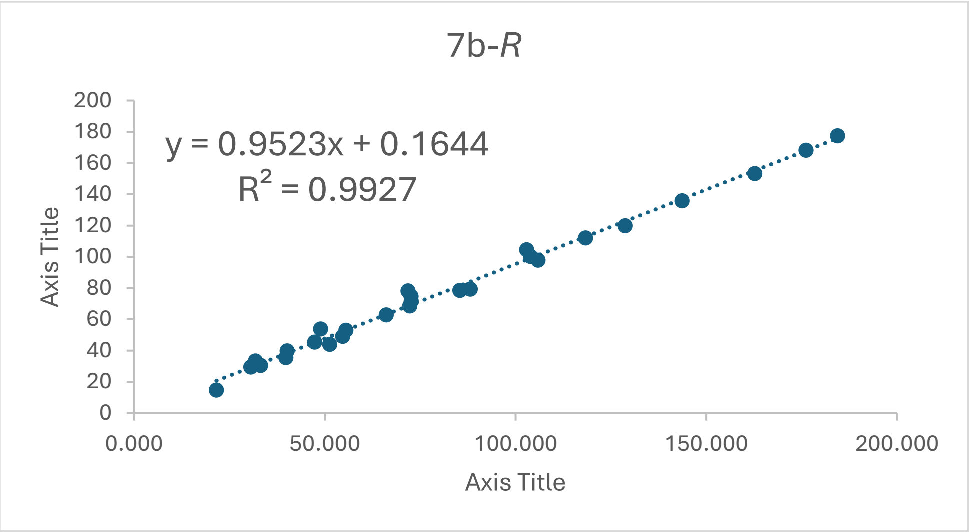

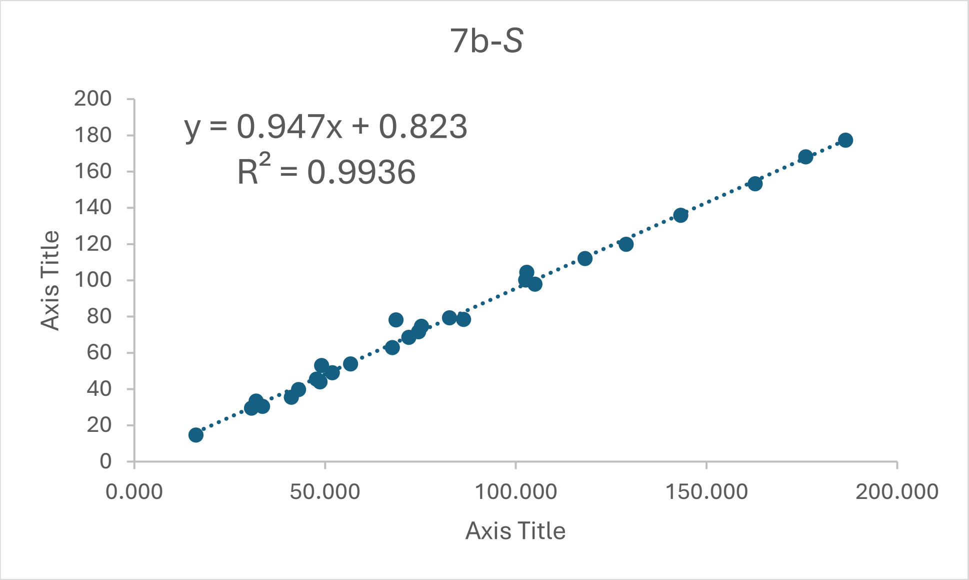

Figure S124. Assignment of the relative stereochemistry of **3** using DP4+ and representing the DP4+ probabilities for each candidate structure

Functional		Solvent?		Basis Set		Type of Data	
mPW1PW91		PCM		6-311+G(d,p)		Shielding Tensors	
		DP4+	0.00%	100.00%	-	-	-
Nuclei	sp2?	Experimental	Isomer 1	Isomer 2	Isomer 3	Isomer 4	Isomer 5
C		14.6	166.5	171.8			
C		168.1	11.9	12.0			
C	x	112	69.7	69.8			
C		29.4	157.4	157.3			
C		45.4	140.7	140.2			
C		97.8	82.1	83.0			
C		35.4	148.2	146.8			
C	x	153.2	25.3	25.2			
C		100.1	84.1	85.4			
C		74.6	115.4	112.7			
C		78.1	116.2	119.4			
C		71.6	115.37	113.47			
C		78.4	102.63	101.67			
C		49	133.33	136.10			
C		62.8	121.90	120.36			
C		33.3	156.20	156.09			
C	x	135.8	44.34	44.75			
C	x	119.8	59.25	59.06			
C		104.4	85.10	85.13			
C		52.9	132.51	138.91			
C	x	177.3	3.59	1.54			
C		53.8	139.13	131.31			
C		68.5	115.76	116.05			
C		30.4	154.87	154.40			
C		39.7	147.89	144.96			
C		79.3	99.87775904	105.3725			
C		43.9	136.7203824	139.3417			

(7b-*R*) (7b-S)

Functional	Solvent?		Basis Set		Type of Data	
mPW1PW91	PCM		6-311+G(d,p)		Shielding Tensors	
	Isomer 1	Isomer 2	Isomer 3	Isomer 4	Isomer 5	Isomer 6
sDP4+ (H data)	-	-	-	-	-	-
sDP4+ (C data)	0.00%	100.00%	-	-	-	-
sDP4+ (all data)	0.00%	100.00%	-	-	-	-
uDP4+ (H data)	-	-	-	-	-	-
uDP4+ (C data)	0.01%	99.99%	-	-	-	-
uDP4+ (all data)	0.01%	99.99%	-	-	-	-
DP4+ (H data)	-	-	-	-	-	-
DP4+ (C data)	0.00%	100.00%	-	-	-	-
DP4+ (all data)	0.00%	100.00%	-	-	-	-
